# Sustainable Antiparasitic Agents from an Agro-Industrial Waste: Mitochondria-Targeting Cashew Nutshell Liquid-Derived Phosphonium and Ammonium Salts

**DOI:** 10.1021/acs.jmedchem.5c01617

**Published:** 2025-09-08

**Authors:** Bianca Martinengo, Cecilia Baldassarri, Kayhan Ilbeigi, Hamed E. Alkhalaf, Aditya Sarode, Ehab Kotb Elmahallawy, Ba Reum Kwon, Amos Sarpong Agyei, Aigerim Abdimanova, Ludmila Ferreira de Almeida Fiuza, Raquel Azevedo, Ketlym da Conceição, Marcos Meuser Batista, Ellyêssa Nascimento Borges, Kleber Santiago Freitas e Silva, Éder Jéferson Souza Cardoso, Natália Cipriano Monteiro, Lais Flavia Nunes Lemes, Luiz Antonio Soares Romeiro, Antonio Alonso, Maria de Nazaré Correia Soeiro, Guy Caljon, Bryan W. Brooks, Harry P. De Koning, Maria Laura Bolognesi

**Affiliations:** † Department of Pharmacy and Biotechnology, 9296 Alma Mater Studiorum—University of Bologna, Via Belmeloro 6, Bologna 40126, Italy; ‡ Medicinal Chemistry Unit, School of Pharmacy, Chemistry Interdisciplinary Project (ChIP), 26660 University of Camerino, Via Madonna delle Carceri, Camerino 62032, Italy; § School of Infection and Immunity, College of Medical, Veterinary and Life Sciences, 3526 University of Glasgow, Glasgow G43 2DX, U.K.; ∥ Laboratory of Microbiology, Parasitology and Hygiene (LMPH), Infla-Med Centre of Excellence, University of Antwerp, 2610 Wilrijk, Belgium; ⊥ Department of Pure & Applied Chemistry, 3527 University of Strathclyde, 295 Cathedral Street, Glasgow G1 1XL, U.K.; # Department of Zoonoses, Faculty of Veterinary Medicine, 14643 Sohag University, Sohag 82524, Egypt; ¶ Grupo de Investigación en Sanidad Animal y Zoonosis (GISAZ), Unidad de Investigación Zoonosis y Enfermedades Emergentes (ENZOEM), Departamento de Sanidad Animal, 16735 Universidad de Córdoba, Córdoba 14071, Spain; ∇ Department of Environmental Science, Baylor University, Waco, Texas 76798-7266, United States; ○ Laboratório de Biologia Celular do Instituto Oswaldo Cruz, Fiocruz. Avenida Brasil 4365, Manguinhos, Rio de Janeiro CEP 21040360, Brazil; ⧫ Faculdade de Farmácia, 67824 Universidade Federal de Goiás, Goiânia, Goiás 74605-220, Brazil; †† Instituto de Física, Universidade Federal de Goiás, Goiânia, Goiás 74690-900, Brazil; ‡‡ Laboratório de Desenvolvimento de Inovaçõ Terapêutica, Centro de Medicina Tropical, Faculdade de Medicina, 28127 Universidade de Brasília, Brasília 70910-900, Brazil; §§ Department of Public Health, Baylor University, Waco, Texas 76798-7343, United States

## Abstract

Innovative, sustainable therapies are urgently needed for neglected vector-borne parasitic diseases. In this study, we leveraged cashew nutshell liquid (CNSL), an agro-industrial byproduct, to develop biobased phosphonium and ammonium salts (**5**–**25**) targeting parasite mitochondria. By combining CNSL-derived C8 alkyl chains with lipophilic cations, we synthesized novel compounds exhibiting highly potent *in vitro* and *ex vivo* activity against *Trypanosoma* and *Leishmania* spp., including veterinary-relevant strains like *T. b. evansi* and *T. b. equiperdum.* Compounds **5** and **7** outperformed reference drugs, demonstrating subnanomolar efficacy against *Trypanosoma brucei* spp., high selectivity indices (>1000), and no cross-resistance with current therapies, underscoring their potential as next-generation antitrypanosomal agents. Reduced activity against *T. brucei* overexpressing alternative oxidase and against *Trypanosoma congolense* supports a mitochondrial mechanism. Preliminary bioassays in zebrafish and *Daphnia magna* indicated ecotoxicity lower than antiparasitic activity. These CNSL-derived agents represent promising, environmentally safer antiparasitic candidates aligned with One Health and Green Chemistry principles.

## Graphical abstract

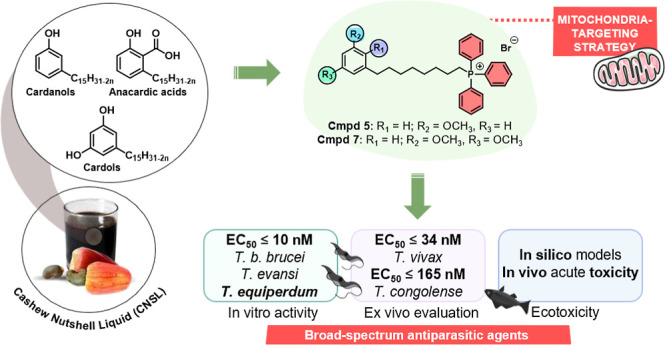

## Introduction

The discovery of new antiparasitic drugs is critical for controlling infectious diseases that severely impact human, animal, and environmental health, especially in low- and middle-income countries.^1^ Vector-borne parasitic diseases (VBPDs) caused by kinetoplastid parasites, such as *Trypanosoma brucei*, *Trypanosoma cruzi*, and *Leishmania* spp., pose challenges due to drug resistance, toxicity, complex treatment regimens, and poor accessibility.^2,3^ Trypanosomiases include several types of human and veterinary pathologies that are mainly endemic in Africa, the Americas and South-Eastern Asia.^4,5^ Although the recent introduction of fexinidazole as the first oral treatment for the treatment of human African trypanosomiasis (HAT) constitutes a major step forward in the control of HAT,^6^ challenges remain. Moreover, the positive trend seen with HAT has not yet been achieved for animal African trypanosomiasis (AAT). Current AAT therapies are inadequate or even inexistent, and infections continue to spread as nontsetse-transmitted animal trypanosomiasis (NTTAT) caused by *Trypanosoma vivax*, *T. b. evansi* and *T. b. equiperdum*.^5^

American trypanosomiasis or Chagas disease (CD) represents an ongoing risk to humans, dogs, and various wild or domestic mammals in regions across the Americas (including the Southern United States) where the infected triatomine bugs are found.^7^ The approved treatments—nifurtimox and benznidazole—have limited effectiveness in the later chronic stage and often cause severe side effects, leading many patients (20%) to discontinue the prolonged treatment regimen. Despite residing in major economies such as Argentina, Brazil, and Mexico, over 90% of patients—including pregnant women at risk of vertical transmission and their newborns—lack access to current therapies.^8^

Leishmaniasis is a VBPD caused by several *Leishmania* spp., manifesting as cutaneous (CL), muco-cutaneous (MCL) or visceral leishmaniasis (VL), the latter being potentially fatal if untreated.^9^ VL poses a significant public health burden in low-income regions all around the world and it is also increasingly endemic in Southern and South-Eastern Europe, including Spain, Italy, Portugal, Greece, France and Georgia^10^ due to environmental shifts including impacts of climate change on the distribution of the sand fly vector.^11^ Existing drugs generally have drawbacks, such as route of administration, the need for trained professionals for administration, toxicity, high cost^12^ in addition to high levels of resistance to some of the main drugs.^13^ Considering the continued expansion of leishmaniasis, the number of pathogenic species, increasing drug resistance and the fact that leishmaniasis will probably become an emerging zoonosis, the pharmacological arsenal needs to be improved urgently. Meanwhile, although barely studied, the environmental impact of many antileishmanial and antitrypanosomal drugs—accumulated over decades of parasite control efforts—is a growing concern.^14^

In the absence of licensed vaccines—aside from progress with canine leishmaniasis vaccines^15^— and given the limitations of current therapies, there is an urgent need for novel small-molecule therapeutics that prioritize sustainability^16^ to meet global targets for disease control and elimination.^17^ Herein, the integration of Green Chemistry and the One Health approach within drug discovery might offer a promising path forward.^18^ The principles of One Health recognize the interconnectedness of human and animal health, and environmental integrity,^19^ while Green Chemistry^20^ includes principles aimed at realizing renewable, low-impact sources, processes and products. Together, these frameworks support the development of accessible and environmentally responsible therapeutics, particularly for VBPDs.^16^

Motivated by these considerations, we have explored the discovery of new, inherently benign antiparasitic drugs starting from an agro-industrial waste, the cashew nutshell liquid (CNSL).^21–23^ It is a renewable, low-cost feedstock rich in phenolic lipids, with cardanols (**1**) being the highest-yield derivatives (60–70%), followed by cardols (**2**) (15–20%), and traces of anacardic acids (**3**) and 2-methylcardols (**4**) (Figure 1).

**Figure 1:**
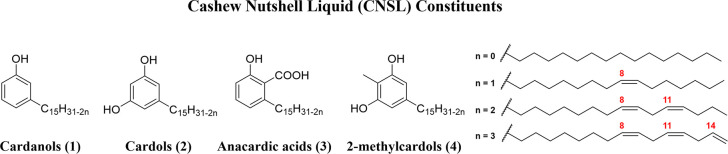
Chemical structures of cashew nutshell liquid (CNSL) constituents (**1**–**4**).

They carry a pentadecyl chain in meta position, which, depending on the manufacturing process, may be saturated, or mono-, di- or triolefinic (Figure 1).^24^ Importantly, CNSL production is concentrated in regions highly affected by VBPDs, such as Africa, South America, and Southeast Asia. Ideally, leveraging CNSL locally could promote pharmaceutical self-reliance, reduce environmental impacts, and foster circular bioeconomies.^25^

Despite known biological activity—including antimicrobial and antiparasitic effects—^26^ native CNSL components **1**-**4** often lack potency, necessitating medicinal chemistry exploration. Here, we report the synthesis and biological evaluation of a new class of CNSL-derived, mitochondria-targeting phosphonium and ammonium compounds (**5**–**25**), designed with sustainability principles. These derivatives were assessed for *in vitro* and *ex vivo* activity against various *Trypanosoma* and *Leishmania* strains and species, mammalian cytotoxicity, and for preliminary insights into their mechanisms of action. We also evaluated ecotoxicological profiles of these molecules to initially understand environmental hazards of new antiparasitic drugs.

## Results and Discussion

### Design

We aimed to leverage medicinal chemistry to enhance the antiparasitic activity of CNSL (Figure 2). To preserve the green and biobased nature of the resulting molecules, we pursued a ligand-based strategy that minimizes synthetic complexity and the number of reaction steps. We envisioned utilizing the unique scaffolds of compounds **1**–**3** as carriers for conjugation with a lipophilic cation, which—according to the Nernst equation—preferentially accumulates in mitochondria. Structural modification of different classes of compounds into mitochondria-targeting “vehicles” has proven highly effective, thanks to its simple chemical synthesis and efficient targeting.^27^

**Figure 2:**
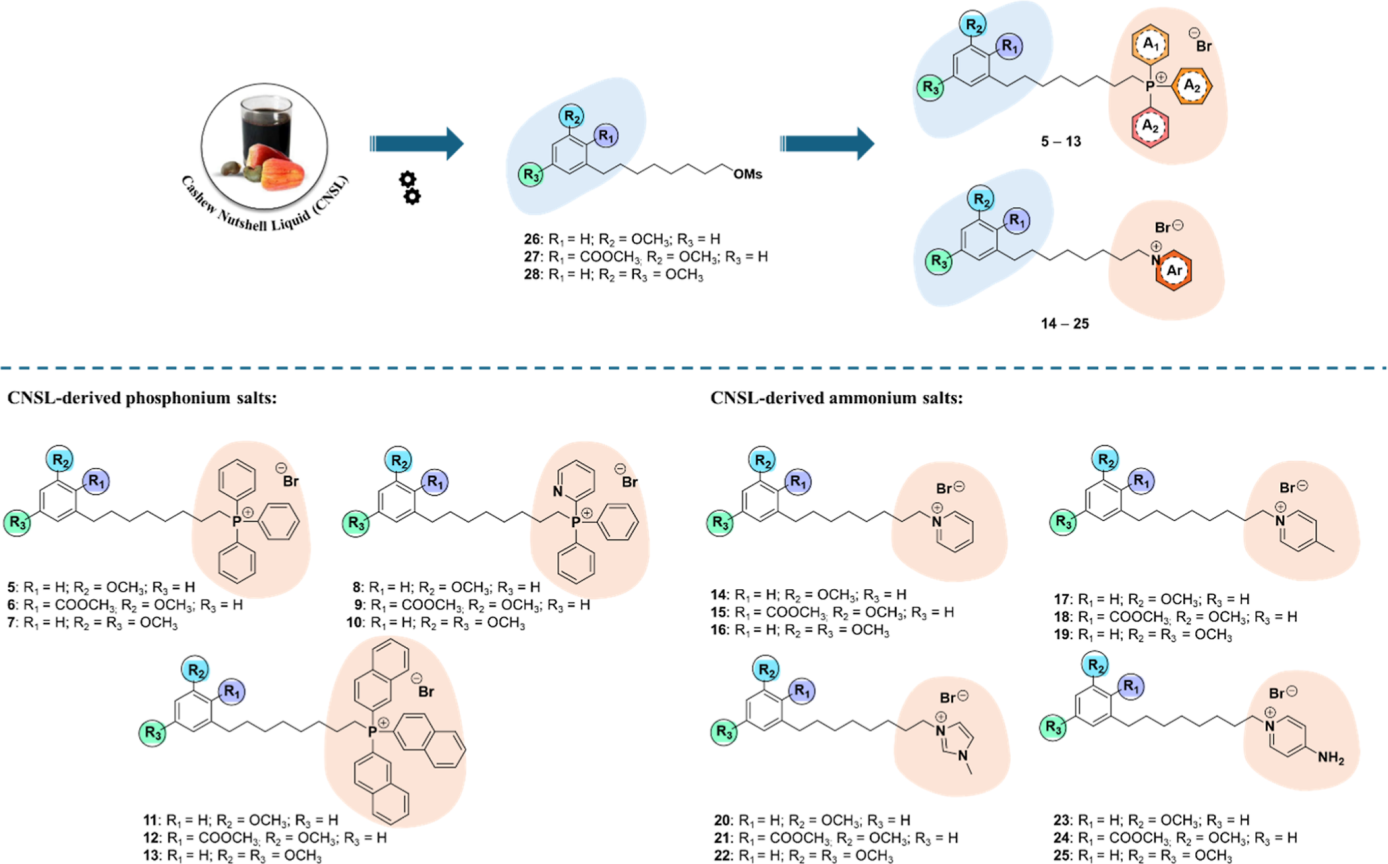
Design and chemical structures of CNSL-derived phosphonium (**5**–**13**) and ammonium (**14**–**25**) salts, starting from mesylate precursors **26**–**28**.

Particularly, parasite mitochondria are attractive drug targets due to their structural and functional differences from host mitochondria, allowing for selective disruption of parasite bioenergetics with minimal host toxicity.^28^

To incorporate the lipophilic cations at the end of the aliphatic side chain of **1–3**, we targeted CNSL intermediates with a mesylate group (**26–28**),^29^ which could be readily substituted with phosphonium (triphenylphosphonium – TPP^+^, diphenyl-2-pyridylphosphonium, tris­(naphthalen-1-yl)­phosphonium in **5**-**13**) and ammonium (pyridinium, methylpyridinium, 1-methylimidazolinium, 4-aminopyridinium in **14–25**) head groups (Figure 2). Designed compounds **5–25**, featuring a unique cationic structure, may selectively accumulate in the mitochondria of parasites, where they can induce the production of reactive oxygen species (ROS) and/or inhibit the mitochondrial F_o_F_1_ ATPase,^30^ ultimately leading to parasite cell death.^27^ To note, several series of TPP^+^ salts were developed by Dardonville and collaborators as powerful and selective agents against *Trypanosoma* and *Leishmania* spp.^30–34^

We further hypothesized that the cationic amphiphilic nature of compounds **5**–**25** could provide an additional antiparasitic mechanism through membrane-disruptive activity. Positively charged molecules can interact electrostatically with the anionic headgroups of membrane phospholipids, potentially compromising membrane integrity. Notably, phenolic and resorcinolic lipids exhibit strong amphiphilic properties and have shown high affinity for lipid bilayers.^35^ Importantly, we have reported that CNSL-derived miltefosine (MIL) analogues increase membrane protein dynamics and cause disruption at the lipid–protein interface of parasite membranes, with selectivity against the parasites and minimal hemolytic potential.^36^

### Chemistry

The synthetic route was developed considering green chemistry principles (i.e., less hazardous or solvent-free reactions, reduced reaction time, avoidance of chromatographic purification) as much as possible.

Target phosphonium (**5**–**13**) and ammonium (**14**–**25**) salts were synthesized following the convergent approach depicted in Scheme 1, taking advantage of previously synthesized CNSL-derived C8-mesylates (**26–28**) as starting reagents^29^ (Scheme 1).

**Scheme 1:**
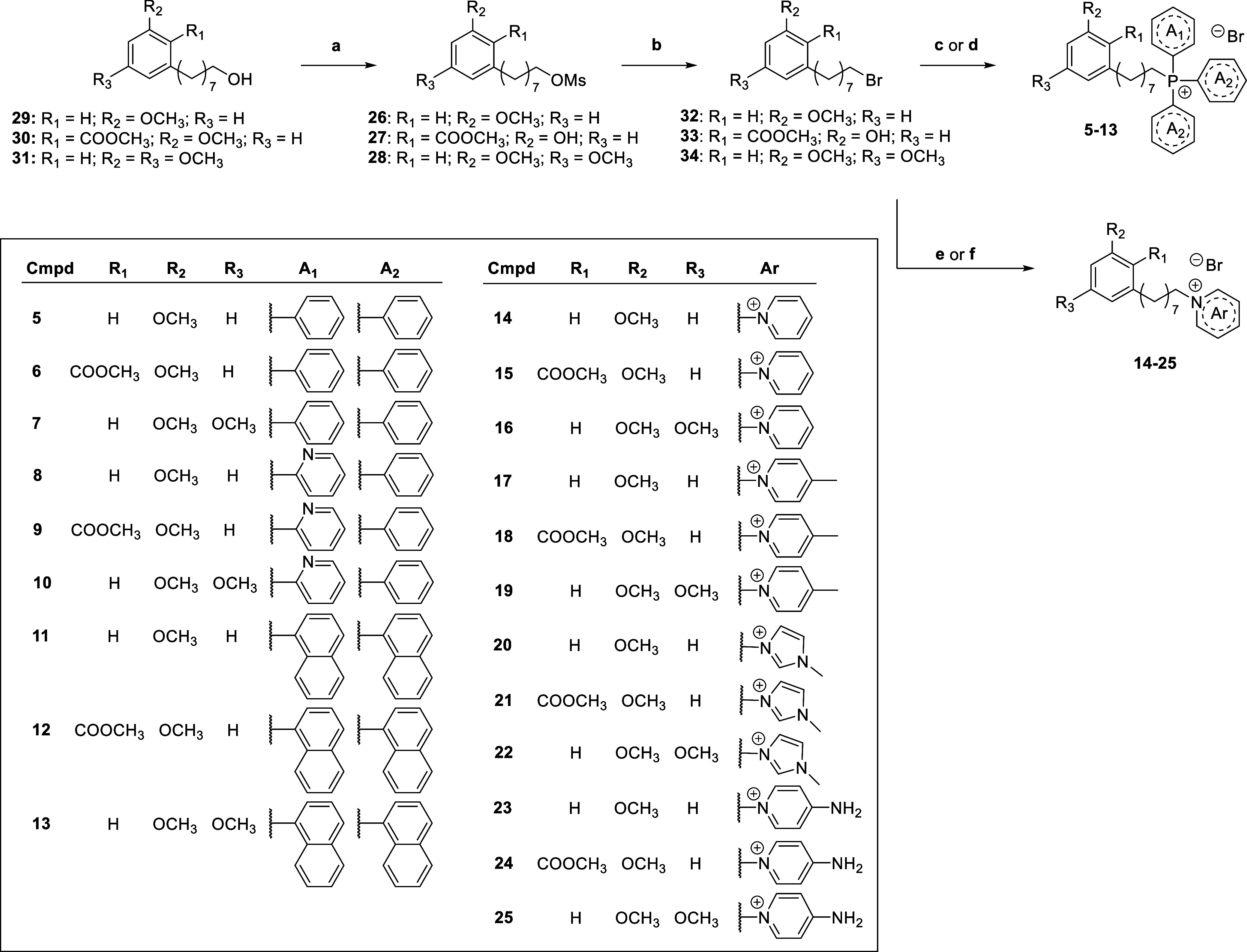
Synthesis of Final Compound **5–25** ^a^

a Reagents and conditions: (a) MsCl, Et_3_N, DMAP cat., 2-MeTHF, 0 °C → rt, 3 h (66–85%); (b) lithium bromide, acetone, reflux, 1 h (71–95%); (c) PPh_3_ (for **5**–**7**) or diphenyl-2-pyridylphosphine (for **8**–**10**), neat, N_2_, 80 °C, 2 h (45–87%); (d) tris­(naphthalen-1-yl)­phosphine (for **11**–**13**), DMF, 110 °C, 40 h (2–6%); (e) pyr (for **14**–**16**), 4-methylpyridine (for **17**–**19**) or 1-methylimidazole (for **20**–**22**), neat, N_2_, 80 °C, 2 h (22–94%); (f) 4-aminopyridine (for **23**–**25**), acetonitrile, N_2_, 80 °C, 4 h (22–77%).

As reported,^29^ unsaturated components from CNSL were extracted and methylated using methyl iodide and then subjected to ozonolysis and sodium borohydride reduction to produce C8 alcohol derivatives **29–31** (Scheme S1). In the following mesylation reaction, dichloromethane was replaced by biobased 2-methyltetrahydrofuran (2-MeTHF), which provides a sustainable alternative to previously used dichloromethane.^37^

Unfortunately, a further bromination step to more reactive bromides (better leaving groups) was necessary to transform the mesylates into the target phosphonium and ammonium salts. Thus, bromides **32–34** were synthesized from **26–28** by using inert lithium bromide and acetone as green solvent. We optimized a reported protocol^38^ by decreasing the molar excess of lithium bromide from 1:5 to 1:2 and reduction of reaction time. Finally, the phosphonium **5**–**13** and the ammonium salts **14**–**25** were synthesized by nucleophilic substitution of the appropriate bromine precursor, applying a solvent-free protocol,^38^ which was very fast compared to previous reports^33^ and led to generally good yield (45–86% for **5**–**10** and 22–94% for **14**–**22**) (Scheme 1). Due to the poor solubility of tri­(naphthalen-2-yl) phosphine in common solvents, for compound **11**–**13** the nucleophilic substitution was performed using dimethylformamide (DMF), being clearly not compatible with the drive toward more sustainable and eco-friendly protocols. Regarding ammonium salts **23**–**25**, they were obtained using acetonitrile to reduce use of DMF (yield 22–77%) (Scheme 1).

### Biology

Once synthesized, the compounds entered a screening cascade aimed at assessing their potential against several parasites responsible for major human and animal VBPDs.

### Cytotoxicity

Given the reported problem of selectivity of some phosphonium compounds,^39^ we opted to first test our series for mammalian cytotoxicity to verify their suitability for further antiparasitic drug development. Thus, a preliminary evaluation of viability profiles in human foreskin fibroblast (HFF) was performed to progress only those molecules devoid of human cell toxicity to the antiparasitic activity assays.

The data collected in Table 1 shows that cytotoxicity against human cells was quite low, with CC_50_ values ranging from 10 μM to >200 μM (highest concentration tested) for compounds **5**–**25** (Table 1). This finding allowed us to rule out nonspecific mechanisms of action potentially associated with the amphiphilic character of these compounds or off-target activity at human mitochondria at micromolar concentrations.

**Table 1:** Evaluation of the Test Compounds’ Activity (EC_50_, Mean ± SEM, nM) on Trypomastigotes of Wild-Type and Multidrug-Resistant Strains of *T. b. brucei*, and Toxicity Profile (CC_50_ Values: Mean ± SEM, μM) on HFF Cell Lines

	*T. b. brucei*							cytotoxicity	
	EC_50_ ^a^± SEM^b^ (nM)							CC_50_ ^c^± SEM^b^ (μM)	
cmpd	WT s427^d^	B48^e^	RF^f^	SUR10^g^	RF^f^	ISMR1^h^ ^,^ ^o^	RF^f^	HFF^i^	SI^j^
**5**	0.78 ± 0.18	0.99 ± 0.12	1.26	8.01 ± 0.69	10.2	10.36 ± 1.56	2.10	19.38 ± 2.72	24,715
**6**	5.83 ± 1.23	7.36 ± 1.03	1.26	4.95 ± 0.39	0.8	9.70 ± 3.12	1.89	39.86 ± 2.16	6834
**7**	0.52 ± 0.14	1.10 ± 0.14	2.12	1.23 ± 0.12	2.4	13.41 ± 2.50	1.29	20.90 ± 1.80	40,478
**8**	1.36 ± 0.33	1.64 ± 0.26	1.20	2.88 ± 0.45	2.1	8.25 ± 1.85	1.94	21.18 ± 2.22	15,562
**9**	8.71 ± 2.12	6.11 ± 1.51	0.70	7.14 ± 0.96	0.8	8.75 ± 2.75	1.16	39.53 ± 5.37	4540
**10**	1.00 ± 0.17	1.96 ± 0.38	1.97	7.46 ± 0.78	7.5	11.82 ± 0.81	1.26	19.97 ± 2.63	20,010
**11**	4.10 ± 0.32	3.08 ± 0.62	0.75	5.34 ± 0.55	1.3	5.25 ± 1.65	1.29	10.02 ± 1.18	2444
**12**	6.43 ± 0.87	4.94 ± 0.49	0.77	8.18 ± 1.04	1.3	8.30 ± 2.60	1.51	14.32 ± 0.09	2227
**13**	6.40 ± 1.13	6.03 ± 1.36	0.94	9.88 ± 0.73	1.5	6.59 ± 1.99	1.50	9.91 ± 1.39	1548
**14**	5340 ± 330	5220 ± 380	0.98	n.d.	n.d.	n.d.	n.d.	73.73 ± 19.27	24
**15**	3930 ± 490	6090 ± 1150	1.55	n.d.	n.d.	n.d.	n.d.	>200	>51
**16**	2260 ± 190	1940 ± 90	0.86	n.d.	n.d.	n.d.	n.d.	90.10	40
**17**	1590 ± 60	1450 ± 80	0.92	n.d.	n.d.	n.d.	n.d.	193.5 ± 6.50	122
**18**	2390 ± 150	2990 ± 570	1.25	n.d.	n.d.	n.d.	n.d.	>200	>84
**19**	1340 ± 40	1190 ± 70	0.89	n.d.	n.d.	n.d.	n.d.	113.40	85
**20**	2530 ± 60	2850 ± 700	1.12	n.d.	n.d.	n.d.	n.d.	196.0 ± 4.0	77
**21**	2640 ± 250	3070 ± 430	1.16	n.d.	n.d.	n.d.	n.d.	>200	>76
**22**	2110 ± 210	2290 ± 270	1.08	n.d.	n.d.	n.d.	n.d.	n.d.	n.d.
**23**	800 ± 270	940 ± 100	1.18	n.d.	n.d.	n.d.	n.d.	83.19 ± 6.12	104
**24**	940 ± 160	1200 ± 80	1.29	n.d.	n.d.	n.d.	n.d.	149.5 ± 30.5	160
**25**	4370 ± 130	2730 ± 630	0.63	n.d.	n.d.	n.d.	n.d.	73.10 ± 6.55	17
**29**	84,250 ± 3800	n.d.	n.d.	n.d.	n.d.	n.d.	n.d.	n.d.	n.d.
**30**	53,350 ± 3470	n.d.	n.d.	n.d.	n.d.	n.d.	n.d.	n.d.	n.d.
**31**	59,670 ± 1600	n.d.	n.d.	n.d.	n.d.	n.d.	n.d.	n.d.	n.d.
**PMD** ^k^	4.71 ± 1.30	169 ± 19	36	5.06 ± 0.90	1.1	156.5 ± 20.2	41.24	n.d.	n.d.
**SUR** ^l^	78.6 ± 2.0	n.d.	n.d.	969 ± 272	12.3	n.d.	n.d.	n.d.	n.d.
**ISM** ^m^	n.d.	n.d.	n.d.	n.d.	n.d.	2589 ± 146	6.04	n.d.	n.d.
**PAO** ^n^	n.d.	n.d.	n.d.	n.d.	n.d.	n.d.	n.d.	1.74 ± 0.49	n.d.

a EC_50_: 50% effective concentration.

b SEM: standard error of the mean.

c CC_50_: 50% cytotoxic concentration.

d Trypomastigotes of *T. b. brucei* s427.

e B48 strain is a mutant derived from the TbAT1-KO strain with a nonfunctional high affinity pentamidine transporter (HAPT). This strain is resistant to diminazene, pentamidine, and melaminophenyl arsenicals.

f Resistance factor relative to WT: EC_50(test strain)_/EC_50(control strain)_.

g SUR10: BSF of *T. b. brucei* rendered resistant to suramin by *in vitro* exposure.

h ISMR1: clonal, *Tb*427WT-derived dys-kinetoplastid cell line, adapted to *in vitro* growth in 1 μM isometamidium (ISM) by stepwise increases in the medium concentration of the drug.

i HFF: Human Foreskin Fibroblast cell line.

j SI: selectivity index = CC_50_/EC_50_ (*T. brucei* WT).

k PMD: pentamidine.

l SUR: suramin.

m ISM: isometamidium.

n PAO: phenylarsine oxide.

o The EC_50_ values for the strain ISMR1 were obtained using a lower cell density and longer incubation time than for all other strains, as described,^40^ in parallel with a further set of 427WT determinations using the same protocol; the RF values given are relative to that second set of values.

Owing to the lack of major cytotoxicity, all the compounds were screened against a broad panel of parasites, including animal *Trypanosoma* species (*T. b. brucei*, *T. b. equiperdum*, *T. b. evansi*, *T. vivax*, *T. congolense*), two species of *Leishmania* (*L. mexicana* and *L. amazonensis*, both causing CL) and *T. cruzi*, responsible for CD.

### *In Vitro* Activity against *T. b. brucei*

The limited chemotherapeutic and chemoprophylaxis options for managing AAT (also known in much of Africa as Nagana) caused by *T. b. brucei*, based on diminazene aceturate (DA) and isometamidium chloride (ISMc), pose a significant challenge to the World Organisation for Animal Health (WOAH)’s control targets,^41^ especially because of widespread resistance.^5^ The preferred target product profiles (TPP) describe a single treatment, active against the entire range of species,^42^ and against parasite strains resistant to the existing drugs.^43^

For these reasons, the trypanocidal activity of compounds **5**–**25** was assessed against bloodstream trypomastigotes of both the standard drug-sensitive strain Lister 427 wild-type (427WT) and several (multi)­drug-resistant strains of *T. b. brucei* (Table 1). The results were compared with those of reference drugs used in veterinary medicine (isometadinium (ISM)) and human treatments (pentamidine (PMD) and suramin (SUR)). Clone B48 is highly resistant to DA, PMD, melarsoprol and cymelarsan; SUR10 is an *in vitro*-adapted clone ∼10-fold resistant to suramin;^34^ ISMR1 is highly resistant to ISM, ethidium bromide (homidium), DA and PMD.^40^ Additionally, the activity of the starting alcohols **29**–**31**, which lack the lipophilic cation, was assessed to validate the design rationale.

As evident from the data in Table 1, the inclusion of the mitochondrion-targeting lipophilic cation head was essential to obtain potent activity, as shown for salicylhydroxamic acid (SHAM)-based lipophilic cation inhibitors.^32^ Indeed, all phosphonium and ammonium salts **5**–**25** (EC_50_ = 0.52–5340 nM) were far more active than the alcohols **29**–**31** (53.35–84.25 μM). The trypanocidal activity of phosphonium derivatives **5**–**13** was remarkable, with potency ranging from subnanomolar to low nanomolar concentrations (0.52–8.71 nM). The phosphonium conjugates were between 613- and 1539-fold more potent than their parent ammonium salts (800–5340 nM). This result is consistent with earlier studies on lipophilic cation-linked TAO inhibitors^30,31^ as well as diphenyl cationic trypanocides,^44^ and probably reflects the higher lipophilicity (see Table S4 for details) and charge dispersion around the phosphorus atom in the TPP^+^ cation with respect to ammonium ones, which is optimal for membrane permeation and accumulation in the mitochondrion. Remarkably, cardanol **5** and cardol **7** derivatives showed subnanomolar activity, with EC_50_ values an order of magnitude below that of the HAT benchmark drug PMD.

As current drugs are becoming ineffective due to drug resistance, cross-resistance between existing drugs and new ones is of critical importance.^5^

For this reason, **5**–**25** were tested against the multidrug-resistant strain B48, which is highly resistant to the first-line diamidine and melaminophenyl arsenical trypanocides. This strain displayed 36-fold resistance to PMD (Table 1), but there was no significant difference in sensitivity to the current test compounds, with resistance factors (RF) consistently close to 1 except for **7** and **10** (∼2-fold). The low differences in susceptibility between the WT and multidrug-resistant B48 cell lines means that cross-resistance with existing first-line HAT and AAT drugs, including PMD, DA, melarsomine, and melarsoprol, is unlikely with these compounds. This is intriguing, considering the fact that diamidines also have mitochondrial targets.^45,46^ However, it could be attributed to the fact that both diamidine and melaminophenyl arsenical resistance in *T. brucei* is associated with the loss of specific cell surface transporters, whereas the TPP^+^-derivatives are likely to passively diffuse across biological membranes, indicating that they do not utilize the known drug transporters P2/*Tb*AT1 (aminopurine transporter) and HAPT (high-affinity PMD transporter).^47^

Finally, the activity of the most active compounds (**5**–**13**) was assessed against *T. b. brucei* suramin-resistant strain SUR10 and *T. b. brucei* ISMR1, a clonal, *Tb*427WT-derived dyskinetoplastid cell line, adapted to *in vitro* growth in 1 μM of ISM by stepwise increases in the medium concentration of the drug.^40^ **5**–**13** showed activity in the nanomolar range, with EC_50_ values ranging from 1.23–9.88 nM against *T. b. brucei* SUR10, and slightly higher against ISMR1 (5.25–13.41 nM), resulting in good to excellent activity compared to reference drug PMD. In this case, cross-resistance with ISM is at most 2.1-fold (**5**), which is remarkable as this strain is resistant to all the mitochondrion-targeting phenanthridine and diamidine drugs, while only compound **5** and **10** displayed real cross resistance with SUR, by 10.2 and 7.5-fold respectively (*P* < 0.001 and *P* < 0.01, respectively, by unpaired *t*test). Suramin resistance is a particular concern for the treatment of *T. b. evansi* infections in camels.^48^

As previously discussed, the phosphonium series demonstrated cytotoxicity against the human HFF cell line in the micromolar range (CC_50_ values between 10.02 and 39.86 μM), resulting in remarkably high selectivity indices (all SI > 1500 and up to ∼40,000). In contrast, ammonium salts derivatives, being active in the micromolar range, exhibited much lower SI (17–160).

### *In Vitro* Activity against *T. congolense*, *T. b. equiperdum*, *T. b. evansi*

Besides *T. brucei*, other *Trypanosoma* spp., such as *T. congolense*, *T. b. evansi*, *T. b. equiperdum* and *T. vivax* are responsible of AAT.^41,43^ *T. b. equiperdum* is an important veterinary trypanosome endemic in Africa and Asia, also found in the Middle East, South-East Europe and South America and responsible for a disease called dourine in equids. It causes severe symptoms like genital lesions, fever, and neurological issues. It spreads through breeding and is therefore not limited to the habitat or distribution of any vector. Slaughter and strict biosecurity are key, as no effective treatments or vaccines exist.^49^ *T. b. evansi* causes surra, one of the most important diseases of camels. Camel raising in Africa and buffalo production in Asia are severely affected by surra, with suramin being relatively widely used to treat these infections but treatment options include DA, ISM, quinapyramine and cymelarsan depending on animal species, disease severity and drug availability.^50^ Worryingly, an increasing number of reports of resistance to each of those chemicals, particularly DA and ISM, indicate their future utility to be in jeopardy.^5^ Thus, the most active phosphonium salts derivatives **5**–**13** were evaluated also against *T. b. equiperdum*, *T. b. evansi* and to a DA-resistant (4C2) and sensitive control clone (IL3000) of *T. congolense*, always in comparison with the reference drugs (Table 2).

**Table 2:** Evaluation of the Test Compounds Activity (EC_50_, Mean ± SEM, nM) on Trypomastigotes of *T. b. equiperdum*, *T. b. evansi*, *T. congolense* WT and *T. congolense* Multidrug Resistant Strain 4C2

	*T. b. equiperdum* ^a^		*T. b. evansi* ^b^		*T. congolense* WT^c^		*T. congolense* 4C2 BSF^d^	
cmpd	EC_50_ ^e^± SEM^f^ (nM)	RF^g^	EC_50_ ^e^± SEM^f^ (nM)	RF^g^	EC_50_ ^e^± SEM^f^ (nM)	RF^g^	EC_50_ ^e^± SEM^f^ (nM)	RF^g^
**5**	5.17 ± 0.42	6.59	1.60 ± 0.44	2.04	89.88 ± 2.51	115	84.20 ± 6.63	0.94
**6**	7.21 ± 0.93	1.24	3.66 ± 0.50	0.63	603.8 ± 24.4	104	860.8 ± 37.6	1.43
**7**	0.50 ± 0.01	0.97	1.67 ± 0.36	3.24	128.3 ± 4.6	248	156.0 ± 13.3	1.22
**8**	3.04 ± 0.45	2.23	2.44 ± 0.25	1.80	68.07 ± 4.75	50	68.13 ± 5.45	1.00
**9**	32.36 ± 1.54	3.72	18.93 ± 3.87	2.17	519.7 ± 23.3	60	739.5 ± 107.4	1.42
**10**	1.75 ± 0.22	1.75	4.86 ± 1.77	4.87	70.88 ± 3.12	71	78.77 ± 6.50	1.11
**11**	1.43 ± 0.20	0.35	3.46 ± 0.36	0.84	9.89 ± 1.52	2.4	15.55 ± 2.47	1.57
**12**	5.24 ± 0.07	0.82	6.00 ± 0.97	0.94	31.92 ± 3.10	5.0	39.10 ± 2.61	1.22
**13**	7.42 ± 0.45	1.16	7.21 ± 1.26	1.13	20.41 ± 1.72	3.2	18.42 ± 0.81	0.90
**PMD** ^h^	3210 ± 370	0.68	22.51 ± 2.41	4.78	96.03 ± 3.02	20.4	577.4 ± 66.2	6.01
**DA** ^i^	n.d.	n.d.	n.d.	n.d.	178.7 ± 6.2	n.d.	818.0 ± 21.7	4.58

a Trypomastigotes (BSF) of *T. b. equiperdum* BoTat 1.1.

b Trypomastigotes (BSF) of *T. b. evansi* AnTat 3.3.

c Trypomastigotes (BSF) of *T. congolense* IL3000.

d Trypomastigotes (BSF) of *T. congolense* 4C2. The strain is resistant to diminazene aceturate and pentamidine.

e EC_50_: 50% effective concentration.

f SEM: standard error of the mean.

g Resistance factor relative to WT.

h PMD: pentamidine.

i DA: diminazene aceturate.

Phosphonium salt derivatives **5**–**13** demonstrated exceptional *in vitro* activity against *T. b. equiperdum*, with EC_50_ values ranging from 0.50 to 32.36 nM (Table 2), a level of potency that is particularly remarkable given the absence of reported effective treatments for infections with this parasite. This demonstrates the significant potential of these compounds as a novel therapeutic avenue for managing dourine in equids. Furthermore, they exhibited remarkable activity against *T. b. evansi* and *T. congolense* (Table 2). Particularly, compounds **5**–**13** showed similar nanomolar activity against *T. b. evansi* (within the 1.60–18.93 nM range), along with a similar safety profile, reinforcing their broad-spectrum trypanocidal potential. While activity against *T. congolense* was substantially lower compared to other strains, most of them still outperformed current treatments, such as PMD and the standard veterinary drug DA, in terms of efficacy against both the wild-type and multidrug-resistant (4C2) strains. Importantly, there was no observed cross-resistance with the diamidine drugs, as indicated by resistance factors consistently close to 1. This is particularly important because both *T. b. brucei* clone ISMR1 and *T. congolense* clone 4C2 display significantly reduced mitochondrial membrane potential as part of their respective resistance adaptations^40,51^ and the absence of cross-resistance shows, surprisingly, that such an adaptation will not lead to significant levels of resistance to these mitochondrially targeted cardols and cardanols. This important conclusion further highlights the promising therapeutic utility of these phosphonium salt derivatives.

### *In Vitro* Activity against *T. cruzi*

Next, the antiparasitic activity of all derivatives against *T. cruzi* (the causative agent of CD) was evaluated against intracellular amastigotes within L929 fibroblast cells and against bloodstream forms, using benznidazole (Bz) as a reference drug.^52^

To further assess compound toxicity, an initial dose–response study was conducted on uninfected L929 mouse fibroblasts. CC_50_ values after 96 h of exposure ranged from 11.0 to 75.8 μM (Table 3). Selected phosphonium salts and ammonium derivatives—specifically cardanol-based compounds **14**, **17**, and **20**, along with 4-aminopyridinium conjugates **23**–**25**—showed significant cytotoxicity (CC_50_ = 11.0–18.1 μM). In contrast, alcohols **29**–**31** were significantly less toxic (CC_50_ = 159–200 μM), consistent with the known cytotoxicity levels associated with lipophilic cations.^39^

**Table 3:** Evaluation of the Test Compounds’ Activity against Intracellular and Bloodstream Trypomastigote Forms (BTs) of *Trypanosoma cruzi* (EC_50_ Values, Mean ± SD), and Toxicity Profile (LC_50_ Values, Mean ± SD, μM) on L929 Cell Lines after 96 h of Drug Exposure

	intracellular *T. cruzi* ^a^		BTs *T. cruzi* ^b^	L929^c^	
cmpd	EC_50_ ^d^± SD^e^ (μM)	EC_90_ ^f^± SD^e^ (μM)	EC_50_ ^d^± SD^e^ (μM)	LC_50_ ^g^± SD^e^ (μM)	SI^h^
**5**	0.32 ± 0.07	0.64 ± 0.18	0.12 ± 0.01	12.50 ± 0.00	39
**6**	0.64 ± 0.11	1.47 ± 0.23	0.19 ± 0.02	13.50 ± 1.47	21
**7**	0.44 ± 0.12	0.83 ± 0.27	0.17 ± 0.07	12.50 ± 0.00	28
**8**	0.60 ± 0.07	1.36 ± 0.10	0.12 ± 0.10	13.50 ± 1.47	23
**9**	1.27 ± 0.19	2.48 ± 0.79	0.09 ± 0.07	13.50 ± 1.47	11
**10**	0.56 ± 0.20	1.14 ± 0.48	0.19 ± 0.13	12.50 ± 0.00	22
**11**	0.30 ± 0.04	0.56 ± 0.12	0.15 ± 0.17	12.50 ± 0.00	42
**12**	n.d.	n.d.	0.20 ± 0.14	12.50 ± 0.00	n.d.
**13**	0.33 ± 0.06	0.64 ± 0.19	0.27 ± 0.12	12.50 ± 0.00	38
**14**	n.d.	n.d.	5.42 ± 0.99	14.60 ± 0.00	n.d.
**15**	n.d.	n.d.	n.d.	83.10 ± 3.23	n.d.
**16**	n.d.	n.d.	4.04 ± 0.59	68.50 ± 11.44	n.d.
**17**	3.35 ± 1.33	8.85 ± 0.35	1.15 ± 0.01	14.60 ± 0.00	5
**18**	n.d.	n.d.	4.45 ± 0.35	39.00 ± 2.07	n.d.
**19**	n.d.	n.d.	1.78 ± 0.22	15.30 ± 1.00	n.d.
**20**	7.14 ± 0.98	>10	5.03 ± 2.60	15.60 ± 1.50	>2
**21**	n.d.	n.d.	9.74 ± 2.47	75.80 ± 1.13	n.d.
**22**	n.d.	n.d.	4.65 ± 0.39	43.20 ± 0.50	n.d.
**23**	1.46 ± 0.36	3.32 ± 0.95	0.79 ± 0.05	14.90 ± 0.00	10
**24**	n.d.	n.d.	1.1 ± 0.7	18.10 ± 4.51	n.d.
**25**	4.43 ± 0.00	8.18 ± 1.57	1.13 ± 0.31	11.00 ± 4.24	3
**29**	n.d.	n.d.	n.d.	>200	n.d.
**30**	n.d.	n.d.	n.d.	159.40 ± 7.40	n.d.
**31**	n.d.	n.d.	n.d.	>200	n.d.
**Bz** ^i^	2.32 ± 0.61	7.63 ± 1.76	8.55 ± 2.81	>200	>76

a Intracellular amastigotes form of *T. cruzi* (Tulahuen strain).

b Bloodstream trypomastigote forms of *T. cruzi* (Y strain, DTU II).

c L929 mice fibroblast cell lines.

d EC_50_: 50% effective concentration.

e SD: standard deviation.

f EC_90_: 90% effective concentration.

g LC_50_: 50% lethal concentration.

h SI: selectivity index.

i Bz: beznidazole.

Following the toxicity profiling, a preliminary screening of compounds **5**–**25** against intracellular *T. cruzi* amastigotes was conducted at a fixed concentration of 10 μM (comparable to the Bz EC_90_). Twelve of the 21 compounds reduced parasitism by ≥ 50% and were selected for further evaluation through full dose–response assays (Table 3). EC_50_, EC_90_ values, and selectivity indices (SI) were determined after 96 h of drug exposure.^53^

Notably, phosphonium salts (compounds **5**–**13**) demonstrated up to an order of magnitude higher potency than Bz against intracellular amastigotes, with EC_50_ values ranging from 0.32 to 1.27 μM compared to 2.32 μM for Bz. Compounds **5**, **7**, **11**, and **13** were the most active, showing robust anti-*T. cruzi* activity in infected L929 cells. Although some cytotoxicity was detected, the selectivity indices (SI = 28–42) remained acceptable.

Against the bloodstream trypomastigotes, a high activity was also found. Except for the compound **21**, all compounds were as active or even more potent than Bz. Compound **9** was one of the most potent reaching EC_50_ value = 0.09 μM after 24 h of drug exposure, being about 95-fold more active than the reference drug (Table 3). Notably its bloodstream trypomastigote activity was 14-fold higher than that against the intracellular form (EC_50_ value = 1.27 μM). Bloodstream trypomastigotes are a nondividing form and we can thus conclude that the test compounds do not act on any aspect of cell division as part of their antiparasitic properties. Moreover, it seems possible that the compounds have greater utility against extracellular than intracellular parasites; all African trypanosome species lack an intracellular stage in their life cycle.

### *Ex Vivo* Activity against *T. vivax* and *T. congolense*

Building on the promising *in vitro* results demonstrating the nanomolar potency of our phosphonium salt derivatives against various *Trypanosoma* species, including *T. congolense*, we advanced to *ex vivo* studies using *T. vivax* ILRAD700 and *T. congolense* MSORO M7H strains, both the original and ISM-resistant (ISM-R) variant (Table 4).

**Table 4:** *Ex Vivo* Evaluation of the Test Compounds’ Activity against BS Trypomastigote Forms of *T. vivax* ILRAD700 and *T. congolense* Original and ISM-R (MSORO M7H) Strains (EC_50_ Values, Mean ± SEM)

	*T. vivax* (ILRAD700)^a^	*T. congolense*(MSORO M7H)^b^	*T. congolense* ISM-R (MSORO M7H)^c^
cmpd	EC_50_ ^d^± SEM^e^ (nM)	EC_50_ ^d^± SEM^e^ (nM)	
**5**	32.25 ± 7.27	165.29 ± 47.50	227.47 ± 10.64
**6**	16.05 ± 8.27	186.10 ± 43.75	304.68 ± 26.52
**7**	33.70 ± 20.11	126.36 ± 91.28	247.95 ± 33.44
**8**	29.49 ± 0.04	121.10 ± 30.89	206.22 ± 45.25
**9**	19.67 ± 8.85	264.28 ± 99.35	373.65 ± 42.05
**10**	24.08 ± 11.17	189.84 ± 35.48	229.49 ± 38.70
**11**	240.80 ± 47.4	215.23 ± 63.69	282.87 ± 65.86
**12**	227.50 ± 64.2	191.57 ± 58.44	209.94 ± 24.77
**13**	98.59 ± 9.51	305.53 ± 13.87	340.81 ± 62.76
**29**	18175 ± 3955	64000 ± 0	64000 ± 0
**30**	21450 ± 2210	64000 ± 0	64000 ± 0
**31**	21740 ± 70	64000 ± 0	59393 ± 4607
**DA** ^f^	498.60 ± 90.40	754.04 ± 23.19	687.60 ± 78.30

a Bloodstream trypomastigotes forms of *T. vivax* ILRAD700.

b Bloodstream trypomastigotes forms of *T. congolense* (MSORO M7H).

c Bloodstream trypomastigotes forms of *T. congolense* ISM-R (MSORO M7H).

d EC_50_: 50% effective concentration.

e SEM: standard error of the mean.

f DA: diminazene aceturate.

Guided by prior efficacy data, we selected the most active compounds (**5**–**13**) for *ex vivo* evaluation. These phosphonium salt derivatives demonstrated outstanding activity, with EC_50_ values in the nanomolar range. Against *T. vivax*, EC_50_ values ranged from 16.05 to 240.80 nM, while for *T. congolense*, slightly higher EC_50_ values were observed (192.90–356.05 nM) (Table 4), consistent with previous *in vitro* findings against *T. b. brucei* strain IL3000 (Table 2). The strong activity and absence of biologically significant differences between the original and ISM-R strains of *T. congolense* MSORO M7H suggests that the tested compounds do not share the same resistance mechanisms as ISM. All tested compounds outperformed the reference drug DA, against both parasite species, underscoring their potential as next generation antitrypanosomal agents. Notably, the anacardic acid derivatives **6**, bearing a TPP^+^ moiety, and **9**, featuring a diphenyl-2-pyridylphosphonium headgroup, exhibited the most potent activity among the series (16.05 and 19.67 nM, respectively) against *T. vivax*. In contrast, against *T. congolense* cardanol (**5** and **8**) and cardol (**7** and **10**) derivatives carrying both TPP^+^ and diphenyl-2-pyridylphosphonium seemed to be the most efficacious (Table 4).

### *In Vitro* Activity against *L. mexicana*

*Leishmania* parasites adopt two major morphological forms during their life-cycle stages: promastigotes in the insect vector and amastigotes in the mammalian host. First, compounds **5**–**25** and **29–32** were evaluated against promastigotes of the *L. mexicana* Cas9 strain,^54^ causing CL, as previously described.^55^ Interestingly, several of the compounds were more active against *L. mexicana* promastigotes Cas9 strain than reference drug amphotericin B (AmB) and many were more active than PMD, displaying EC_50_ values spanning from 0.05 to 16.14 μM (Table 5).

**Table 5:** Tested Compounds Activity Upon *in vitro* *L. mexicana* Promastigote (EC_50_ Values – μM – Mean ± SEM) and *ex vivo* and Intracellular Amastigotes of *L. amazonensis*, Toxicity on PMM (EC_50_ and LC_50_ Values - μM - Mean ± SD) and SI

		*L. amazonensis* ^b^				
	*L. mexicana*Cas9 WT^a^	*ex vivo*		intracellular in PMM	PMM^c^	SI^d^
cmpd	EC_50_ ^e^± SEM^f^ (μM)	% reduced parasite viability (30 μM)	EC_50_ ^e^± SD^g^ (μM)	EC_50_^e^ ± SD^g^ (μM) (SI)	LC_50_ ^h^± SD^f^ (μM)	
**5**	0.17 ± 0.08	94	0.37 ± 0.09	0.01 ± 0	<6.25	n.d.
**6**	0.20 ± 0.05	94	0.29 ± 0.08	0.03 ± 0	<6.25	n.d.
**7**	0.19 ± 0.02	96	0.34 ± 0.18	0.05 ± 0	<6.25	n.d.
**8**	0.13 ± 0.03	94	0.30 ± 0.14	0.08 ± 0.07	<6.25	n.d.
**9**	0.14 ± 0.03	94	0.40 ± 0.14	0.11 ± 0.05	<6.25	n.d.
**10**	0.15 ± 0.02	100	0.37 ± 0.17	0.04 ± 0.01	<6.25	n.d.
**11**	0.05 ± 0.01	99	0.35 ± 0.06	0.03 ± 0	<6.25	n.d.
**12**	0.16 ± 0.03	100	0.42 ± 0.04	0.05 ± 0.03	<6.25	n.d.
**13**	0.14 ± 0.03	96	0.26 ± 0.09	0.05 ± 0.02	<6.25	n.d.
**14**	4.57 ± 0.59	63	5.44 ± 0.52	n.d.	89.18 ± 4.78	16
**15**	16.14 ± 3.59	47	n.d.	n.d.	n.d.	n.d.
**16**	7.53 ± 1.88	76	7.83 ± 0.89	n.d.	137.01 ± 64.47	17
**17**	0.43 ± 0.08	93	2.65 ± 0.55	0.65 ± 0.58 (57)	36.74 ± 7.07	14
**18**	5.90 ± 0.15	93	9.05 ± 0.35	n.d.	68.93 ± 10.67	8
**19**	2.36 ± 0.49	98	4.24 ± 0.04	n.d.	37.99 ± 8.33	9
**20**	1.09 ± 0.22	74	3.77 ± 0.77	0.17 ± 0.15 (602)	102.4 ± 1.27	27
**21**	7.67 ± 1.74	69	9.70 ± 2.20	n.d.	>100	10
**22**	3.40 ± 0.51	80	4.84 ± 2.49	n.d.	157.75 ± 39.53	33
**23**	0.23 ± 0.06	98	0.10 ± 0.04	0.05 ± 0	<6.25	n.d.
**24**	1.42 ± 0.18	87	2.55 ± 0.25	n.d.	33.58 ± 1.09	13
**25**	2.20 ± 0.47	94	1.27 ± 0.37	0.15 ± 0.02 (84)	12.62 ± 3.06	10
**29**	173.93 ± 2.78	<20	>30	n.d.	n.d.	n.d.
**30**	>200	<20	>30	n.d.	n.d.	n.d.
**31**	186.10 ± 0.78	<20	>30	n.d.	n.d.	n.d.
**PMD** ^i^	0.76 ± 0.04	82.5	n.d.	n.d.	n.d.	n.d.
**AmB** ^j^	0.15 ± 0.01	n.d.	n.d.	n.d.	n.d.	n.d.
**MIL** ^k^	n.d.	83.5	1.38 ± 0.17	1.31 ± 0.8 (100)	131.5 ± 34.65	95

a Promastigotes of *L. mexicana* Cas9 strain.

b Intracellular (amastigotes) forms of *L. amazonensis* (MHOM/BR/77/LTB0016 strain).

c PMM: peritoneal mouse macrophages.

d SI: Selectivity index based on *ex vivo* assays.

e EC_50_: 50% effective concentration.

f SEM: standard error of the mean.

g SD: standard deviation.

h LC_50_: 50% lethal concentration.

i PMD: pentamidine.

j AmB: amphotericin B.

k MIL: miltefosine.

### *Ex Vivo* Activity against *L. amazonensis*

Next, assays were performed using *L. amazonensis* amastigotes obtained from infected mouse cutaneous lesions (Table 5).^56^ *L. amazonensis* accounts for a broader spectrum of diseases, from cutaneous lesions, to mucocutaneous or visceralization.

In the first set of analysis, a fixed dose (30 μM) was used as cutoff. 20 out of 21 phosphonium and ammonium salts reduced the parasitism ≥50% (only the anacardic acid derivative **15**, carrying pyridinium lipophilic head showed just a 47% reduction) and were moved forward to the dose–response curve assays (Table 5). As expected, compounds **29**–**31** did not reduce parasitism (<20%). As depicted in Table 5, 10 compounds were more potent than the reference drugs after 48 h of drug incubation at 32 °C *in vitro*, reaching EC_50_ values ranging from 0.10–0.42 μM.

Then, cytotoxicity of compounds **5**–**25** was assessed toward peritoneal mouse macrophages (PMM) and obtained after 48 h of compounds’ incubation at 37 °C. The cellular viability was analyzed using the Alamar Blue (resazurin sodium salt) reagent. Due to toxicity against peritoneal mouse macrophages (PMM) (<6.25 μM for the most active compounds), it was not possible to determine SI.

Finally, the phosphonium salts series **5**–**13** and selected ammonium salts derivatives were tested for their activity on intracellular amastigotes of *L. amazonensis* in infected PMM (ratio 2.5:1, parasite/host cell) after 48 h treatment (Table 5). Our findings demonstrate that all the 13 compounds tested were highly active against intracellular forms of *L. amazonensis* in PMM, exhibiting higher potency than MIL, being about 2 and 131-fold more potent than this reference drug. Compounds **5, 6, 7, 8, 10–13** and **23** displayed EC_50_ values lower than 0.1 μM (Table 5). Compounds **17**, **20** and **25** also displayed high selectivity indexes (57, 602 and 84, respectively) suggestive of hit compounds.

### *In Vitro* Drug Susceptibility Growth Tests

We also evaluated our best-performing compounds in two *in vitro* drug susceptibility growth tests with *T. brucei* that have served as the basis for the identification of recently approved fexinidazole: (1) a standard assay with end-point measurement to determine drug efficacy; (2) a wash-out assay to test for reversibility and speed of drug action.^57^ Together, these assays allow an estimate of the dynamics of drug action *in vitro* and devise appropriate treatment regimens for subsequent *in vivo* experiments. Phosphonium salts **5** and **7** were selected as (i) displaying subnanomolar activity against the parasite, (ii) possessing >10,000-fold *in vitro* selectivity with a human cell line (HFF) and (iii) representing the two main components of CNSL, being cardanols and cardols, functionally derivatized with TPP^+^ and representing the most accessible compounds from a synthetic point of view.

Studies of time-dose response (time-to-kill) of *T. brucei* following exposure to **5** and **7** are illustrated in Figure 3. To determine whether **5** and **7** inhibited growth and/or cause cell death of *T. b. brucei*, cell growth curves were performed by treating 427WT trypomastigotes with concentrations corresponding to 2× the EC_50_ and 5× the EC_50_ values, using untreated cells as a control.

**Figure 3:**
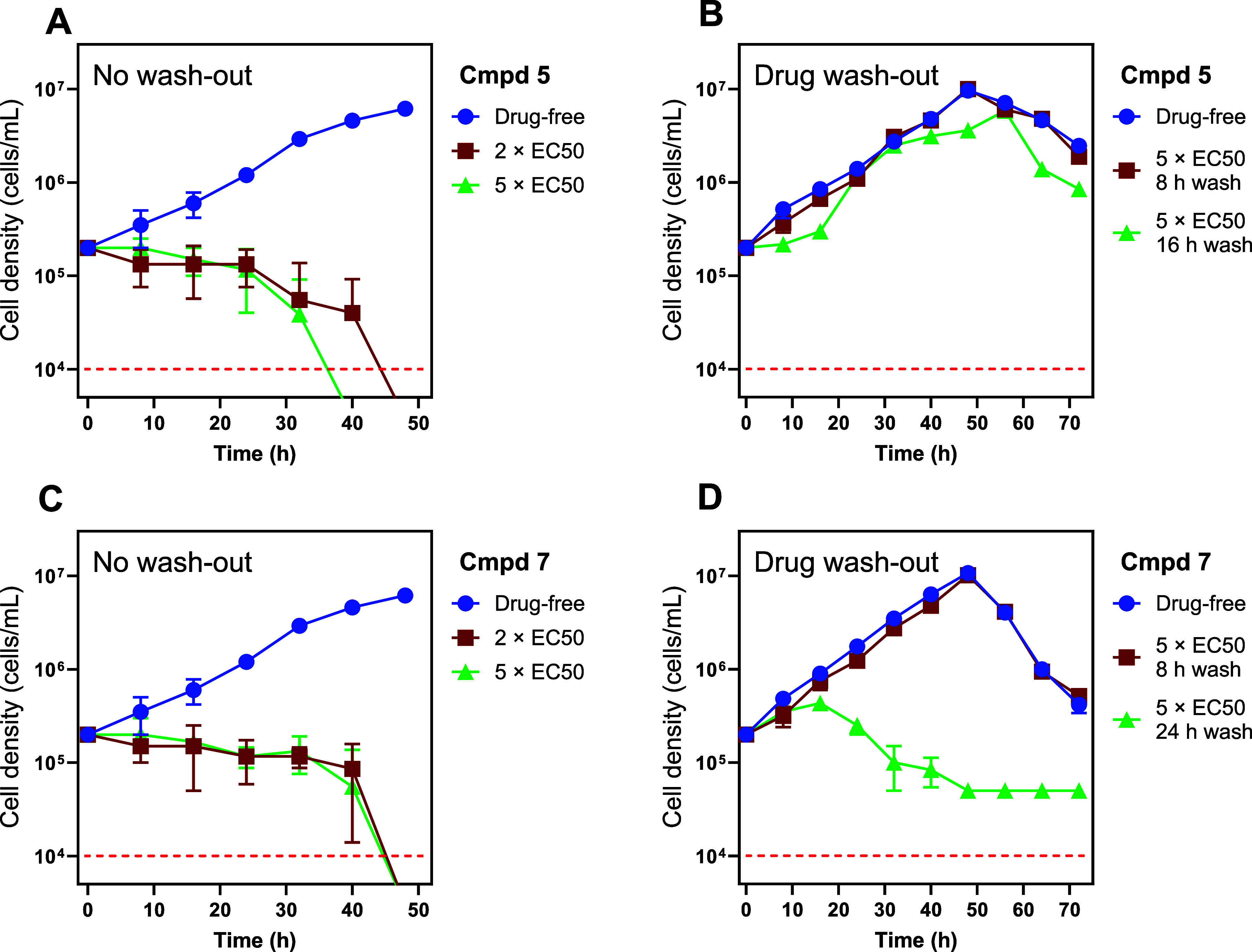
Growth curves of *T. b. brucei* s427 bloodstream forms in the presence of **5** (panels A, B) or **7** (panels C, D). Cells were either grown continuously in the presence of a concentration of 2× or 5× EC_50_ of test compound (i.e., 3.8 nM and 1.56 nM respectively), or without test compound as a control (panels A,C). Alternatively, the cells were washed into fresh medium without test compound at the indicated time (panels B,D), in order to test the reversibility of the action of the compound. Cells were counted under phase contrast microscopy using a hemocytometer, samples from three independent cultures grown in parallel. Symbols show the average and SEM of three independent determinations. The dashed red line indicates the limit of detection by hemocytometer.

The results at 48 h indicated that both compounds exhibit time- and dose-dependent effects on *T. brucei* viability. At 2× EC_50_ and 5× EC_50_, both **5** and **7** (Figure 3, panels A and C) caused significant growth inhibition and cell death over time. In the case of **5**, higher concentrations led to a collapse in the trypanosome population between 32 and 40 h of exposure to 5× EC_50_ **5**, whereas this took between 40 and 48 h at the lower concentration of 2× EC_50_ (panel A). The difference in response to the two dosages was less pronounced for **7** (Panel C), with apparent clearance of the population (to the limit of detection) after 40 h. Washing the drug out from the culture media after 8 h restored the cells to normal growth (**5** and **7**); washing after 16 h (**5**) also enabled growth, but after a lag phase and to a reduced maximum density (**5**) (panel B). However, an exposure of 24 h (**7**) could not be fully reversed as the cells did not resume growth, even after a further 48 h incubation in drug-free culture medium (panel D). These findings suggest that exposure time plays a critical role in the reversibility effects of these compounds. At 5× EC_50_ (just 3.8 nM and 1.56 nM for **5** and **7**, respectively) the drugs clearly have a rapid growth-inhibitory effect and the damage to the cells becomes irreversible after approximately 24 h, followed by cell death at 40 h of exposure.

### Mode of Action studies

#### Propidium Iodide Assays of Cellular Permeability

Based on the collected results, compound **5** was selected as the most promising member of the phosphonium salts series. This choice was further supported by its derivation from the cardanol mixture—the most abundant component of CNSL (60–70%)—making it particularly attractive from both sustainability and cost perspectives. Cost is a critical factor in developing treatments for neglected tropical diseases, especially animal trypanosomiasis.

Propidium iodide (PI) assays assessing plasma membrane integrity were performed to monitor the effects of **5** on *T. b. brucei* survival in real time (Figure 4).^58^

**Figure 4:**
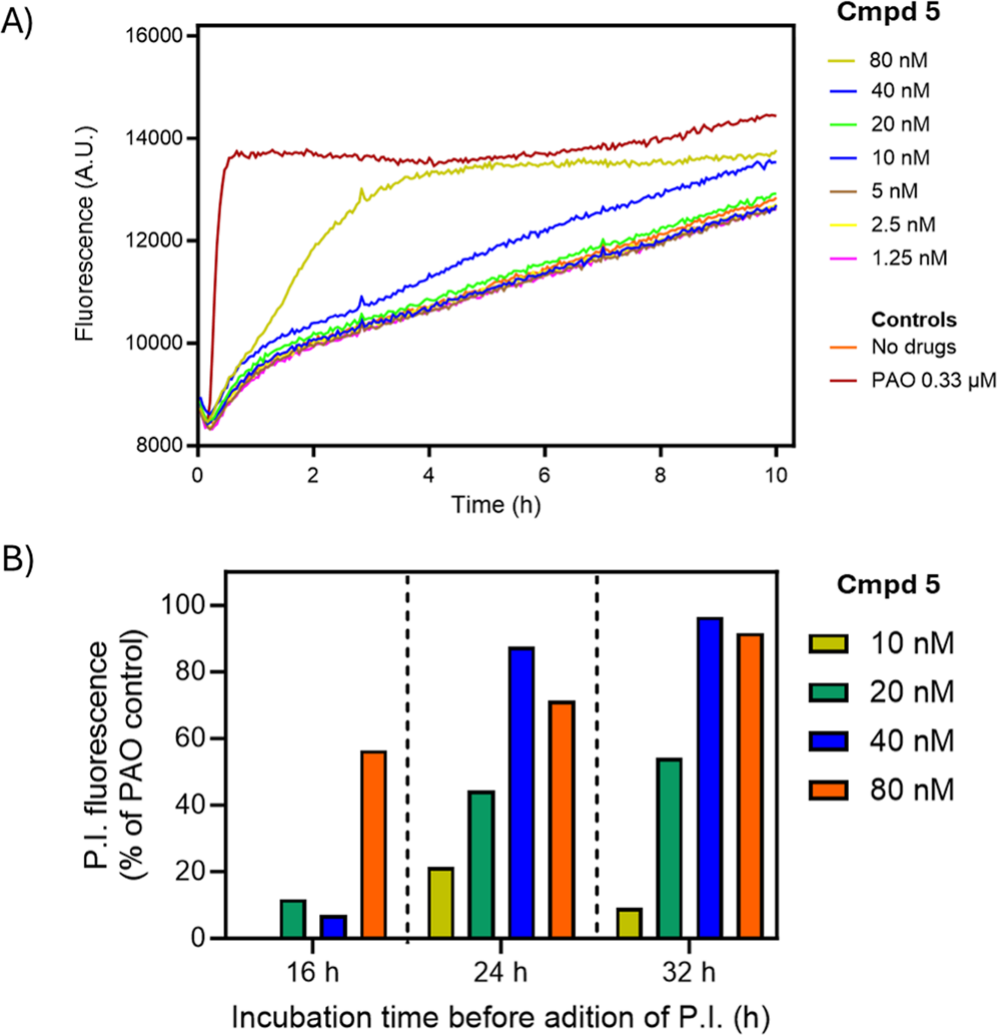
Propidium iodide assays of cellular permeability in *T. b. brucei*. (A) *T. b. brucei* bloodstream forms were incubated with various concentrations of **5**, and the arsenical phenylarsine oxide (PAO) as positive control, in 96 well plates at 37 °C and 5% CO_2_. The fluorescence of propidium was monitored for 10 h. A.U., artificial units of fluorescence. (B) *T. b. brucei* bloodstream forms were incubated with various concentrations of **5** in an incubator at 37 °C and 5% CO_2_. At the indicated time points after the addition of **5**, samples were taken, cell densities were normalized and 100 μL added to a well of a 96-well plate containing propidium iodide. The fluorescence was recorded for 30 min and the no-drug control was taken as 0% whereas the cells lysed with PAO were taken as 100%.

The membrane-perturbing effects of **5** on *T. brucei* S427 trypomastigotes were dose-dependent (Figure 4A). These results show that increasing concentrations of **5** lead to a significant increase in membrane permeability, as indicated by a rise in fluorescence but over the course of the 10 h experiment, recorded in the continuous presence of PI, only 40 nM and 80 nM lead to an increase of fluorescence over background (caused by the very slow uptake of PI by endocytosis).^59^ This demonstrates that **5** disrupts the integrity of the parasite’s membrane in a matter of hours, similar to the positive control phenylarsine oxide (PAO), a known membrane-permeabilizing agent,^58^ but only at concentrations that represent approximately 50 – 100× EC_50_, while lower concentrations clearly take longer to affect cellular permeability and viability, consistent with the growth curves presented in Figure 3.

As evident from Figure 4A, the slow rise in background PI fluorescence precludes an experimental exposure above 10 h at the very most. We therefore incubated the cells again with various concentrations of **5**, now in the absence of PI, except for the last 30 min before the present measuring point. This allowed us a read-out for PI fluorescence based on cellular permeation rather than slow uptake, at 16 h, 24 and 32 h (Figure 4B). This experiment clearly shows dose-dependent PI ingress, reaching almost 100% of the PAO control at 24 and 32 h with 40 nM and 80 nM, with 80 nM also causing a strong signal at 16 h. The signal for 20 nM was 11.8% at 16 h, rising to 44.3% and 54.1% at 24 and 32 h, respectively.

These findings are consistent with the hypothesis that **5** causes cell death by compromising the membrane, although it is more likely that the measurements represent loss of membrane integrity upon cell death. The slow progression of fluorescence increases, particularly at concentrations shown to inhibit growth (Figure 3), reinforce the notion that these exert a predominantly trypanostatic effect and that the more rapid cell death seen at >50× EC_50_ may represent a different type of toxicity that is less specific to the targeted pathogen. It thus appears that there is, for *T. brucei* at a minimum, a safe window of specific drug action because of the very low EC_50_ values, but that this may become a more rapid mechanism, possibly by membrane disruption, at much higher concentrations.

#### Preliminary Investigation of TAO as Potential Target

As previously discussed, respiration of trypomastigotes of *T. brucei* relies on an alternative oxidase known as the TAO.^60^ In recent years, different series of TAO inhibitors based on the structure of the traditional inhibitor SHAM have been reported by the research groups of Dardonville and De Koning. These molecules, when conjugated with a mitochondrion-targeting lipophilic cation tail via a methylene linker led to very potent trypanocides against wild-type and multidrug-resistant *T. brucei* strains.^32^

Building on these findings and in order to evaluate TAO as a potential target of the CNSL-based phosphonium series, a *T. brucei* cell line overexpressing TAO (*Tb*TAO^oe^) was used in parallel with other strains to determine the EC_50_ in comparison with the EC_50_ values obtained with the wild-type control. In these experimental conditions, TAO inhibitors were expected to display reduced activity against *Tb*TAO^oe^, whereas compounds that predominantly act with a different mode of action (MoA) against the parasite, display an unchanged activity.^32^

Hence, selected compounds were tested on a *T. b. brucei* cell line overexpressing TAO, to further investigate the potential MoA of CNSL-based phosphonium salts (Table 6). In a side-by-side comparison, all compounds tested, **5, 6**–**8** and **10–11**, displayed significantly higher EC_50_ values (from 1.94- to 5.42-fold; all P < 0.001) against *Tb*TAO^oe^, similarly to TAO-inhibitor SHAM and consistent with earlier tests with TAO inhibitors (Table 6);^32^ PMD activity was not significantly different against the two cell lines (RF = 0.76; *P* = 0.24). This pointed to a possible involvement of TAO in the MoA of the tested compounds. In addition to this, the substantial drop in activity against *T. congolense* strains was consistent with this interpretation, as these parasites are much less dependent on TAO for their energy metabolism.^61^

**Table 6:** Evaluation of the Test Compounds Activity (EC_50_, Mean ± SEM, nM) on Trypomastigotes Wild-Type and *T. brucei* Cell Line Over-Expressing TAO

	*T.b. brucei*		
	EC_50_ ^a^± SEM^b^ (nM)		
cmpd	WT s427^c^	TAO oe^d^	RF^e^
**5**	6.20 ± 0.60	22.87 ± 0.28	3.69
**6**	7.34 ± 0.63	39.80 ± 1.02	5.42
**7**	17.48 ± 1.33	38.37 ± 0.57	2.20
**8**	11.44 ± 0.63	42.38 ± 1.01	3.70
**10**	18.05 ± 1.32	45.29 ± 0.18	2.51
**11**	5.13 ± 0.32	9.94 ± 0.33	1.94
**PMD** ^f^	1.70 ± 0.26	1.28 ± 0.02	0.76
**SHAM** ^g^	42.24 ± 2.81	136.53 ± 4.77	3.23

a EC_50_: 50% effective concentration.

b SEM: standard error of the mean.

c Trypomastigotes of *T. b. brucei* s427.

d *T. brucei* overexpressing TAO.

e Resistance factor relative to WT.

f PMD: pentamidine.

g SHAM: salicylhydroxamic acid.

As seen from the collective data in Tables 1–4, trypanosomes of the Trypanozoon subgenus (*T. brucei*, *T. b. evansi*, *T. b. equiperdum*), all sharing a similar mitochondrial metabolism, are highly sensitive to our test compounds. In contrast, *T. congolense* (subgenus Nannomonas) and *T. vivax* (subgenus Duttonella), while expressing a functional TAO orthologue, feature a more complex mitochondrion that renders them less susceptible to TAO inhibitors,^61^ and TAO inhibitors have consistently been observed to be less potent against *T. congolense* *in vitro*.^32,34^ Alternative Oxidase has been less studied in *T. cruzi* and in *Leishmania* species,^62^ but is not believed to be a valid drug target in these parasites as it is much less essential in their metabolism and may in fact be inactive.^61^ Thus, the pattern of highly potent activity against *T. brucei*, *T. b. evansi* and *T. b. equiperdum* but more modest activity against the other kinetoplastids correlates very well with the role of TAO in their respective energy metabolisms. The pattern on *T. brucei* growth for **5** and **7** was also highly similar to that of a known, very potent inhibitor of TAO (IC_50_ for recombinant TAO is 1.3 nM), which displayed a dose-dependent period of 24–36 h of trypanostatic effects followed by a second phase of trypanosomal cell death;^34^ wash-out of the drug after 24 h did not reverse the compound’s effect, due to cell cycle arrest in the G1 phase.^34^ Due to the common presence of the TPP^+^ moiety and the overall structure similarity, it is possible that the two classes of compounds may act similarly by inhibiting TAO. The activity against other trypanosomatids, as well as the toxicity against some mammalian cell types, is likely attributable to a distinct, less specific action at much higher concentrations, as also shown in the propidium iodide experiments depicted in Figure 5.

**Figure 5:**
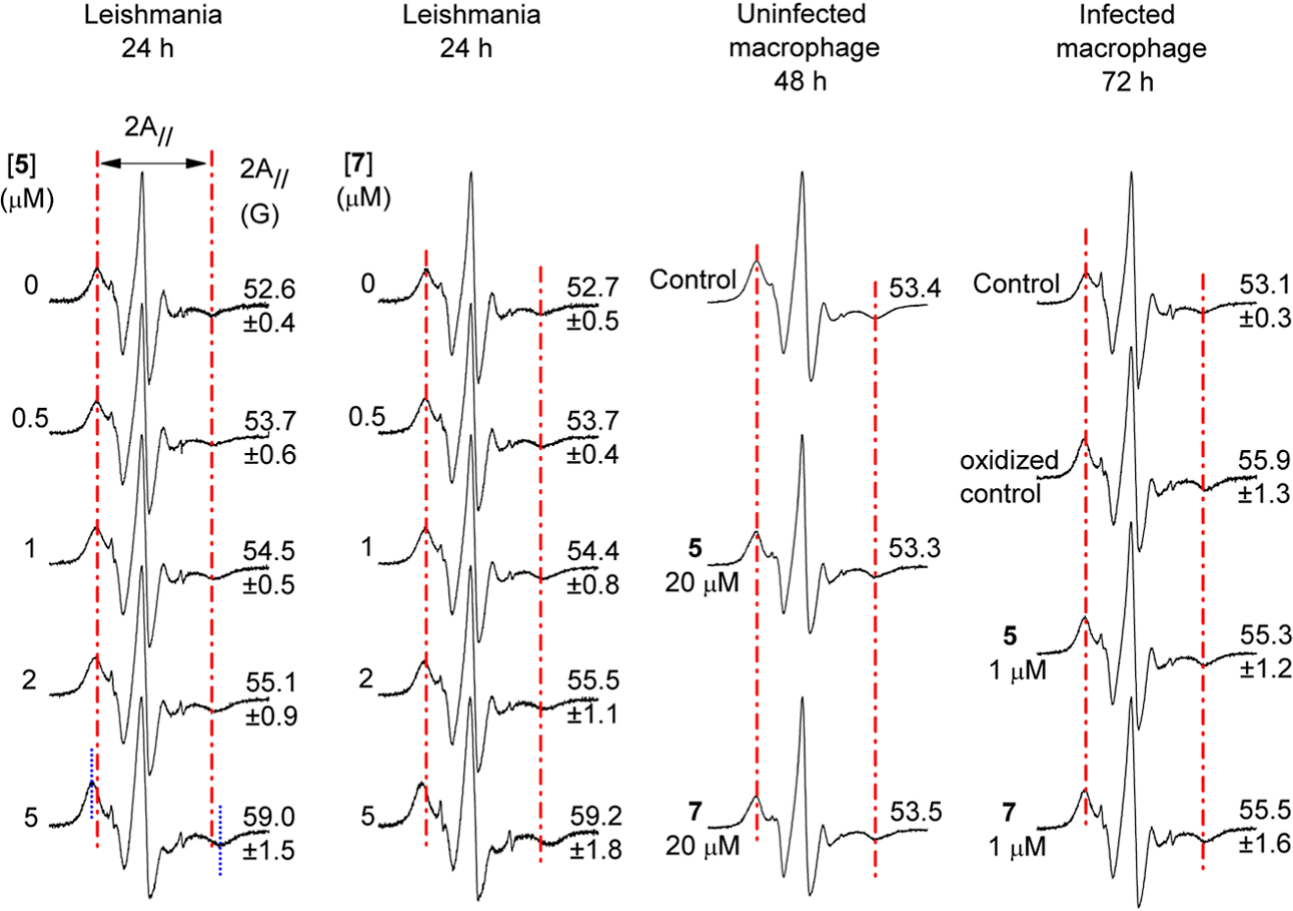
Representative EPR spectra of the spin label 5-DSA incorporated into the plasma membranes of *L. amazonensis* promastigotes and J774.A1 macrophages showing untreated samples and those treated with compounds **5** and **7** at the indicated concentrations. The mean ± SD values of the EPR parameter 2A_//_ (outer hyperfine splitting) are provided for each EPR spectrum. 2A_//_ is measured directly from the EPR spectrum and is defined as the magnetic field separation between the first and last inverted peaks, indicated by the vertical red lines. The SD is not shown for uninfected macrophages because the compounds do not cause membrane alteration even at high concentrations. All means were significantly different in the first two columns from those of the control samples (without treatment) at *P* < 0.05. In the third column, there was no change, and in the fourth column, the means were different from the mean of control. The intensity of the spectra is given in arbitrary units (*Y*-axis), and the total scan range of the magnetic field in each EPR spectrum was 100 G (*X*-axis).

#### Evaluation of Membrane-Perturbing Activity of Compound **5** and **7**

In light of the collected results, the amphiphilic nature of our compounds and previous findings with CNSL-derived MIL analogues indicating interactions at the lipid–protein interface,^36^ we investigated the membrane-perturbing activity of compounds **5** and **7** in *L. amazonensis*.

Initially, their leishmanicidal and cytotoxic effects were assessed, in comparison with MIL as a reference drug. As shown in Table 7, compounds **5** and **7** exhibited significant activity against *L. amazonensis* promastigotes, with EC_50_ values around 1 μM, outperforming MIL, which had an EC_50_ of 10 μM. However, cytotoxicity against J774.A1 macrophages yielded CC_50_ values of approximately 10 μM for compounds **5** and **7**, compared to 80 μM for MIL.

**Table 7:** Antiproliferative Activity against the Protozoan Parasite *L. amazonensis*, Cytotoxicity in J774A.1 Macrophage and Hemolytic Potential^h^

	cytotoxicity				hemolysis	
	*L. amazonensis* ^a^	non infected macrophage^b^	infected macrophage^c^			
cmpd	EC_50_ ^e^± SD^f^ (μM)	CC_50_ ^g^± SD^f^ (μM)		SI^d^	HC_50i_ ± SD^f^ (μM)	SI^d^
**5**	1.01 ± 0.27	10.2 ± 1.9	10.7 ± 2.9	10	21.0 ± 1.7	21
**7**	0.98 ± 0.31	10.0 ± 2.2	10.3 ± 3.2	10	20.5 ± 3.1	21
**MIL** ^g^	9.8 ± 2.4	81 ± 18	86 ± 21	8	10.3 ± 4.1	10

a *L. amazonensis* (IFLA/BR/67/PH8 or 75/Josefa reference strain).

b J774A.1 macrophage (48 h).

c *L. amazonensis* infected J774A.1 macrophage 48 h postinfection.

d SI: selectivity index (CC_50_/EC_50_ or HC_50_/EC_50_).

e EC_50_ = the concentration required to inhibit 50% of the parasites.

f SD: standard deviation.

g CC_50_ = the concentration required to cause cytotoxicity in 50% of the cells.

h HC_50_ = concentration required to hemolyze 50% of erythrocytes. Statistical significance: the means for the **5** and **7** compounds are not significantly different from each other, but both are significantly different from the means for MIL (*P* < 0.05).

To evaluate potential membrane disruption in human cells, the hemolytic activity of the compounds was measured (Table 7). Compounds **5** and **7** both exhibited hemolytic effects, though at lower levels than MIL and both compounds showed a SI of 10, slightly higher than MIL’s SI of 8. Notably, when comparing activity against parasites versus erythrocytes (EC_50_/HC_50_), **5** and **7** demonstrated an SI of 21, surpassing MIL’s SI of 10 (Table 7).

We also exploited spin-probe electron paramagnetic resonance (EPR) spectroscopy for assessing lipid peroxidation and protein oxidation in biological membranes, by monitoring the membrane rigidity resulting from eventual oxidative processes. Figure 5 shows the EPR spectra of spin label 5-DSA incorporated into the plasma membrane of *L. amazonensis* promastigotes, comparing untreated cells to those treated with different concentrations of **5** and **7**.

The parasite membrane became more rigid in the treated samples, as indicated by the increase in the mean value of the 2A_//_ parameter. For 24 h exposure, this effect was observed even at compound concentrations as low as 0.5 μM, which corresponds to 0.5 times the IC_50_ values. At the concentration of 5 μM, there was an increase in 2A_//_ of approximately 6 G, indicating a notable decrease in membrane fluidity. Interestingly, no alteration in membrane fluidity was observed in macrophages, even at very high concentrations of the compounds (200 μM). To examine whether the compounds could alter the membrane dynamics of the *Leishmania*-infected macrophages, we performed a general spin labeling on the membranes within this macrophage system. However, it is expected that the spin label only accesses the external membranes of the macrophage-amastigote system. This is due to the nitroxide moiety of the spin probe is reduced and losing its EPR signal upon entering the cell. The nitroxide radical can be reduced by cytoplasmic agents such as ascorbic acid, Fe^2+^, or free thiol groups. Furthermore, EPR measurements are performed within 15 min of spin labeling to prevent partial signal loss.

Figure 5 presents EPR spectra from two control groups of macrophages infected with *Leishmania* for 72 h in column 4. In control, no membrane alterations were observed in infected macrophages. Upon treatment with compounds **5** and **7** at 1× EC_50_ for promastigotes, membrane rigidity increased modestly, indicated by a shift in 2A_//_ from ∼53 to ∼55 G. In oxidized control, significant membrane rigidity developed after 72 h of infection, even without compound exposure. Further experiments revealed that membrane changes typically occur only when macrophage quality is suboptimal, characterized by poor adhesion and size heterogeneity, whereas high-quality macrophages maintain stable membrane fluidity even after prolonged infection.

### Ecotoxicity

#### In Silico Acute Ecotoxicity Predictions of Compounds **5** and **7**

The development of eco-friendly, sustainable products to combat parasitic diseases is a growing priority in modern healthcare. In this context, it is crucial to design antiparasitic agents that not only effectively reduce parasite burdens but also minimize environmental impact, ensuring longer term safety and cost-effectiveness.^14^ This strategy aligns with the One Health approach, which is essential not only for advancing human and animal health but also for addressing the environmental concerns associated with conventional antiparasitic drugs. Despite the potential ecotoxicity of commercially available antileishmanial and antitrypanosomatid drugs, comprehensive studies evaluating their environmental impact from a One Health perspective remain limited.^14,63^

Based on reported *in vitro* and *ex vivo* results, compounds **5** and **7** were identified as the most promising candidates due to the observed antiparasitic activity. We initially predicted aquatic toxicity (e.g., fish, Daphnid, and green algae) of compounds **5** and **7** using the in silico model ECOSAR v2.2 (Supporting Information),^64^ and observed the predicted aquatic toxicity of compounds **5** and **7** to be moderately toxic to aquatic life (Table S3).^65^ Specifically, the estimated fish 96 h LC_50_ was 1.95 mg/L and 1.73 mg/L for compounds **5** and **7**, respectively (Table S3). Similar toxicity values were predicted for Daphnid and green algae, with 48 h and 96 h EC_50_ values at 1.41 and 2.88 mg/L for compound **5** and 1.27 and 2.67 mg/L for compound **7**, respectively (Table S3). Thus, these in silico predictions suggested that compound **7** was slightly more toxic than compound **5** in fish, Daphnid, and green algae.

#### *In Vivo* Acute Ecotoxicity Evaluation of Compounds **5** and **7**

Based on the predicted aquatic toxicity results for compounds **5** and **7**, preliminary 96 h or 48 h acute toxicity bioassays were performed using embryo-larval zebrafish (*Danio rerio*) and *Daphnia magna* (*D. magna*) models, respectively. The observed 96 h LC_50_ and 48 h EC_50_ values were 0.52 mg/L and 0.33 mg/L for compound **5**, and 0.97 mg/L and 0.29 mg/L for compound **7**, respectively. When comparing these *in vivo* results to the in silico predictions, the experimental data showed higher sensitivity than the estimations based on computational models (Tables 8 and S3).

**Table 8:** Acute Toxicity (LC_50_ or EC_50_ Values) of Compounds **5** and **7** to Embryo-Larval Zebrafish (*Danio rerio*) (96 h), and *Daphnia magna* Neonates (48 h)

				model organism		
				Danio rerio	Daphnia magna	
compound	MW (g/mol)	log *K*_ow_ ^a^	solubility in water (mg/mL)	96 h LC_50_ (mg/L; nM)	24 h EC_50_ (mg/L; nM)	48 h EC_50_ (mg/L; nM)
**5**	561.53	4.64	0.03	0.52; 926.04	0.33; 594.80	0.17; 308.08
**7**	591.56	4.73	0.02	0.97; 1644.80	0.29; 483.47	0.29; 483.47

a *K*_ow_^a^: octanol–water partition coefficient.

Moreover, the in silico model predicted compound **7** to be slightly more toxic than compound **5** in both zebrafish and *D. magna*, highlighting the necessity of complementing quantitative structure–activity relationship (QSAR)-based predictions—typically reliant on log *K*_ow_ values—with empirical *in vivo* validation for biologically active molecules. Importantly, the acute aquatic toxicity of compounds **5** and **7** toward common fish and invertebrate models was lower than their efficacy against the parasites studied here. For example, EC_50_ values of compound **5** to *L. mexicana* and *L. amazonensis* were 170 nM and 370 nM (Table 5), respectively, but zebrafish LC_50_ and *D. magna* EC_50_ values were 926 nM and 308 nM, respectively (Table 8). Similarly, EC_50_ values for compound **7** were 190 nM and 340 nM for *L. mexicana* and *L. amazonensis* (Table 5), but again zebrafish LC_50_ and *D. magna* EC_50_ values were higher at 1645 nM and 483 nM, respectively (Table 8). Comparable trends were observed for *Trypanosoma* spp., underscoring the favorable therapeutic index of these compounds. Such comparative sensitivity differences are promising for developing these new antiparasitic medications with reduced environmental impact. It is also important to note that the aquatic toxicity estimates for compounds **5** and **7** in zebrafish and *D. magna* result in classification of these medications as chemicals of high toxicity (Tables 8 and S2).^65^ However, in the present study we have only examined predicted and measured acute toxicity, but environmental risk is also dependent on a number of factors influencing exposure and chronic toxicity. Consequently, future studies are needed to develop an understanding of environmental fate, including persistence, mobility and bioaccumulation, of these promising antiparasitic compounds.

## Conclusions

Antiparasitic drug discovery remains critical in combating infectious diseases affecting humans and animals, with growing attention to sustainability. Traditional treatments, especially for VBPDs, often fall short, highlighting the need for innovative alternatives with reduced environmental impacts. Furthermore, any new drugs should be from novel chemical classes and not cross-resistant with the drugs they are meant to replace. This study explores CNSL as a biobased starting material for developing novel sustainable antiparasitic agents.

We applied a mitochondrion-targeting design strategy to synthesize new biobased phosphonium and ammonium salts by combining short-chain (C8) CNSL derivatives with lipophilic cations. This strategy led to the discovery of several highly potent phosphonium salts against *Trypanosoma* and *Leishmania* spp., exhibiting excellent selectivity indices (>1000) and no apparent cross-resistance with current trypanocidal drugs. It proved significantly more effective than our previously reported approach based on conjugation with a quinone scaffold.^21^ Notably, compounds **5** and **7**—derived from cardanol and cardol scaffolds, respectively—demonstrated strong trypanocidal effects.

Promising *ex vivo* results further supported their efficacy across various parasite species, suggesting potential broad-spectrum antiparasitic activity beyond *in vitro* settings, although the highest selectivity indices were found for parasites of the Trypanozoon subgenus, including *T. b. evansi* and *T. b. equiperdum*, responsible for the deadly veterinary conditions surra and dourine, respectively, across most continents. While these findings are encouraging, additional studies are needed to fully elucidate their mechanism of action and therapeutic potential.

In light of mounting environmental concerns surrounding antiparasitic drugs,^63^ a preliminary ecotoxicological evaluation of compounds **5** and **7** was perfomed in aquatic species. To the best of our knowledge, this is the first study to integrate both in silico predictions and *in vivo* testing using zebrafish and *D. magna* models during the early stages of the drug discovery pipeline. Notably, the acute toxicity results diverged from the in silico predictions, underscoring the need for integrated approaches to improve the accuracy of ecotoxicological assessments in drug development.^66^ Nonetheless, experimental validation remains essential. Though the acute toxicity of compounds **5** and **7** would be categorized as high by regulatory agencies, it is important to note that their toxicity toward the parasites studied here was significantly greater. Future efforts are necessary to examine the factors influencing the exposure (e.g., persistence, mobility, bioaccumulation) and environmental risks of these potentially promising therapeutics in terrestrial and aquatic systems. It is anticipated that this will quickly lead to application of standardized ecotoxicological assays, including a scale with acceptable and dangerous ranges, as advocated by COST Action CA21111, OneHealthDrugs.^14,63^

In summary, CNSL-derived molecules represent a sustainable and effective platform for antiparasitic drug development. Their strong activity against multiple parasite species, favorable toxicity profile, and origin from a renewable agro-industrial byproduct align with the principles of One Health and Green Chemistry. Continued research will be essential to optimize these compounds and advance their development for both human and veterinary applications against VBPDs.

## Experimental Part

### Chemistry

All chemicals were purchased from Aldrich Chemistry (Milan, Italy), Alfa Aesar (Milan, Italy) and FluoroChem (Cambridge, UK) were of the highest purity. Technical CNSL mixtures were kindly gifted by Resibras (Brazil or Portugal). Solvents were of analytical grade. Reaction progress was followed by thin layer chromatography on precoated silica gel 60 F254 plates (Merck, Darmstad, Germany). Chromatographic separations were performed on 0.040–0.063 mm silica gel 40 columns via the flash method (Merck) or by automated flash column chromatography using a Teledyne Isco CombiFlash Rf or Rf200i with Normal Phase RediSep Rf Disposable Columns. All ^1^H, ^13^C and ^31^P nuclear magnetic resonance (NMR) spectra were recorded at ambient temperature on either a Varian Gemini spectrometer (Varian Medical System Italia, Milan, Italy) at 400 (^1^H) and 101 (^13^C and ^31^P) MHz, or on a Bruker Ascend 600 spectrometer. Chemicals shifts (δ) are reported in parts per million (ppm) relative to SiMe_4_ and referenced to the residual solvent peak for ^1^H (7.26 ppm in CDCl_3_, 3.31 ppm in CD_3_OD, 2.50 ppm in DMSO-*d*_6_) and ^13^C NMR spectra (77.15 ppm in CDCl_3_, 49.00 ppm in CD^3^OD, 39.51 ppm in DMSO-*d*_6_). Coupling patterns are described as singlet (s), doublet (d), triplet (t), quartet (q), quintet (quint.), multiplet (m), broad (br), or a combination of the listed splitting patterns. Coupling constants (*J*) are given in Hertz (Hz). Ultra-HPLC–mass spectrometry analyses were run on a Waters ACQUITY Arc system (Milan, Italy) consisting of a QDa mass spectrometer equipped with an electrospray ionization interface and a 2489 UV/vis detector. The detected wavelengths were 254 and 365 nm. Analyses were performed on an XBridge BEH C18 column with a 10 × 2.1 mm internal diameter (particle size 2.5 μm) with an XBridge BEH C18 VanGuard Cartridge precolumn with a 5 × 2.1 mm internal diameter (particle size 1.8 μm) (Waters). The mobile phases were H_2_O (0.1% formic acid) and MeCN (0.1% formic acid). Electrospray ionization in positive and negative mode was applied in the mass scan range of 50–1200 Da. The authors used a generic method and linear gradient: 0–0.78 min, 20% B; 0.78–2.87 min, 20–95% B; 2.87–3.54 min, 95% B; 3.54–3.65 min, 95–20% B; 3.65–5.73, 20% B; and a lipophilic method and linear gradient: 0–0.78 min, 40% B, 0.78–2.87 min, 40–95% B; 2.87–3.54 min, 95% B; 3.54–3.65 min, 95–40% B; 3.65–5.73, 40% B. The flow rate was 0.8 mL/min. HRMS spectra were recorded on a Waters Xevo G2-XS quadrupole time-of-flight apparatus operating in electrospray mode. Compounds were named based on the naming algorithm developed by CambridgeSoft and used in ChemBioDraw Ultra (PerkinElmer, Milan, Italy, version 20.0). All of the biologically tested compounds were >95% pure by HPLC.

#### Purification of Cardanols (1) and Cardols (2) Mixtures from Technical CNSL

20.0 g of *t*CNSL donated from the company Resibras was purified by silica gel column chromatography (hexane/ethyl acetate, 95:5 → 65:35) to provide 14.0 g of the mixture of cardanols (70% of the applied mass) and 4.8 g of the mixture of cardols (24% of the applied mass).

#### Extraction of Anacardic Acid (3) Mixtures from Natural CNSL

A solution of 15.0 g of calcium hydroxide in methanol/water (6:1, 210 mL) was added to 30.0 g of *n*CNSL. The system was stirred at 60 °C for 3 h. After this period, the mixture was concentrated under vacuum and filtered. The solid was transferred to a 1 L Erlenmeyer in which were added ethyl acetate (150 mL), distilled water (50 mL), and 50% HCl solution to reach pH = 1.0. The resulting solution was washed with saturated sodium chloride solution (50 mL) and dried over anhydrous Na_2_SO_4_. The solvent was evaporated under reduced pressure, and the mixture was purified by silica gel column chromatography (hexane/ethyl acetate, 100:0 → 70:30), giving 16.5 g of the anacardic acid mixture, corresponding to approximately 55% of the mass of *n*CNSL used.

### General Procedure a (for Compounds **5–10**)

A 5 mL round-bottom flask was charged with a stir bar and appropriate bromide (0.5 mmol, 1.0 equiv). Triphenylphosphine (for compounds **5–7** and **2.35**) or diphenyl-2-pyridylphosphine (for compounds **8–10**) (1.0 equiv) were added in one portion, then the reaction was heated to 80 °C under nitrogen for 2 h. The resulting viscous oil was purified by silica gel column chromatography. First, with dichloromethane/ethanol (100:0), until removal of unreacted phosphine and phosphine oxide (byproduct), then dichloromethane/ethanol (95:5) to obtain the target compound.

### General Procedure B (for Compounds **11**–**13**)

A 10 mL round-bottom flask was charged with a stir bar and appropriate bromide (0.6 mmol, 1.0 equiv). Tris­(naphtalen-1-yl) phosphine (1.0 equiv) was added in one portion, followed by the addition of dimethylformamide (3.0 mL). The reaction was heated at 110 °C under nitrogen for 40 h. After allowing it to cool to room temperature, the solvent was removed under vacuum. The resulting viscous oil was purified by silica gel column chromatography. First, with dichloromethane/ethanol (100:0), until removal of unreacted phosphine and phosphine oxide (byproduct), then dichloromethane/ethanol (95:5) to obtain the target compound.

### General Procedure C (for Compounds **14**–**22**)

A 5 mL round-bottom flask was charged with a stir bar and appropriate bromide (0.5 mmol, 1.0 equiv). Pyridine (for compounds **14–16**), 4-methylpyridine (for compounds **17–19**), or 1-methylimidazole (for compounds **20–22**) (4.0 equiv) were added in one portion, then the reaction was heated to 80 °C under nitrogen for 2 h. The resulting viscous oil is triturated primarily to remove excess starting material. The crude product is dissolved in the minimal amount of dichloromethane, followed by the dropwise addition of diethyl ether. A sticky solid precipitate is observed, and the supernatant is carefully removed. The precipitate was washed twice with diethyl ether, then any residual solvent is removed under high vacuum.

### General Procedure D (for Compounds **23–25**)

A 10 mL round-bottom flask was charged with a stir bar and appropriate bromide (0.6 mmol, 1.0 equiv). 4-Methylpyridine (1.0 equiv) was added in one portion, followed by the addition of acetonitrile (3.0 mL). The reaction was heated at 80 °C under nitrogen for 2 h. After allowing it to cool to room temperature, the solvent was removed under vacuum. The resulting viscous oil is triturated. The crude product is dissolved in the minimal amount of dichloromethane, followed by the dropwise addition of diethyl ether. A sticky solid or oily precipitate is observed, and the supernatant is carefully removed. The precipitate was washed twice with diethyl ether, then any residual solvent is removed under high vacuum.

### General Procedure F (for Compounds **35–37**)

Mixtures of cardanols **1** (10.0 g, 33.5 mmol), or cardols **2** (6.0 g, 18.7 mmol) or anacardic acids **3** (12.5 g, 36.5 mmol) were stirred with potassium carbonate (3.0 equiv) in acetone (300 mL). After 2 h, methyl iodide (4.0 or 6.0 equiv) was added, and the reaction was refluxed at 110 °C with a cooling system at −8 °C for 24 h. After cooling, the solvent was evaporated in vacuo. Distilled water (40 mL) was added, and the residue was extracted with dichloromethane (3 × 30 mL). The combined organic phases were washed with 10% HCl solution (10 mL) and brine solution (10 mL), dried over anhydrous Na_2_SO_4_, filtered, and concentrated under vacuum. The crude product was purified by column chromatography (hexane/ethyl acetate, 95:5 → 80:20), providing the unchanged mixture of saturated derivatives and the mixtures of O-methylcardanols (**32**, 80%), *O*-methylated methylanacardates (**33**, 74%), *O*,*O*-dimethylcardols (**34**, 66%) with different unsaturation degrees as brown oils. The mixtures have been used for the further step without characterization.

### General Procedure G (for Compounds **29**–**31**)

To an ozonation flask was added a solution (5.0 mmol, 1.0 equiv) of the *O*-methylatedcardanol **32** or *O*-methylated methylanacardate mixture **33** or *O*,*O*-dimethylated cardol **34** in dichloromethane/methanol (1:1, 60 mL) (see Supporting Information). The reaction was cooled at 0 °C with an ice–water bath, and the solution was treated with ozone for approximately 3 h. After purging the reaction mixture with nitrogen, NaBH_4_ (20.0 mmol, 4.0 equiv) was added, and the mixture was left warming to room temperature, under vigorous stirring for 24 h. The reaction was quenched with water and 10% aqueous HCl (10 mL) and extracted with dichloromethane (3 × 30 mL). The combined organic layers were washed with brine solution (10 mL), dried over anhydrous Na_2_SO_4_, and evaporated. The residue was purified by chromatography (petroleum ether/toluene/ethyl acetate), providing the corresponding alcohol.

### General Procedure H (for Compound **26–28**)

Compounds **29–31** (0.7 mmol, 1.0 equiv), triethylamine (1.3 equiv) and a catalytic amount of 4-dimethylaminopyridine were stirred in 2-methyltetrahydrofuran (0.35 mL) at 0 °C for 15 min. Then, methanesulfonyl chloride (1.3 equiv) was added dropwise to the mixture and stirred at room temperature for 3 h. The reaction was quenched with water and extracted with ethyl acetate (3 × 10 mL). The organic layers were collected, dried over anhydrous Na_2_SO_4_, filtered, and concentrated under vacuum. The crude products were purified by silica gel column chromatography.

### General Procedure I (for Compounds **32**–**34**)

The appropriate mesylated compound (2.5 mmol, 1.0 equiv) was taken up in 6.3 mL of acetone. Lithium bromide (3.0 equiv) was added in one portion, and the reaction mixture was heated to reflux for 2 h. After allowing it to cool to room temperature, the reaction mixture was partitioned between 15 mL ethyl acetate and 10 mL of distilled water. The aqueous layer was additionally extracted twice (2 × 15 mL), then the combined organic layers were washed with brine solution, dried over Na_2_SO_4_, filtered, and concentrated under vacuum. The crude products were purified by silica gel column chromatography.

#### (8-(3-Methoxyphenyl)­octyl)­triphenylphosphonium Bromide (**5**)

Following general procedure **A**, the desired compound was obtained from **32** (48.9 mg, 0.2 mmol) and triphenylphosphine (42.8 mg, 0.2 mmol). Purification by silica gel column chromatography (dichloromethane/ethanol, 100:0 → 95:5) yielded to **5** as a whitish sticky solid. Yield 87%. ^1^H NMR (400 MHz, CDCl_3_): δ 7.87–7.67 (m, 15H), 7.17 (td, *J* = 7.3, 1.9 Hz, 1H), 6.74–6.69 (m, 3H), 3.85–3.82 (m, 2H), 3.78 (s, 3H), 2.54–2.50 (m, 2H), 1.70 (br s, 2H), 1.55–1.51 (m, 2H), 1.24–1.23 (m, 6H). ^13^C NMR (101 MHz, CDCl_3_): δ 159.6, 144.6, 135.1 (d, *J* = 3.1 Hz), 133.8 (d, *J* = 9.9 Hz), 130.5 (d, *J* = 12.5 Hz), 129.2, 120.9, 118.9, 118.0, 114.2, 110.9, 55.2, 36.00, 31.3, 30.5, 30.4, 29.1 (d, *J* = 9.1 Hz), 23.1, 22.6. ^31^P NMR (101 MHz, CDCl_3_): δ 24.98. LCMS *m*/*z* [M]^+^ 481.38. HRMS (ESI) *m*/*z*: calcd for [M]^+^, 481.2655; observed, 481.2653.

#### (8-(3-Methoxy-2-(methoxycarbonyl)­phenyl)­octyl)­triphenylphosphonium Bromide (**6**)

Following general procedure **A**, the desired compound was obtained from **33** (40.0 mg, 0.1 mmol) and triphenylphosphine (29.4 mg, 0.1 mmol). Purification by silica gel column chromatography (dichloromethane/ethanol, 100:0 → 95:5) yielded to **6** as a pink sticky solid. Yield 83%. ^1^H NMR (400 MHz, CDCl_3_): δ 7.81–7.74 (m, 9H), 7.67–7.64 (m, 6H), 7.24–7.20 (m, 1H), 6.73 (ddd, *J* = 15.4, 8.1, 0.9 Hz, 2H), 3.83 (s, 3H), 3.76 (s, 3H), 3.70–3.64 (m, 2H), 2.46–2.42 (m, 2H), 1.59–1.56 (m, 4H), 1.50–1.42 (m, 2H), 1.23–1.15 (m, 6H). ^13^C NMR (101 MHz, CDCl_3_): δ 169.1, 156.1, 141.2, 135.1 d, *J* = 3.0 Hz), 133.7 (d, *J* = 9.9 Hz), 130.5, 130.5, 130.4, 123.5, 121.6, 118.9, 118.0, 108.3, 56.0, 52.3, 33.5, 31.1, 30.4 (d, *J* = 15.6 Hz), 29.3, 29.1 (d, *J* = 13.3 Hz), 23.0, 22.7, 22.6, 22.5. ^31^P NMR (101 MHz, CDCl_3_): δ 25.17. LCMS *m*/*z* [M]^+^, 539.39.

#### (8-(3,5-Dimethoxyphenyl)­octyl)­triphenylphosphonium Bromide (**7**)

Following general procedure **A**, the desired compound was obtained from **34** (93.2 mg, 0.3 mmol) and triphenylphosphine (74.2 mg, 0.3 mmol). Purification by silica gel column chromatography (dichloromethane/ethanol, 100:0 → 95:5) yielded to **7** as a white sticky solid. Yield 63%. ^1^H NMR (400 MHz, CDCl_3_): δ 7.85–7.70 (m, 9H), 7.68 (td, *J* = 7.7, 3.5 Hz, 6H), 6.29 (d, *J* = 2.3 Hz, 2H), 6.26 (t, *J* = 2.3 Hz, 1H), 3.83–3.67 (m, 2H), 3.76 (s, 6H), 2.49–2.45 (m, 2H), 1.62–1.59 (m, 4H), 1.53–1.47 (m, 2H), 1.25–1.20 (m, 6H). ^13^C NMR (101 MHz, CDCl_3_): δ 160.8, 145.4, 135.1 (d, *J* = 3.0 Hz), 133.9 (d, *J* = 9.9 Hz), 130.6 (d, *J* = 12.5 Hz), 119.1, 118.3, 106.6, 97.7, 55.4, 36.4, 31.3, 30.5, 29.2 (d, *J* = 9.5 Hz), 22.9. ^31^P NMR (101 MHz, CDCl_3_): δ 25.14. LCMS *m*/*z* [M]^+^, 511.09. HRMS (ESI) *m*/*z*: calcd for [M]^+^, 511.2760; observed, 511.2762.

#### (8-(3-Methoxyphenyl)­octyl)­diphenyl­(pyridin-2-yl)­phosphonium Bromide (**8**)

Following general procedure **A**, the desired compound was obtained from **32** (50.0 mg, 0.17 mmol) and diphenyl-2-pyridylphosphine (47.0 mg, 0.17 mmol). Purification by silica gel column chromatography (dichloromethane/ethanol, 100:0 → 95:5) yielded to **8** as a white sticky solid. Yield 45%. ^1^H NMR (400 MHz, CDCl_3_): δ 8.87 (d, *J* = 4.6 Hz, 1H), 8.46 (t, *J* = 6.9 Hz, 1H), 8.15 (q, *J* = 6.9 Hz, 1H), 7.89–7.77 (m, 6H), 7.71–7.64 (m, 5H), 7.17 (dd, *J* = 8.5, 7.2 Hz, 1H), 6.74–6.69 (m, 3H), 3.78 (s, 3H), 3.77–3.69 (m, 2H), 2.54–2.50 (m, 2H), 1.81 (br s, 2H), 1.55–1.49 (m, 4H), 1.26–1.23 (m, 6H). ^13^C NMR (101 MHz, CDCl_3_): δ 159.7, 151.9 (d, *J* = 19.1 Hz), 144.6, 138.9 (d, *J* = 10.3 Hz), 135.1 (d, *J* = 3.0 Hz), 134.1 (d, *J* = 9.7 Hz), 132.5 (d, *J* = 24.4 Hz), 130.5 (d, *J* = 12.4 Hz), 129.3, 128.1 (d, *J* = 3.4 Hz), 121.0, 118.3, 117.5, 114.3, 111.0, 55.3, 36.1, 31.4, 30.6 (d, *J* = 15.4 Hz), 29.3–29.1 (m), 22.7–22.5 (m). ^31^P NMR (101 MHz, CDCl_3_): δ 20.33. LCMS *m*/*z* [M]^+^, 482.4.

#### (8-(3-Methoxy-2-(methoxycarbonyl)­phenyl)­octyl)­diphenyl­(pyridin-2-yl)­phosphonium Bromide (**9**)

Following general procedure **A**, the desired compound was obtained from **33** (32.0 mg, 0.1 mmol) and diphenyl-2-pyridylphosphine (23.4 mg, 0.1 mmol). Purification by silica gel column chromatography (dichloromethane/ethanol, 100:0 → 95:5) yielded to **9** as a white sticky solid. Yield 75%. ^1^H NMR (400 MHz, CDCl_3_): δ 8.88 (dd, *J* = 4.8, 1.6 Hz, 1H), 8.44 (t, *J* = 6.9 Hz, 1H), 8.20–8.10 (m, 1H), 7.92–7.83 (m, 4H), 7.81–7.75 (m, 2H), 7.68 (tdd, *J* = 8.5, 7.1, 4.0 Hz, 5H), 7.29–7.21 (m, 1H), 6.76 (ddd, *J* = 17.0, 8.0, 0.9 Hz, 2H), 3.87 (s, 3H), 3.80 (s, 3H), 3.77–3.65 (m, 2H), (t, *J* = 7.6 Hz, 2H), 1.63–1.59 (m, 2H), 1.49 (q, *J* = 7.4 Hz, 2H), 1.25–1.21 (m, 6H).^13^C NMR (101 MHz, CDCl_3_): δ 169.1, 156.4, 151.9 (d, *J* = 19.1 Hz), 141.3, 138.9 (d, *J* = 0.4 Hz), 135.2 (d, *J* = 3.1 Hz), 134.1 (d, *J* = 9.6 Hz), 132.5 (d, *J* = 24.4 Hz), 130.5 (d, *J* = 12.6 Hz), 128.2 (d, *J* = 3.6 Hz), 121.7, 118.3, 117.5, 108.5, 56.0, 52.3, 33.5, 31.1, 30.5 (d, *J* = 15.3 Hz), 29.4, 29.0 (d, *J* = 2.2 Hz), 22.7–22.4 (m, 2C). ^31^P NMR (101 MHz, CDCl_3_): δ 20.38. LCMS *m*/*z* [M]^+^, 540.39.

#### (8-(3,5-Dimethoxyphenyl)­octyl)­diphenyl­(pyridin-2-yl)­phosphonium Bromide (**10**)

Following general procedure **A**, the desired compound was obtained from **34** (119.0 mg, 0.4 mmol) and diphenyl-2-pyridylphosphine (95.0 mg, 0.4 mmol). Purification by silica gel column chromatography (dichloromethane/ethanol, 100:0 → 95:5) yielded to **10** as a sticky yellowish solid. Yield 70%. ^1^H NMR (400 MHz, CDCl_3_): δ 8.87 (dd, *J* = 4.9, 1.5 Hz, 1H), 8.24 (t, *J* = 6.8 Hz, 1H), 8.12 (tdd, *J* = 7.6, 5.1, 1.6 Hz, 1H), 7.86–7.73 (m, 6H), 7.67 (td, *J* = 7.6, 3.3 Hz, 5H), 3.73 (s, 6H), 3.65–3.54 (m, 2H), 2.49–2.41 (m, 2H), 1.66–1.42 (m, 6H), 1.27–1.14 (m, 6H). ^13^C NMR (101 MHz, CDCl_3_): δ 169.0, 156.7, 152.1 (d, *J* = 19.0 Hz), 141.5, 138.7 (d, *J* = 10.3 Hz), 135.4 (d, *J* = 3.3 Hz), 134.2 (d, *J* = 9.6 Hz), 132.6 (d, *J* = 24.4 Hz), 130.6 (d, *J* = 12.6 Hz), 128.4 (d, *J* = 3.6 Hz), 121.8, 118.3, 117.5, 108.5, 56.1, 33.6, 31.2, 30.7 (d, *J* = 15.3 Hz), 29.4, 29.2 (d, *J* = 2.2 Hz), 22.7–22.4. ^31^P NMR (101 MHz, CDCl_3_): δ 20.28. LCMS *m*/*z* [M]^+^, 512.39.

#### (8-(3-Methoxyphenyl)­octyl)­tri­(naphthalen-2-yl)­phosphonium Bromide (**11**)

Following general procedure **B**, the desired compound was obtained from **32** (109.0 mg, 0.4 mmol) and tris­(naphtalen-1-yl) phosphine (150.1 mg, 0.4 mmol) in dimethylformamide. Purification by silica gel column chromatography (dichloromethane/ethanol, 100:0 → 95:5) yielded to **11** as a white sticky solid. Yield 6%. ^1^H NMR (400 MHz, CDCl_3_): δ 8.43–8.23 (m, 6H), 7.99 (dd, *J* = 19.9, 8.1 Hz, 6H), 7.84–7.67 (m, 3H), 7.63–7.46 (m, 6H), 7.15 (t, *J* = 7.8 Hz, 1H), 6.73–6.62 (m, 3H), 4.63 (br s, 2H), 3.78 (s, 3H), 2.46–2.42 (m, 2H), 1.40 (quint, *J* = 7.0 Hz, 2H), 1.27 (d, *J* = 11.6 Hz, 2H), 1.13–0.94 (m, 6H). ^13^C NMR (101 MHz, CDCl_3_): δ 159.7, 144.7, 138.0 (d, *J* = 10.9 Hz), 136.9 (d, *J* = 3.3 Hz), 134.3 (d, *J* = 9.5 Hz), 132.8 (d, *J* = 8.5 Hz), 130.5, 129.4 (d, *J* = 22.3 Hz), 127.8, 126.0 (d, *J* = 15.4 Hz), 125.4 (d, *J* = 5.5 Hz), 121.0, 114.3, 111.0, 55.3, 36.0, 31.3, 31.1, 30.3 (d, *J* = 15.6 Hz), 29.9, 29.1, 28.9, 28.7. ^31^P NMR (101 MHz, CDCl_3_): δ 28.64. LCMS *m*/*z* [M]^+^, 631.51.

#### (8-(3-Methoxy-2-(methoxycarbonyl)­phenyl)­octyl)­tri­(naphthalen-2-yl)­phosphonium Bromide (**12**)

Following general procedure **B**, the desired compound was obtained from **33** (210.0 mg, 0.6 mmol) and tris­(naphtalen-1-yl) phosphine (242.0 mg, 0.6 mmol) in dimethylformamide. Purification by silica gel column chromatography (dichloromethane/ethanol, 100:0 → 95:5) yielded to **12** as a white sticky solid. Yield 3%. ^1^H NMR (400 MHz, CD_3_OD): δ 8.44 (d, *J* = 8.3 Hz, 3H), 8.20–8.09 (m, 6H), 7.97 (d, *J* = 8.6 Hz, 3H), 7.72 (td, *J* = 7.8, 2.7 Hz, 3H), 7.64 (t, *J* = 7.6 Hz, 3H), 7.50 (t, *J* = 7.8 Hz, 3H), 7.28 (t, *J* = 8.0 Hz, 1H), 6.82 (dd, *J* = 36.1, 8.0 Hz, 2H), 4.03–3.93 (m, 2H), 3.79 (s, 3H), 3.77 (s, 3H), 2.41 (t, *J* = 7.9 Hz), 1.50–1.34 (m, 6H), 1.16–0.98 (m, 6H). ^13^C NMR (101 MHz, CD_3_OD): δ 169.0, 156.1, 138.2, 135.9, 134.1, 131.6 (d, *J* = 9.3 Hz), 130.1, 128.9, 126.7 (d, *J* = 14.6 Hz), 126.2, 122.5, 109.7, 56.4, 52.5, 34.2, 32.1, 31.0, 30.3, 29.9, 29.2. ^31^P NMR (101 MHz, CDCl_3_): δ 28.60. LCMS *m*/*z* [M]^+^, 689.41.

#### 8-(3,5-Dimethoxyphenyl)­octyl)­tri­(naphthalen-2-yl)­phosphonium Bromide (**13**)

Following general procedure **B**, the desired compound was obtained from **34** (254.0 mg, 0.8 mmol) and tris­(naphtalen-1-yl) phosphine (318.0 mg, 0.8 mmol) in dimethylformamide. Purification by silica gel column chromatography (dichloromethane/ethanol, 100:0 → 95:5) yielded to **13** as a white sticky solid. Yield 2%. ^1^H NMR (400 MHz, CD_3_OD): δ 8.44 (d, *J* = 8.3 Hz, 3H), 8.24–8.07 (m, 6H), 7.97 (d, *J* = 8.6 Hz, 3H), 7.78–7.59 (m, 6H), 7.50 (t, *J* = 7.8 Hz, 3H), 6.46–6.33 (m, 1H), 6.27 (s, 2H), 4.05–3.93 (m, 2H), 3.72 (s, 6H), 2.42 (t, *J* = 7.6 Hz, 2H), 1.56–1.36 (m, 6H), 1.22–1.02 (m, 6H). ^13^C NMR (101 MHz, CD_3_OD): δ 162.2, 138.7 (d, *J* = 11.0 Hz, 3C), 138.2 (d, *J* = 3.3 Hz, 3C), 135.9 (d, *J* = 9.5 Hz), 134.2 (d, *J* = 8.5 Hz), 131.7 (3C), 130.1 (3C), 128.9 (3C), 126.7 (d, *J* = 14.5 Hz, 3C), 126.2 (d, *J* = 5.5 Hz, 3C), 107.5, 98.4, 55.6, 37.0, 32.2, 31.1 (d, *J* = 16.4 Hz), 29.9–29.2 (m, 3C), 25.7 (2C). ^31^P NMR (101 MHz, CDCl_3_): δ 28.74. LCMS *m*/*z* [M]^+^, 661.40.

#### 1-(8-(3-Methoxyphenyl)­octyl)­pyridin-1-ium Bromide (**14**)

Following general procedure **C**, the desired compound was obtained from **32** (50.9 mg, 0.2 mmol) and pyridine (40.4 mg, 0.04 mL, 0.8 mmol). Purification by trituration from diethyl ether, gave **14** as yellowish oil. Yield 65%. ^1^H NMR (400 MHz, CD_3_OD): δ 9.00 (d, *J* = 6.0 Hz, 2H), 8.60 (t, *J* = 7.8 Hz, 1H), 8.12 (t, *J* = 7.0 Hz, 2H), 7.19–7.10 (m, 1H), 6.76–6.67 (m, 3H), 4.67–4.59 (m, 2H), 3.76 (s, 3H), 2.61–2.52 (m, 2H), 2.03 (d, *J* = 7.9 Hz, 2H), 1.60 (quint, *J* = 7.4 Hz, 2H), 1.37 (m, *J* = 12.0 Hz, 8H). ^13^C NMR (101 MHz, CD_3_OD): δ 161.2, 146.9, 145.9, 145.5, 130.2, 129.5, 121.8, 115.2, 111.9, 63.1, 55.5, 36.9, 32.5 (d, *J* = 3.1 Hz), 30.3, 30.1, 30.1, 27.1. LC–MS *m*/*z* [M]^+^, 298.33.

#### 1-(8-(3-Methoxy-2-(methoxycarbonyl)­phenyl)­octyl)­pyridin-1-ium Bromide (**15**)

Following general procedure **C**, the desired compound was obtained from **33** (41.4 mg, 0.1 mmol) and pyridine (55.1 mg, 0.06 mL, 0.4 mmol). Purification by trituration from diethyl ether, gave **15** as yellow oil. Yield 67%. ^1^H NMR (400 MHz, CD_3_OD): δ 9.04–8.98 (m, 2H), 8.60 (tt, *J* = 7.8, 1.4 Hz, 1H), 8.12 (t, *J* = 7.0 Hz, 2H), 7.30 (t, *J* = 8.0 Hz, 1H), 6.91–6.80 (m, 2H), 4.68–4.59 (m, 2H), 3.84 (s, 3H), 3.80 (s, 3H), 2.56–2.47 (m, 2H), 2.02 (t, *J* = 7.4 Hz, 2H), 1.56 (quint, *J* = 7.3 Hz, 2H), 1.44–1.26 (m, 8H). ^13^C NMR (101 MHz, CD_3_OD): δ 170.8, 157.8, 146.9, 142.1, 131.6, 129.5, 122.5, 109.8, 63.1, 56.4, 52.6, 34.3, 32.4, 32.2, 30.1 (d, *J* = 6.3 Hz), 29.9, 27.0. LCMS *m*/*z* [M]^+^, 356.35.

#### 1-(8-(3,5-Dimethoxyphenyl)­octyl)­pyridin-1-ium Bromide (**16**)

Following general procedure **C**, the desired compound was obtained from **34** (40.5 mg, 0.1 mmol) and pyridine (38.6 mg, 0.04 mL, 0.4 mmol). Purification by trituration from diethyl ether, gave **16** as yellowish oil. Yield 63%. ^1^H NMR (400 MHz, CD_3_OD): δ 9.06–9.00 (m, 2H), 8.60 (tt, *J* = 7.8, 1.3 Hz, 1H), 8.12 (t, *J* = 7.0 Hz, 2H), 6.30 (dd, *J* = 16.0, 2.3 Hz, 3H), 4.69–4.60 (m, 2H), 3.74 (s, 6H), 2.52 (t, *J* = 7.6 Hz, 2H), 2.02 (t, *J* = 7 0.0 Hz, 2H), 1.59 (quint, *J* = 7.2 Hz, 2H), 1.36 (m, *J* = 13.0 Hz, 8H). ^13^C NMR (101 MHz, CD_3_OD): δ 162.2, 146.8, 145.9, 129.5, 107.5, 98.5, 63.1, 55.6, 37.1, 32.4 (d, *J* = 9.9 Hz), 30.3, 30.0 (d, *J* = 9.9 Hz), 27.1. LCMS *m*/*z* [M]^+^, 328.34.

#### 1-(8-(3-Methoxyphenyl)­octyl)-4-methylpyridin-1-ium Bromide (**17**)

Following general procedure **C**, the desired compound was obtained from **32** (54.4 mg, 0.2 mmol) and 4-methylpyridine (50.8 mg, 0.05 mL, 0.8 mmol). Purification by trituration from diethyl ether, gave **17** as orange oil. Yield 65%. ^1^H NMR (400 MHz, CD_3_OD): δ 8.85–8.76 (m, 2H), 7.92 (d, *J* = 6.2 Hz, 2H), 7.19–7.10 (m, 1H), 6.72 (tdd, *J* = 6.7, 3.0, 1.9 Hz, 3H), 4.59–4.51 (m, 2H), 3.76 (s, 3H), 2.68 (s, 3H), 2.60–2.52 (m, 2H), 1.99 (q, *J* = 7.3 Hz, 2H), 1.60 (quint, *J* = 7.3 Hz, 2H), 1.35 (m, *J* = 7.3 Hz, 8H). ^13^C NMR (101 MHz, CD_3_OD): δ 161.2 (d, *J* = 8.8 Hz), 145.5, 144.8, 130.2, 129.9, 121.8, 115.2, 111.9, 62.2, 55.5, 36.9, 32.3, 30.3, 30.1, 30.1, 27.2, 22.0. LCMS *m*/*z* [M]^+^, 312.23.

#### 1-(8-(3-methoxy-2-(methoxycarbonyl)­phenyl)­octyl)-4-methylpyridin-1-ium Bromide (**18**)

Following general procedure **C**, the desired compound was obtained from **33** (52.9 mg, 0.2 mmol) and 4-methylpyridine (27.6 mg, 0.03 mL, 0.8 mmol). Purification by trituration from diethyl ether, gave **18** as light orange oil. Yield 22%. ^1^H NMR (400 MHz, CD_3_OD): δ 8.80 (dd, *J* = 6.8, 2.9 Hz, 2H), 7.94–7.88 (m, 2H), 7.30 (td, *J* = 8.1, 3.0 Hz, 1H), 6.90–6.80 (m, 2H), 4.58–4.50 (m, 2H), 3.84 (s, 3H), 3.80 (s, 3H), 2.68 (s, 3H), 2.55–2.47 (m, 2H), 2.03–1.93 (m, 2H), 1.61–1.49 (m, 2H), 1.41–1.26 (m, 8H). ^13^C NMR (101 MHz, CD_3_OD): δ 170.8, 157.8, 144.8 (2C), 143.9, 142.1, 131.6, 130.1 (2C), 122.5, 111.9, 110.8, 109.7, 62.2, 56.4, 52.6, 36.9, 34.3, 32.2 (d, *J* = 10.1 Hz, 2C), 30.1 (d, *J* = 5.6 Hz), 29.9, 27.1 (d, *J* = 5.2 Hz), 21.9. LCMS *m*/*z* [M]^+^, 370.35.

#### 1-(8-(3,5-Dimethoxyphenyl)­octyl)-4-methylpyridin-1-ium Bromide (**19**)

Following general procedure **C**, the desired compound was obtained from **34** (40.3 mg, 0.1 mmol) and 4-methylpyridine (34.1 mg, 0.04 mL, 0.4 mmol). Purification by trituration from diethyl ether, gave **19** as orange oil. Yield 92%. ^1^H NMR (400 MHz, CD_3_OD): δ 8.83–8.77 (m, 2H), 7.93 (d, *J* = 6.3 Hz, 2H), 6.32 (d, *J* = 2.2 Hz, 2H), 6.27 (t, *J* = 2.3 Hz, 1H), 4.58–4.53 (m, 2H), 3.74 (s, 6H), 2.68 (s, 3H), 2.51 (d, *J* = 7.7 Hz, 2H), 1.99 (q, *J* = 7.3 Hz, 2H), 1.59 (quint, *J* = 7.2 Hz, 2H), 1.41–1.27 (m, 8H). ^13^C NMR (101 MHz, CD_3_OD): δ 162.2, 161.2, 146.3, 144.8, 129.9, 107.5, 98.5, 62.2, 55.6, 37.1, 32.4, 32.3, 30.3, 30.1, 30.0, 27.1, 22.0. LCMS *m*/*z* [M]^+^, 342.34.

#### 3-(8-(3-Methoxyphenyl)­octyl)-1-methyl-1*H*-imidazol-3-ium Bromide (**20**)

Following general procedure **C**, the desired compound was obtained from **32** (57.2 mg, 0.2 mmol) and 1-methylimidazole (46.8 mg, 0.05 mL, 0.8 mmol). Purification by trituration from diethyl ether, gave **20** as yellowish oil. Yield 46%. ^1^H NMR (400 MHz, CD_3_OD): δ 8.96 (s, 1H), 7.64 (s, 1H), 7.58 (s, 1H), 7.15 (t, *J* = 8.2 Hz, 1H), 6.77–6.66 (m, 3H), 4.20 (t, *J* = 7.3 Hz, 2H), 3.93 (s, 3H), 3.76 (s, 3H), 2.57 (t, *J* = 7.7 Hz, 2H), 1.88 (quint, *J* = 7.1 Hz, 2H), 1.61 (quint, *J* = 7.2 Hz, 2H), 1.43–1.25 (m, 8H). ^13^C NMR (101 MHz, CD_3_OD): δ 161.2, 145.5, 137.8, 130.2, 125.0, 123.7, 121.8, 115.2, 111.9, 55.5, 50.8, 36.9, 36.5, 32.5, 31.1, 30.3, 30.1, 30.0, 27.2. LCMS *m*/*z* [M]^+^, 301.33.

#### 3-(8-(3-Methoxy-2-(methoxycarbonyl)­phenyl)­octyl)-1-methyl-1*H*-imidazol-3-ium Bromide (**21**)

Following general procedure **C**, the desired compound was obtained from **33** (53.6 mg, 0.2 mmol) and 1-methylimidazole (36.9 mg, 0.04 mL, 0.8 mmol). Purification by trituration from diethyl ether, gave **21** as yellow oil. Yield 94%. ^1^H NMR (400 MHz, CD_3_OD): δ 8.93 (s, 1H), 7.64 (d, *J* = 2.0 Hz, 1H), 7.57 (d, *J* = 2.0 Hz, 1H), 7.30 (dd, *J* = 8.3, 7.7 Hz, 1H), 6.85 (ddd, *J* = 16.6, 8.0, 0.8 Hz, 2H), 4.21 (t, *J* = 7.3 Hz, 2H), 3.93 (s, 3H), 3.85 (s, 3H), 3.79 (s, 3H), 2.56–2.46 (m, 2H), 1.88 (quint, *J* = 7.3 Hz, 2H), 1.60–1.51 (m, 2H), 1.33 (quint, *J* = 4.5 Hz, 8H). ^13^C NMR (101 MHz, CD_3_OD): δ 199.0, 185.9, 170.3, 159.7, 157.2, 153.1, 153.0, 151.8, 150.7, 150.0, 137.9, 84.5, 80.7, 79.0, 64.6, 62.4, 60.4, 59.2, 58.4, 58.3, 58.0, 55.3. LCMS *m*/*z* [M]^+^, 359.35.

#### 3-(8-(3,5-Dimethoxyphenyl)­octyl)-1-methyl-1*H*-imidazol-3-ium Bromide (**22**)

Following general procedure **C**, the desired compound was obtained from **34** (42.3 mg, 0.1 mmol) and 1-methylimidazole (31.5 mg, 0.03 mL, 0.4 mmol). Purification by trituration from diethyl ether, gave **22** as yellowish oil. Yield 63%. ^1^H NMR (400 MHz, CD_3_OD): δ 8.96 (s, 1H), 7.63 (d, *J* = 2.3 Hz, 1H), 7.57 (d, *J* = 2.2 Hz, 1H), 6.34–6.26 (m, 3H), 4.20 (t, *J* = 7.3 Hz, 2H), 3.93 (s, 3H), 3.74 (s, 6H), 2.53 (t, *J* = 7.6 Hz, 2H), 1.88 (quint, *J* = 7.1 Hz, 2H), 1.60 (quint, *J* = 7.1 Hz, 2H), 1.35 (s, 8H). ^13^C NMR (101 MHz, CD_3_OD): δ 162.2, 146.3, 124.9, 123.7, 107.5, 98.5, 55.6, 54.8, 50.8, 37.1, 36.5, 32.4, 31.1, 30.3, 30.1, 30.0, 27.2. LCMS *m*/*z* [M]^+^, 331.24.

#### 4-Amino-1-(8-(3-Methoxyphenyl)­octyl)­pyridin-1-ium Bromide (**23**)

Following general procedure **D**, the desired compound was obtained from **32** (37.3 mg, 0.1 mmol) and 4-aminopyridine (11.8 mg, 0.1 mmol) in acetonitrile. Purification by trituration from diethyl ether, gave **23** as white solid. Yield 67%. ^1^H NMR (400 MHz, CD_3_OD): δ 8.11–8.06 (m, 2H), 7.18–7.11 (m, 1H), 6.87–6.82 (m, 2H), 6.75–6.68 (m, 3H), 4.12 (t, *J* = 7.4 Hz, 2H), 3.76 (s, 3H), 2.61–2.52 (m, 2H), 1.83 (quint, *J* = 7.4 Hz, 2H), 1.66–1.53 (m, 2H), 1.39–1.25 (m, 8H). ^13^C NMR (101 MHz, CD_3_OD): δ 161.2, 160.7, 145.5, 143.9, 130.2, 121.8, 115.2, 111.9, 110.8, 59.2, 55.5, 36.9, 32.5, 31.9, 30.3, 30.1, 30.0, 27.1. LCMS *m*/*z* [M]^+^, 313.23.

#### 4-Amino-1-(8-(3-Methoxy-2-(Methoxycarbonyl)­phenyl)­octyl)­pyridin-1-ium Bromide (**24**)

Following general procedure **D**, the desired compound was obtained from **33** (46.1 mg, 0.1 mmol) and 4-aminopyridine (11.8 mg, 0.1 mmol) in acetonitrile. Purification by trituration from diethyl ether, gave **24** as yellowish oil. Yield 22%. ^1^H NMR (400 MHz, CD_3_OD): δ 8.11–8.06 (m, 2H), 7.30 (dd, *J* = 8.3, 7.7 Hz, 1H), 6.89–6.81 (m, 4H), 4.12 (t, *J* = 7.3 Hz, 2H), 3.84 (s, 3H), 3.80 (s, 3H), 2.55–2.47 (m, 2H), 1.84 (quint, *J* = 7.2 Hz, 2H), 1.56 (quint, *J* = 7.5 Hz, 2H), 1.32 (t, *J* = 5.2 Hz, 8H). ^13^C NMR (101 MHz, CD_3_OD): δ 170.8, 160.7, 157.8, 143.9, 142.1, 131.6, 124.8, 122.5, 110.8, 109.7, 59.2, 56.3, 52.6, 34.3, 32.2, 31.9, 30.2, 30.2, 29.9, 27.0. LCMS *m*/*z* [M]^+^, 371.35.

#### 4-Amino-1-(8-(3,5-dimethoxyphenyl)­octyl)­pyridin-1-ium Bromide (**25**)

Following general procedure **D**, the desired compound was obtained from **34** (34.1 mg, 0.1 mmol) and 4-aminopyridine (11.8 mg, 0.1 mmol) in acetonitrile. Purification by trituration from diethyl ether, gave **25** as yellow oil. Yield 77%. ^1^H NMR (400 MHz, CD_3_OD): δ 8.09 (d, *J* = 6.9 Hz, 2H), 6.86–6.79 (m, 2H), 6.34–6.26 (m, 3H), 4.12 (t, *J* = 7.4 Hz, 2H), 3.74 (s, 6H), 2.53 (t, *J* = 7.6 Hz, 2H), 1.83 (quint, *J* = 7.2 Hz, 2H), 1.60 (quint, *J* = 7.1 Hz, 2H), 1.41–1.26 (m, 8H). ^13^C NMR (101 MHz, CD_3_OD): δ 162.2, 160.7, 146.3, 143.9, 110.8, 107.5, 98.5, 59.2, 55.6, 37.1, 32.3, 31.9, 30.3, 30.0, 27.1. LCMS *m*/*z* [M]^+^, 343.34.

#### 8-(3-Methoxyphenyl)­octyl Methanesulfonate (**26**)

Following general procedure **H**, the desired compound **26** was obtained starting from **29** (384.6 mg, 1.6 mmol), triethylamine (0.3 mL, 2.1 mmol) and a catalytic amount of 4-dimethylaminopyridine were solubilized in 2-methyltetrahydrofuran (3.25 mL). Then, methanesulfonyl chloride (0.2 mL, 2.1 mmol) was added dropwise to the mixture and stirred at room temperature for 3 h. The residue was purified by silica gel flash column chromatography (petroleum ether/toluene/ethyl acetate, 75:5:20) yielding the corresponding mesylate derivative **26** as a transparent oil. Yield 66%. ^1^H NMR (400 MHz, CDCl_3_): δ 7.25–7.15 (m, 1H), 6.80–6.69 (m, 3H), 3.80 (s, 3H), 3.63 (t, *J* = 6.6 Hz, 2H), 2.62–2.53 (t, *J* = 5.6, 2H), 1.65–1.50 (m, 4H), 1.40–1.30 (m, 8H). LCMS *m*/*z* [M + H]^+^, 315.33.

#### Methyl 2-Methoxy-6-(8-((methylsulfonyl)­oxy)­octyl)­benzoate (**27**)

Following general procedure **H**, the desired compound **27** was obtained starting from **30** (158.0 mg, 0.5 mmol), triethylamine (0.1 mL, 0.7 mmol) and a catalytic amount of 4-dimethylaminopyridine were solubilized in 2-methyltetrahydrofuran (1.62 mL). Then, methanesulfonyl chloride (0.1 mL, 0.7 mmol) was added dropwise to the mixture and stirred at room temperature for 3 h. The residue was purified by silica gel flash column chromatography (petroleum ether/toluene/ethyl acetate, 75:5:20) yielding the corresponding mesylate derivative **27** as a transparent oil. Yield 85%. ^1^H NMR (400 MHz, CDCl_3_): δ 7.26 (t, 1H), 6.78 (dd, *J* = 20.8, 8.0 Hz, 2H), 4.21 (t, *J* = 6.6 Hz, 2H), 3.90 (s, 3H), 3.81 (s, 3H), 2.99 (s, 3H), 2.53 (t, 2H), 1.73 (quint, *J* = 6.6 Hz, 2H), 1.61–1.51 (m, 2H), 1.42–1.23 (m, 8H). LCMS *m*/*z* [M-CH_3_]^−^, 341.24.

#### 8-(3,5-Dimethoxyphenyl)­octyl Methanesulfonate (**28**)

Following general procedure **H**, the desired compound **28** was obtained starting from **31** (167.0 mg, 0.5 mmol), triethylamine (0.1 mL, 0.7 mmol) and a catalytic amount of 4-dimethylaminopyridine were solubilized in 2-methyltetrahydrofuran (1.62 mL). Then, methanesulfonyl chloride (0.1 mL, 0.7 mmol) was added dropwise to the mixture and stirred at room temperature for 3 h. The residue was purified by silica gel flash column chromatography (petroleum ether/toluene/ethyl acetate, 75:5:20) yielding the corresponding mesylate derivative **28** as a transparent oil. Yield 80%. ^1^H NMR (400 MHz, CDCl_3_): δ 6.39–6.24 (m, 3H), 4.21 (t, *J* = 6.6 Hz, 2H), 3.78 (s, 6H), 3.00 (s, 3H), 2.54 (t, *J* = 7.8 Hz, 2H), 1.74 (quint, *J* = 6.9 Hz, 2H), 1.64–1.55 (m, 2H), 1.45–1.25 (m, 8H). LCMS *m*/*z* [M + H]^+^, 345.24.

#### 8-(3-Methoxyphenyl)­octan-1-ol (**29**)

Following general procedure **G**, the desired compound was purified by silica gel column chromatography (petroleum ether/toluene/ethyl acetate, 75:5:20) yielding **29** as a yellowish oil. Yield 60%. ^1^H NMR (400 MHz, CDCl_3_): δ 7.22–7.16 (m, 1H), 6.77 (dt, *J* = 7.5, 1.2 Hz, 1H), 6.74–6.70 (m, 2H), 3.80 (s, 3H), 3.63 (t, *J* = 6.6 Hz, 2H), 2.61–2.54 (m, 2H), 1.66–1.51 (m, 2H), 1.44 (s, 2H), 1.37–1.30 (m, 8H). LCMS *m*/*z* [M + H]^+^, 237.31.

#### Methyl 2-(8-Hydroxyoctyl)-6-methoxybenzoate (**30**)

Following general procedure **G**, the desired compound was purified by silica gel column chromatography (petroleum ether/toluene/ethyl acetate, 70:10:20) yielding **30** as a yellowish oil. Yield 70%. ^1^H NMR (400 MHz, CDCl_3_): δ 7.29–7.23 (m, 2H), 6.81 (dd, *J* = 7.8, 0.9 Hz, 1H), 6.76 (dd, *J* = 8.3, 0.9 Hz, 1H), 3.90 (s, 3H), 3.81 (s, 3H), 3.63 (t, *J* = 6.6 Hz, 2H), 2.56–2.51 (m, 2H), 1.62–1.50 (m, 2H), 1.50–1.42 (m, 2H), 1.39–1.27 (m, 8H). LCMS *m*/*z* [M + H]^+^, 295.23.

#### 8-(3,5-Dimethoxyphenyl)­octan-1-ol (**31**)

Following general procedure **G**, the desired compound was purified by silica gel column chromatography (petroleum ether/toluene/ethyl acetate, 75:5:20) yielding **31** as a yellowish oil. Yield 64%. ^1^H NMR (400 MHz, CDCl_3_): δ 6.39–6.24 (m, 3H), 4.21 (t, *J* = 6.6 Hz, 2H), 3.78 (s, 6H), 3.00 (s, 3H), 2.54 (t, *J* = 7.8 Hz, 2H), 1.74 (quint, *J* = 6.9 Hz, 2H), 1.64–1.55 (m, 2H), 1.45–1.25 (m, 8H). LCMS *m*/*z* [M + H]^+^, 345.24.

#### 1-(8-Bromooctyl)-3-methoxybenzene (**32**)

Following general procedure **I**, the desired compound **32** was obtained starting from **26**. The compound (1.0 g, 3.2 mmol) was dissolved in acetone (8.1 mL), then lithium bromide (1.4 g, 16.2 mmol) was added. The residue was purified by silica gel flash column chromatography (petroleum ether/dichloromethane/ethyl acetate, 85:10:5) yielding the corresponding bromine derivative **32** as a transparent oil. Yield 78%. ^1^H NMR (400 MHz, CDCl_3_): δ 7.22–7.16 (m, 1H), 6.77 (d, *J* = 1.2 Hz, 1H), 6.75–6.70 (m, 2H), 3.80 (s, 3H), 3.40 (t, *J* = 6.9 Hz, 2H), 2.58 (t, *J* = 6.7 Hz, 2H), 1.85 (quint, *J* = 6.9 Hz, 2H), 1.65–1.54 (m, 2H), 1.47–1.23 (m, 8H). LCMS *m*/*z* [M + K]^+^, 338.54.

#### Methyl 2-(8-Bromooctyl)-6-methoxybenzoate (**33**)

Following general procedure **I**, the desired compound **33** was obtained starting from **27**. The compound (59.0 mg, 0.2 mmol) was dissolved in acetone (0.5 mL), then lithium bromide (68.6 mg, 0.8 mmol) was added. The residue was purified by silica gel flash column chromatography (petroleum ether/dichloromethane/ethyl acetate, 85:10:5) yielding the corresponding bromine derivative **33** as a yellowish oil. Yield 71%. ^1^H NMR (400 MHz, CDCl_3_): δ 7.26 (t, *J* = 8.0 Hz, 1H), 6.81 (dd, *J* = 7.8, 0.9 Hz, 1H), 6.75 (dd, *J* = 8.3, 0.8 Hz, 1H), 3.90 (s, 3H), 3.81 (s, 3H), 3.39 (t, *J* = 6.8 Hz, 2H), 2.58–2.49 (m, 2H), 1.84 (quint, *J* = 7.0 Hz, 2H), 1.62–1.52 (m, 2H), 1.46–1.36 (m, 2H), 1.35–1.24 (m, 6H). LCMS *m*/*z* [M]^+^, 357.25.

#### 1-(8-Bromooctyl)-3,5-dimethoxybenzene (**34**)

Following general procedure **I**, the desired compound **34** was obtained starting from **28**. The compound (233.0 mg, 0.7 mmol) was dissolved in acetone (1.7 mL), then lithium bromide (176.2 mg, 2.0 mmol) was added. The residue was purified by silica gel flash column chromatography (petroleum ether/dichloromethane/ethyl acetate, 85:10:5) yielding the corresponding bromine derivative **34** as a transparent oil. Yield 95%. ^1^H NMR (400 MHz, CDCl_3_): δ 6.34 (d, *J* = 2.3 Hz, 2H), 6.30 (t, *J* = 2.3 Hz, 1H), 3.78 (s, 6H), 3.40 (t, *J* = 6.9 Hz, 2H), 2.57–2.51 (m, 2H), 1.91–1.78 (m, 2H), 1.64–1.54 (m, 3H), 1.42 (t, *J* = 7.4 Hz, 2H), 1.37–1.27 (m, 6H). LCMS *m*/*z* [M + H]^+^, 331.14.

### Biology

#### Evaluation of the Cytotoxicity Profile of Compounds **5–25**

Toxicity of drugs to mammalian cells was carried out according to a method previously described,^67^ with slight modifications. Phenylarsine oxide (PAO) was used as positive control. All CC_50_ values were determined on at least three independent occasions, they were calculated by nonlinear regression using an equation for a sigmoidal dose–response curve with variable slope using Prism 8.0 (GraphPad Software Inc., San Diego, CA, USA) and are presented as average ±SEM. The selectivity index (SI) was calculated as CC_50_ (HFF)/EC_50_ (*Trypanosoma*).

#### Evaluation of Compounds **5–25** against *T. b. brucei* Wild-Type and Resistant Strains

##### Parasite Strain and Cultures

Several different strains of *T. b. brucei* (BSF) were used in this study: (1) wild type *T. b. brucei*, strain Lister 427 (s427; MiTat 1.2/BS221); (2) A multidrug-resistant strain, B48 which was created from s427-WT after deletion of both the TbAT1/P2 drug transporter and *in vitro* adaptation to pentamidine, leading to the functional loss of the high affinity pentamidine transporter (HAPT1);^68^ (3) BSF of *T. b. brucei* rendered resistant to suramin by *in vitro* exposure; (4) clonal, Tb427WT-derived dys-kinetoplastid cell line, adapted to *in vitro* growth in 1 μM ISM by stepwise increases in the medium concentration of the drug. All the trypanosomes were used only as bloodstream trypomastigotes and cultured in the standard Hirumi’s Modified Iscove’s medium #9 (HMI-9), supplemented with 10% heat-inactivated fetal bovine serum (FBS), 14 μL/L β-mercaptoethanol, and 3.0 g sodium hydrogen carbonate per liter of medium (pH 7.4). Trypanosomes were cultured in vented flasks at 37 °C in a 5% CO_2_ atmosphere.^67^

##### Resazurin-Based Drug Sensitivity Assay

The susceptibilities of trypanosomes to compounds **5–25** were determined as previously described using the resazurin (Alamar Blue) assay.^69^ Briefly, this protocol involves preparing a 96-well cell culture plate with a 100 μL serially diluted test compound (200 μM top concentration) in HMI-9 + 10% FBS (24 wells per control drug or test compound, with the 24th well serving as drug-free). This is followed by adjusting the cell density of mid log-phase trypanosome cultures to the required concentration of 2 × 10^5^ cells/mL (2 × 10^4^ cells/well). Then, 100 μL of the adjusted cell density/culture was added to all 24 wells in the plate, then incubating trypanosomes and test compound for 48 h followed by the addition of 20 μL filter-sterilized resazurin solution prepared by adding 25 mg resazurin sodium salt to 200 mL filter-9 sterilized PBS. This was followed by a further 24 h of incubation. All incubations were carried out at 37 °C with 5% CO_2_. Standard drugs were used as positive control, where appropriate, including PMD, SUR and ISM. Fluorescence was measured in the 96-well plates with a FLUOstar Optima (BMG Labtech, Durham, NC, USA) at wavelengths of 544 nm for excitation, 590 for emission, and a gain of 1250. EC_50_ values were calculated using GraphPad Prism 9.0 software via nonlinear regression with an equation for a sigmoidal dose–response curve with variable slope (GraphPad Software Inc., San Diego, CA, USA) and are presented as average ±SEM.

#### Evaluation of Compounds **5–13** against *T. congolense*, *T. b. equiperdum* and *T. b. evansi*

##### Parasite Strain and Cell Cultures

Other Trypanosoma species used in this study include *T. b. evansi* AntTat 3/3 (Wild-type);^70^ and *T. b. equiperdum* BoTat 1 (bordeaux trypanozoon antigenic type 1) P (wild-type).^71^ All the trypanosomes were used only as bloodstream trypomastigotes and cultured in the standard Hirumi’s Modified Iscove’s medium #9 (HMI-9), supplemented with 10% heat-inactivated fetal bovine serum (FBS), 14 μL/L β-mercaptoethanol, and 3.0 g sodium hydrogen carbonate per liter of medium (pH 7.4). Trypanosomes were cultured in vented flasks at 37 °C in a 5% CO_2_ atmosphere.^67^ Bloodstream forms (BSF) of the *T. congolense* savannah-type strain IL3000 and the diminazene aceturate (DA) and PMD-resistant clone 4C2 were cultured as described by Coustou et al.^72^

##### Resazurin-Based Drug Sensitivity Assay

Trypanosomes and test drugs were incubated for a period of 48 h followed by the addition of Alamar Blue solution and by a further 24 h incubation. Two trypanocides were used as positive controls: PMD and DA. All EC_50_ values were determined on at least three independent occasions, they were calculated by nonlinear regression using an equation for a sigmoidal dose–response curve with variable slope using Prism 8.0 (GraphPad Software Inc., San Diego, CA, USA) and are presented as average ±SEM.

#### Evaluation of Compounds **5–25** against *T. cruzi*

##### Compounds

A stock solution of the compounds at a concentration of 10 mM was prepared in DMSO. Benznidazole (LAFEPE, Brazil) stock solutions in DMSO was prepared at 50 mM. Benznidazole was obtained from the Fiocruz-RJ pharmacy.

##### Trypanosoma cruzi

The trypomastigote forms of Tulahuen strain (DTU VI) expressing the *E. coli* β-galactosidase gene were collected from the supernatant of L929 cell cultures previously infected (host/parasite cell ratio 10:1).^52^ Purified parasites were added to RPMI 1640 medium supplemented with 5% fetal bovine serum (FBS) to perform assays at 37 °C in 5% CO_2_.^52^ Trypomastigotes of the Y strain of *T. cruzi* (Discrete Typing Unit (DTU) II) were acquired by cardiac puncture of Swiss Webster mice at the peak of parasitemia.^73^

##### Mammalian Cell Cultures

L929 cell lines were routinely maintained through weekly dissociation with 0.01% trypsin solution followed by plating with 4 × 10^3^ cells per well in 96-wells microplates and sustained at 37 °C in RPMI 1640 medium (Sigma-Aldrich).^52^

##### Cytotoxicity Assay

L929 culture cells were seeded in 96 well plates with 4 × 103 cells per well and sustained in RPMI medium supplemented with 10% FBS. The compounds were added (0–200 μM, serially diluted 1:2) and the noninfected cultures incubated for 96 h at 37 °C/5% CO_2_. Then, their viability was assessed by Alamar Blue reagent following the manufacturer’s specifications. Controls were carried out with cultures kept under the same conditions in the absence of the compounds. The data was expressed by the LC_50_ value which represents the concentration capable of inducing a 50% loss of cellular viability.^52^

##### *In Vitro* Activity against Intracellular Forms of *T. cruzi*

(Tulahuen strain transfected with β-galactosidase gene, DTU VI): L929 cells were infected with trypomastigotes obtained from the supernatant of infected L929 cultures. After 2 h of interaction (10 parasites per host cell), the parasites which were not internalized were removed by replacing the RPMI medium. After 48 h of incubation, the compounds were added to the infected cultures (first step at 10 μM, and those compounds that presented >50% activity were next investigated by a dose–response assay, under drug concentrations serially diluted 1:2 up to 10 μM). In both conditions, the cultures incubated for 96 h at 37 °C/5% CO_2_. Benznidazole and DMSO (solvent used for the compounds) were run in parallel as positive and negative controls, respectively. After the elapsed time, 50 μL/well of CPRG (chlorophenol red-β-d-galactopyranoside) was added and a reading was done in a spectrophotometer at 570 nm.^52^ The activity of the compounds was expressed by the EC_50_, which represents the concentration capable of inducing a 50% loss of viability in the parasites.^52^

##### *In Vitro* Activity on Bloodstream Trypomastigotes of *T. cruzi*

(BT, Y strain, DTU II): Swiss male mice were inoculated with 5 × 10^4^ bloodstream trypomastigotes and at the parasitaemia peak (eighth dpi), euthanized using 3% isoflurane and blood samples obtained by cardiac puncture (CEUA FIOCRUZ L038–2017). Then, 100 μL of parasite suspension (10^7^/mL in RPMI medium +5% FBS) was added to the same volume of RPMI +5% FBS containing treatment with the studied compounds (serially diluted −1:2) at twice the desired final concentration. After 24 h at 37 °C, the number of live parasites was determined by light microscopy quantification using a Neubauer chamber. Untreated controls were carried out with parasites kept under the same conditions and the reference drug (benznidazole) was run in parallel. The activity of the compounds was expressed by the EC_50_ values. The assays were done at least twice, in duplicate.

##### Data Analysis and EC_50_ and LC_50_ Calculation

EC_50_, and LC_50_ calculation, as well as the 95% confidence interval presented in lieu of standard deviation, were performed by Prism Graphpad Version 9.1.0 using nonlinear regression with the data obtained in at least two assays in triplicate.

#### Evaluation of Compounds **5**–**13** Against *T. vivax* and *T. congolense*

##### Animals

Female Swiss mice were purchased from Janvier (France). Food for laboratory rodents (Carfil, Arendonk, Belgium) and drinking water were available ad libitum. Animals were kept in quarantine for at least 5 days prior to infection and were randomly allocated to the experimental units.

##### *Ex vivo* Activity against *T. vivax* and *T. congolense*

*T. vivax* (ILRAD700), kindly provided by the Vrije Universiteit Brussel (VUB), and *T. congolense* (MSORO M7H and ISM resistant), a kind gift from Prof. Jan Van Den Abbeele, were used for *ex vivo* assays. The *ex vivo* experiments were performed as previously described.^74^ Briefly, donor mice were intraperitoneally inoculated with 10^4^ trypanosomes. Approximately 5–6 days post infection, blood was harvested via cardiac puncture, and parasites were subsequently isolated using the mini-anion exchange centrifugation technique (mAECT), following established protocol for *T. b. brucei* and *T. b. evansi*.^75–77^ Test compounds were serially diluted 4-fold, starting from the highest in-test concentration of 64 μM. Parasites were then seeded at the density of 1 × 10^5^ parasite per well. After a 24 h incubation period with the compounds at 37 °C in a 5% CO_2_ atmosphere, resazurin was added to assess cell viability. Plates were further incubated under identical conditions for another 24 h. Cell viability was determined by fluorescence reading (Tecan, GENios), and EC_50_ values were determined by nonlinear regression analysis of dose–response curves with variable slopes, performed using Prism version 10.4 (GraphPad Software Inc., San Diego, CA, USA).

##### Ethics Statement

The use of laboratory rodents was carried out in strict accordance to all mandatory guidelines (EU directives, including the Revised Directive 2010/63/EU on the Protection of Animals used for Scientific Purposes that came into force on 01/01/2013, and the declaration of Helsinki in its latest version) and was approved by the Ethical Committee of the University of Antwerp, Belgium [UA-ECD 2023–76].

#### Evaluation of Compounds **5–25** against *L. mexicana*

##### Parasite Strain and Cell Cultures

*L. mexicana* cas9 T7 strain^54^ was used as promastigotes grown in HOMEM (Life Technologies) supplemented with 10% FBS 1% of a penicillin–streptomycin solution (Life Technologies) at 25 °C, as described.^55^ These assays were performed using doubling dilution series starting at 100 μM of test compound, over either 1 rows of a 96-well culture plate with the last well left for no-drug control—i.e. Eleven 2-fold dilutions performed in 100 μL of the appropriate medium for the parasite species. For *L. mexicana*, the procedure and concentrations were the same, but the wells received 100 μL of 2 × 10^6^ promastigotes, the plates were incubated at 27 °C for 72 h before and for 48 h after the addition of the resazurin solution.^78^ Fluorescence was read on a FLUOstar Optima plate reader (BMG Labtech, Ortenberg, Germany) at excitation wavelength 544 nm and emission wavelength 620 nm. The EC_50_ values and fluorescence data were plotted to a sigmoid curve with variable slope, using the GraphPad Prism 9 software package to obtain the EC_50_ values. All EC_50_ values were determined on at least three independent occasions and are presented as average ±SEM.

#### Evaluation of Compounds **5**–**25** against *L. amazonensis*

##### Compounds

A stock solution of the compounds at a concentration of 10 mM was prepared in DMSO. Miltefosine (Sigma) stock solutions in DMSO were prepared at 30 mM.

##### Activity in *L. amazonensis* *Ex Vivo* Model

*L. amazonensis* (MHOM/BR/77/LTB0016) strain was used in all assays and was purified from mice lesions. Male BALB/c mice (weighing 18–21 g) were provided by the Institute of Sciences and Technologies in Biomodels (ICTB-Fiocruz/RJ/Brazil). The animals were housed in propylene cages (max. Six animals per cage) at ambient temperature (22 ± 2 °C) under a 12 h light/dark cycle, supplying sterilized water and chow ad libitum. The animals were subcutaneously infected (left hind footpad) with 20 μL containing 106 amastigotes. *Ex vivo* assays were performed to evaluate the direct effect of **13–20** on the free amastigotes purified directly from mouse lesions as described by Santos et al.^56^ Parasites were seeded with a density of 10^7^/mL parasites per well (100 μL) in a 96-well plate. Then, 100 μL of each agent was added. In a first step, amastigotes were incubated for 48 h using a fixed concentration (30 μM). Those compounds that reduced ≥50% the amastigote’s viability were moved to dose–response curves (up to 30 μM). MIL was used as reference drug in all assays. Parasite viability was assessed by AlamarBlue reaction (560–590 nm) to calculate the EC_50_ values by a nonlinear regression curve.^56^

##### Peritoneal Mouse Macrophage (PMM) Infection by Amastigotes of *L. amazonensis*

After 24 h of plating, the PMM cultures were rinsed with phosphate buffered saline (PBS) and infected with amastigotes of *L. amazonensis* at 2.5:1 parasite/host cell ratio with an interaction time of 2 h, as reported by Santos et al.^56^ To favor the parasite-host cell interactions to occur, 500 μL of medium containing parasites was added to each well of the 24-well plates instead of the usual 1 mL volume of medium. The plates were then incubated at 37 °C and 5% CO_2_. After the interaction time, the medium containing noninternalized parasites was removed, the host cells were rinsed twice with PBS, and the medium replaced with supplemented RPMI 1640 medium. After 24 and 48 h of incubation, the cultures were fixed using Bouin solution and stained using 10% Giemsa (Sigma-Aldrich). The coverslips were then fixed on a microscopic slide with Permount mounting medium. At least 200 randomly selected cells per microscopic slide were examined at ×1000 magnification under a Zeiss Axioplan microscope (Carl Zeiss Inc., Thornwood, NJ) to determine the percentage of PMMs containing amastigotes, the number of amastigotes per infected host cell and the infection indexes (multiplication of the former two parameters—the percentage of infected host cells by the number of parasites per infected host cell).^56^

##### Data Analysis and EC_50_ and LC_50_ Calculation

EC_50_, and LC_50_ calculation, as well as the 95% confidence interval presented in lieu of standard deviation, were performed by Prism Graphpad Version 9.1.0 using nonlinear regression with the data obtained in at least two assays in triplicate.

##### Ethics Statement

All procedures have been carried out following the ethical guidelines established and approved by the Committee of Ethics for the Use of Animals (CEUA) of Fiocruz (number: L-038/2017-A4, L-017/2023).

### *In Vitro* Drug Susceptibility growth Tests

In the first series of experiments, *T. b. brucei* 427WT cells were grown to mid log-phase in standard HMI-9/FBS medium distributed in in six-well plates at 2 × 10^5^ cells mL^–1^ and incubated with two different concentrations of test compounds (2 × EC_50_, 5 × EC_50_) for 48 h at 37 °C and 5% CO_2_. Untreated parasites, used as control, were grown in parallel. The cells were counted by using hemocytometer cell counter (cell count/mL × 10^4^) at 0, 8, 16, 24, 32, 40, 48 h of incubation. Each experiment was performed as two independent replicates, and each sample was counted at least twice. In the second series, the cells were washed by centrifugation into drug-free fresh medium at preset times (either 8 h, 16 h or 24 h for different experiments) and incubated further under the same conditions until a total of 72 h.

### Mode of Action studies

#### Propidium Iodide Assays of Cellular Permeability

In a first experiment, a doubling dilution of **5** in 100 μL HMI-9/10% FCS, ranging from 80 nM to 1.25 nM final concentration) was prepared in white 96-well plates (Greiner) at 2× strength; control cells received 100 μL of medium without drugs or phenylarsine oxide (PAO; Sigma) for a final concentration of 0.33 μM. To each well 100 μL of a suspension of 2 × 10^7^ *T. b. brucei* bloodstream forms of strain s427 (WT) was added. The reaction was then started by the addition of propidium iodide (PI, 9 μM final concentration) and the plates were placed in a FLUOstar OPTIMA fluorimeter (BMG Labtech) at 37 °C with 5% CO_2_, and the fluorescence should be observed at 544 nm excitation and 620 nm emission for 10 h. The fluorescence values were graphically visualized using GraphPad Prism version 9.0 or higher. The second experiment was performed essentially identically but three separate plates were prepared in parallel, and placed in an incubator (37 °C, 5% CO_2_) and PI was only added when the plate was placed in the fluorimeter, 30 min before the final time point (16 h, 24 or 32 h). Fluorescence was recorded for this half hour and final values taken for graphical analysis in Prism.

### Evaluation of the Antileishmanial Activity of Compound **5** and **7** Associated with Plasma Membrane Rigidity

#### Chemicals

5-Doxyl-stearic acid (5-DSA), RPMI-1640 culture medium, l-glutamine, penicillin G, streptomycin, 3-(4,5-dimethylthiazol-2-yl)-2,5-diphenyl tetrazolium bromide (MTT) and MTF were purchased from Sigma-Aldrich (St. Louis, MO, USA). Heat-inactivated fetal bovine serum (FBS) was purchased from Corning Life Sciences (NY, USA).

#### Cell Viability Assays in *L. amazonensis* and J774.A1 Macrophagesquio

Promastigotes of the *L. amazonensis* (IFLA/BR/67/PH8 or 75/Josefa reference strain) were cultivated at 26 °C in 24-well plates containing 2 mL of RPMI 1640 culture medium, supplemented with 20% FBS, 2 mM l-glutamine, 100 U/mL penicillin and 100 μg/mL streptomycin. Promastigotes at the sixth day of growth in culture (stationary growth phase) were used in 96-well plates at a cell concentration of 1 × 10^7^ cells/mL, containing 100 μL of RPMI medium supplemented with 10% FBS in each well. **5** and **7** compounds were first diluted in DMSO to 10 mM and then further diluted into the culture medium before parasites were added. Final concentrations of the compounds ranged from 0.2 to 8 μM. MIL, initially diluted in ethanol, was used as a positive control. After incubation at 26 °C for 24 h, cell viability was assessed by measuring the reduction of water-soluble methylthiazolyldiphenyl-tetrazolium bromide (MTT) to formazan by mitochondrial reductases. The compounds’ half-maximal inhibitory concentration (IC_50_) values were determined by fitting a sigmoid curve to the concentration–response data. J774A.1 macrophages (ATCC TIB-67) were maintained in RPMI 1640 medium (pH 7.4), supplemented with l-glutamine and 10% FBS, and incubated at 37 °C in an atmosphere of 5% CO_2_. Macrophages at a concentration of 1 × 10^5^ cells/mL in RPMI medium supplemented with 10% FBS were cultured in 96-well plates and maintained at 37 °C with 5% CO_2_ for 2 h to allow adherence. Then, the compounds were added to the wells at increasing concentrations from 0.6 to 80 μM, with a final volume of 200 μL. Cytotoxicity was also assessed in macrophages infected with *L. amazonensis*. In these experiments, after allowing 2 h period for the macrophages to adhere to the 96-well plate, parasites were added at a ratio of 20 per macrophage. After 24 h of infection, the culture medium was replaced to remove as many noninternalized parasites as possible, and the cells were then exposed to the compounds BIM7 and MB7. After incubation for 24 or 48 h, 20 μL of MTT was added to each well to estimate cell viability. The cytotoxic concentration (CC_50_) was then calculated using the same method described above for the *Leishmania* parasite.

#### Hemolytic Potential

Human blood was first treated with EDTA, diluted three times in PBS, and then centrifuged at 1800*g* for 10 min at 4 °C. After removing the plasma, the cells were washed twice with PBS. Compounds previously dissolved in DMSO at 10 mM were prediluted in 1.8 mL of PBS before adding 200 μL of PBS containing the erythrocytes. Assuming an erythrocyte volume of 90 fL, the final erythrocyte concentration in the suspension was adjusted to 5 × 10^7^ cells/mL, equivalent to a 0.45% hematocrit. After incubation at 37 °C, the cells were centrifuged at 10,000*g*, and the amount of hemoglobin in the supernatant was evaluated by measuring the absorbance at 540 nm. The percentage of hemolysis was calculated based on the total hemolysis obtained by incubating the cells in ultrapure water. The concentration of the compound that causes 50% hemolysis (HC_50_) of the cells was determined by fitting a sigmoid curve to the experimental data.

#### EPR Spectroscopy in Parasites, J774A.1 Macrophages and Leishmania-Infected Macrophage

Similar to the antiproliferative assay, the *L. amazonensis* promastigotes were diluted in RPMI 1640 supplemented with 10% FBS in 24-well culture plates, achieving a final cell concentration of 2 × 10^7^ parasites/mL (2 mL per well). The promastigotes were then treated with the compounds at different concentrations ranging from 0.5 to 5 μM. Compounds **5** and **7** were initially diluted in DMSO to a concentration of 10 mM, then further diluted into the culture medium before the parasites were added. After 24 h at 26 °C, the samples were centrifuged at 25,000*g* for 10 min. The supernatant was discarded and the parasites were washed once more in 1 mL of PBS and then resuspended in 50 μL of PBS. Each sample containing 4 × 10^7^ parasites was spin-labeled with 5-DSA. To incorporate the spin label into the parasite membranes, a 0.5 μL aliquot of a 5-DSA ethanolic solution (5 mg/mL) was added to each 50 μL sample. For the EPR measurement, the sample was transferred to a 1 mm i.d. capillary tube, which was flame-sealed at one end. The capillary was centrifuged at 2,5000*g* for 5 min, and the parasite pellet, approximately 2 mm in size, was placed in the center of the resonance cavity of a Bruker EPR EMXplus spectrometer (Bruker BioSpin GmbH, Rheinstetten, Germany). EPR spectra were recorded with the following instrumental settings: microwave power, 20 mW; microwave frequency, 9.45 GHz; modulation amplitude, 1.0 G; magnetic field scan, 100 G; sweep time, 168 s; and sample temperature, 25 ± 1 °C. The EPR experiments with macrophages were performed using a procedure similar to that used for *Leishmania* measurements, with the differences that the macrophage concentration was 30-fold lower (5 × 10^5^ cells/mL) and that exposure to the compounds was carried out after a 2 h period for the macrophages to adhere to the 24-well plate. In the case of macrophages infected with *Leishmania*, the same macrophage concentration and 2 mL sample volume were used. Macrophages were added to the wells and allowed to adhere to the plate for 2 h. After this period, *L. amazonensis* promastigotes were added at a ratio of 20 parasites per macrophage. After 24 h, most of the noninternalized *Leishmania* were removed by changing the medium. The cells were then exposed to the compounds **5** and **7**. After 72 h of incubation, the cells and culture medium were collected from the plate and transferred to Eppendorf tubes, which were then centrifuged at 4,500*g* for 10 min at 4 °C. Cells were washed once with 1 mL of PBS, and the procedures for spin labeling and EPR measurements were performed as described above.

#### Statistical Analysis

Data were expressed as means ± standard deviations (SD) from at least three independent experiments. The means were compared using a one-way analysis of variance (ANOVA). Tukey’s test was used to identify significant differences (*P* < 0.05) between the means of the different treatments.

### Evaluation of *in Vivo* acute Ecotoxicity of Compounds **5** and **7**

#### Test Organisms

*D. magna* and zebrafish (*D. rerio*) are globally recognized standard freshwater species for aquatic toxicity testing, and these animal models were employed in this study to determine the acute ecotoxicity of compounds **5** and **7**. Waterborne exposure was carried out for both *D. magna* and zebrafish. The sex characteristics for <24 h old neonates of *D. magna* and embryo-larval zebrafish cannot be determined at this stage.^79,80^

#### *D. magna* (*D. magna*) Maintenance and Exposure

*D. magna* neonates (<24 h old) were obtained from a commercial supplier (Environmental Consulting & Testing, Wisconsin, USA), which were cultured and maintained following US Environmental Protection Agency.^81^ *D. magna* was fed with *Selenastrum capricornutum* and a mixture of yeast-cerophyll-tout (YCT) and acclimated for 2 h prior to the exposure in a temperature-controlled incubator under a 16:8 h light/dark photoperiod at 21 ± 1 °C. Acute toxicity immobilization tests for *D. magna* were conducted according to the US EPA Methods for Aquatic Toxicity Procedures^82^ in the Baylor Environment & Health (BEHR) Laboratory at Baylor University. Two replicates with five neonates were placed in each glass beakers filled with 25 mL exposure solutions to the given treatment (i.e., water control, solvent control (0.01% MeOH v/v), 0.32, 0.63, 1.25, 2.5, 5, and 10 mg/L of compound **5** or **7**) for 4 h under a 16:8 h light/dark photoperiod at 21 ± 1 °C. The immobility of *D. magna* was monitored every 24 h after the exposure.

#### Zebrafish (*D. rerio*) Maintenance and Exposure

Wild-type (5D strain) adult zebrafish (*D. rerio*) were maintained in a temperature-controlled room at 26 ± 1 °C with a 16:8 h light/dark photoperiod and fed with freshly hatched *Artemia nauplii* twice a day. On the initial day of experiment, fertilized embryos were collected in the early morning, prior to initiation of exposure. For fish acute toxicity tests, freshly fertilized wild-type zebrafish embryos (<2 h of fertilization, hpf) were obtained from healthy adult zebrafish in the Baylor Environment & Health (BEHR) Laboratory at Baylor University. The US EPA Methods for Aquatic Toxicity Procedures^82^ was adopted for 96 h exposure. Two replicates with 10 embryos were placed in each glass beaker filled with 50 mL exposure solutions to the given treatment (i.e., water control, solvent control (0.01% MeOH v/v), 0.32, 0.63, 1.25, 2.5, 5, and 10 mg/L of compound 5 or 7). The exposure media were freshly prepared and renewed after 48 h. The number of dead embryos and larvae was checked routinely every 24 h.

#### Statistical Analysis

Statistical analysis was performed using SPSS version 22.0 for Windows (SPSS, Chicago, IL, USA). The immobility of *D. magna* and mortality of embryo-larval zebrafish in response to different treatments were analyzed with probit analysis to determine the 48 h EC_50_ and the 48- and 96 h LC_50_ values, respectively.

#### Ethics Statement

The zebrafish exposures have been carried out following the ethical guidelines established and approved by the Institutional Animal Care and Use Committee (IACUC) of Baylor University, TX, USA (No. 1069945-16).

## Supplementary Material





## Notes

Preliminary aquatic toxicity prediction, in silico physicochemical descriptors calculation, ^1^H, ^13^C, ^31^P NMR and HPLC-MS of final compounds **5**–**13̧** ^1^H, ^13^C NMR and HPLC–MS of final compounds **14**–**25**, HRMS of compounds **5** and **7** (PDF)
Molecular formula strings and associated data for final compounds **5**–**25** and intermediates **29**–**31** (CSV)

## Abbreviations

2-MeTHF: 2-methyltetrahydrofuran
AAT: animal African trypanosomiasis
AmB: amphotericin B
CD: Chagas disease
CL: cutaneous leishmaniasis
CNSL: cashew nuyshell liquid
DA: diminazene aceturate
DMF: dimethylformamide
EPR: electron paramagnetic resonance
HAPT: high-affinity pentamidine transporter
HAT: human African trypanosomiasis
HFF: human foreskin fibroblast
ISM: isometadinium
ISMc: isometamidium chloride
MCL: muco-cutaneous leishmaniasis
MIL: miltefosine
MoA: mode of action
NTTAT: nontsetse-transmitted animal trypanosomiasis
PAO: phenylarsine oxide
PI: propidium iodide
PMD: pentamidine
PMM: peritoneal mouse macrophages
QSAR: quantitative structure–activity relationship
RF: resistance factor
ROS: reactive oxygen species
SHAM: salicylhydroxamic acid
SI: selectivity index
SUR: suramin
TAO: trypanosome alternative oxidase
TPP^+^: triphenylphosphonium
TPP: target product profiles
VBPDs: vector-borne parasitic diseases
VL: visceral leishmaniasis
WHOA: World Organisation for Animal Health
WT: wild-type
