# Supplementary material for: Sustainable Antiparasitic Agents from an Agro-Industrial Waste: Mitochondria-Targeting Cashew Nutshell Liquid-Derived Phosphonium and Ammonium Salts
Source: J Med Chem. 2025 Sep 8;68(18):19438–62. doi: 10.1021/acs.jmedchem.5c01617 (PMC12481484; doi:10.1021/acs.jmedchem.5c01617)
Supplement: Supplementary file 1 [file jm5c01617_si_001.pdf]

SUPPORTING INFORMATION

Sustainable antiparasitic agents from an agro-industrial waste: mitochondria-targeting cashew nutshell liquid-derived phosphonium and ammonium salts

Bianca Martinengo,[1] Cecilia Baldassarri,[2,3] Kayhan Ilbeigi,[4] Hamed E. Alkhalaf,[3] Aditya Sarode,[3,5] Ehab Kotb Elmahallawy,[3,6,7] Ba Reum Kwon,[8] Amos Sarpong Agyei,[8] Aigerim Abdimanova,[8] Ludmila Ferreira de Almeida Fiuza,[9] Raquel Azevedo,[9] Ketlym da Conceição,[9] Marcos Meuser Batista,[9] Ellyêssa Nascimento Borges,[10] Kleber Santiago Freitas e Silva,[10] Éder Jéferson Souza Cardoso,[11] Natália Cipriano Monteiro,[12] Laís Flávia Nunes Lemes,[12] Luiz Antonio Soares Romeiro,[12] Antonio Alonso,[11] Maria de Nazaré Correia Soeiro,[9] Guy Caljon,[4] Bryan W. Brooks,[8,13] Harry P. De Koning,[3] Maria Laura Bolognesi*[1]

[1]Department of Pharmacy and Biotechnology, Alma Mater Studiorum - University of Bologna, Via Belmeloro 6, 40126, Bologna, Italy

[2]Medicinal Chemistry Unit, School of Pharmacy, Chemistry Interdisciplinary Project (ChIP), University of Camerino, Via Madonna delle Carceri, 62032 Camerino, Italy

[3]School of Infection and Immunity, College of Medical, Veterinary and Life Sciences, University of Glasgow, Glasgow G43 2DX, United Kingdom

[4]Laboratory of Microbiology, Parasitology and Hygiene (LMPH), Infla-Med Centre of Excellence, University of Antwerp, 2610 Wilrijk, Belgium

[5]Department of Pure & Applied Chemistry, University of Strathclyde, 295 Cathedral Street, Glasgow, G1 1XL, United Kingdom

[6]Department of Zoonoses, Faculty of Veterinary Medicine, Sohag University, Sohag 82524, Egypt

[7]Grupo de investigación en Sanidad Animal y Zoonosis (GISAZ), Unidad de Investigación Zoonosis y Enfermedades emergentes (ENZOEM), Departamento de Sanidad Animal, Universidad de Córdoba, 14071, Córdoba, Spain

[8]Department of Environmental Science, Baylor University, Waco, TX76798-7266, USA

[9]Laboratório de Biologia Celular do Instituto Oswaldo Cruz, Fiocruz. Avenida Brasil 4365, Manguinhos, CEP 21040360, Manguinhos, Rio de Janeiro, Brazil

[10]Faculdade de Farmácia, Universidade Federal de Goiás, 74605-220, Goiânia, GO, Brazil

[11]Instituto de Física, Universidade Federal de Goiás, 74690-900, Goiânia, GO, Brazil

[12]Laboratório de Desenvolvimento de Inovações Terapêuticas, Núcleo de Medicina Tropical, Faculdade de Medicina, Universidade de Brasília, 70910-900, Brasília, Brazil

[13]Department of Public Health, Baylor University, Waco, TX76798-7343, USA

*Corresponding author: Maria Laura Bolognesi (marialaura.bolognesi@unibo.it)

Table of Contents

1. Preliminary Aquatic Toxicity Prediction Using ECOSAR v2.2 4

2. *In silico* physicochemical descriptors calculation 6

3. Chemistry 10

3.1 Synthesis of CNSL-derived C8-mesylates (26 – 28) 10

4. Spectral copies of ^{1}H, ^{13}C NMR, ^{31}P NMR and HPLC-MS of final compounds 5 – 13 12

^{1}H NMR, ^{13}C NMR, ^{31}P NMR, HPLC-MS chromatogram and HRMS of compound **5**. 12

^{1}H NMR, ^{13}C NMR, ^{31}P NMR and HPLC-MS chromatogram of compound **6**. 15

^{1}H NMR, ^{13}C NMR, ^{31}P NMR, HPLC-MS chromatogram and HRMS of compound **7**. 17

^{1}H NMR, ^{13}C NMR, ^{31}P NMR and HPLC-MS chromatogram of compound **8**. 20

^{1}H NMR, ^{13}C NMR, ^{31}P NMR and HPLC-MS chromatogram of compound **9**. 22

^{1}H NMR, ^{13}C NMR, ^{31}P NMR and HPLC-MS chromatogram of compound **10**. 24

^{1}H NMR, ^{13}C NMR, ^{31}P NMR and HPLC-MS chromatogram of compound **11**. 26

^{1}H NMR, ^{13}C NMR, ^{31}P NMR and HPLC-MS chromatogram of compound **12**. 28

^{1}H NMR, ^{13}C NMR, ^{31}P NMR and HPLC-MS chromatogram of compound **13**. 30

5. Spectral copies of ^{1}H, ^{13}C NMR and HPLC-MS of final compounds 14 – 25. 32

^{1}H NMR, ^{13}C NMR and HPLC-MS chromatogram of compound **14**. 32

^{1}H NMR, ^{13}C NMR and HPLC-MS chromatogram of compound **15**. 34

^{1}H NMR, ^{13}C NMR and HPLC-MS chromatogram of compound **16**. 36

^{1}H NMR, ^{13}C NMR and HPLC-MS chromatogram of compound **17**. 38

^{1}H NMR, ^{13}C NMR and HPLC-MS chromatogram of compound **18**. 40

^{1}H NMR, ^{13}C NMR and HPLC-MS chromatogram of compound **19**. 42

^{1}H NMR, ^{13}C NMR and HPLC-MS chromatogram of compound **20**. 44

^{1}H NMR, ^{13}C NMR and HPLC-MS chromatogram of compound **21**. 46

^{1}H NMR and ^{13}C NMR of compound **22**. 48

^{1}H NMR, ^{13}C NMR and HPLC-MS chromatogram of compound **23** 49

^{1}H NMR, ^{13}C NMR and HPLC-MS chromatogram of compound **24**. 51

^{1}H NMR, ^{13}C NMR and HPLC-MS chromatogram of compound **25**. 53

6. References 55

1. Preliminary Aquatic Toxicity Prediction Using ECOSAR v2.2

This model estimates if acute and chronic toxicity of a chemical to aquatic organisms, including fish, aquatic invertebrates, and aquatic plants, using predictive SAR software.

Aquatic toxicity prediction was carried out as a preliminary assessment of the compounds. ECOSAR includes a maximum for the log K_{ow} value that indicates if the compound is insoluble then it cannot develop a toxicity prediction for aquatic organisms when the log K_{ow} is higher than the limit (Table S1).

Table S1. Maximum limit of $\log_{ow}$ value for aquatic toxicity parameters in ECOSAR v2.2.

Organism			
Fish (LC_{50}[a], 96 h)	Daphnid (EC_{50}[b], 48 h)	Green algae (EC_{50}, 96 h)	ChV[c]
5.0	5.0	6.4	8.0

Value reported in mg/L. [a]LC_{50}: concentration in water that kills 50% of organism in a continuous exposure; [b]EC_{50}: concentration that gives decreases of growth of 50% of algae, or results in 50% immobilization (a surrogate for mortality) in Daphnids relative to the control in continuous exposure; [c]ChV: chronic value, is a geometric average of NOEC (non-observed effect concentration) and LOEC (lowest observed effect concentration).[1]

The program also includes classification of toxicity cutoff values (Table S2) that are used by the United States Environmental Protection Agency (US EPA), which consists of high, moderate, and low concern, assessed from the acute and chronic toxicity parameter.[2]

Table S2. Classification of aquatic toxicity levels.

Aquatic toxicity levels		
High Concern	*Moderate Concern*	*Low Concern*
Any of the 3 acute values are	Any of the 3 acute values are between 1.0 mg/L and	All 3 acute values are >100 mg/L, and all three chronic values are >10.0 mg/L, or there are "No Effects at Saturation" (or

< 1.0 mg/L, or any of the chronic values are < 0.1 mg/L	100 mg/L, OR any of the chronic values are between 0.1 mg/L and 10.0 mg/L	NES). NES occurs when a chemical is not soluble enough to reach the effect concentration, i.e., the water solubility is lower than an effect concentration, or, for liquids, when Kow criteria are exceeded for an endpoint. For solids, NES is expected if Kow exceeds the specific SAR Kow cutoffs, or the effective concentration is more than one order of magnitude (> 10 X) less than water solubility.

The toxicity prediction results (Table S3) indicated that the compounds **5** and **7** presents LC_{50} and EC_{50} values between 1.0 mg/L and 100 mg/L and ChV value comprises between 0.1 mg/L and 10 mg/L. Based on these results, both compounds can be considered as "*moderate concern*" toxicity level according to EPA classification.

Table S3. Results of prediction of aquatic toxicity for compounds 5 and 7 using ECOSAR v2.2.

cmpd	MW (g/mol)	Log K_{ow}^{a}	solubility in water (mg/mL)	Organism: Fish LC_{50}^{b} (mg/L) (96 h)	Fish ChV^{c}	Daphnid LC_{50}^{d} (mg/L) (48 h)	Daphnid ChV^{c}	Green algae EC_{50}^{e} (mg/L) (96 h)	Green algae ChV^{c}
**5**	561.53	4.64	0.03	1.95	0.254	1.41	0.271	2.88	1.30
**7**	591.56	4.73	0.02	1.73	0.228	1.27	0.249	2.67	1.22

$^{a}K_{ow}^{a}$: octanol−water partition coefficient; $^{b}LC_{50}$: concentration in water that kills 50% of organism in a continuous exposure; ^{c}ChV: chronic value, is a geometric average of NOEC (non-observed effect concentration) and LOEC (lowest observed effect concentration) (EPA, 2013); $^{d}EC_{50}$: concentration that gives decreases of growth of 50% of algae, or results in 50% immobilization (a surrogate for mortality) in Daphnids relative to the control in continuous exposure.

2. *In silico* physicochemical descriptors calculation

The design of new drug candidates for NTDs is inherently challenging, requiring a careful balance between optimizing target selectivity and pharmacokinetic properties. In this context, targeting the mitochondria presents a promising strategy, as these organelles possess unique features that can be leveraged to enhance drug accumulation. Compounds **5–25** have been specifically designed to target mitochondria, incorporating key elements to facilitate selective mitochondrial accumulation. Mitochondrial targeting compounds must navigate multiple cellular membranes to reach their destination, necessitating molecules with certain physicochemical characteristics that enable them to cross these barriers.[3] A key feature for mitochondrial accumulation is the positive charge of molecules, which is drawn to the negatively charged mitochondrial membrane potential.[4] However, to penetrate the mitochondrial matrix, molecules must also exhibit sufficient lipophilicity, as the inner mitochondrial membrane is highly lipophilic due to its cardiolipin content.[5] Therefore, compounds exhibiting both positive charge and lipophilicity are more likely to accumulate within mitochondria.[6]

To rationalize the physicochemical properties of the CNSL-derived compounds **5**–**25**, key molecular descriptors were calculated using SwissADME (http://www.swissadme.ch/) as open-access tool. Specifically, selected structural 2D descriptors for lipophilicity (aromatic rings,[7] logP), polarity (TPSA, HBD/HBA) and flexibility (rotatable bond) were calculated for **5**–**25** and reference compounds (Table S4). The logP values of these compounds, ranging from 2.32 to 9.15, suggest favourable lipophilicity, particularly for compound **5**–**10**, which are expected to easily permeate biological membranes, including the mitochondrial membranes. The topological polar surface area (TPSA) values, which reflect the polarity of the compounds, ranged from 13.11 Å^2 to 65.43 Å^2, with lower values being beneficial for membrane permeability. Similarly, the hydrogen bond acceptor (HBA)/hydrogen bond donor (HBD)

descriptors and the rotatable bond count (13–15) highlight the molecular flexibility of the compounds, which is critical for conformational adaptability and membrane interaction.

However, while high lipophilicity aids in mitochondrial accumulation, it also brings potential challenges related to solubility and bioavailability, which must be considered during further optimization. For instance, lipophilicity of compounds **11**–**13** is extremely high. These descriptors provide valuable insights into the drug-likeness of the compounds, focusing on properties critical for predicting membrane permeability, solubility, and overall bioavailability, all of which are key factors for drug efficacy, especially when targeting the mitochondria of parasites and possibly penetrating the BBB in the case of CNS involvement in *T. brucei* second-stage infections.

Finally, regarding potential concerns about the metabolic stability of the anacardic acid methyl ester derivatives **6**, **9**, **12**, **15**, **18**, **21**, and **24**, previous experiments with CNSL methyl ester analogues[8] showed no decomposition over a 6-hour period. These findings suggest that the methyl esters in the current series are also likely to be similarly metabolically stable.

Table S4. Predicted *in silico* physicochemical descriptors for compounds **5**–**25** and reference compounds.

**Cmpd**	**Structure**	**MW (g/mol)**	**n. AR**[a]	**logP**[b]	**HBA**[c]	**HBD**[d]	**TPSA (Å²)**[e]	**RB**[f]
**5**	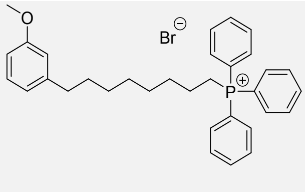	561.53	4	6.48	1	0	22.82	13
**6**	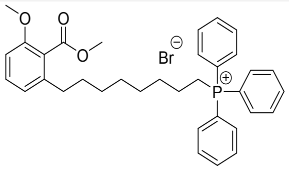	619.57	4	6.32	3	0	49.12	15

**7**	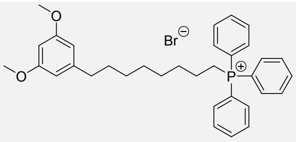	591.56	4	6.47	2	0	32.05	14
**8**	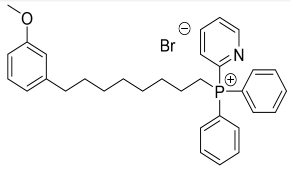	562.52	4	5.81	2	0	35.71	13
**9**	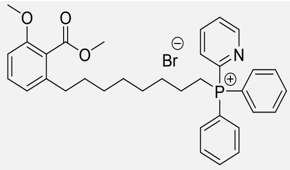	620.56	4	5.74	4	0	62.01	15
**10**	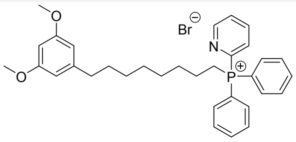	592.55	4	5.75	3	0	44.94	14
**11**	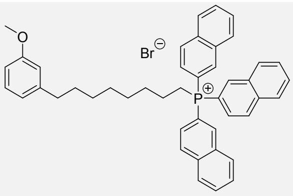	711.71	7	9.11	1	0	22.82	13
**12**	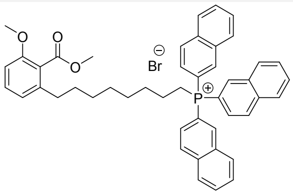	769.74	7	8.98	3	0	49.12	15
**13**	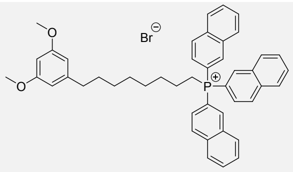	741.73	7	9.05	2	0	32.05	14
**14**	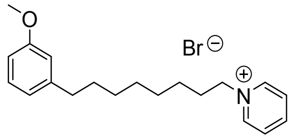	378.35	2	2.94	1	0	13.11	10
**15**	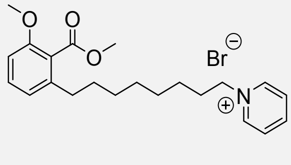	436.38	2	3.01	3	0	39.41	10

**16**	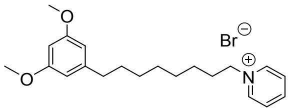	408.37	2	2.93	2	0	22.34	10
**17**	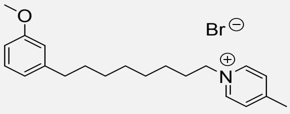	392.37	2	3.23	1	0	13.11	10
**18**	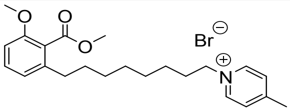	450.41	2	3.21	3	0	39.41	12
**19**	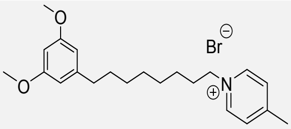	422.40	2	3.24	2	0	22.34	11
**20**	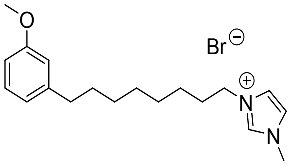	381.35	2	2.32	1	0	18.04	10
**21**	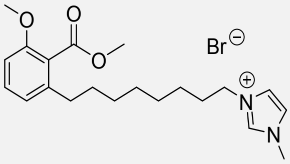	439.39	2	2.46	3	0	44.34	12
**22**	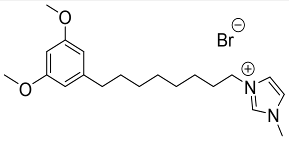	411.38	2	2.34	2	0	27.27	11
**23**	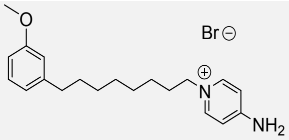	393.36	2	2.33	1	1	39.13	10
**24**	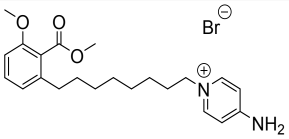	451.40	2	2.32	3	1	65.43	12
**25**	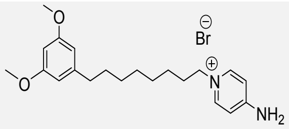	423.39	2	2.30	2	1	48.36	11
**LDT72**	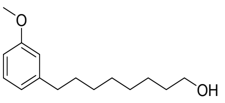	236.35	1	3.75	2	1	29.46	9

**LDT74**	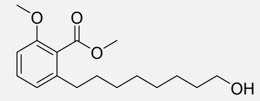	294.39	1	3.68	4	1	55.76	11
**LDT490**	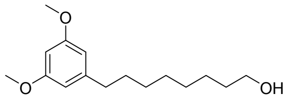	266.38	1	3.67	3	1	38.69	10
**PMD[g]**		340.42	2	2.72	4	4	118.20	10
**SUR[h]**		1297.28	8	2.59	23	12	534.03	22
**DA[i]**		515.52	2	0.90	19	9	269.29	11
**Bz[j]**		260.25	2	0.49	4	1	92.74	6
**AmB[k]**		n.d.	n.d.	n.d.	n.d.	n.d.	n.d.	n.d.
**MIL[l]**		407.57	0	3.35	4	0	68.40	20

[a]n. AR: n. aromatic rings; [b]consensus logP; [c]HBA: H-bond acceptors; [d]HBD: H-bond donors; [e]TPSA: Topological Polar Surface Area; [f]RB: rotatable bonds; [g]PMD: pentamidine; [h]SUR: suramine; [i]DA: diminazene aceturate; [j]Bz: beznidazole; [k]AmB: amphotericin B; [l]MIL: miltefosine.

3. Chemistry

3.1 Synthesis of CNSL-derived C8-mesylates (26 – 28)

Scheme S1. Synthesis of CNSL-derived C8-mesylates (**26-28**)[a]

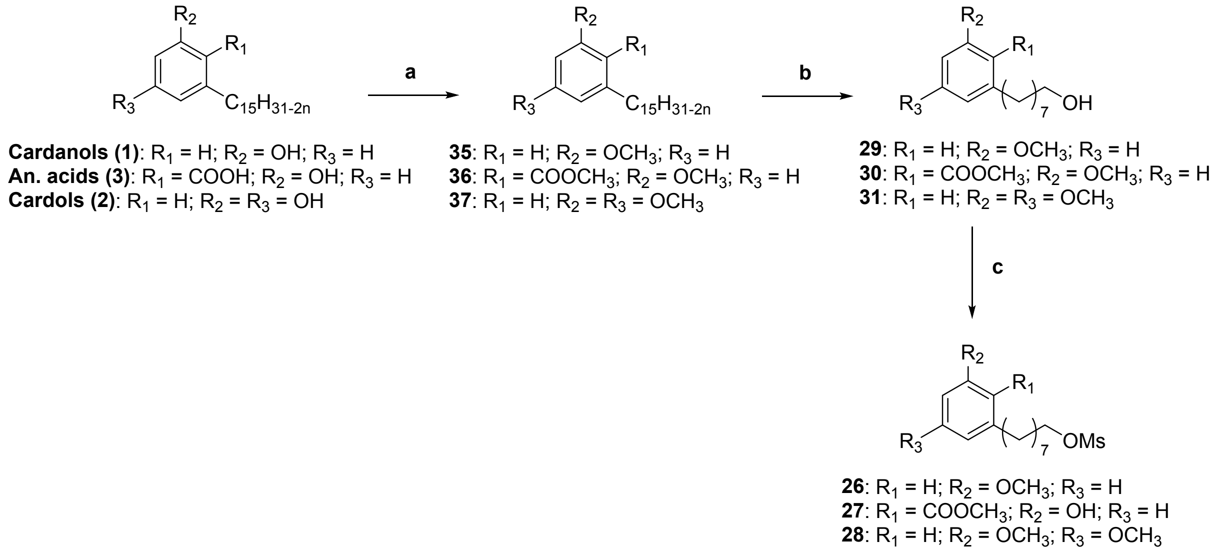

[a]Reagents and conditions: (a) K_2CO_3 and acetone; MeI, 110 °C, 24 h (66−80%); (b) 1) O_3, DCM/MeOH, 0 °C; 2) $NaBH_4$, rt, 24 h (60−70%), (c) methanesulfonyl chloride, TEA, DCM, 12 h, and rt (60–85%).

4. Spectral copies of ^{1}H, ^{13}C NMR, ^{31}P NMR and HPLC-MS of final compounds 5 – 13

^{1}H NMR, ^{13}C NMR, ^{31}P NMR, HPLC-MS chromatogram and HRMS of compound **5**.

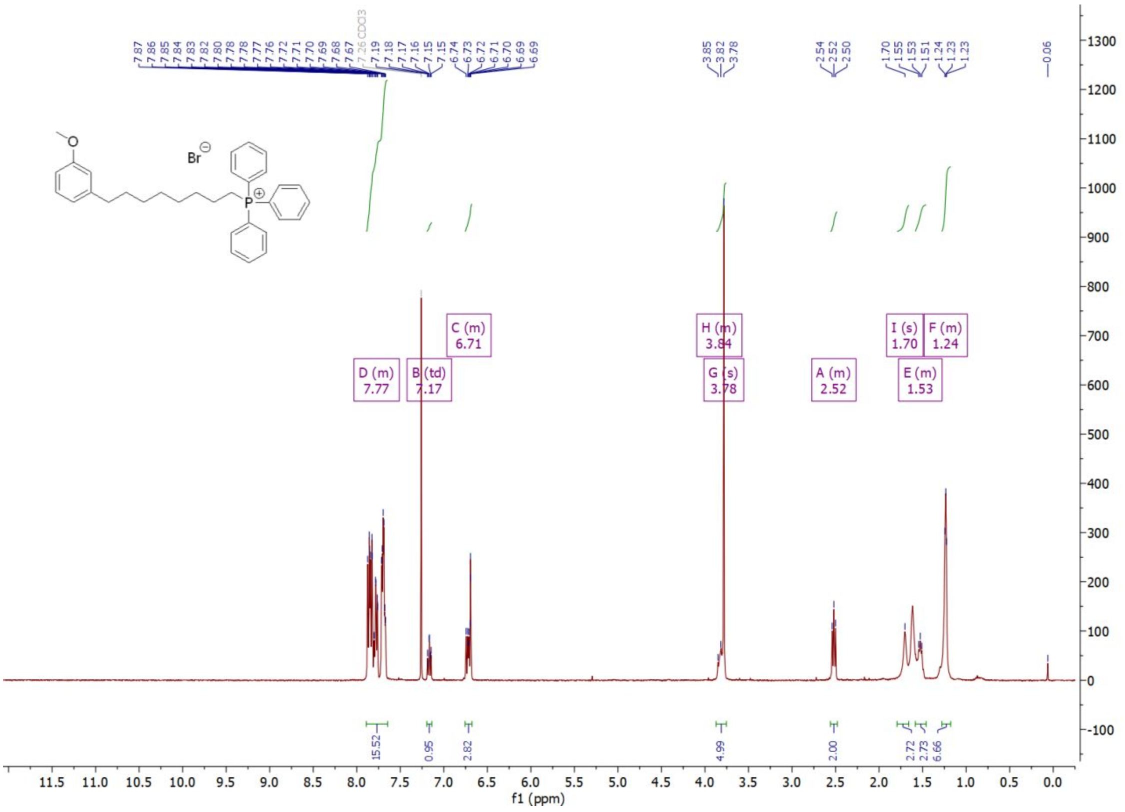

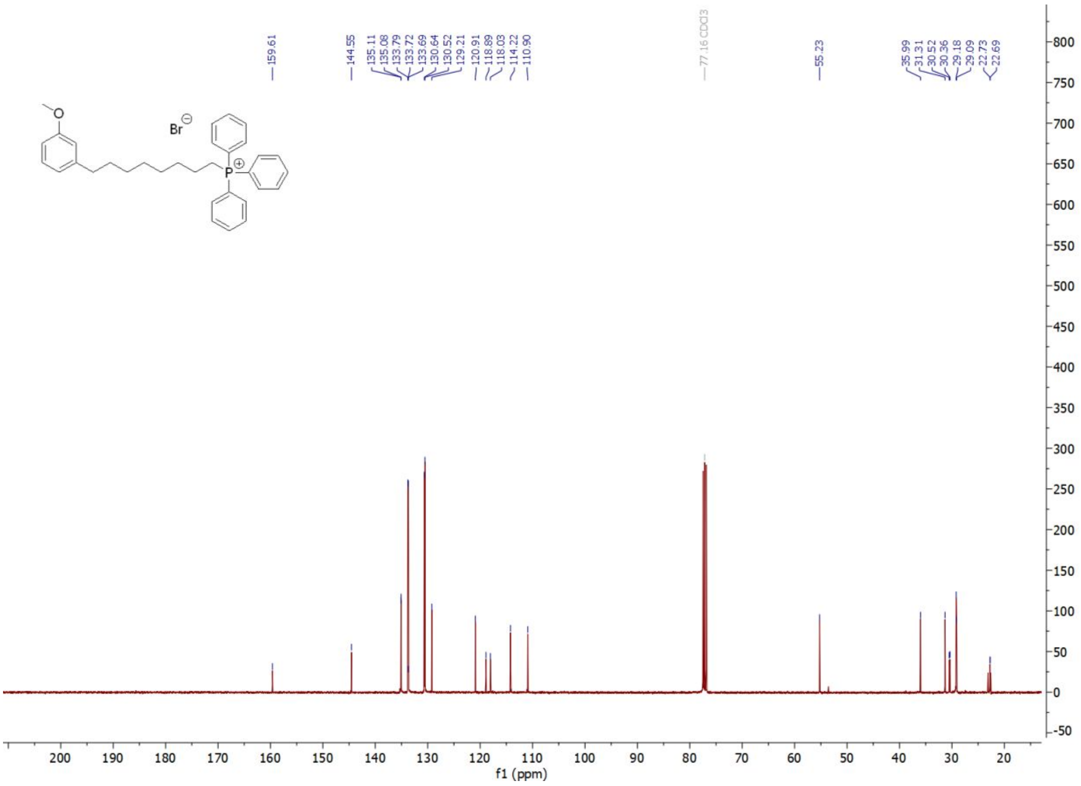

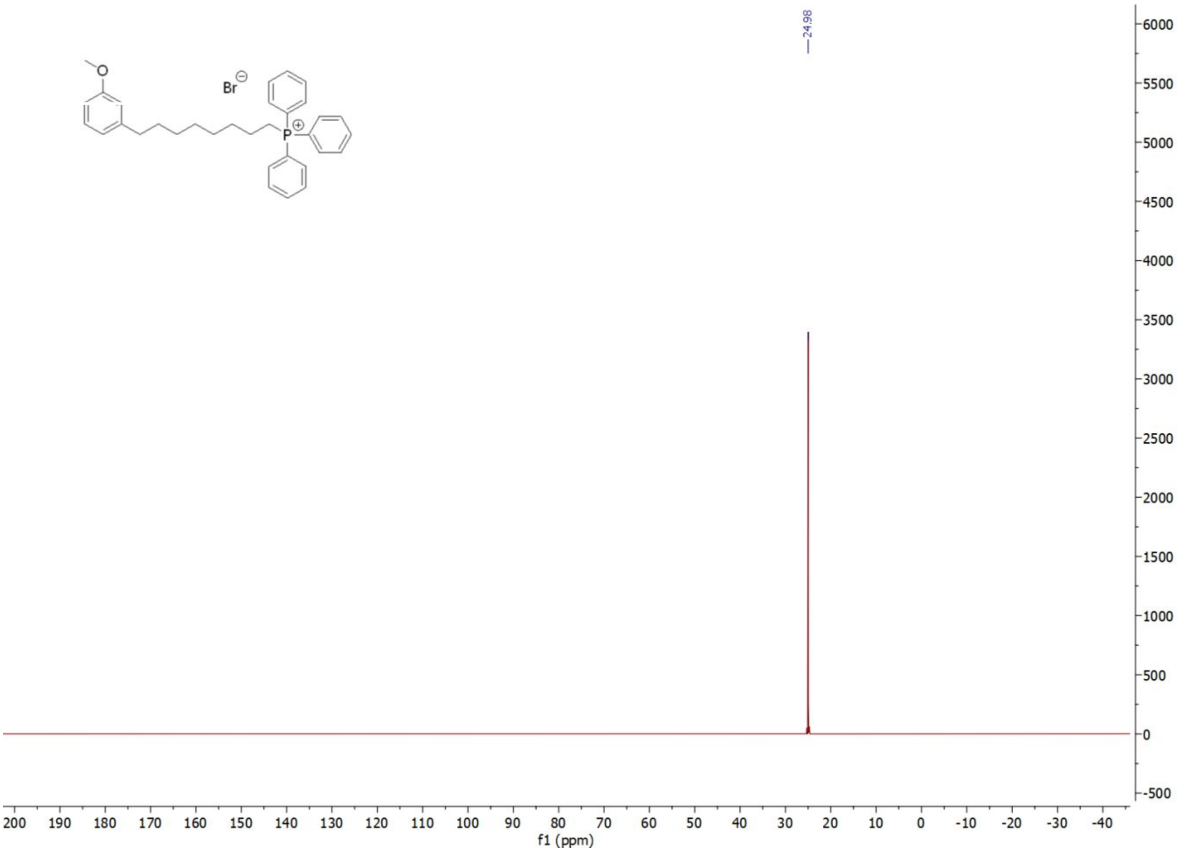

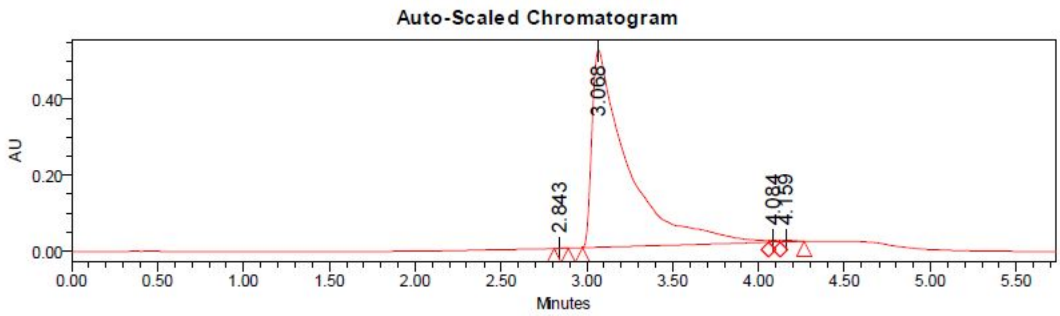

Peak Results

	Name	RT	Area	Height	% Area
1		2.843	1928	775	0.03
2		3.068	7605218	520216	99.46
3		4.084	17315	4905	0.23
4		4.159	22432	4489	0.29

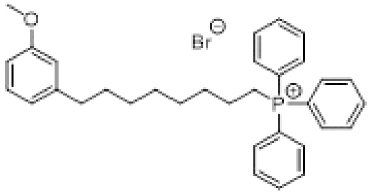

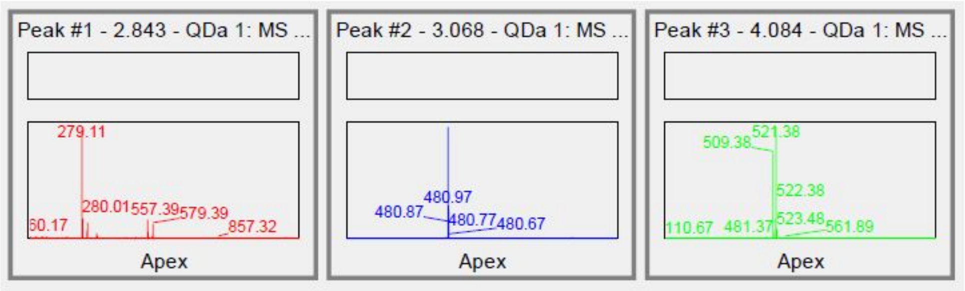

Item name: BIM7_1ul
Item description:
Component name: BIM7-Br

Channel name: Time 0.1420 +/- 0.0072 minutes

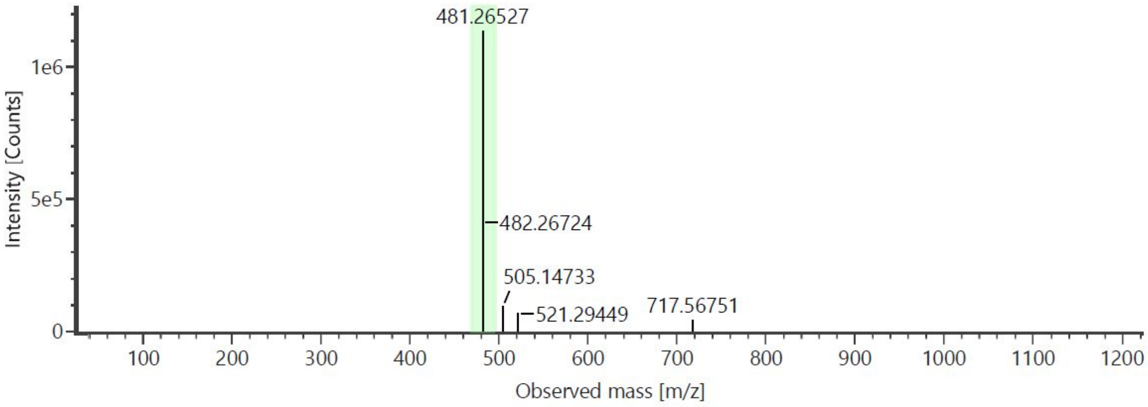

^{1}H NMR, ^{13}C NMR, ^{31}P NMR and HPLC-MS chromatogram of compound **6**.

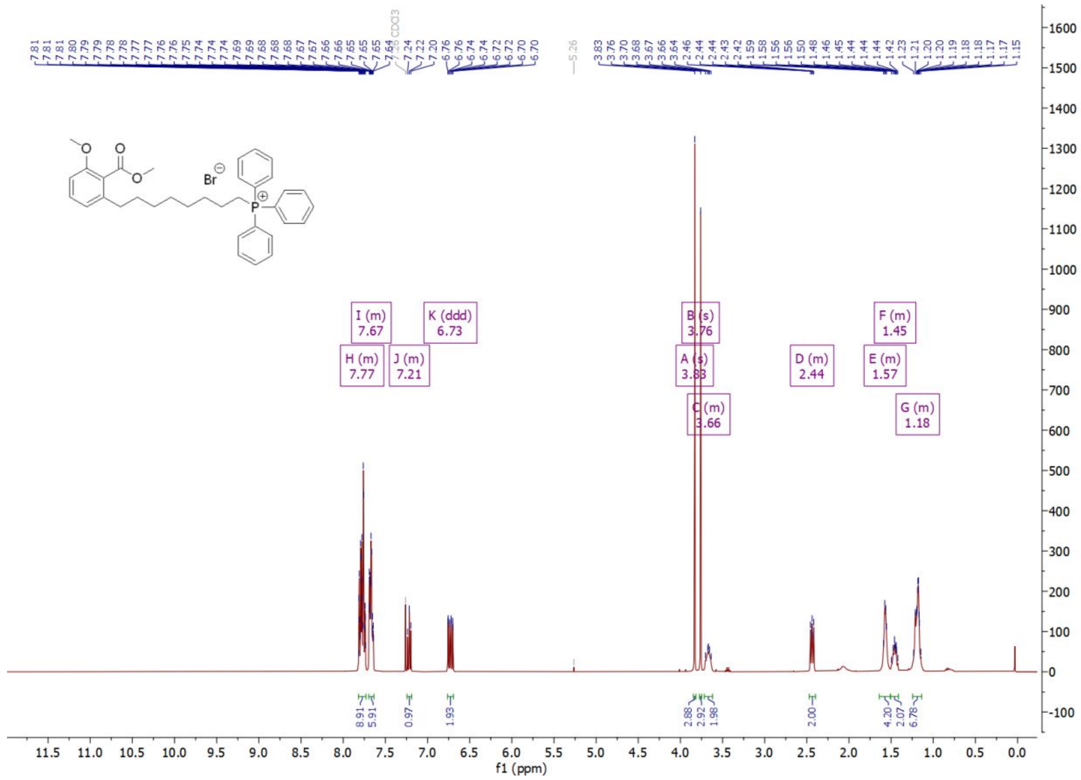

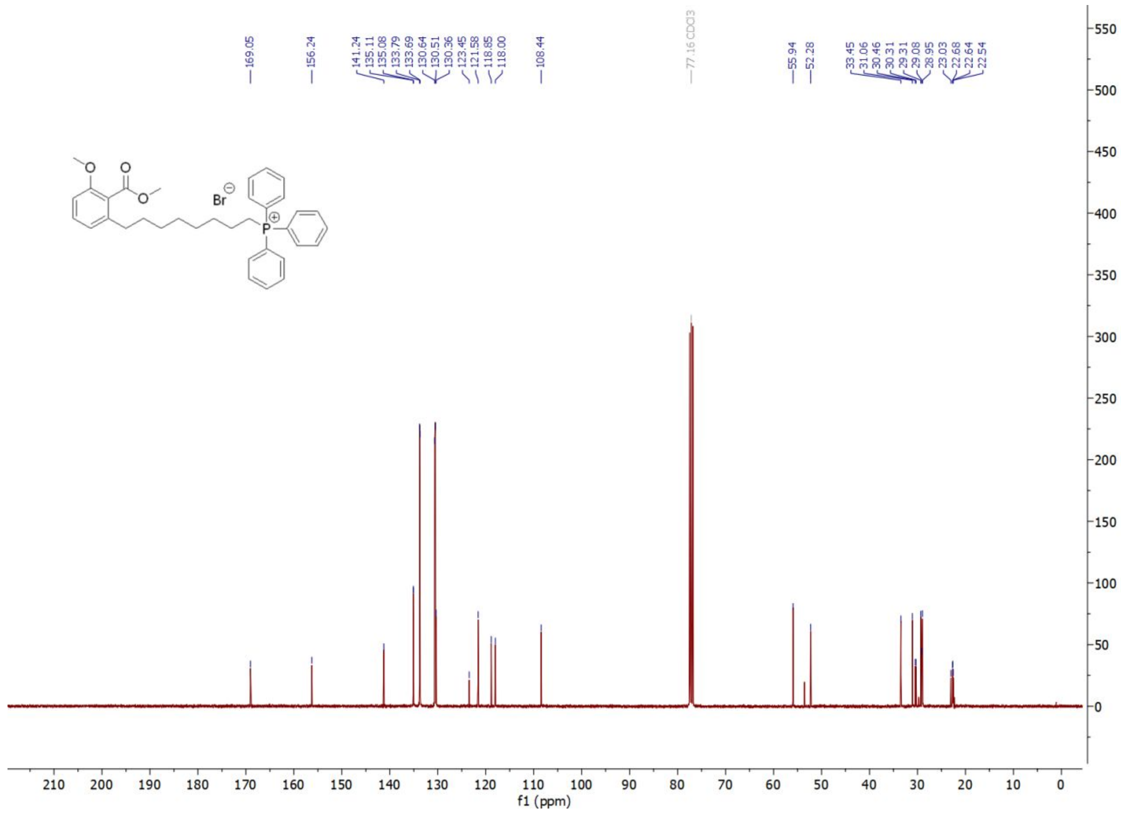

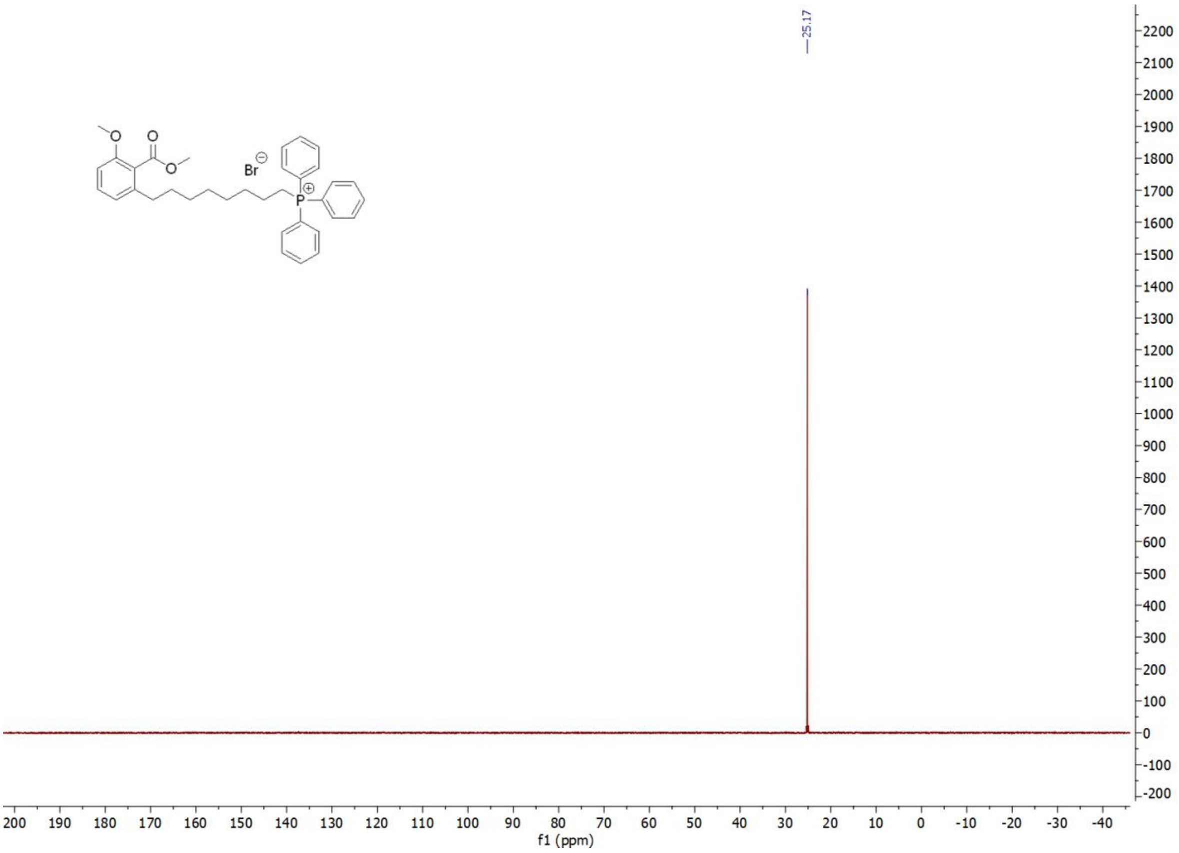

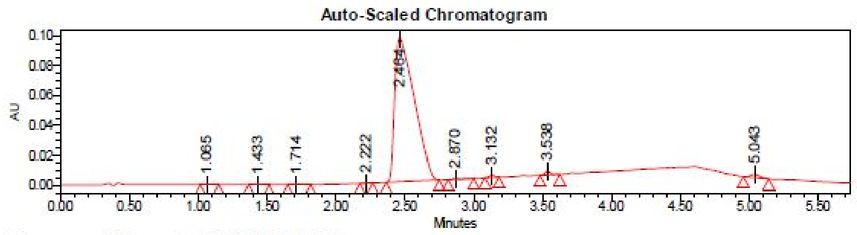

Processed Channel: W2489 ChA 254nm

	Processed Channel	Retention Time (min)	Area	% Area	Height
1	W2489 ChA 254nm	1.065	1276	0.13	330
2	W2489 ChA 254nm	1.433	1391	0.14	259
3	W2489 ChA 254nm	1.714	1151	0.12	198
4	W2489 ChA 254nm	2.222	424	0.04	135
5	W2489 ChA 254nm	2.464	944135	97.24	96850
6	W2489 ChA 254nm	2.870	2648	0.27	574
7	W2489 ChA 254nm	3.132	3868	0.40	1437
8	W2489 ChA 254nm	3.538	6547	0.67	2514
9	W2489 ChA 254nm	5.043	9490	0.98	2214

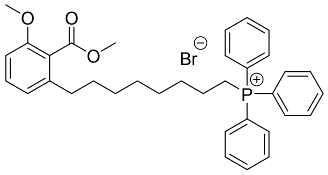

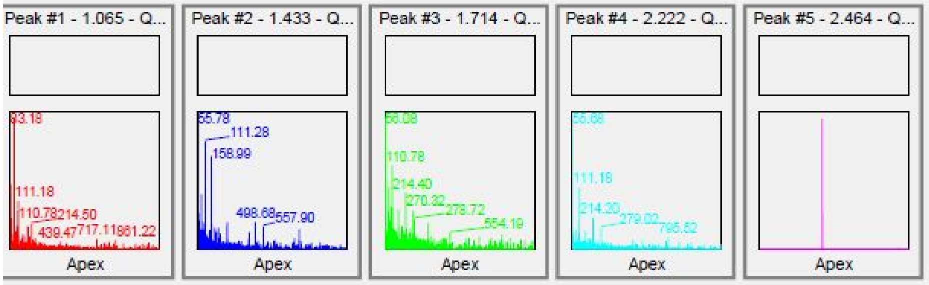

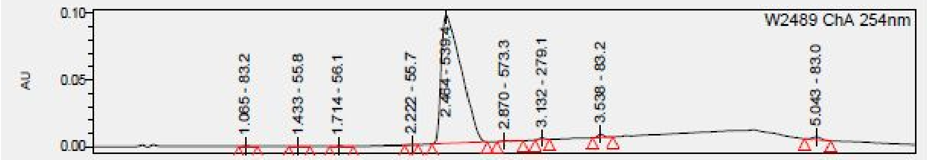

^{1}H NMR, ^{13}C NMR, ^{31}P NMR, HPLC-MS chromatogram and HRMS of compound **7**.

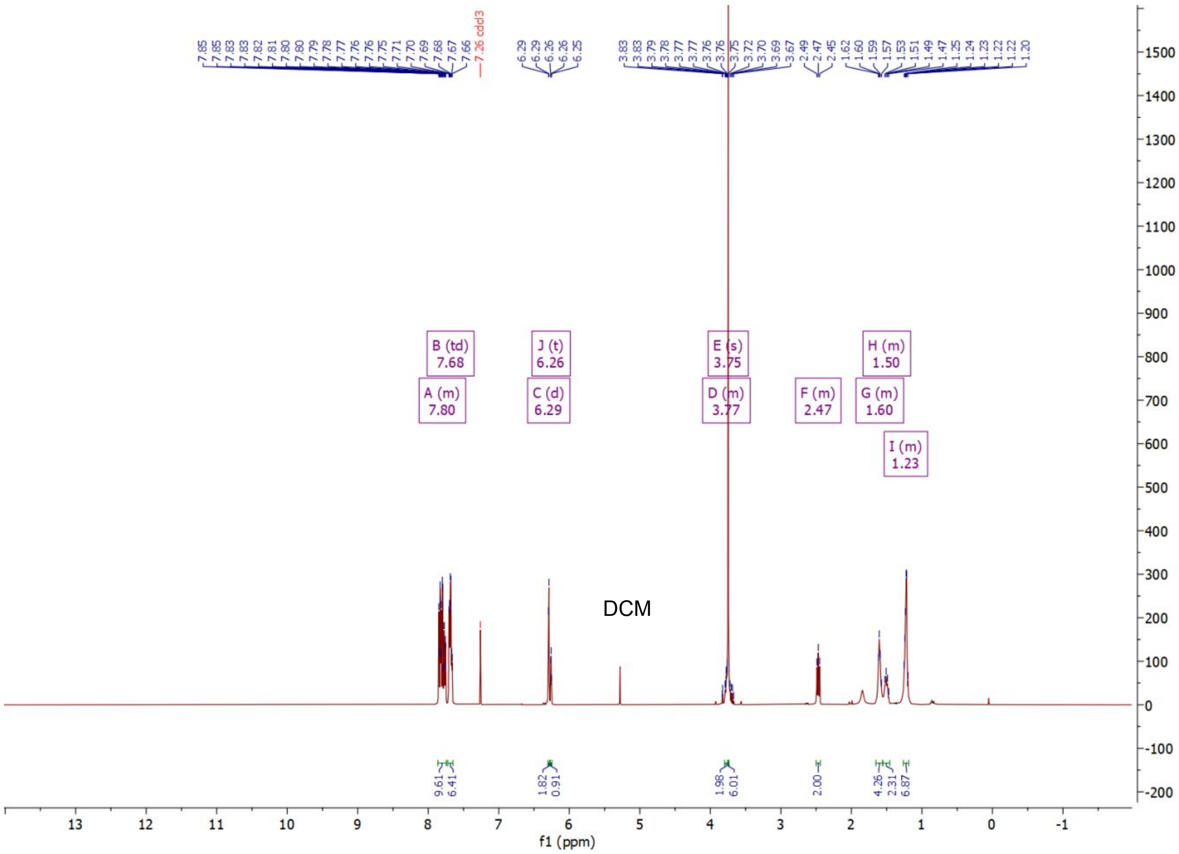

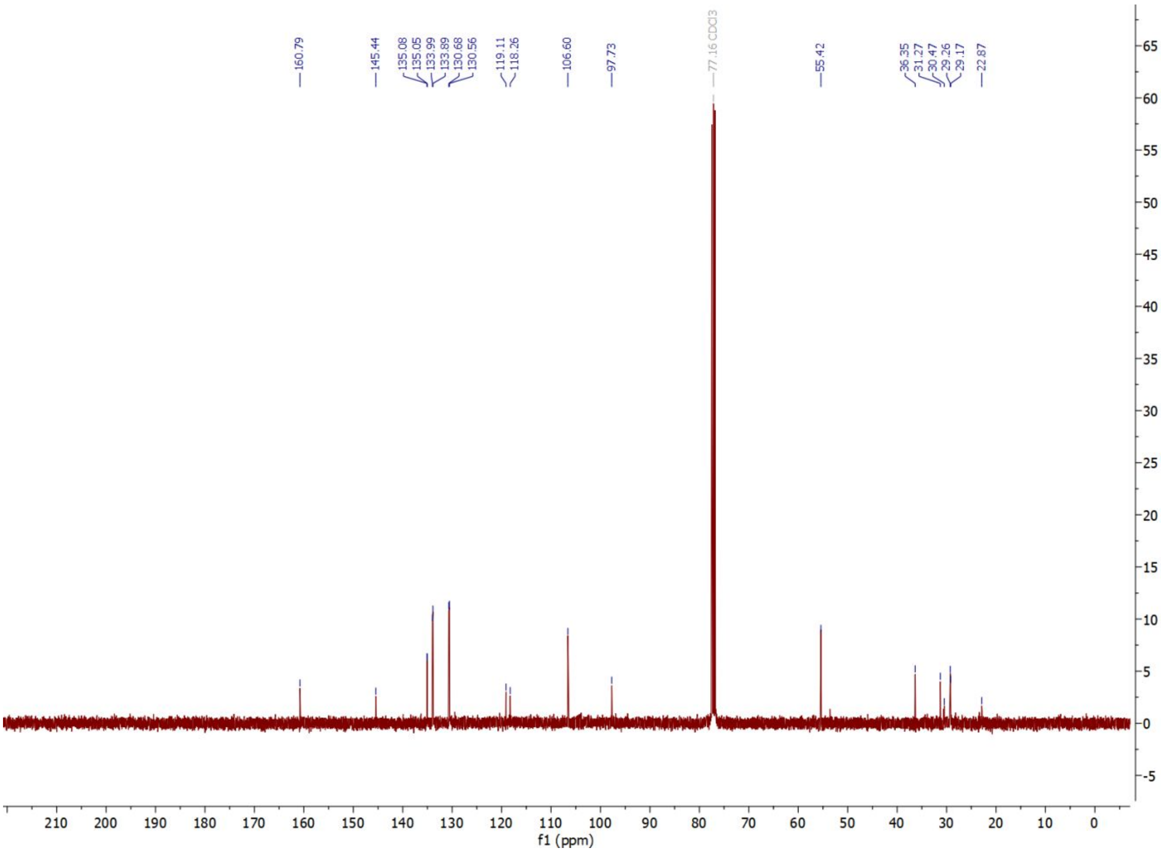

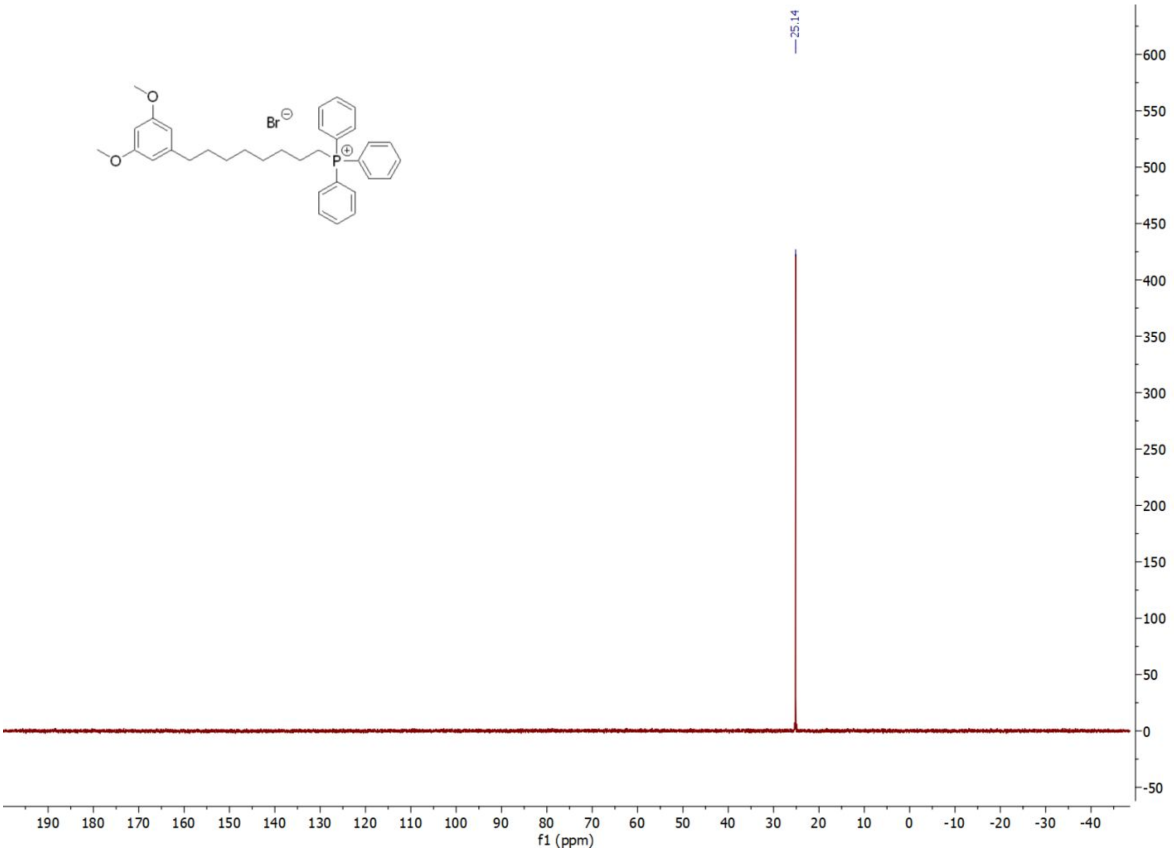

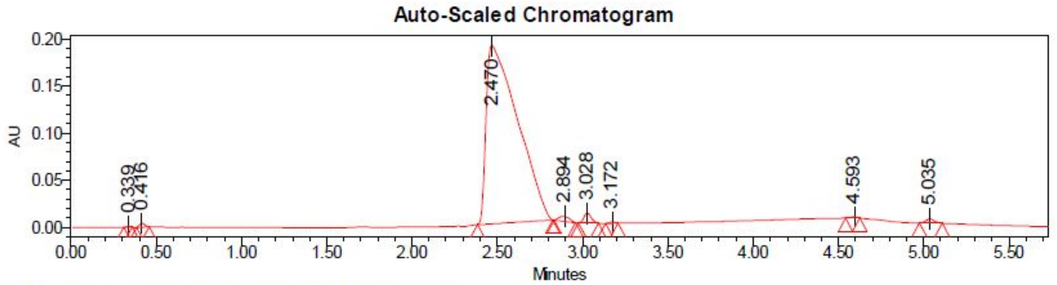

Processed Channel: W2489 ChA 254nm

	Processed Channel	Retention Time (min)	Area	% Area	Height
1	W2489 ChA 254nm	0.339	1634	0.06	1015
2	W2489 ChA 254nm	0.416	9895	0.39	4194
3	W2489 ChA 254nm	2.470	2431145	96.53	190404
4	W2489 ChA 254nm	2.894	22716	0.90	5063
5	W2489 ChA 254nm	3.028	31979	1.27	10592
6	W2489 ChA 254nm	3.172	2941	0.12	1261
7	W2489 ChA 254nm	4.593	2700	0.11	1149
8	W2489 ChA 254nm	5.035	15650	0.62	4607

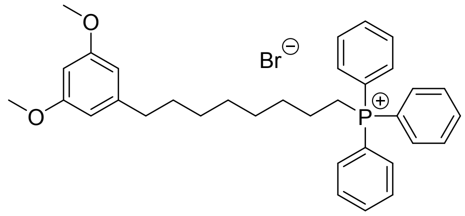

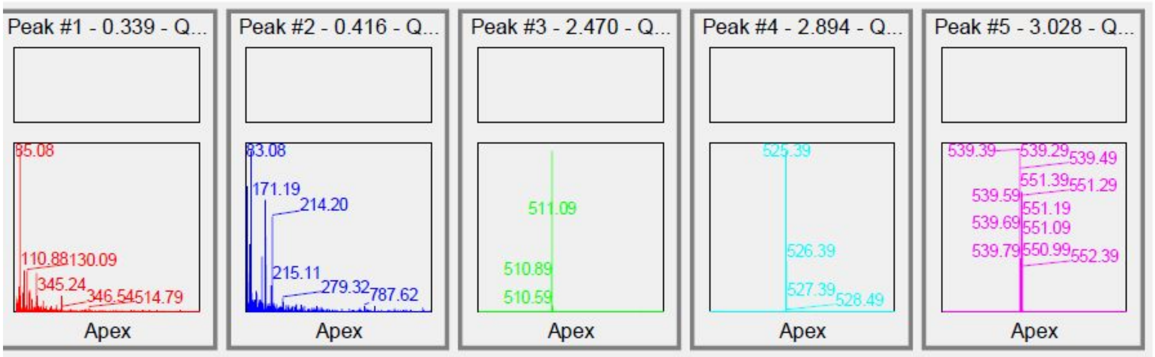

Item name: MB7_0.1ul
Item description:
Component name: MB7-Br

Channel name: Time 0.0646 +/- 0.0212 minutes

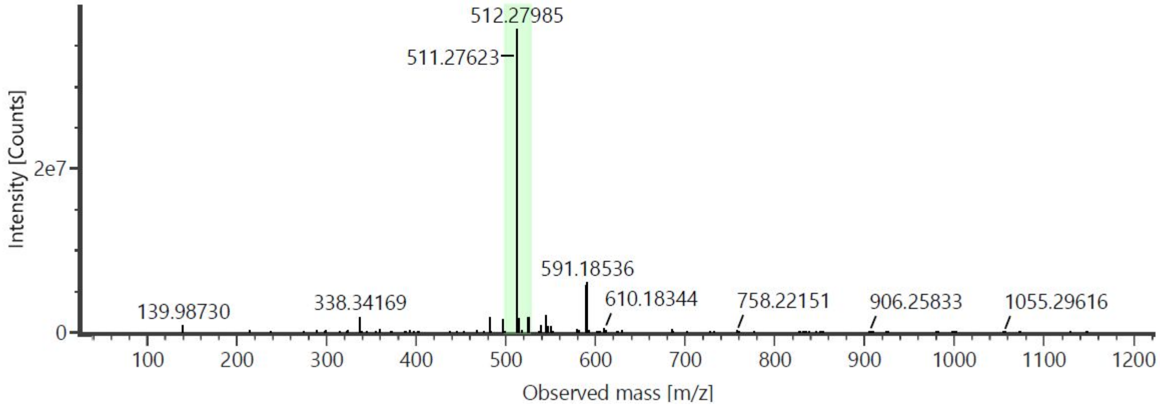

^{1}H NMR, ^{13}C NMR, ^{31}P NMR and HPLC-MS chromatogram of compound **8**.

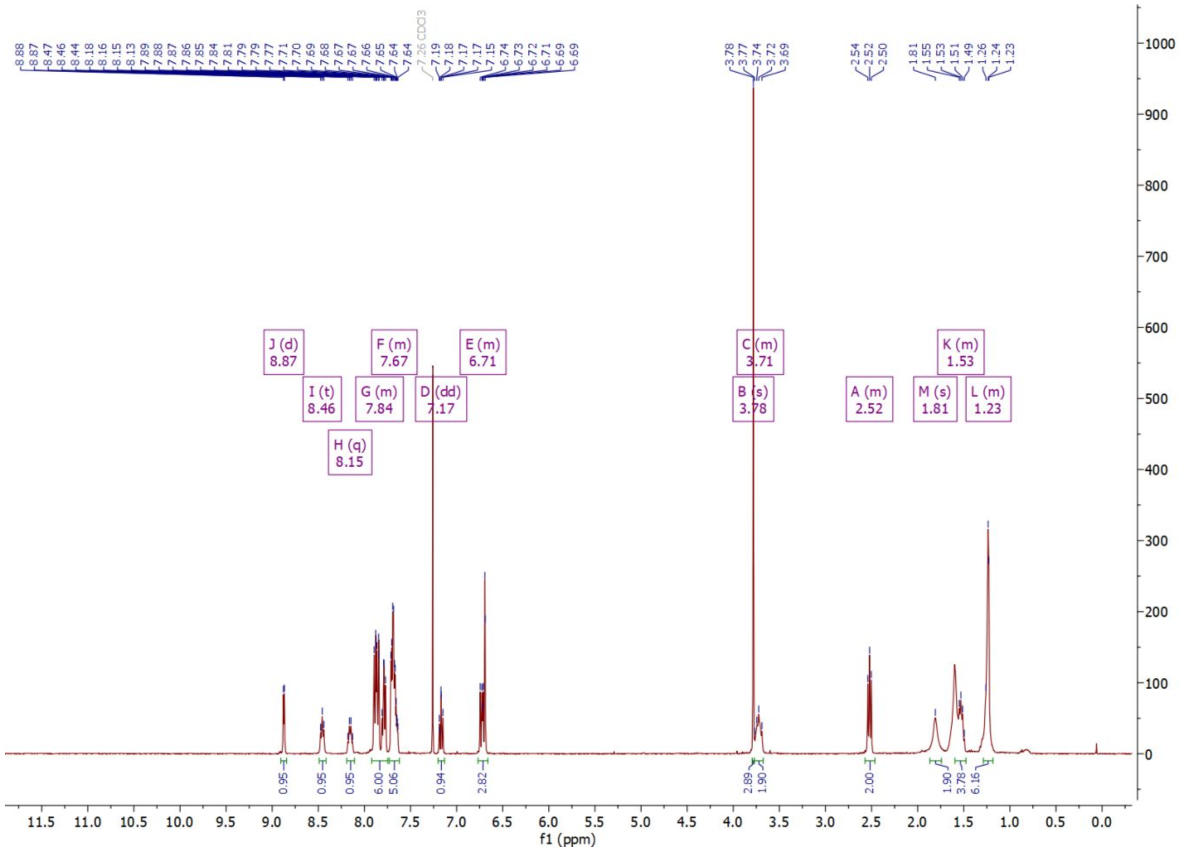

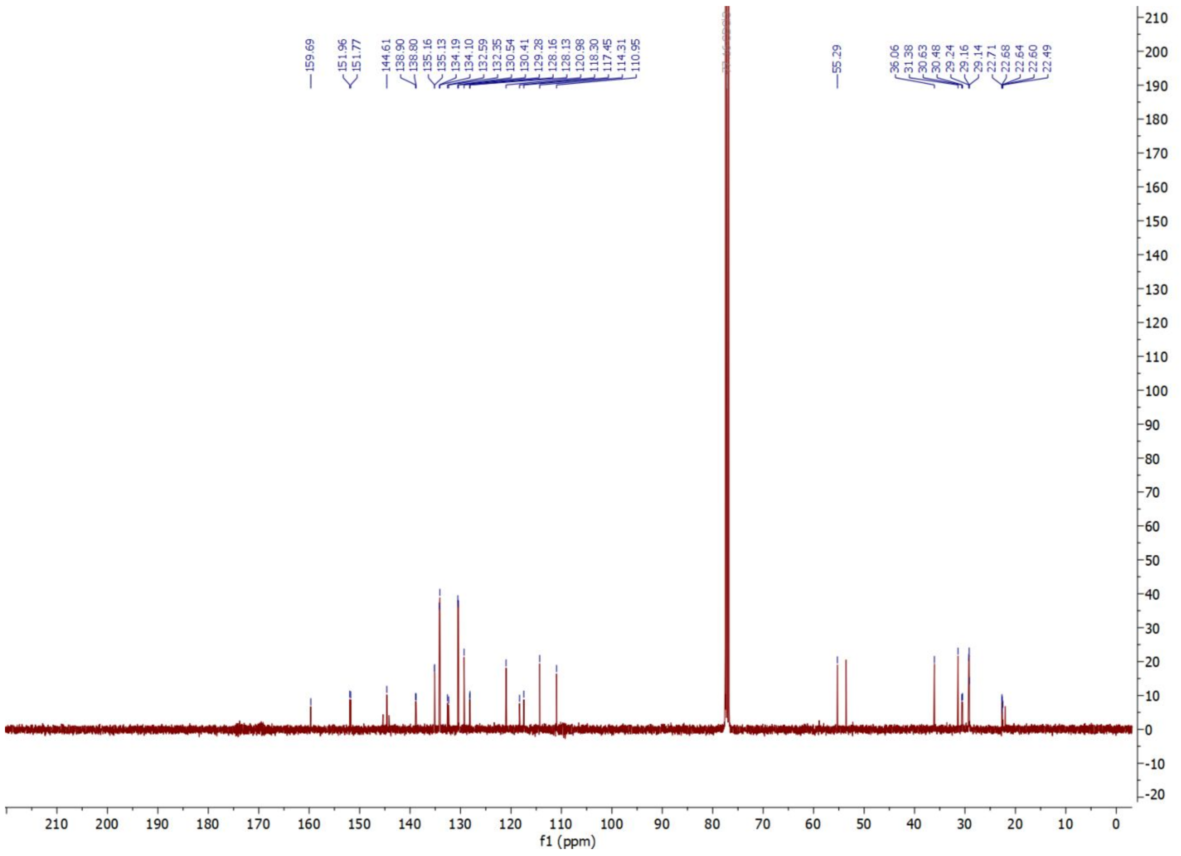

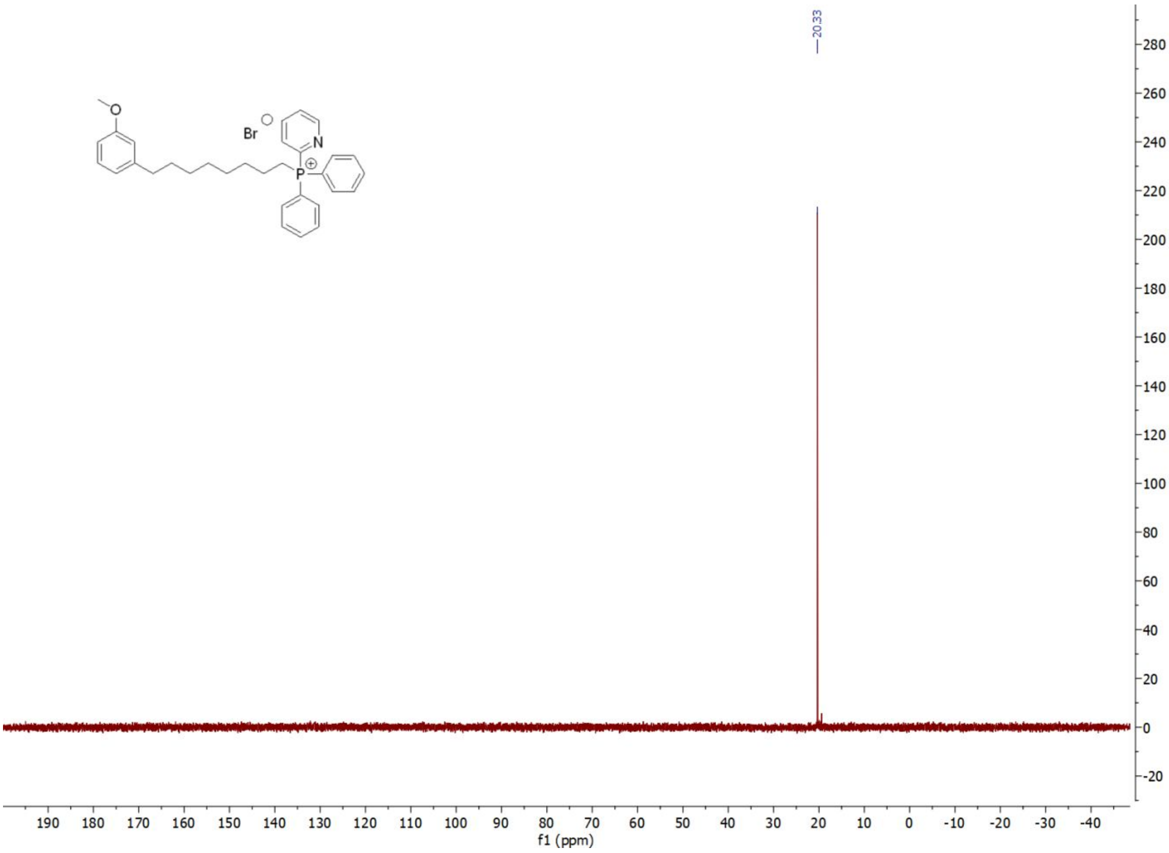

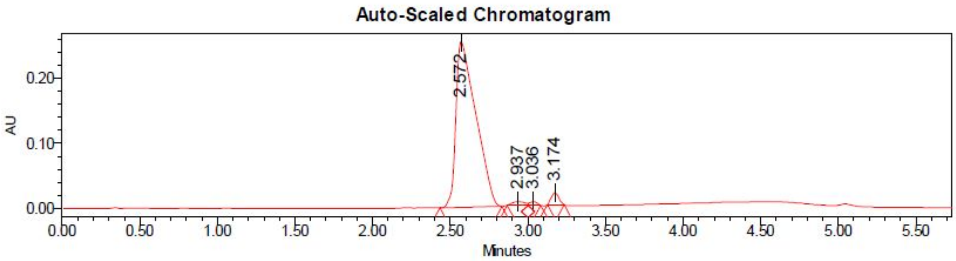

Processed Channel: W2489 ChA 254nm

	Processed Channel	Retention Time (min)	Area	% Area	Height
1	W2489 ChA 254nm	2.572	2263038	95.31	254888
2	W2489 ChA 254nm	2.937	31894	1.34	5926
3	W2489 ChA 254nm	3.036	18749	0.79	5879
4	W2489 ChA 254nm	3.174	60806	2.56	18174

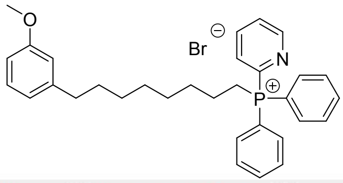

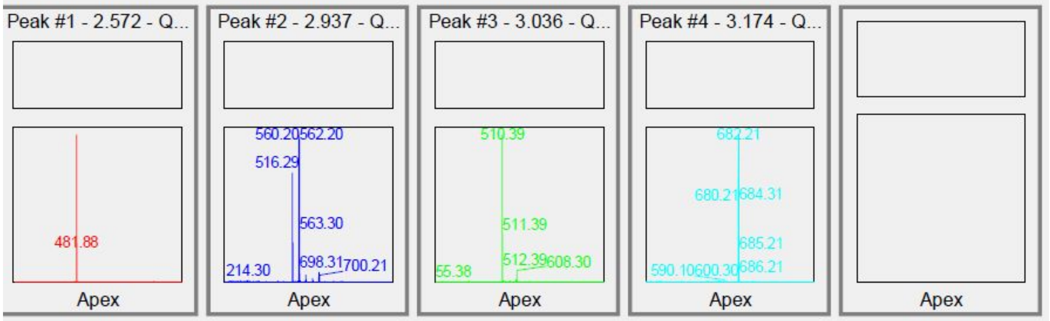

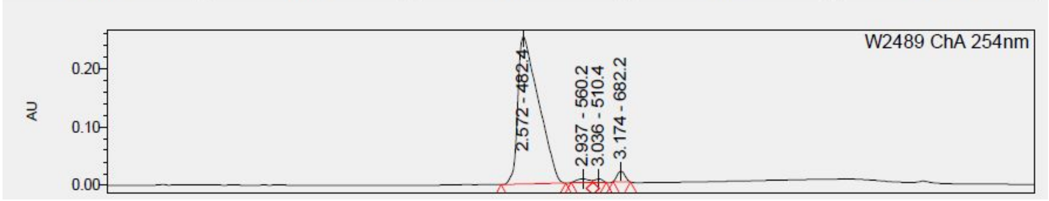

^{1}H NMR, ^{13}C NMR, ^{31}P NMR and HPLC-MS chromatogram of compound **9**.

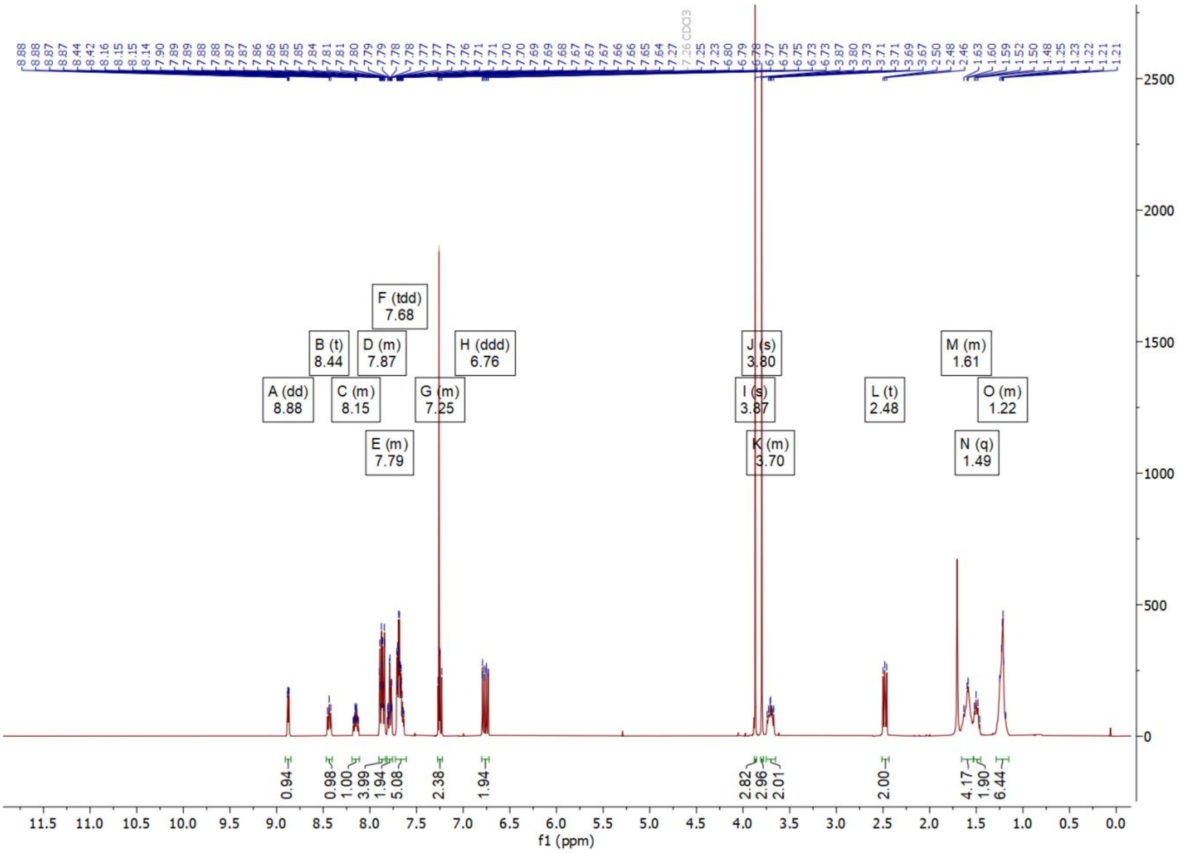

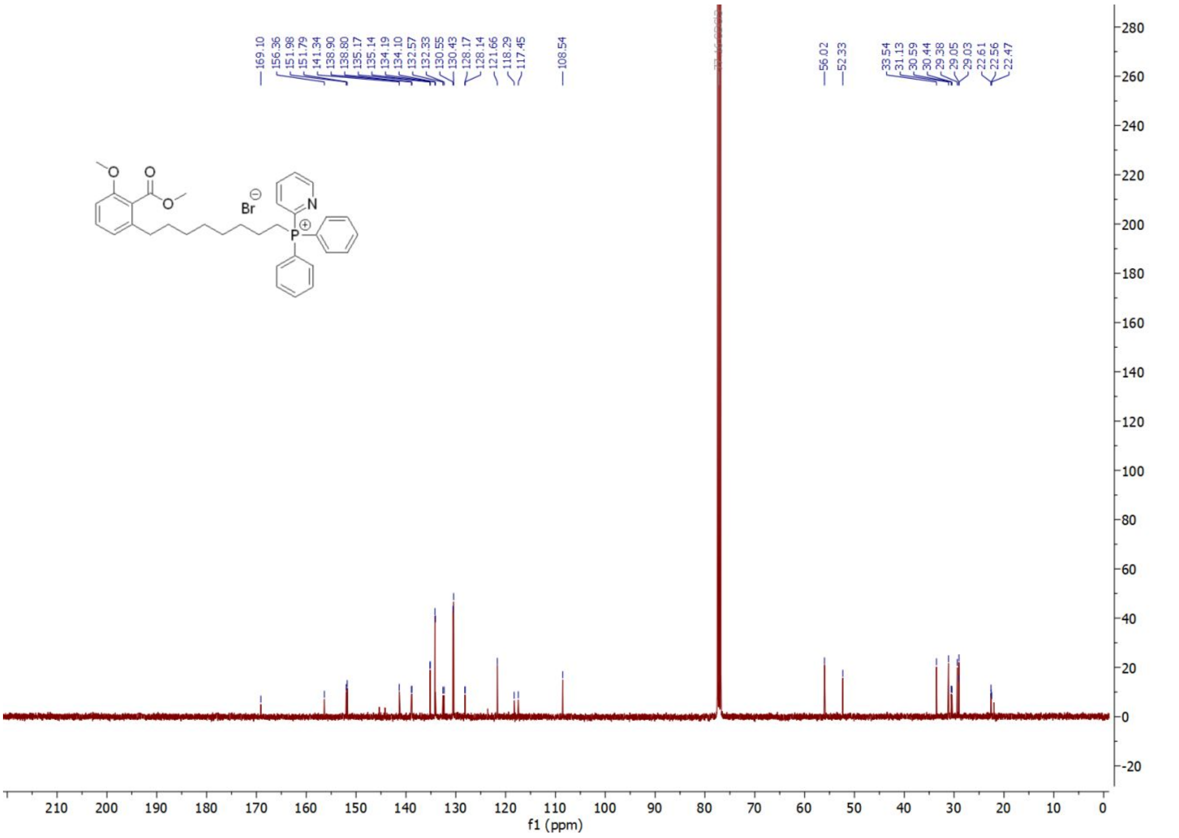

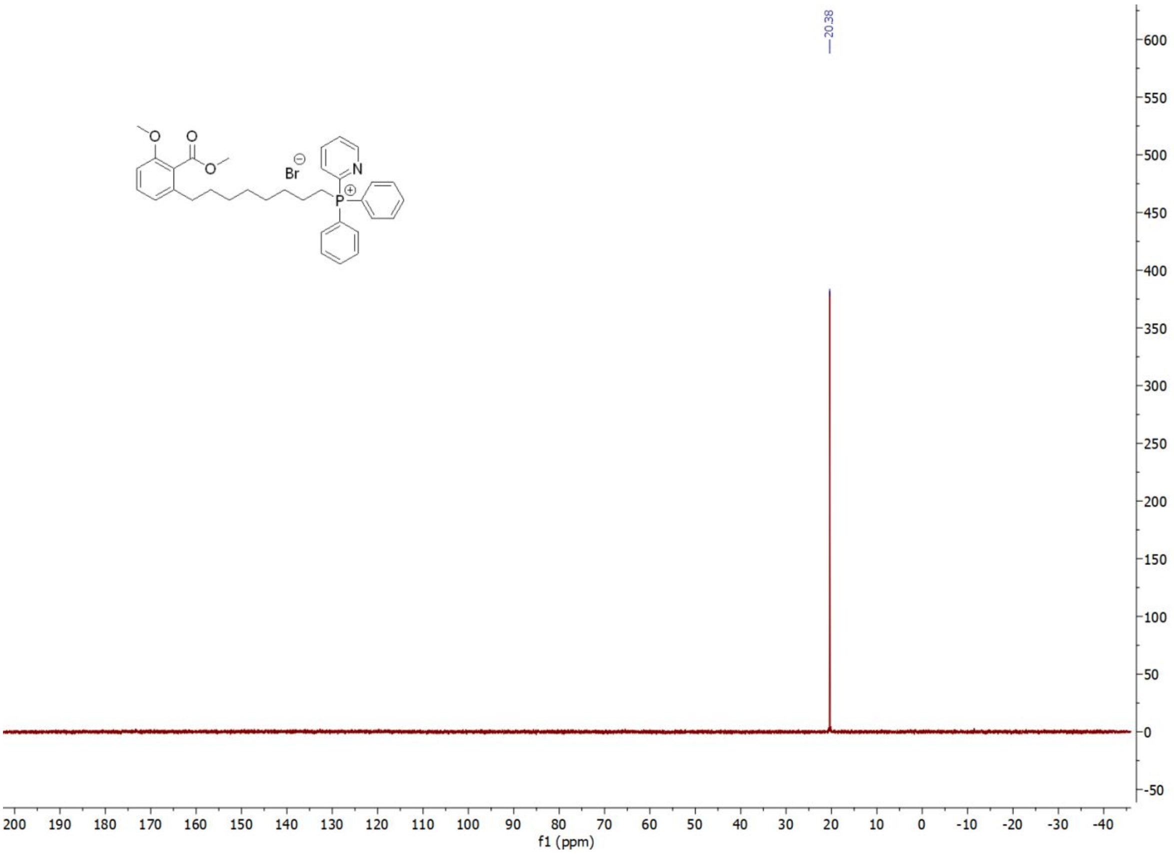

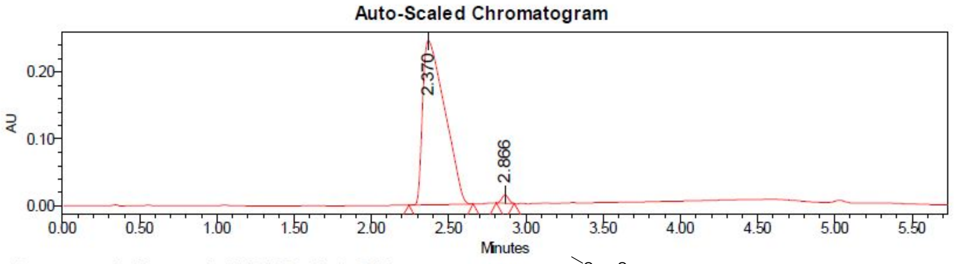

Processed Channel: W2489 ChA 254nm

	Processed Channel	Retention Time (min)	Area	% Area	Height
1	W2489 ChA 254nm	2.370	2453981	98.47	246679
2	W2489 ChA 254nm	2.866	38186	1.53	12653

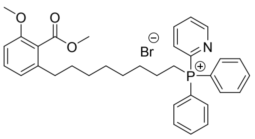

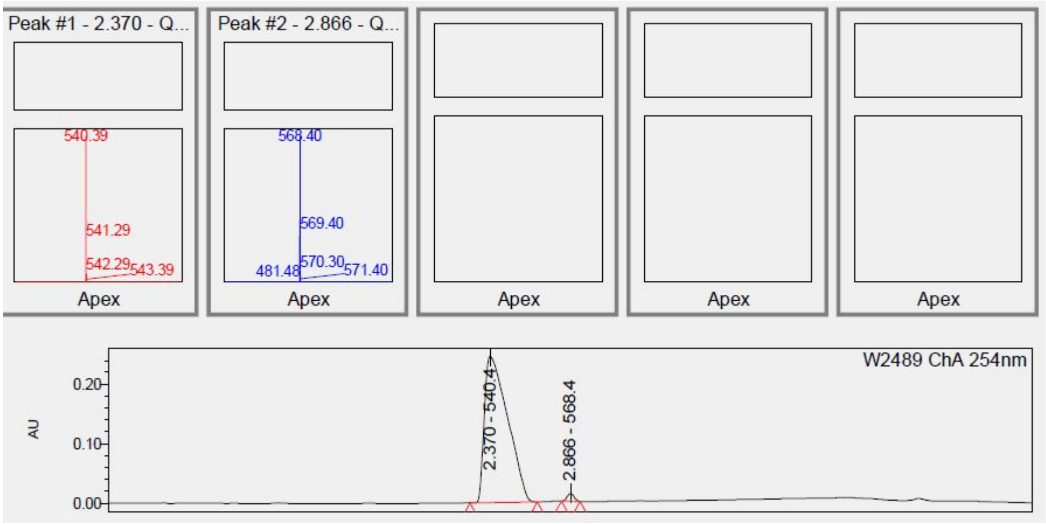

^{1}H NMR, ^{13}C NMR, ^{31}P NMR and HPLC-MS chromatogram of compound **10**.

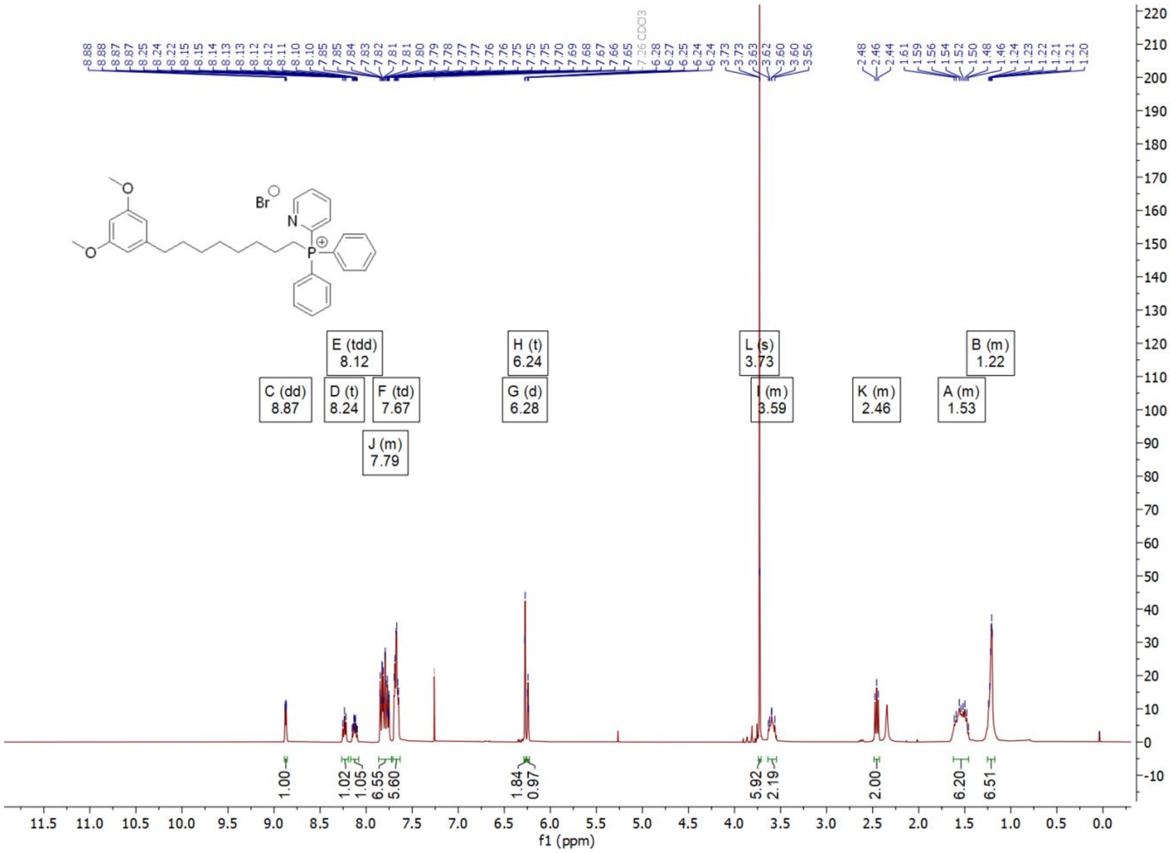

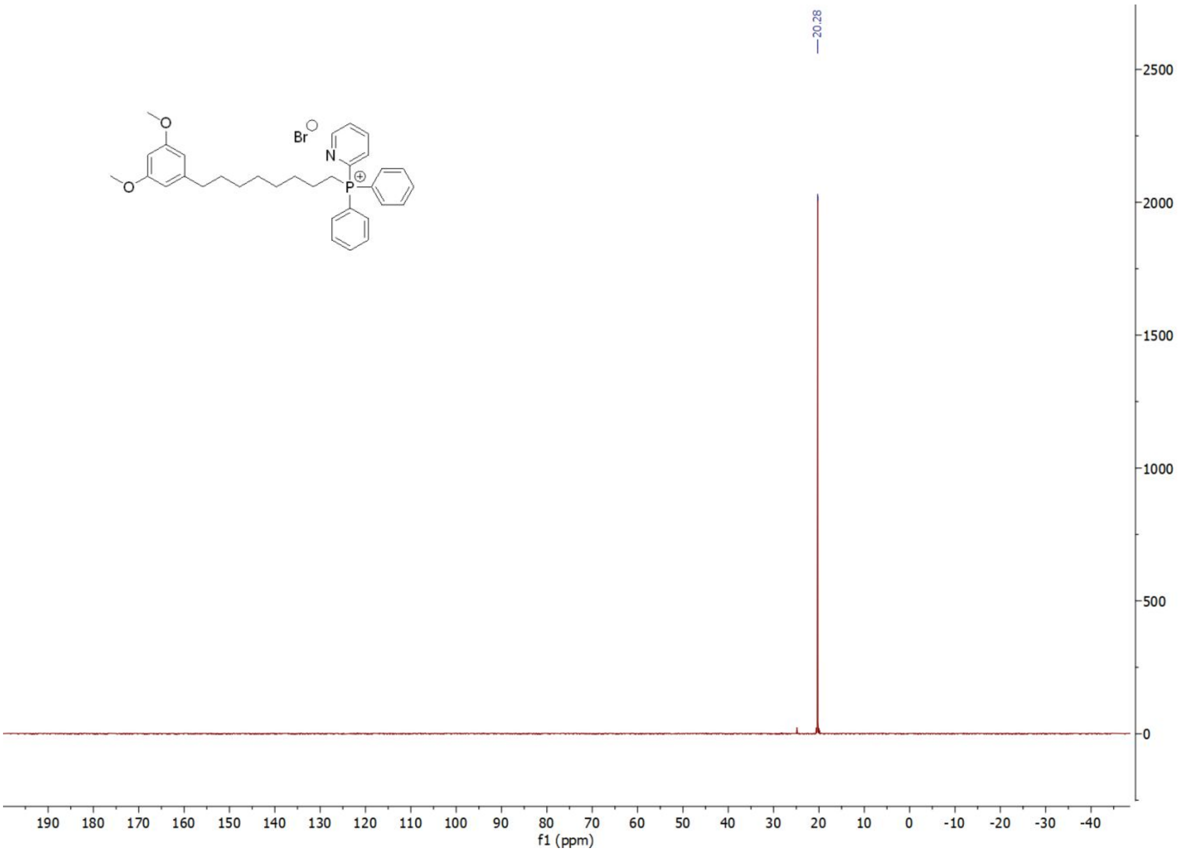

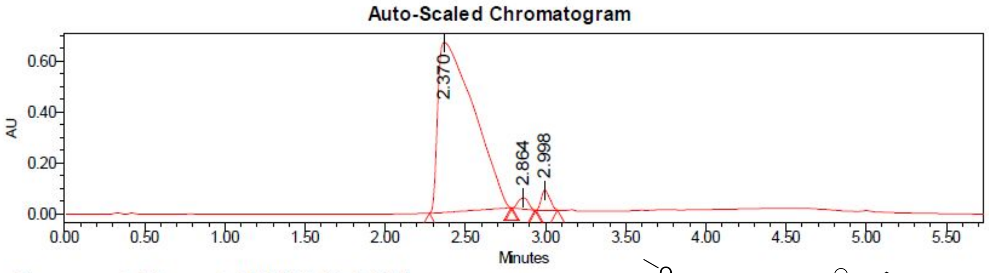

Processed Channel: W2489 ChA 254nm

	Processed Channel	Retention Time (min)	Area	% Area	Height
1	W2489 ChA 254nm	2.370	10433423	95.62	667377
2	W2489 ChA 254nm	2.864	194017	1.78	43374
3	W2489 ChA 254nm	2.998	284050	2.60	77113

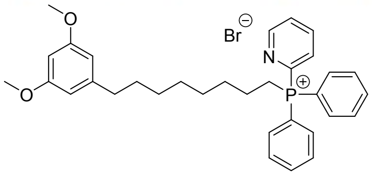

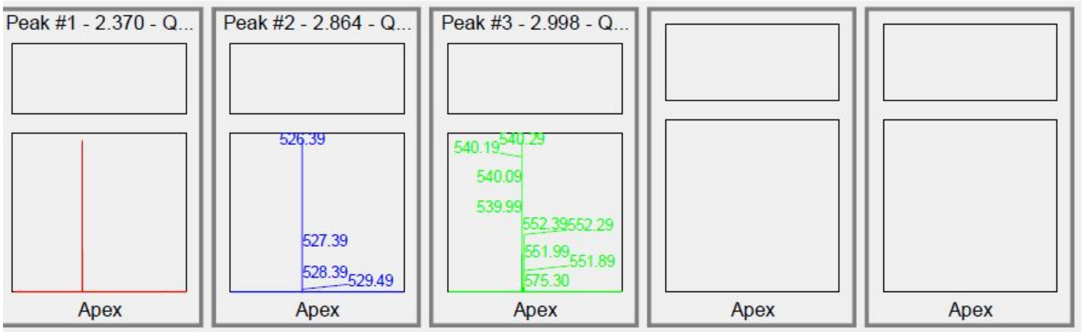

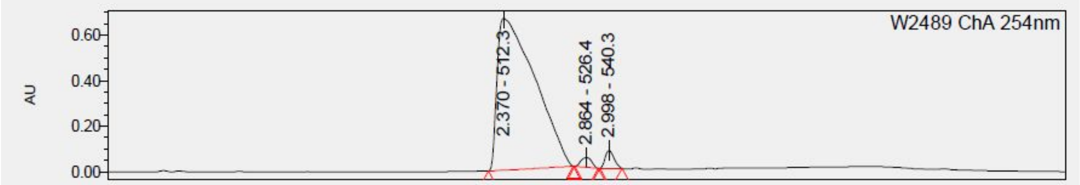

^{1}H NMR, ^{13}C NMR, ^{31}P NMR and HPLC-MS chromatogram of compound **11**.

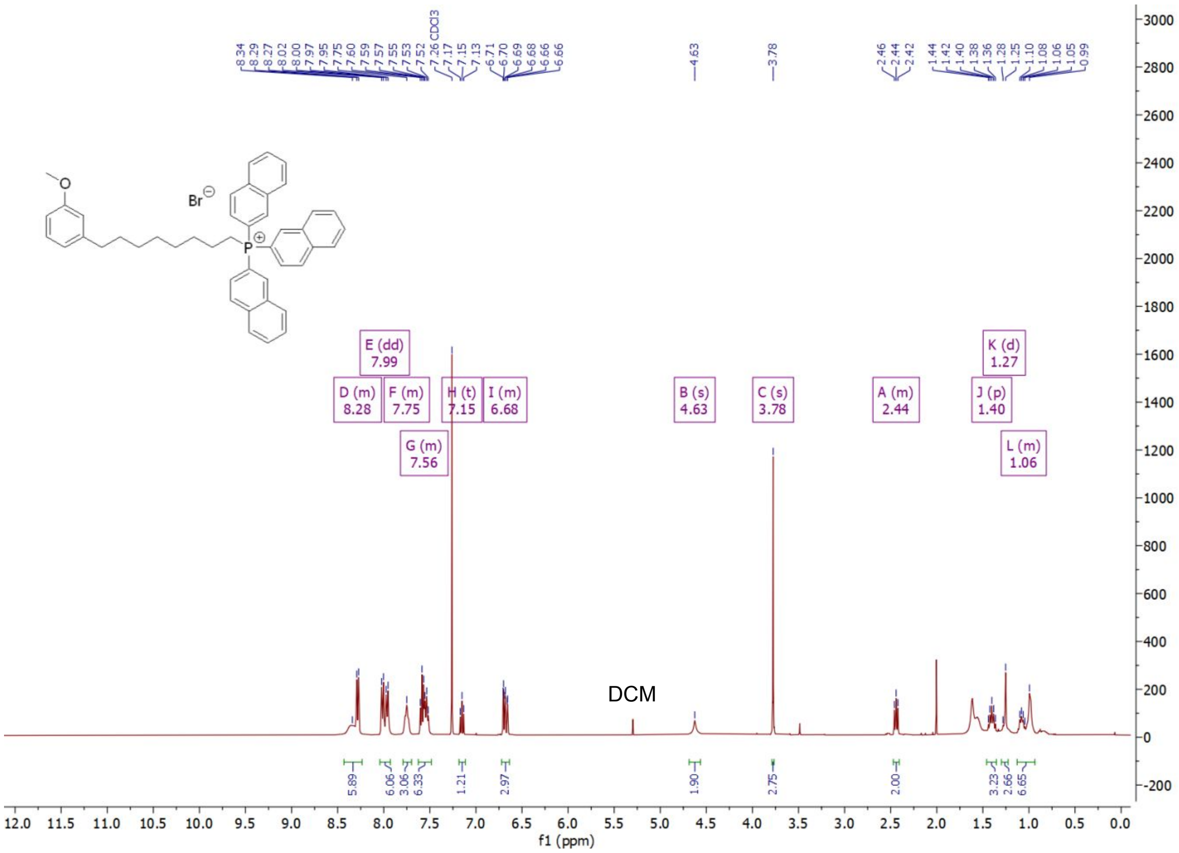

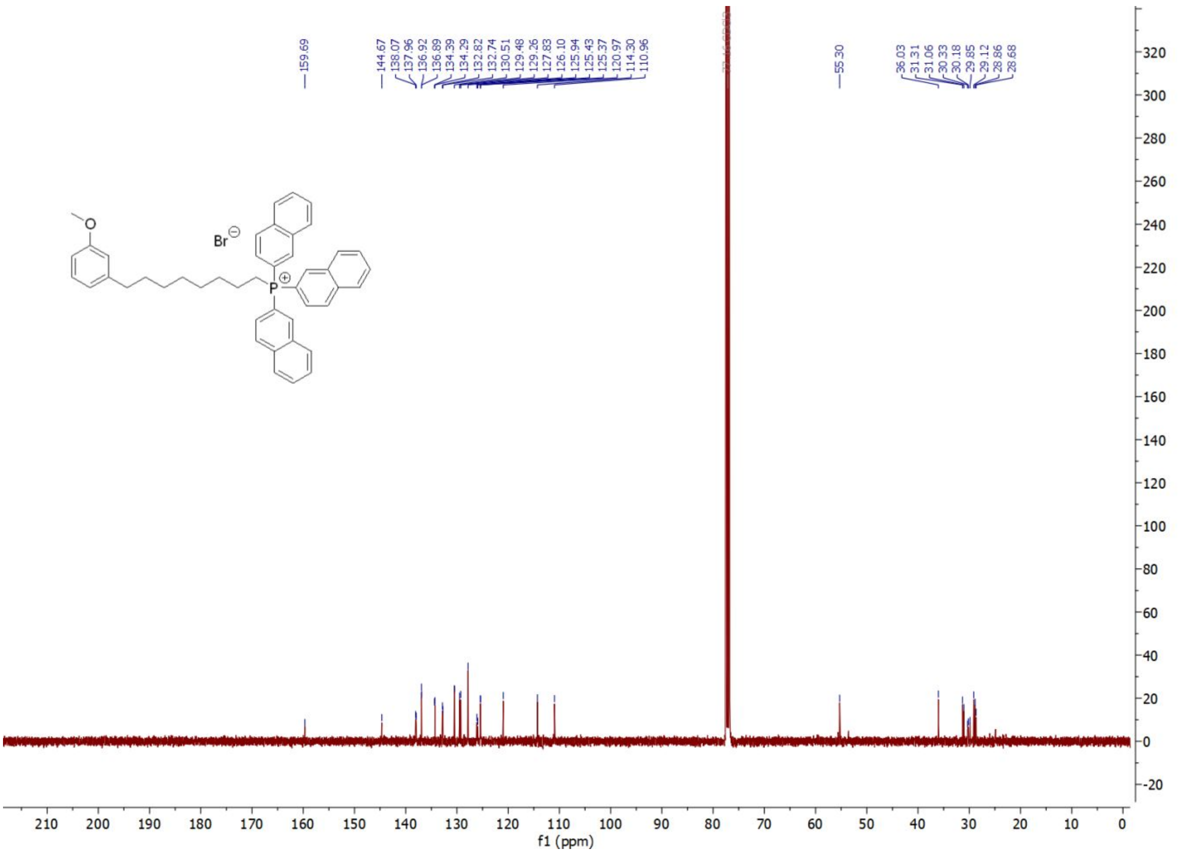

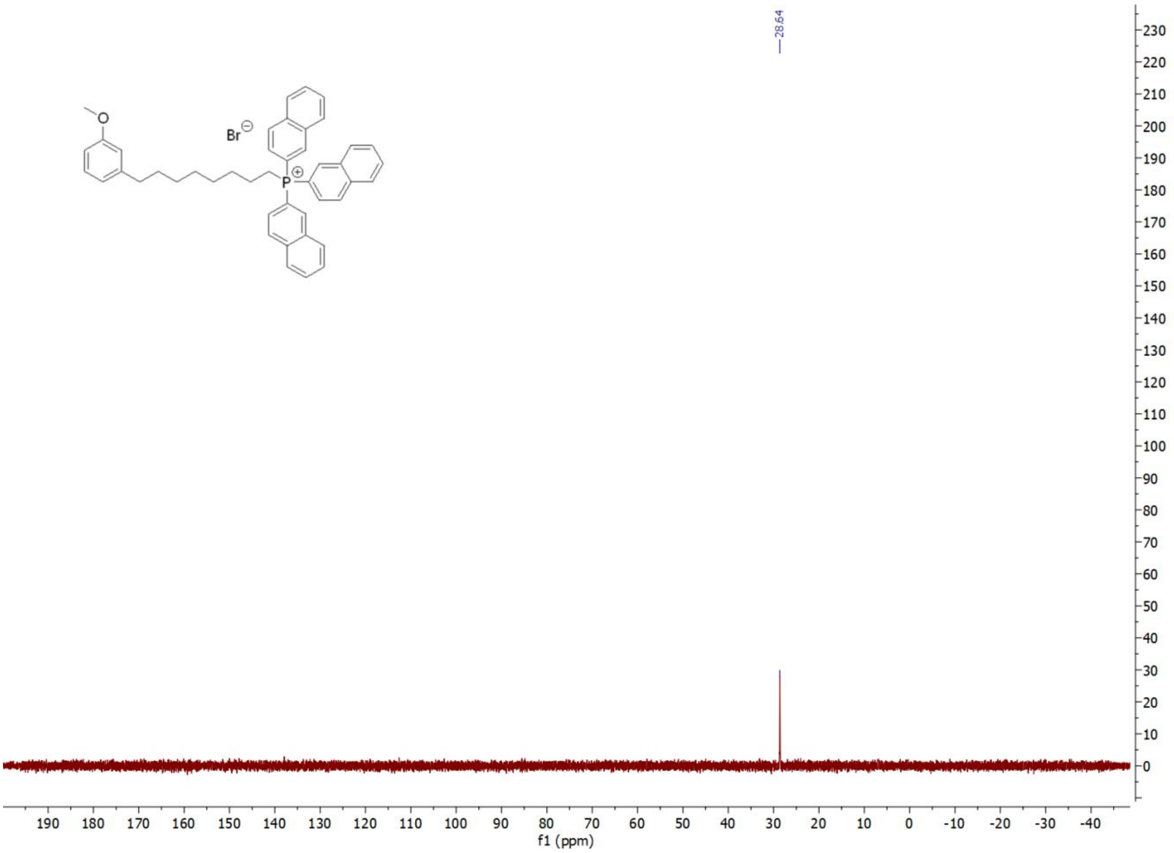

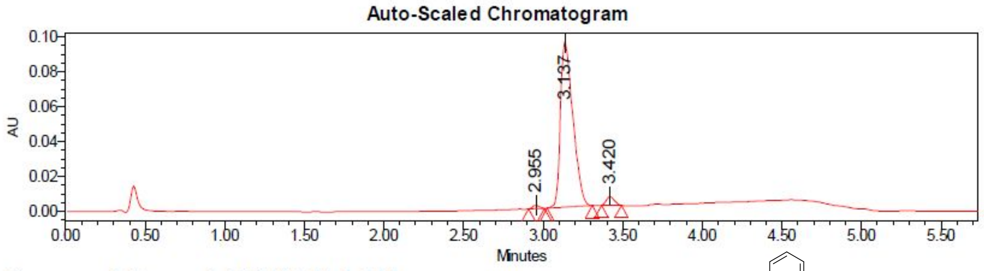

Processed Channel: W2489 ChA 254nm

	Processed Channel	Retention Time (min)	Area	% Area	Height
1	W2489 ChA 254nm	2.955	5408	1.01	1933
2	W2489 ChA 254nm	3.137	510511	95.75	95330
3	W2489 ChA 254nm	3.420	17258	3.24	4968

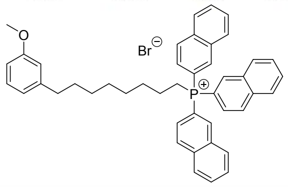

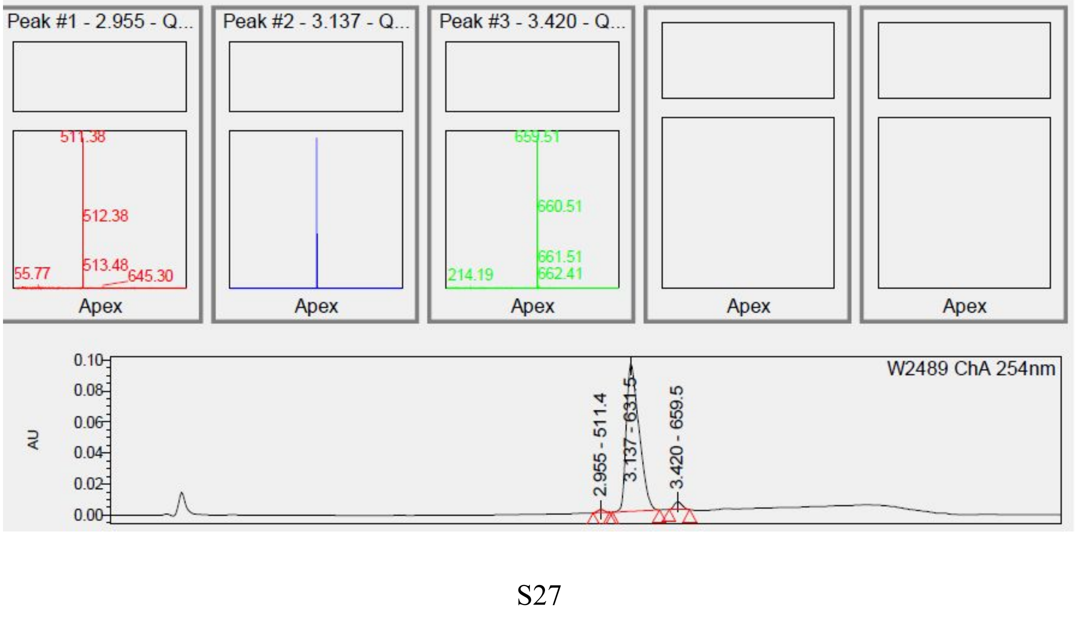

^{1}H NMR, ^{13}C NMR, ^{31}P NMR and HPLC-MS chromatogram of compound **12**.

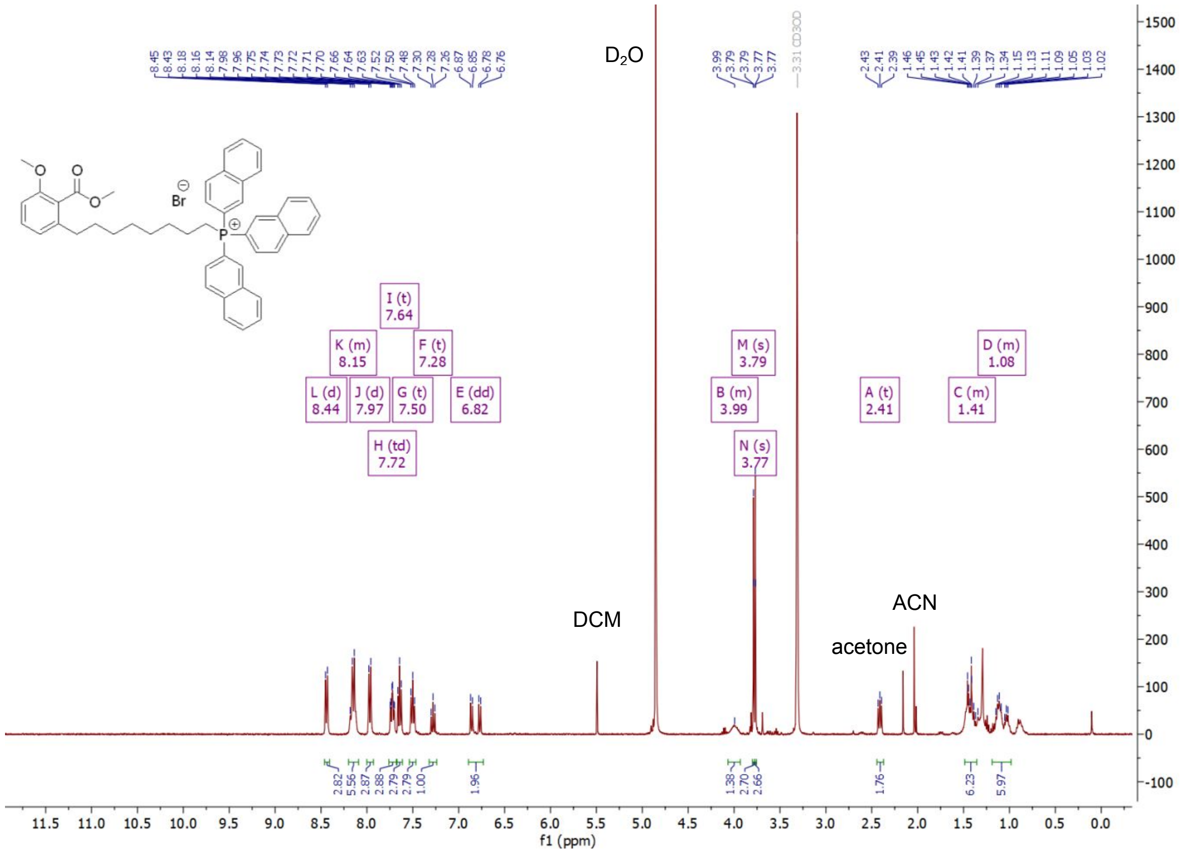

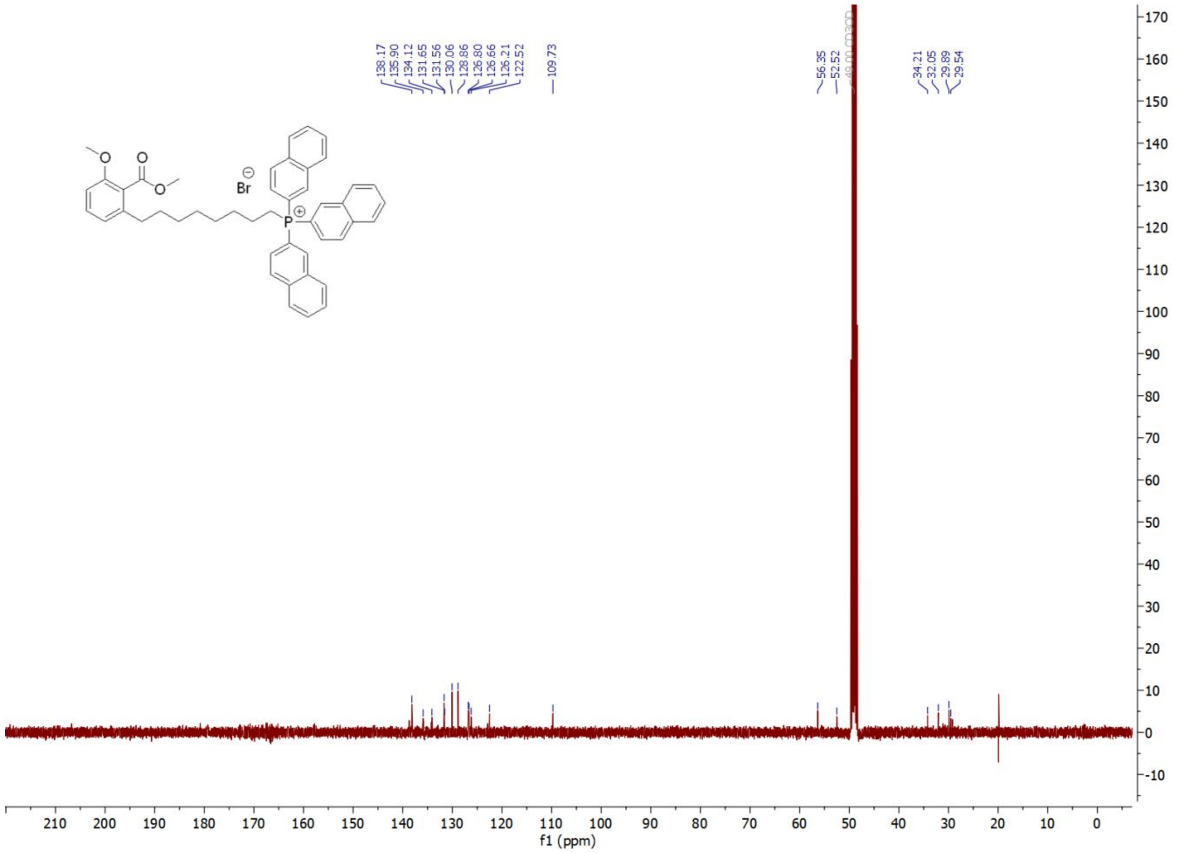

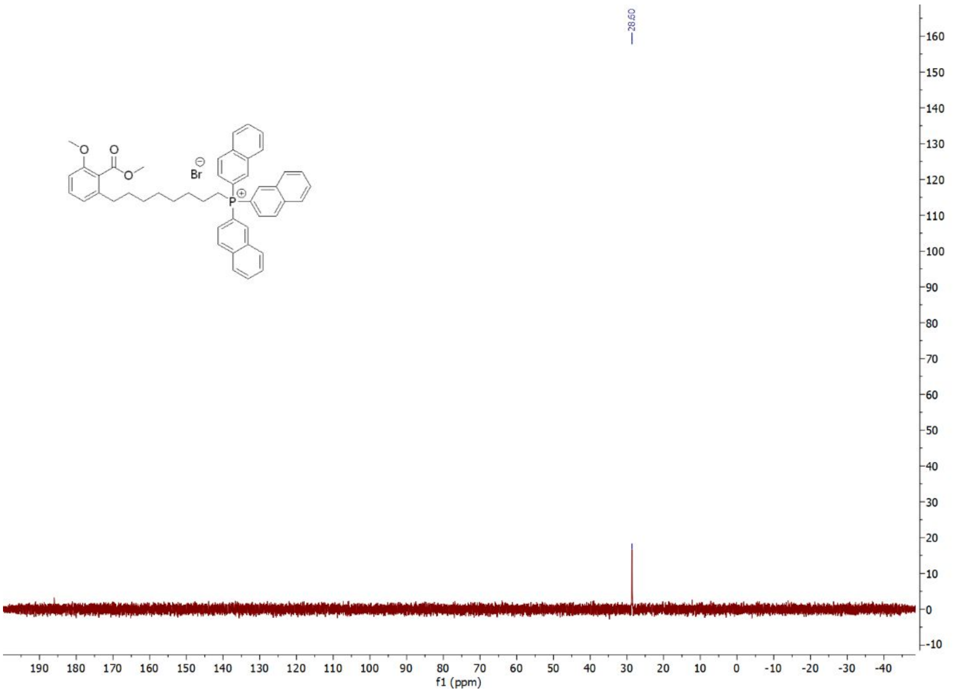

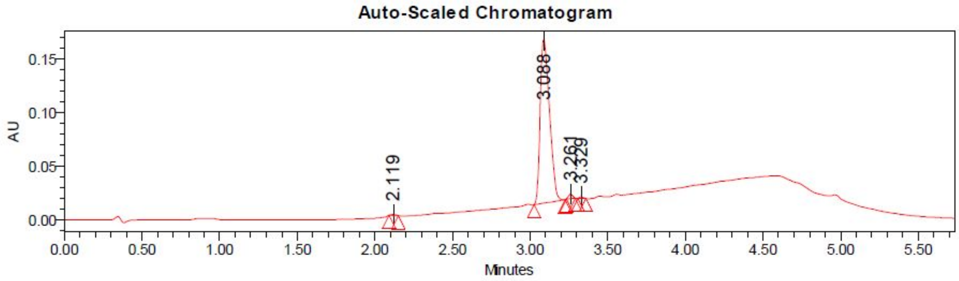

Peak Results

	Name	RT	Area	Height	% Area
1		2.119	1784	811	0.28
2		3.088	635247	154430	98.06
3		3.261	8306	4186	1.28
4		3.329	2506	1492	0.39

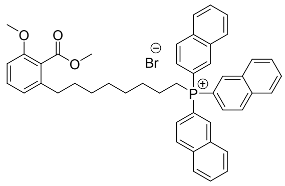

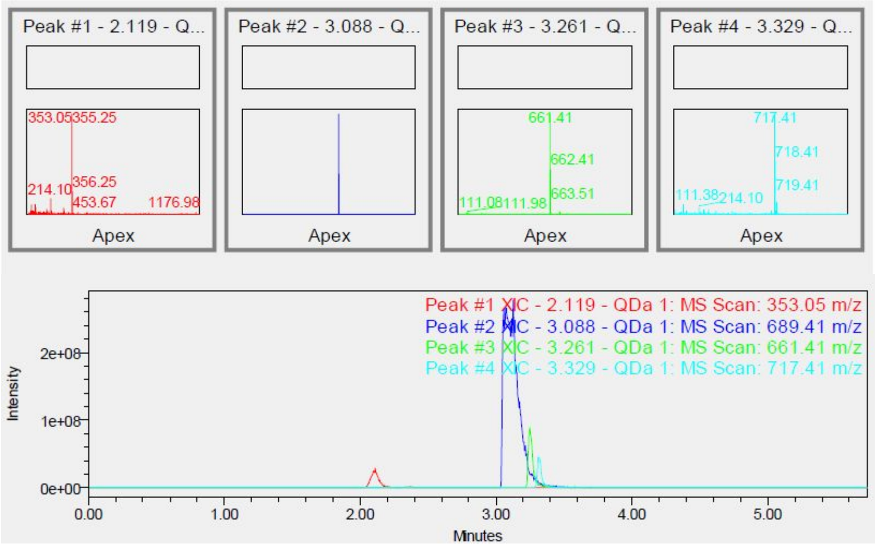

^{1}H NMR, ^{13}C NMR, ^{31}P NMR and HPLC-MS chromatogram of compound **13**.

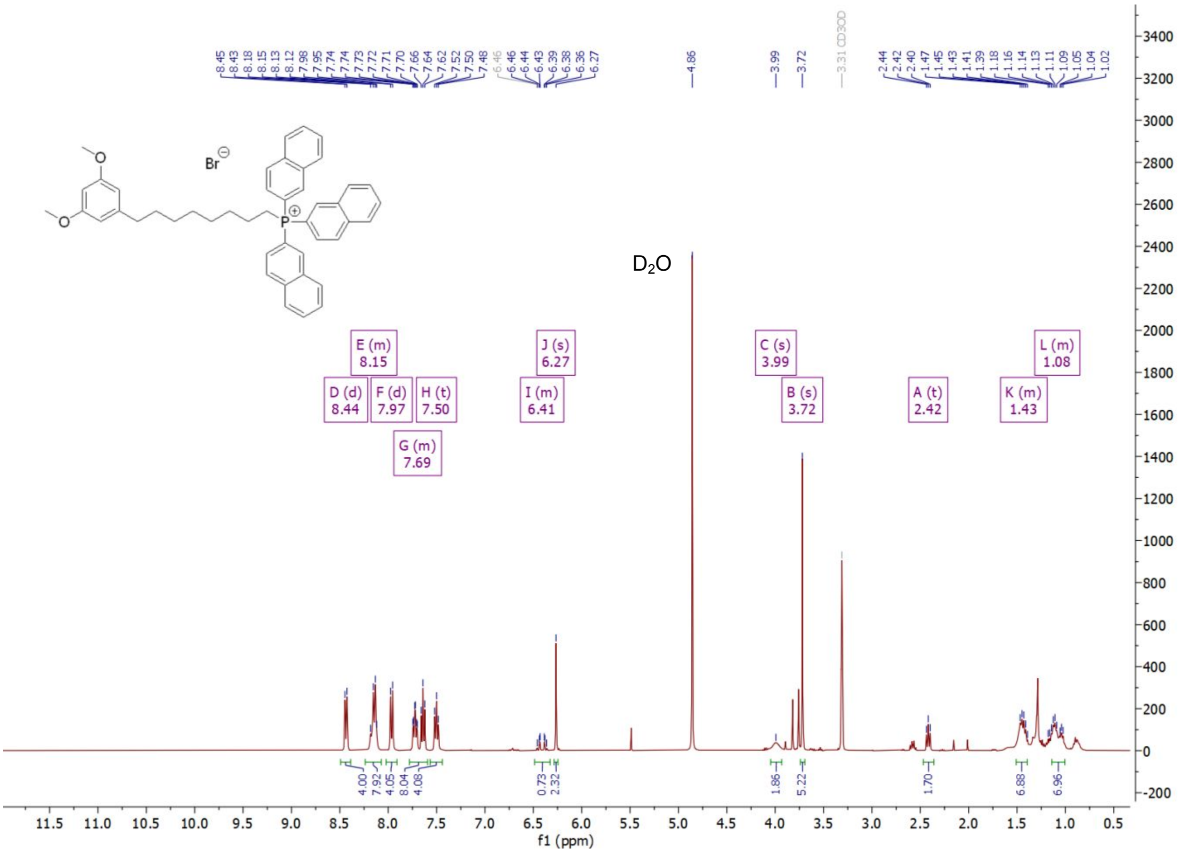

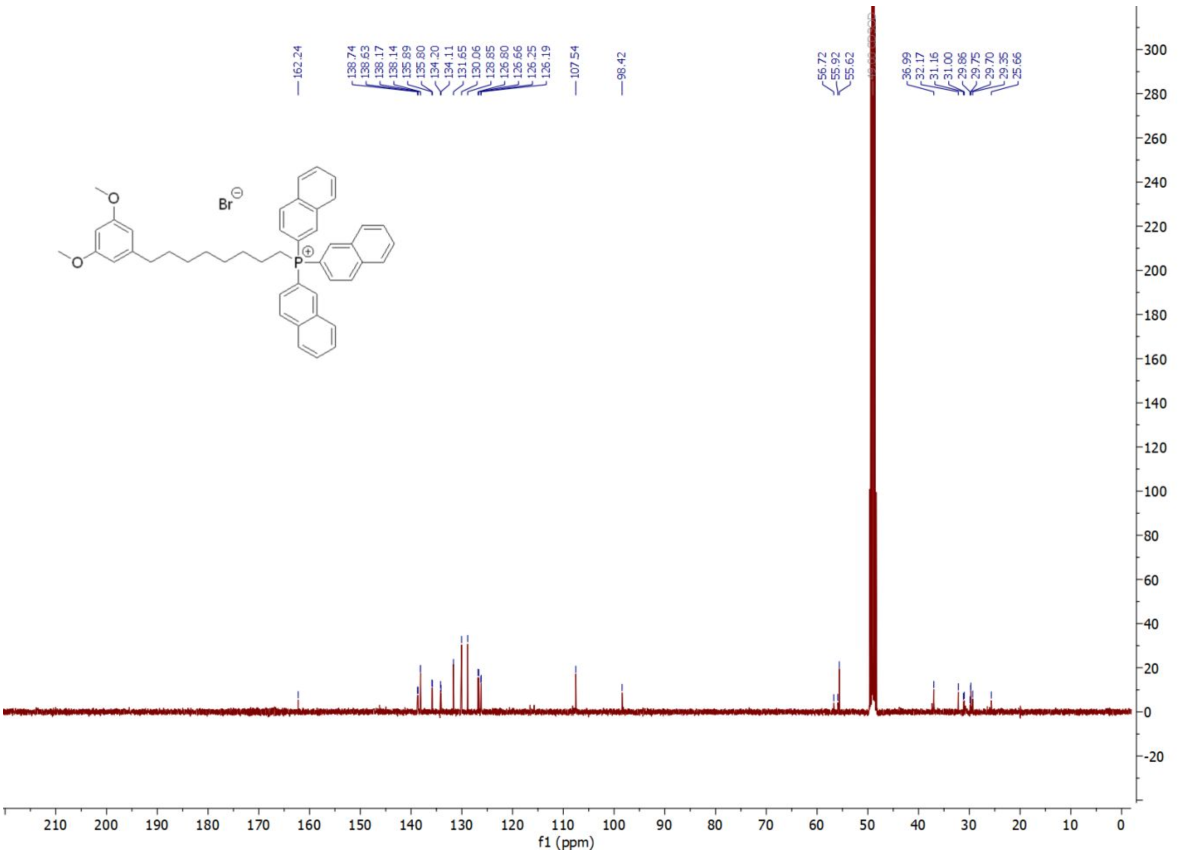

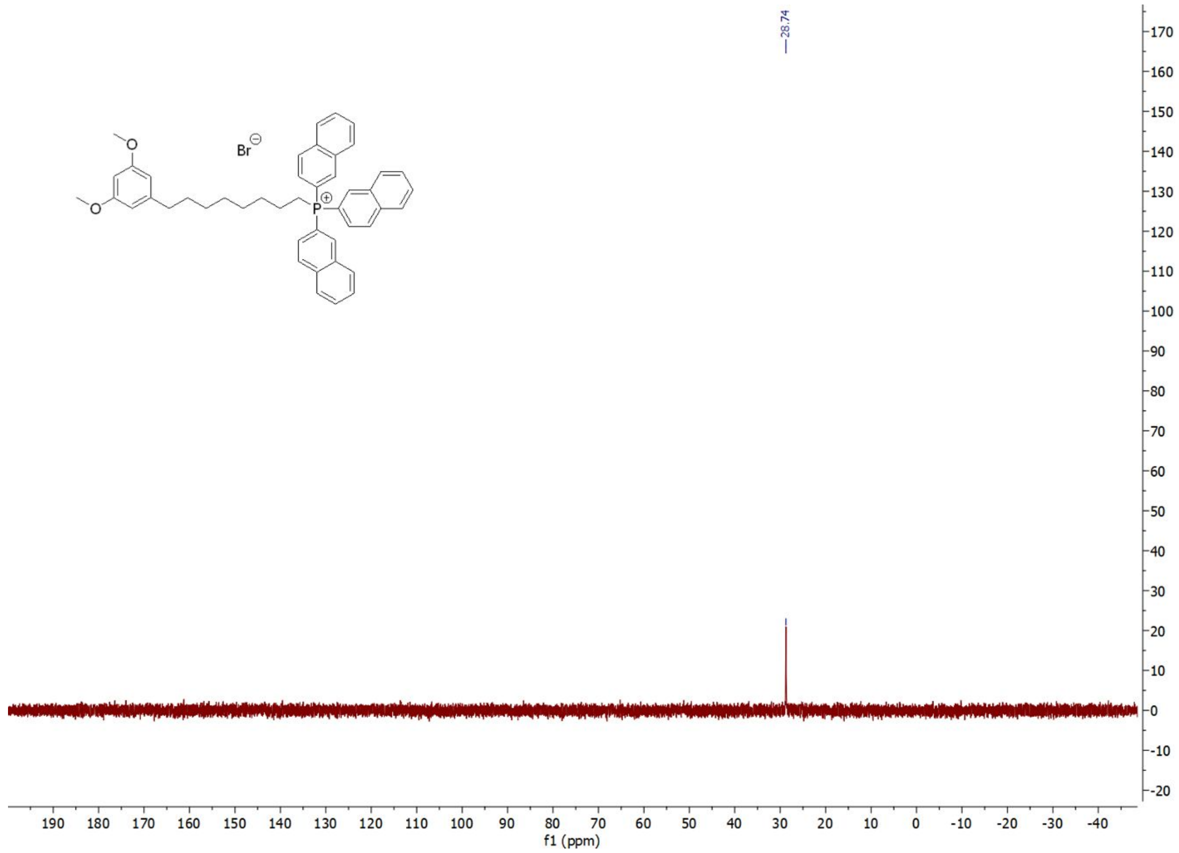

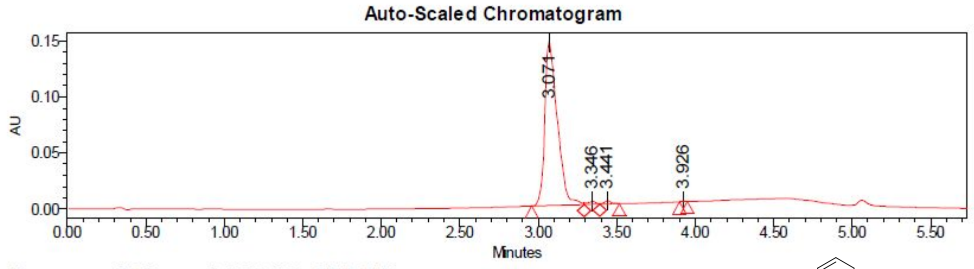

Processed Channel: W2489 ChA 254nm

	Processed Channel	Retention Time (min)	Area	% Area	Height
1	W2489 ChA 254nm	3.071	801833	97.60	147494
2	W2489 ChA 254nm	3.346	9860	1.20	2596
3	W2489 ChA 254nm	3.441	8793	1.07	2857
4	W2489 ChA 254nm	3.926	1066	0.13	610

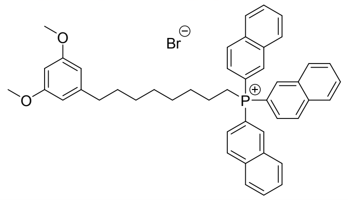

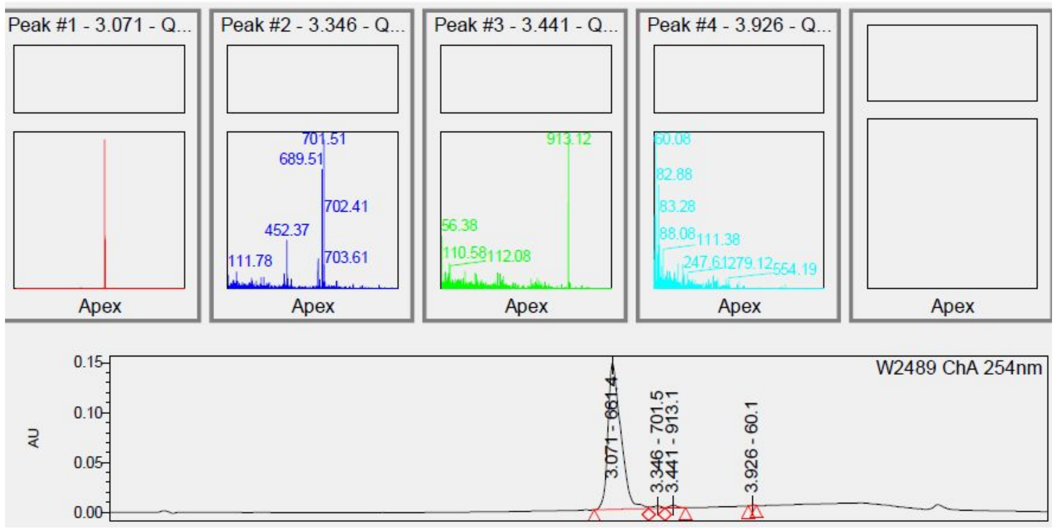

5. Spectral copies of ^{1}H, ^{13}C NMR and HPLC-MS of final compounds **14** – **25**.

^{1}H NMR, ^{13}C NMR and HPLC-MS chromatogram of compound **14**.

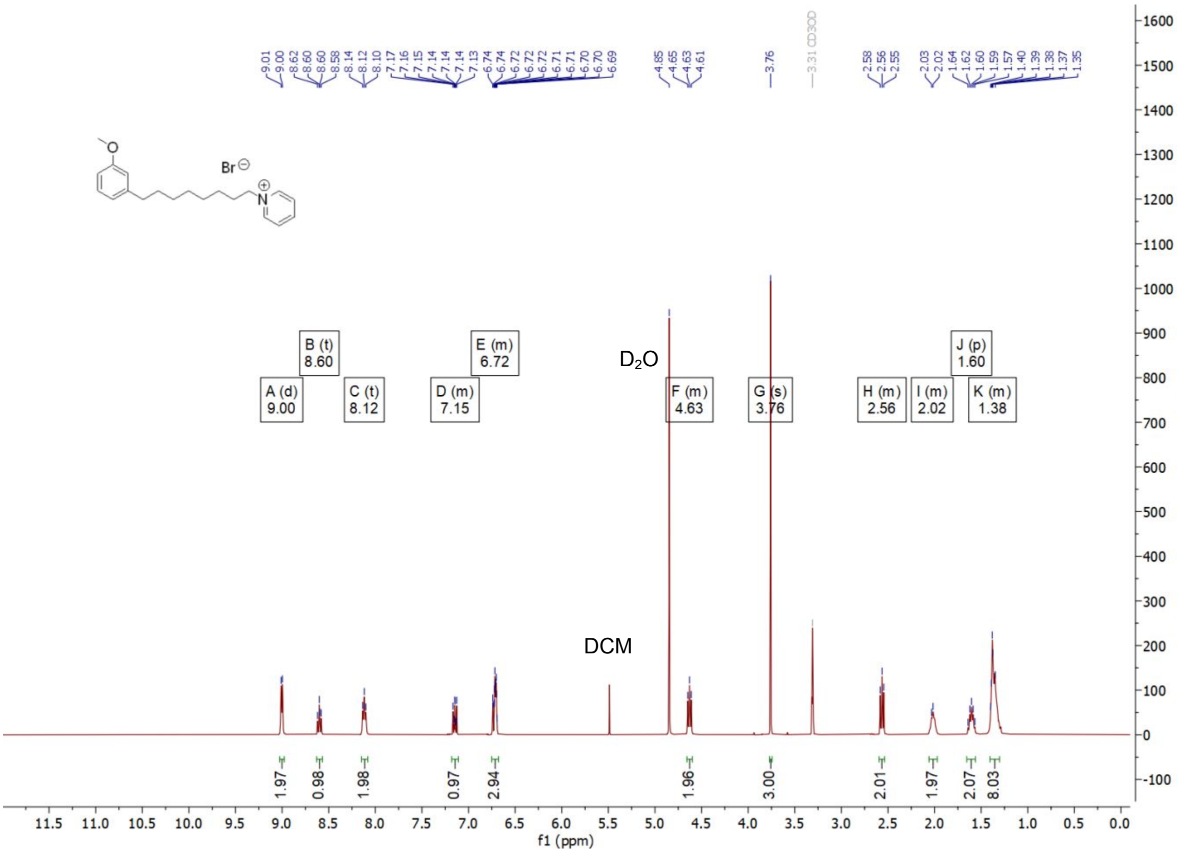

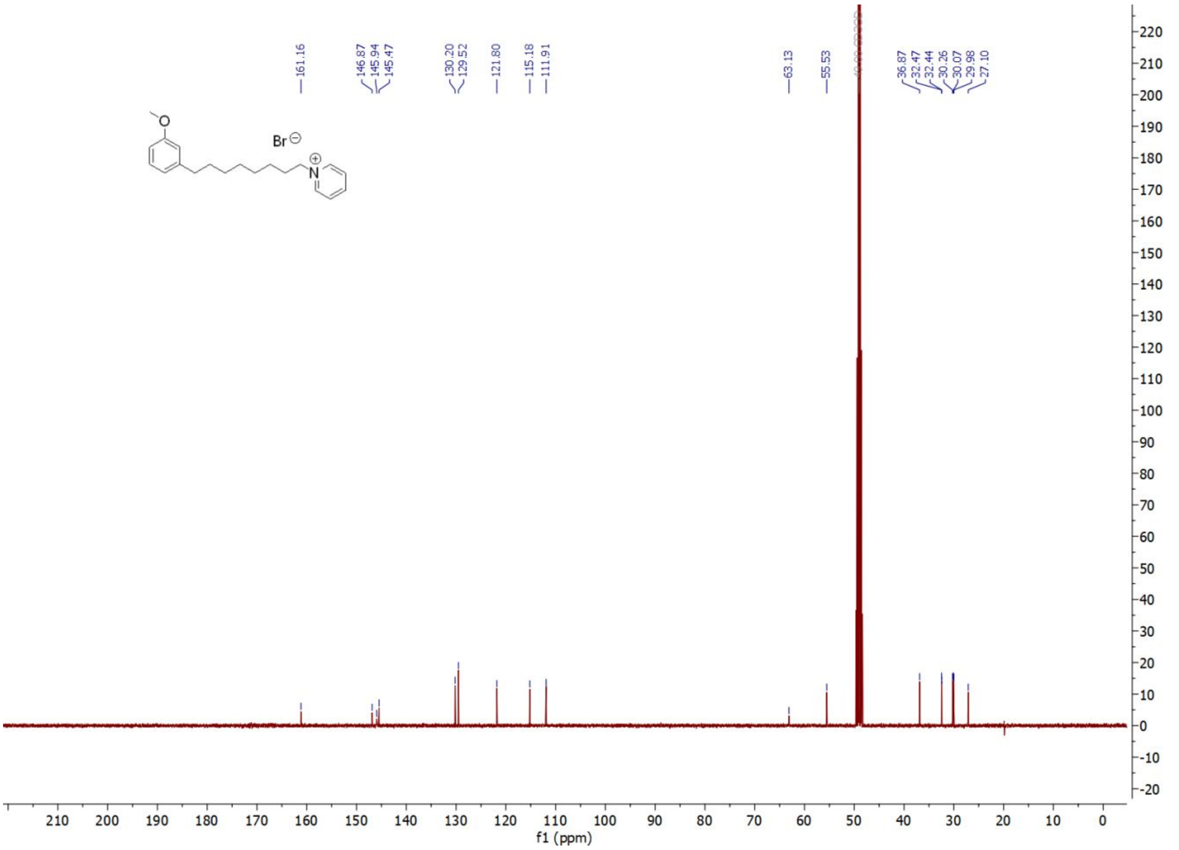

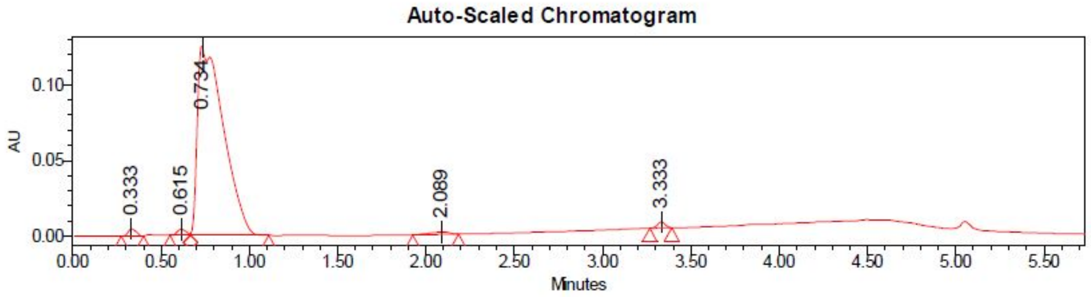

Processed Channel: W2489 ChA 254nm

	Processed Channel	Retention Time (min)	Area	% Area	Height
1	W2489 ChA 254nm	0.333	15004	1.12	4766
2	W2489 ChA 254nm	0.615	14521	1.08	4042
3	W2489 ChA 254nm	0.734	1291385	96.07	124795
4	W2489 ChA 254nm	2.089	11664	0.87	1523
5	W2489 ChA 254nm	3.333	11659	0.87	3973

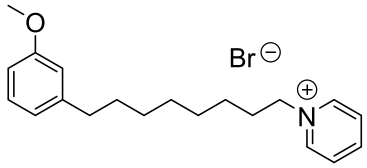

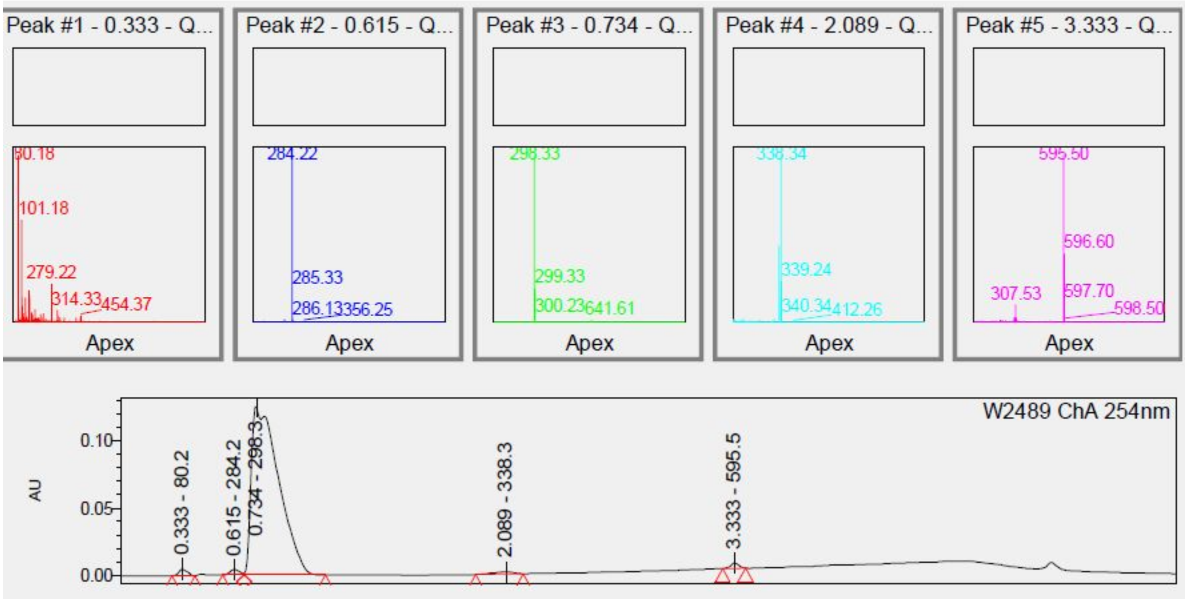

^{1}H NMR, ^{13}C NMR and HPLC-MS chromatogram of compound **15**.

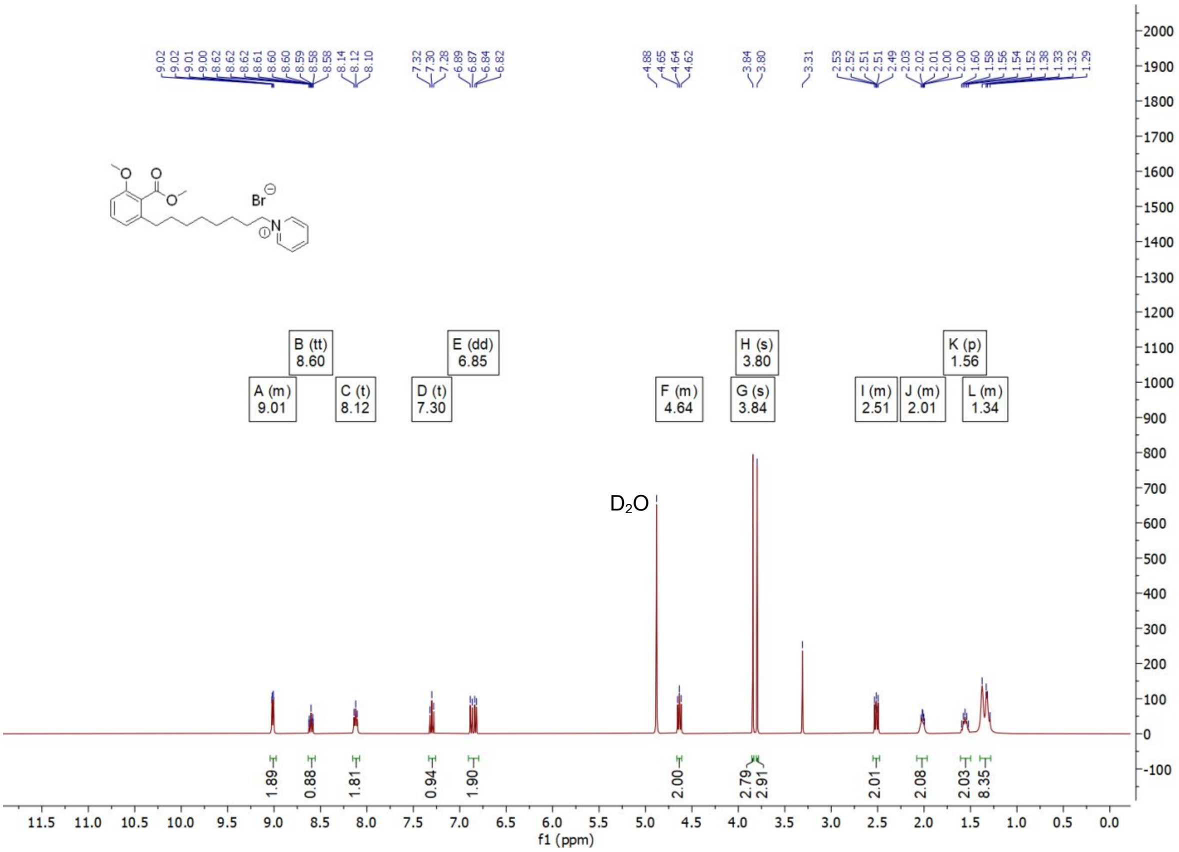

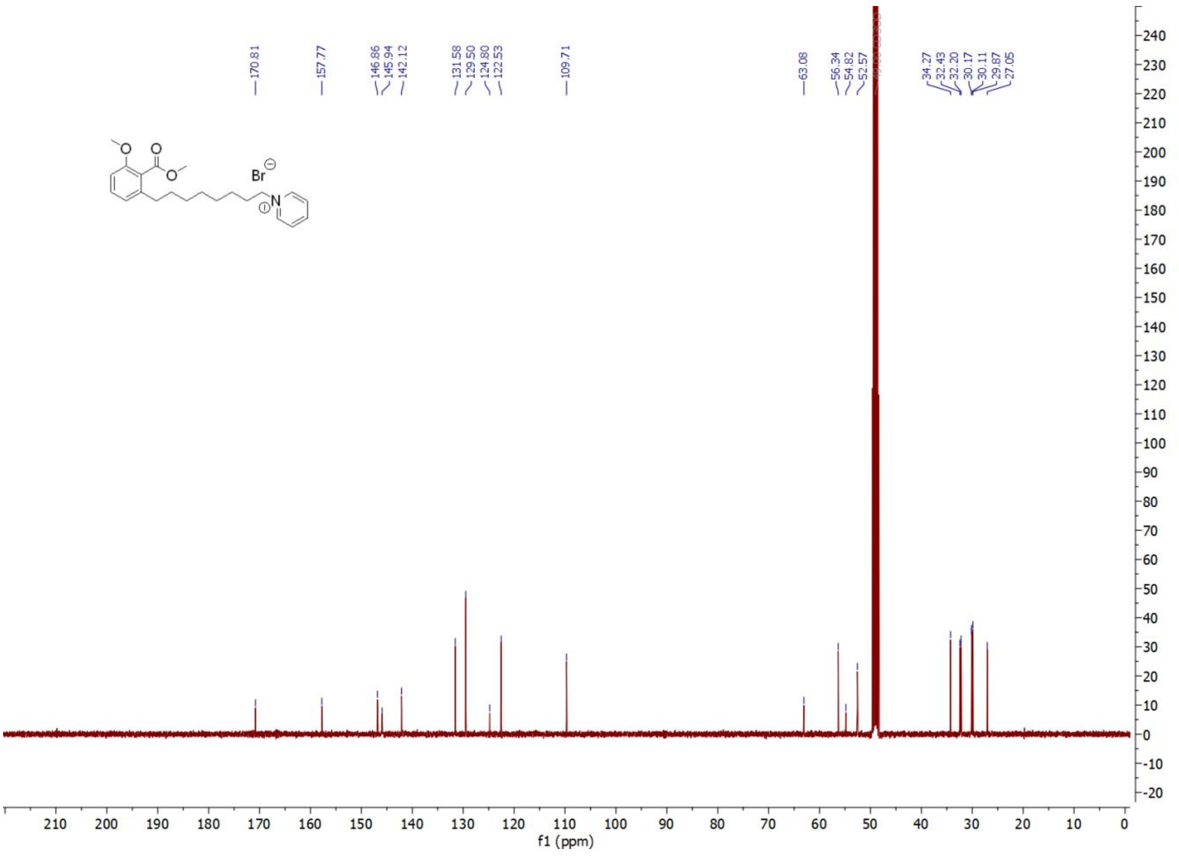

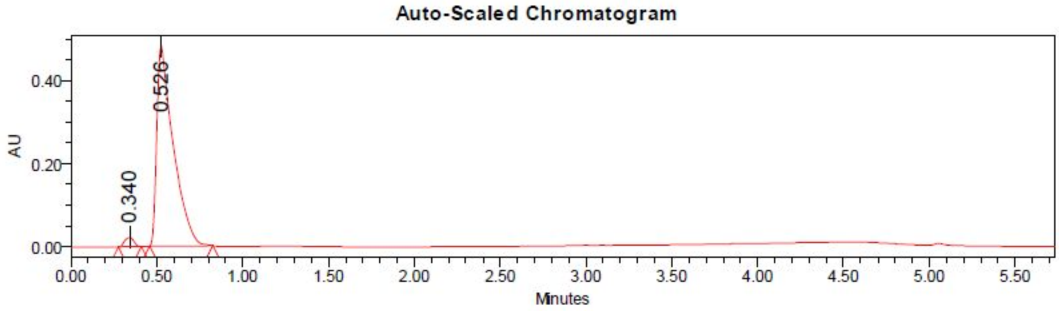

Peak Results

	Name	RT	Area	Height	% Area
1		0.340	78755	21907	2.38
2		0.526	3232179	483600	97.62

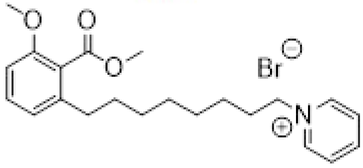

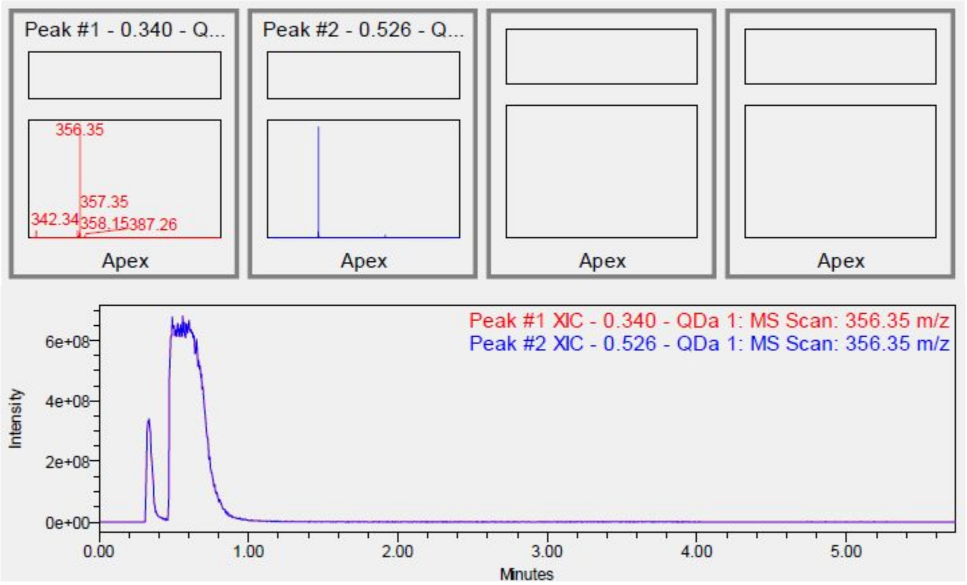

^{1}H NMR, ^{13}C NMR and HPLC-MS chromatogram of compound **16**.

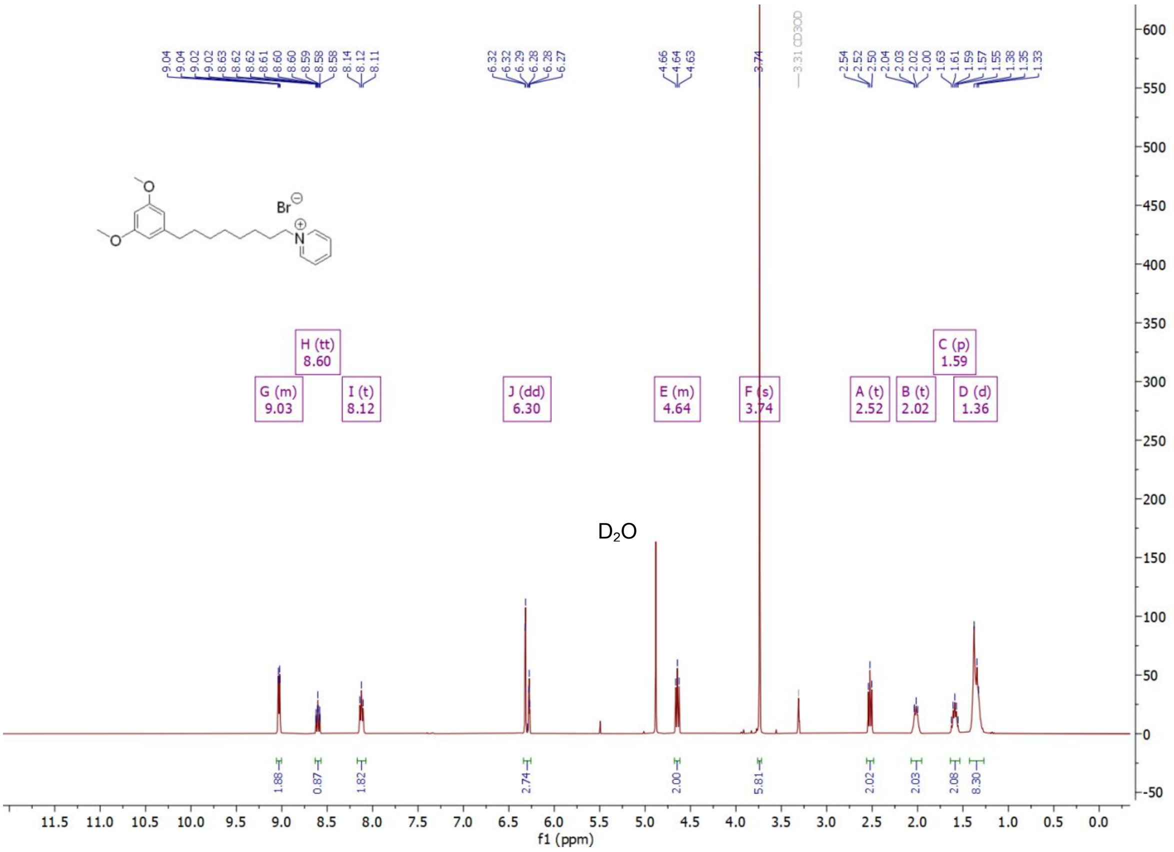

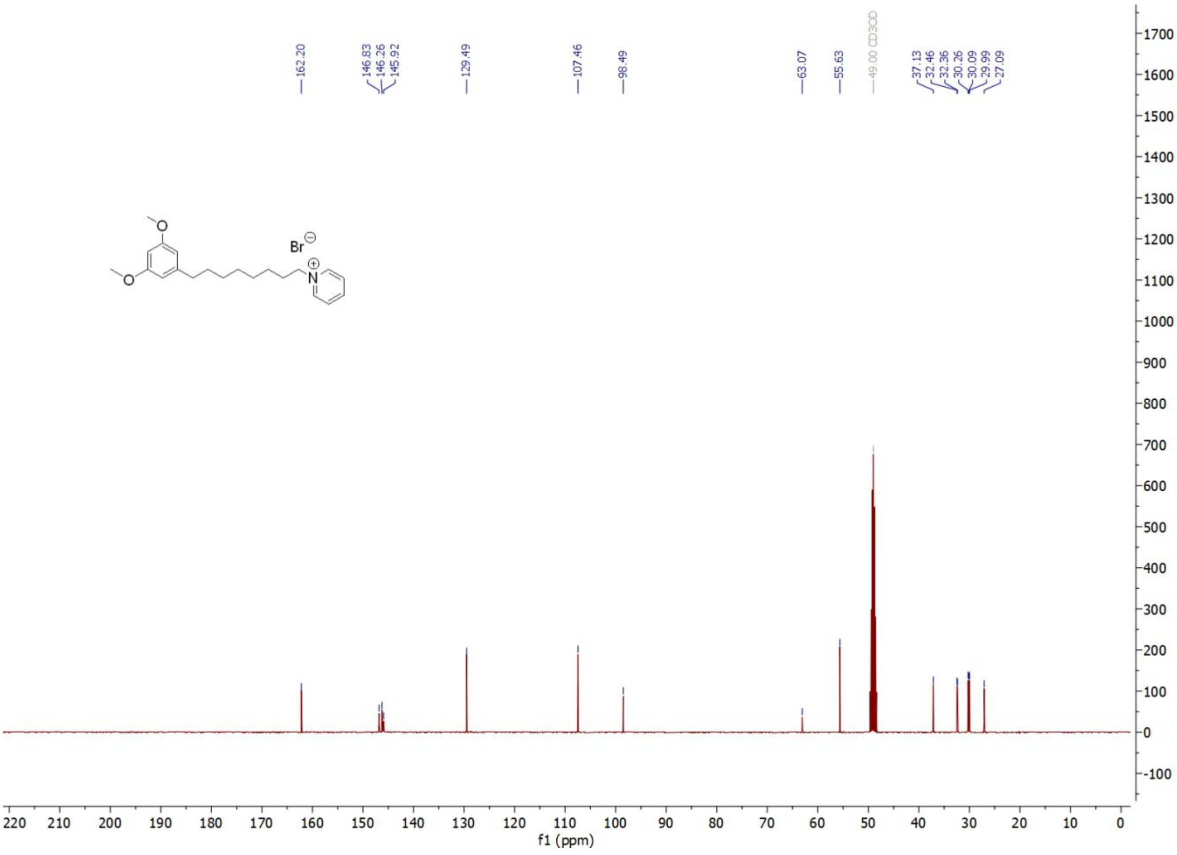

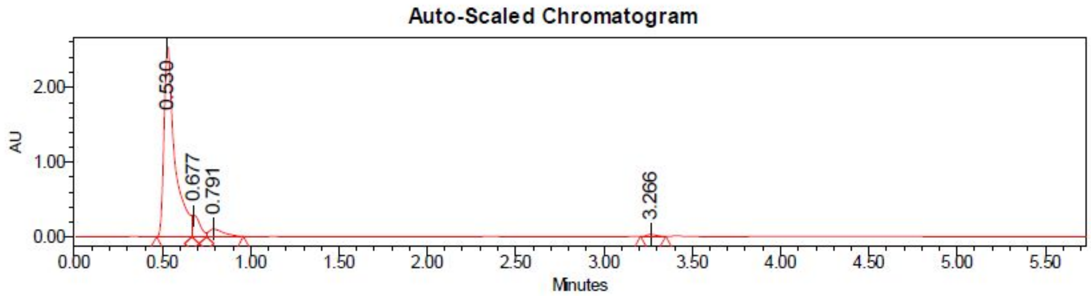

Processed Channel: W2489 ChA 254nm

	Processed Channel	Retention Time (min)	Area	% Area	Height
1	W2489 ChA 254nm	0.530	10873240	87.49	2565221
2	W2489 ChA 254nm	0.677	879993	7.08	283488
3	W2489 ChA 254nm	0.791	592457	4.77	100746
4	W2489 ChA 254nm	3.266	81586	0.66	23877

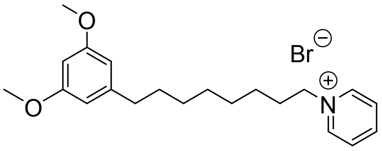

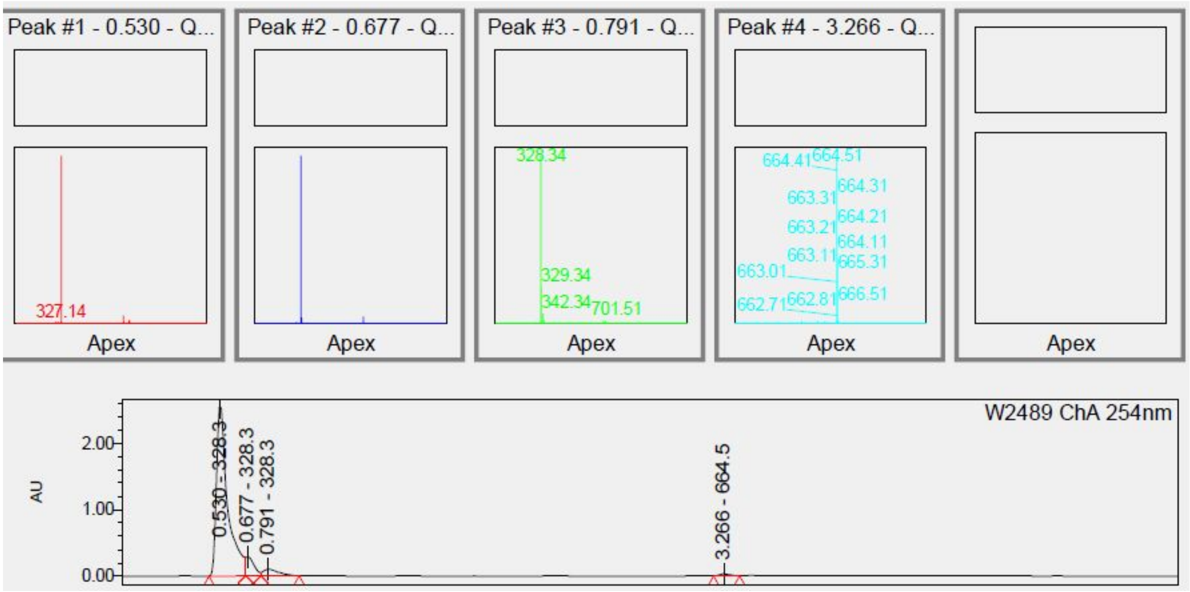

^{1}H NMR, ^{13}C NMR and HPLC-MS chromatogram of compound **17**.

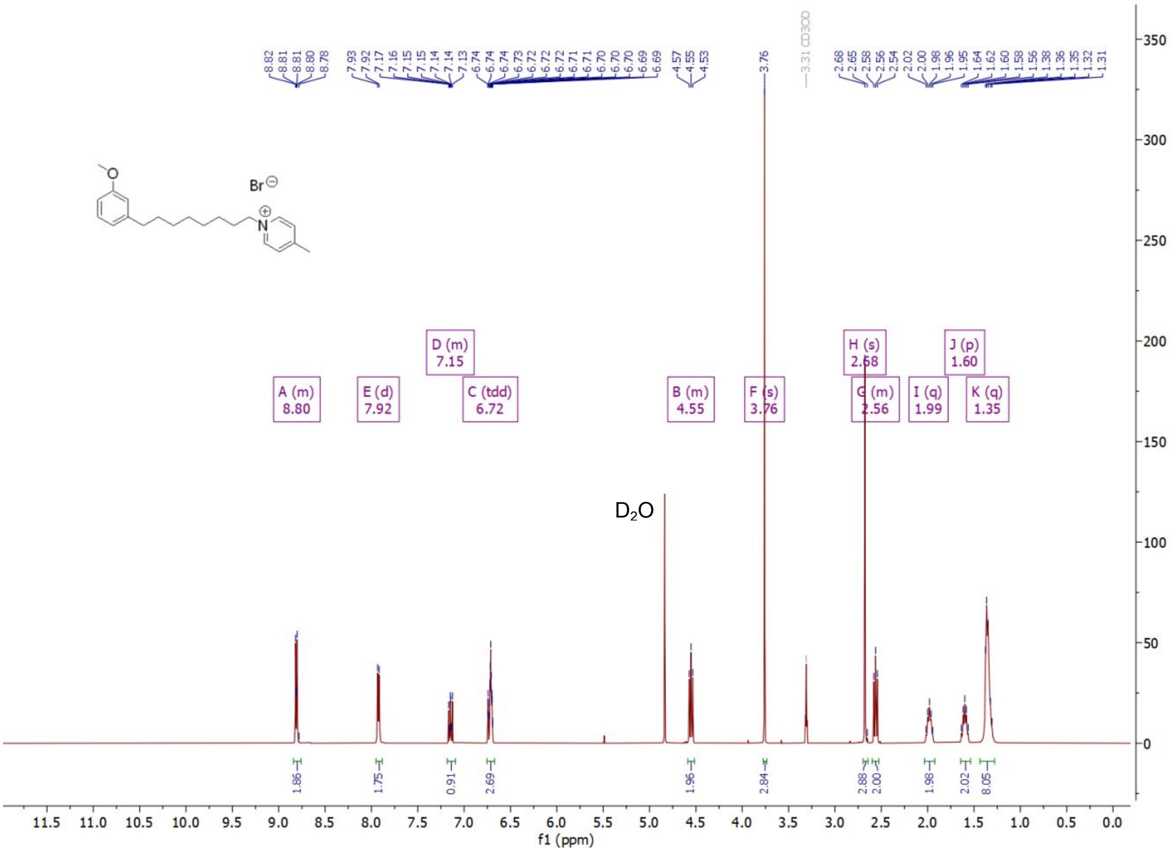

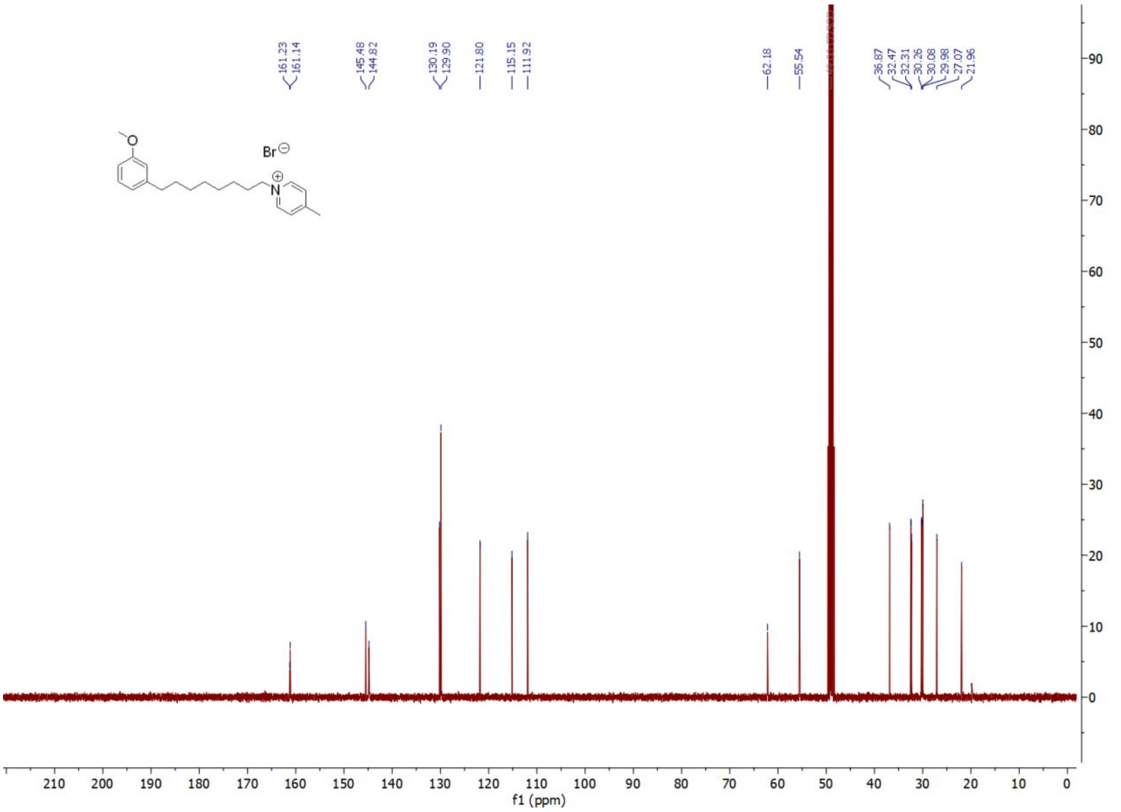

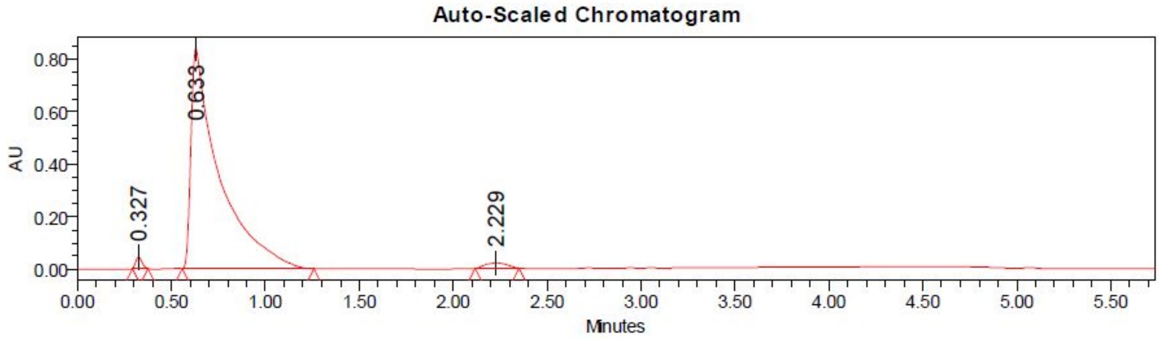

Peak Results

	Name	RT	Area	Height	% Area
1		0.327	99993	43440	1.10
2		0.633	8816367	840134	97.08
3		2.229	165480	20501	1.82

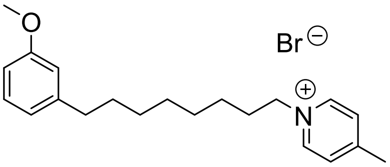

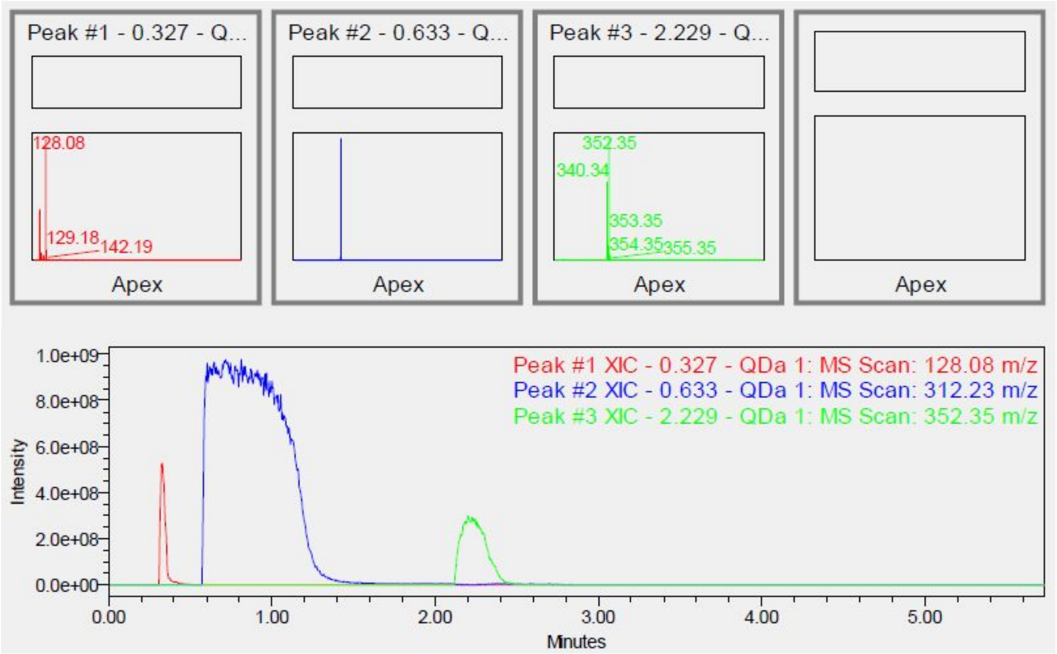

^{1}H NMR, ^{13}C NMR and HPLC-MS chromatogram of compound **18**.

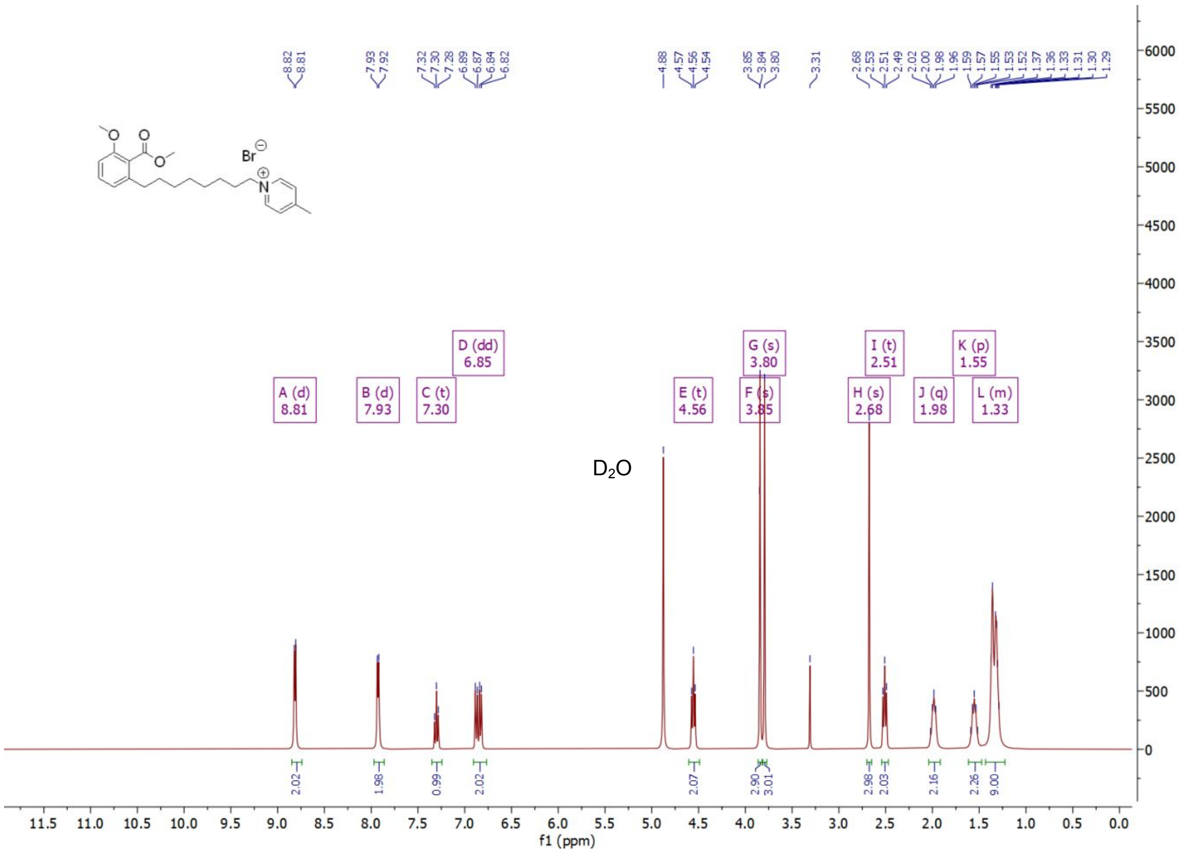

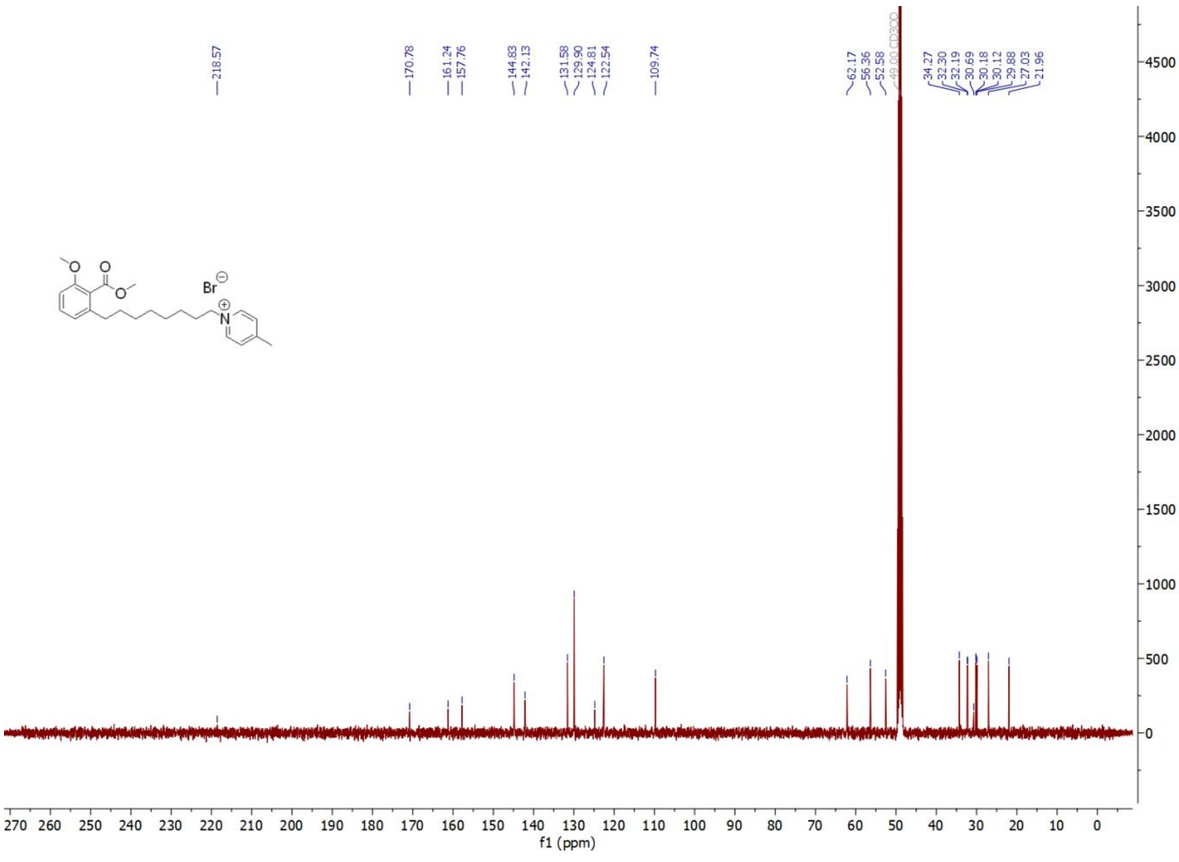

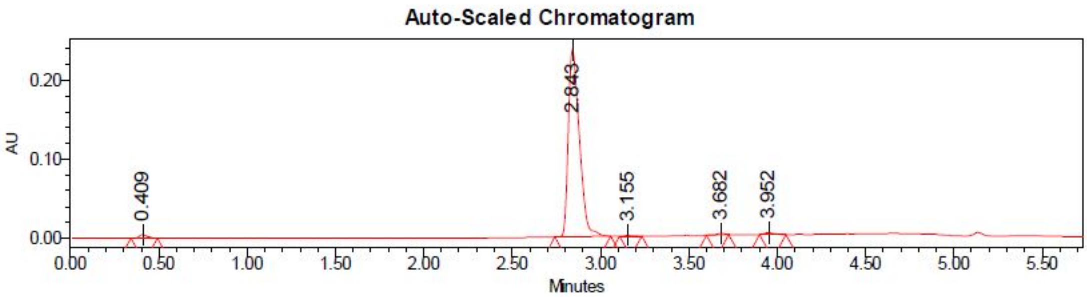

Processed Channel: W2489 ChA 254nm

	Processed Channel	Retention Time (min)	Area	% Area	Height
1	W2489 ChA 254nm	0.409	13557	1.27	4365
2	W2489 ChA 254nm	2.843	1034234	96.98	238829
3	W2489 ChA 254nm	3.155	4284	0.40	1174
4	W2489 ChA 254nm	3.682	6007	0.56	1875
5	W2489 ChA 254nm	3.952	8307	0.78	2382

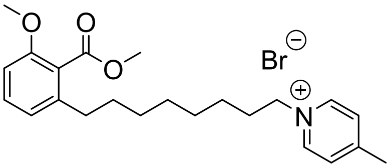

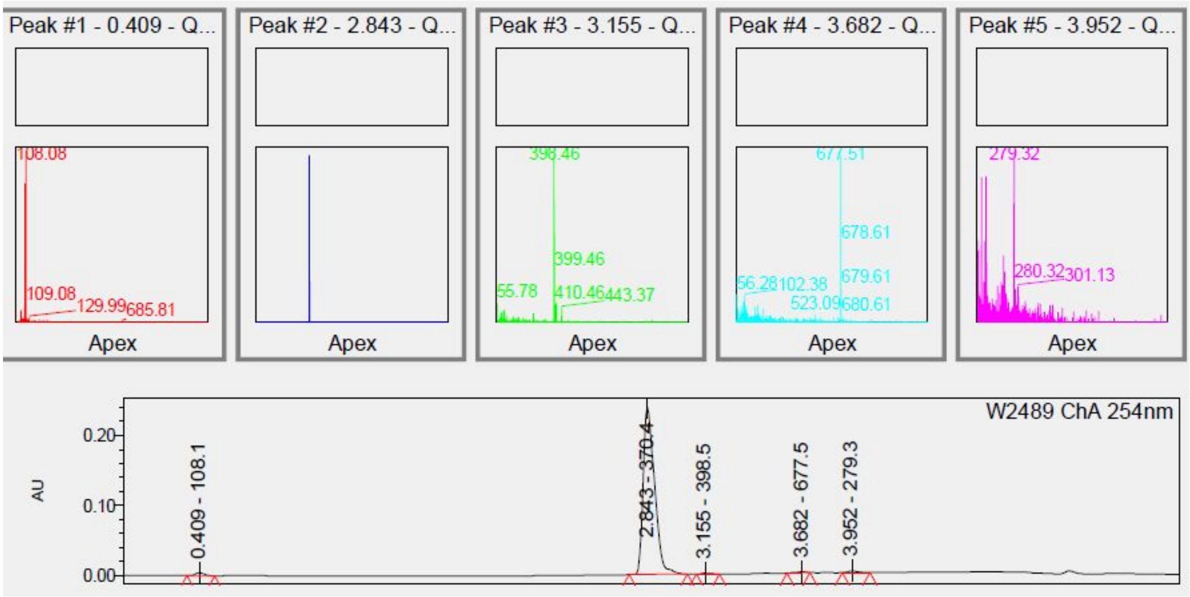

^{1}H NMR, ^{13}C NMR and HPLC-MS chromatogram of compound **19**.

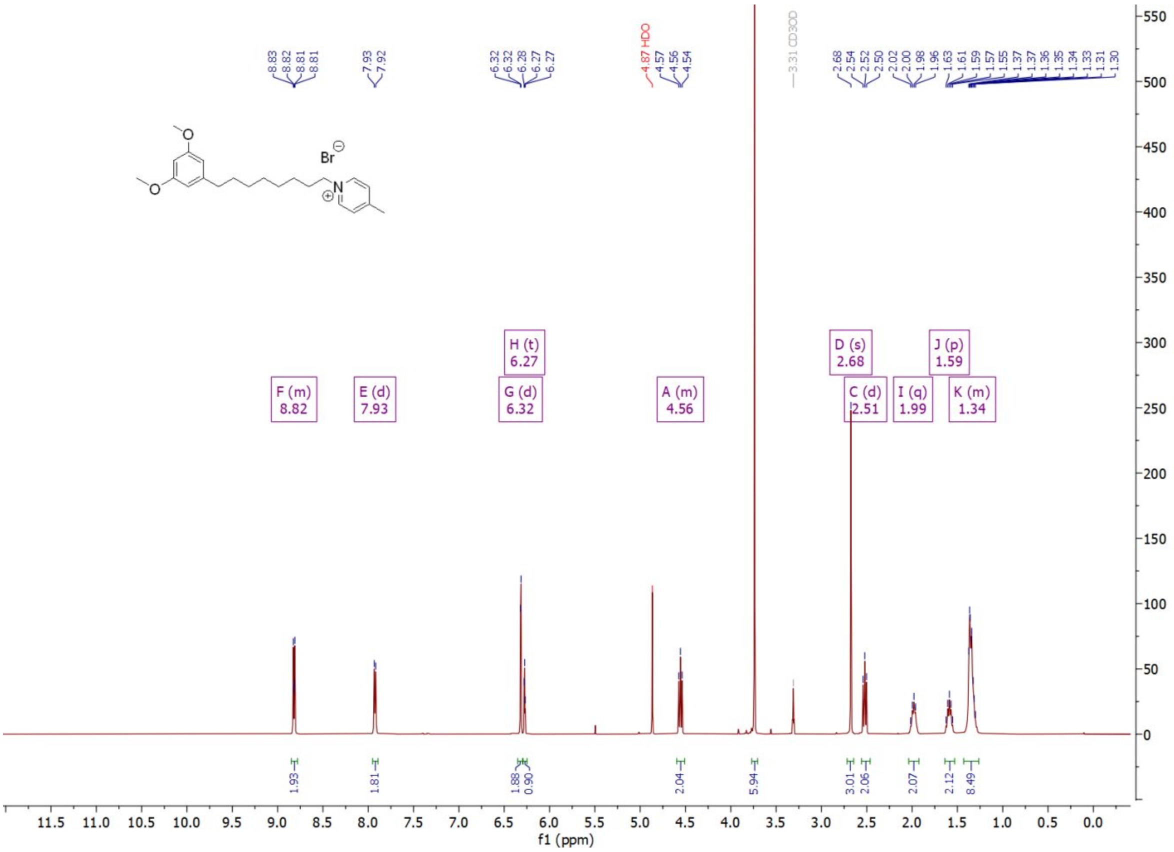

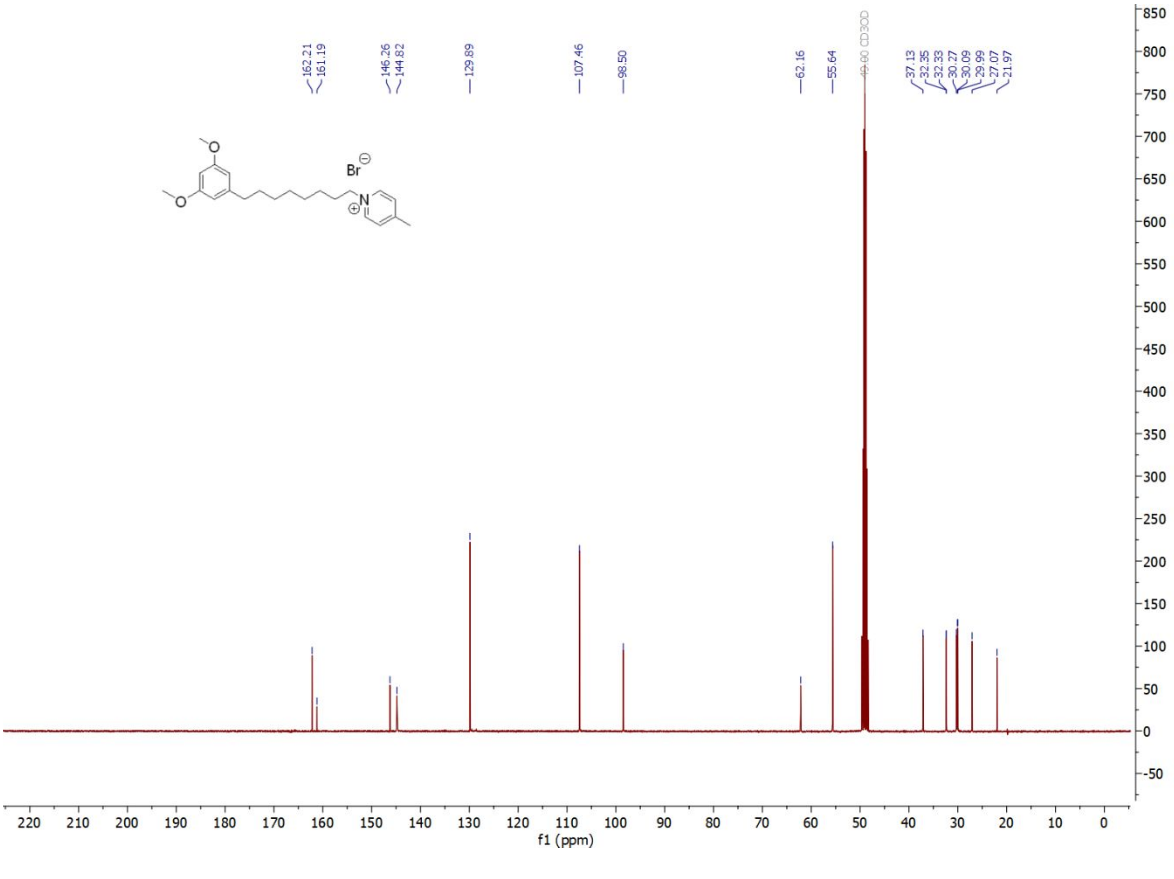

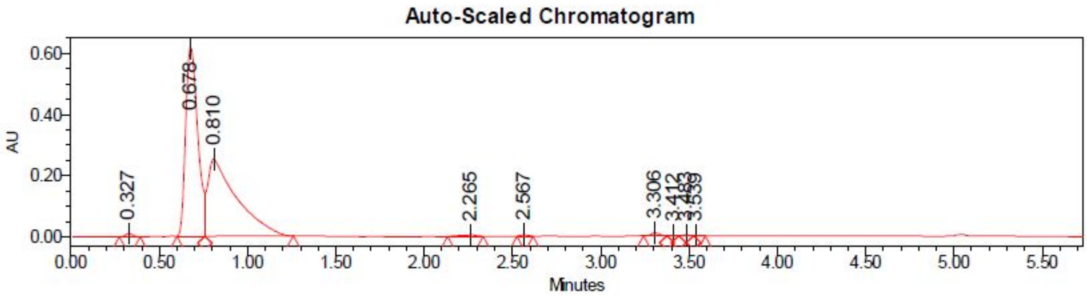

Processed Channel: W2489 ChA 254nm

	Processed Channel	Retention Time (min)	Area	% Area	Height
1	W2489 ChA 254nm	0.327	35086	0.59	11841
2	W2489 ChA 254nm	0.678	2936169	49.20	620931
3	W2489 ChA 254nm	0.810	2899313	48.58	252731
4	W2489 ChA 254nm	2.265	34818	0.58	5033
5	W2489 ChA 254nm	2.567	14519	0.24	5151
6	W2489 ChA 254nm	3.306	31800	0.53	9397
7	W2489 ChA 254nm	3.412	2479	0.04	948
8	W2489 ChA 254nm	3.483	10523	0.18	3627
9	W2489 ChA 254nm	3.539	2804	0.05	1224

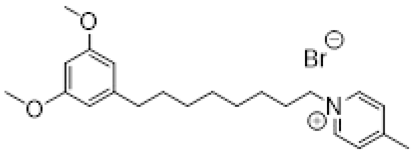

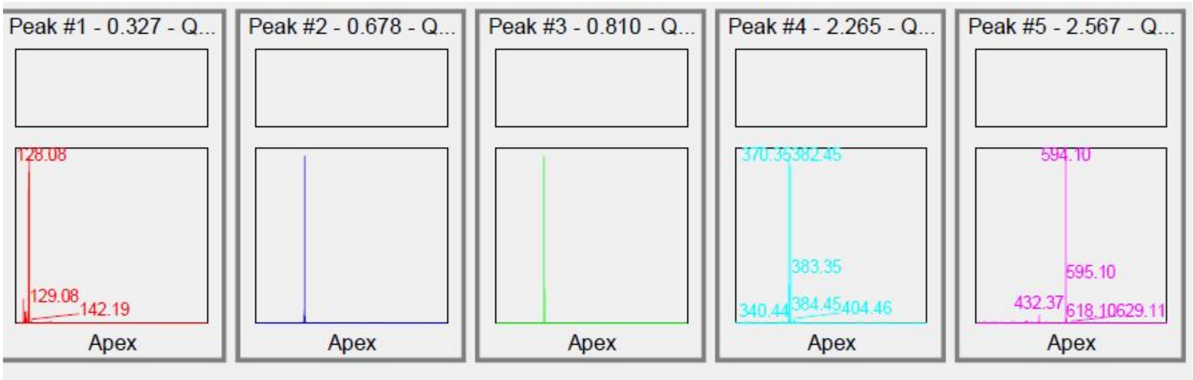

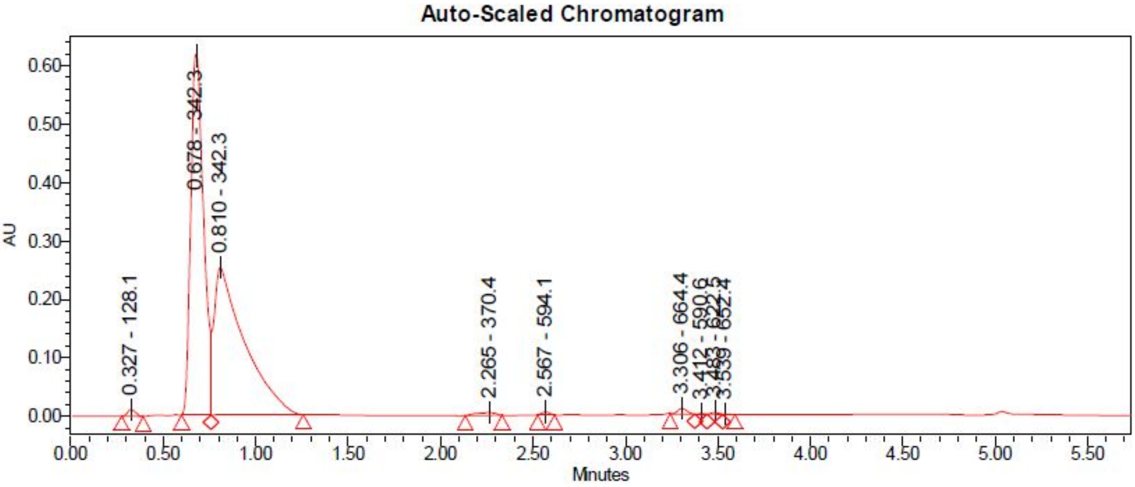

^{1}H NMR, ^{13}C NMR and HPLC-MS chromatogram of compound **20**.

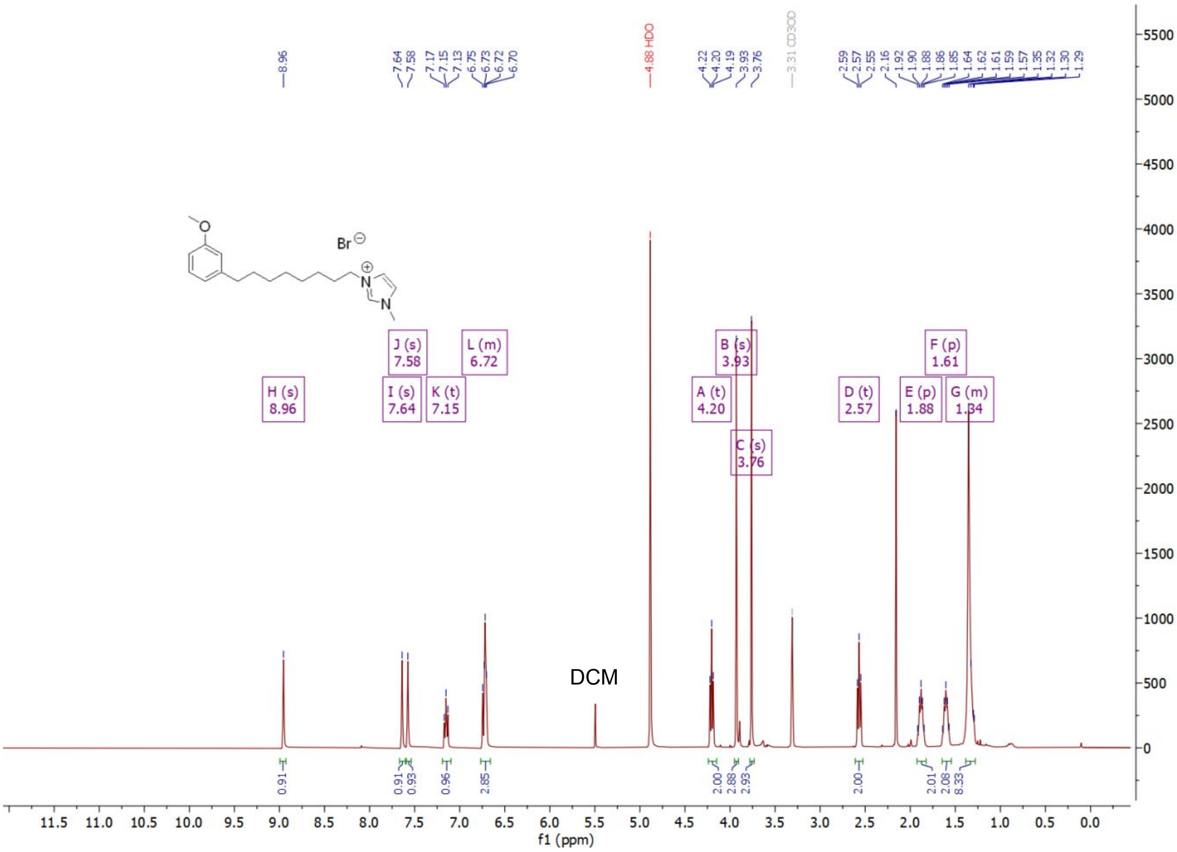

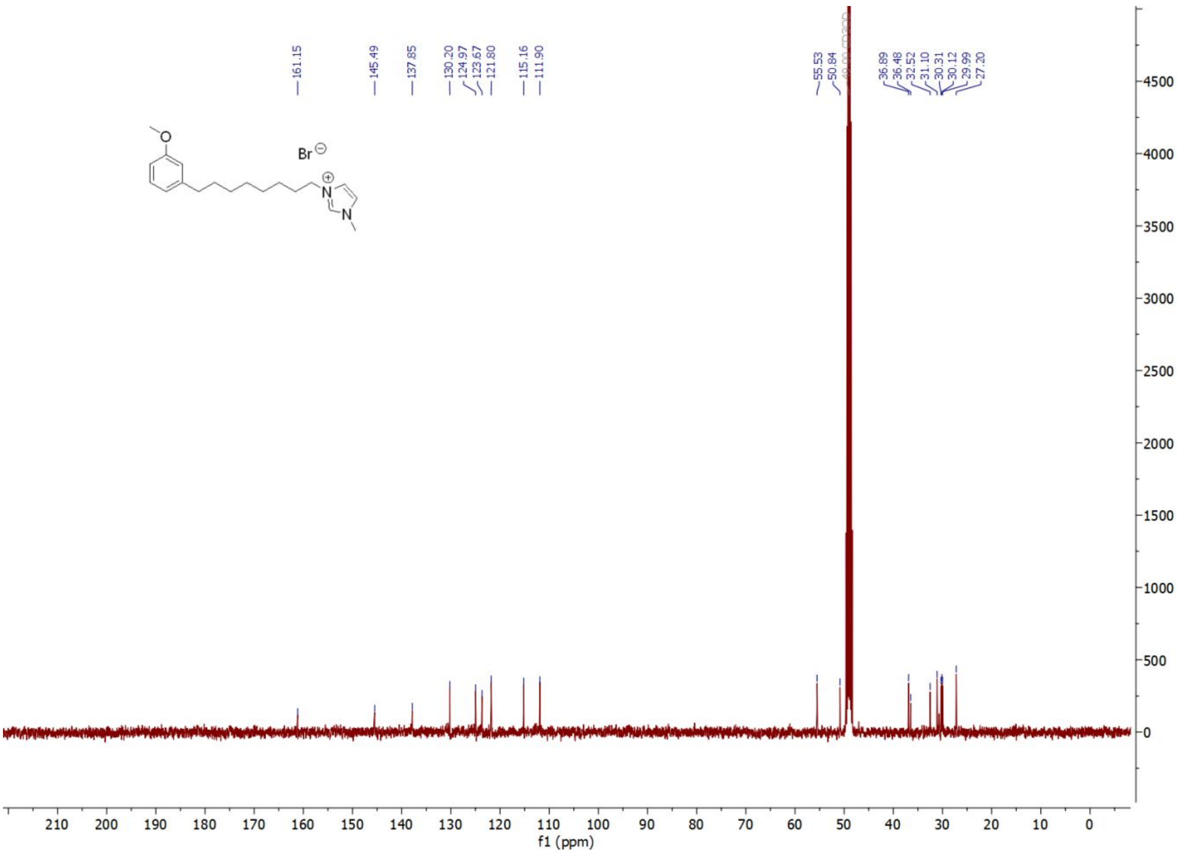

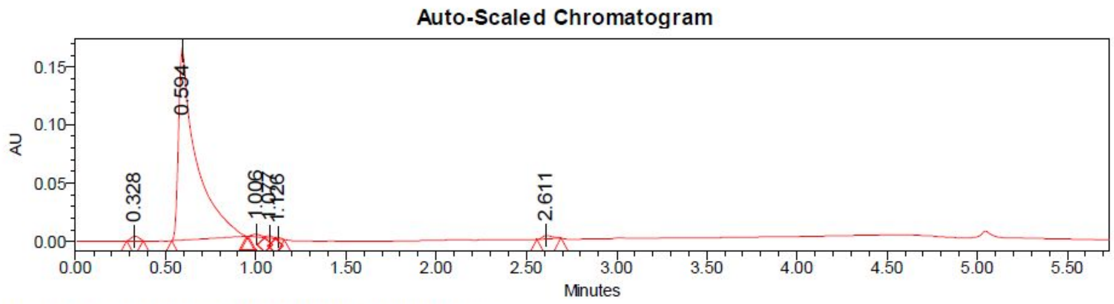

Processed Channel: W2489 ChA 254nm

	Processed Channel	Retention Time (min)	Area	% Area	Height
1	W2489 ChA 254nm	0.328	12362	1.06	3985
2	W2489 ChA 254nm	0.594	1125636	96.57	164716
3	W2489 ChA 254nm	1.006	8059	0.69	2258
4	W2489 ChA 254nm	1.077	5367	0.46	2122
5	W2489 ChA 254nm	1.126	2657	0.23	1274
6	W2489 ChA 254nm	2.611	11563	0.99	2843

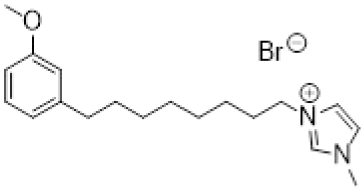

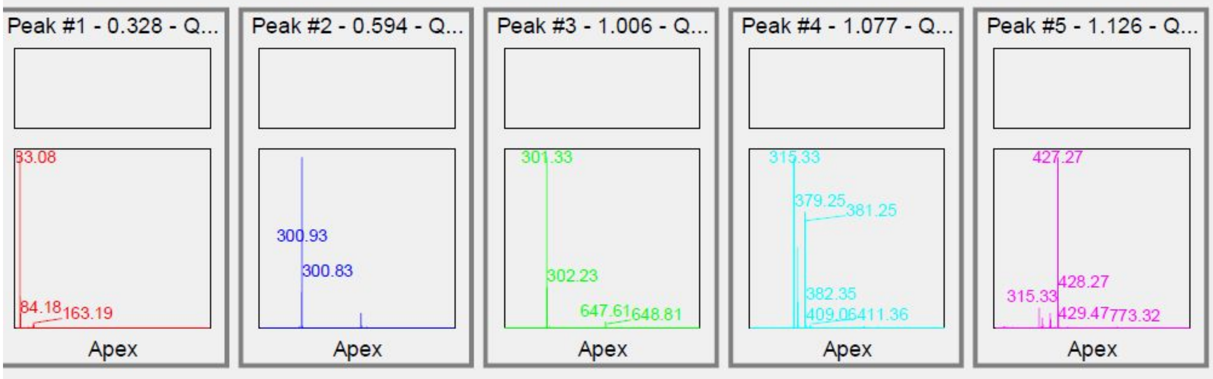

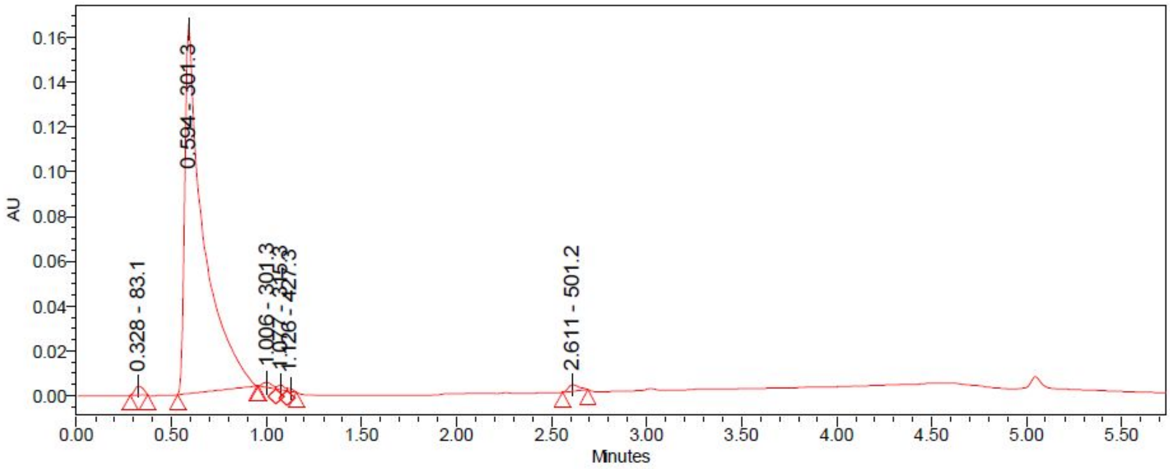

^{1}H NMR, ^{13}C NMR and HPLC-MS chromatogram of compound **21**.

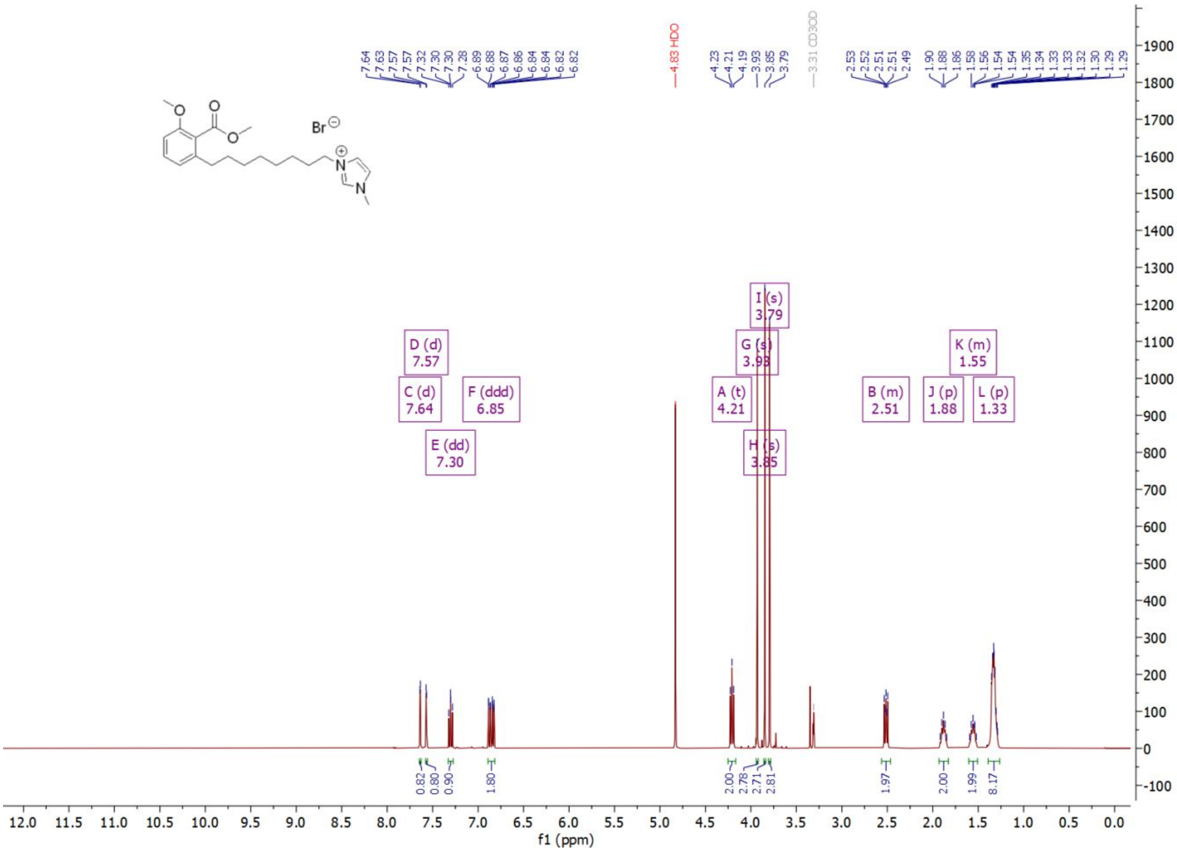

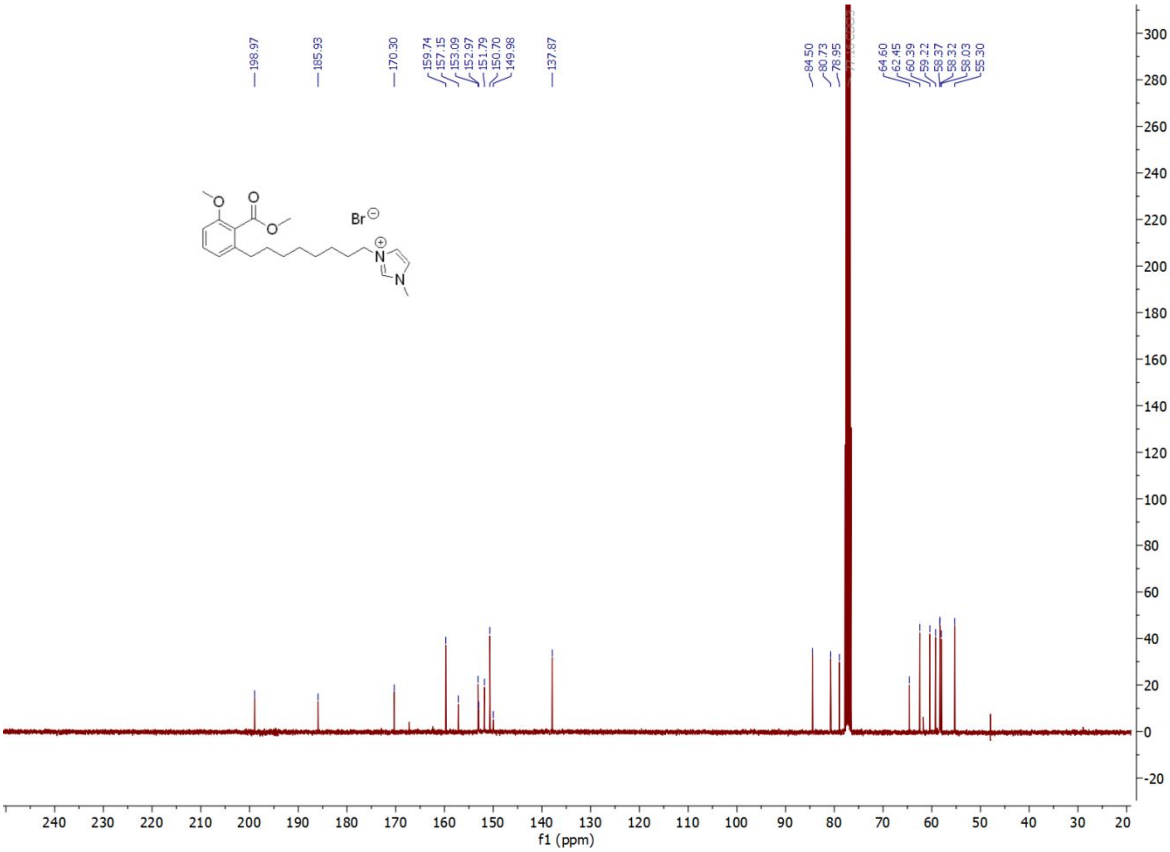

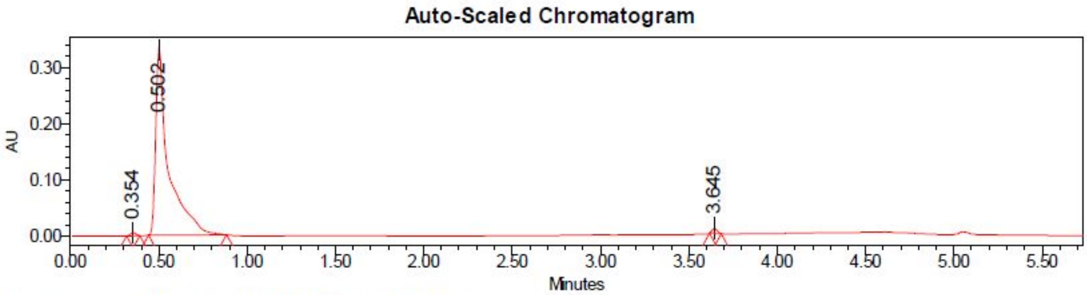

Processed Channel: W2489 ChA 254nm

	Processed Channel	Retention Time (min)	Area	% Area	Height
1	W2489 ChA 254nm	0.354	12032	0.70	5210
2	W2489 ChA 254nm	0.502	1698708	98.28	333667
3	W2489 ChA 254nm	3.645	17629	1.02	8516

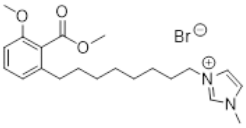

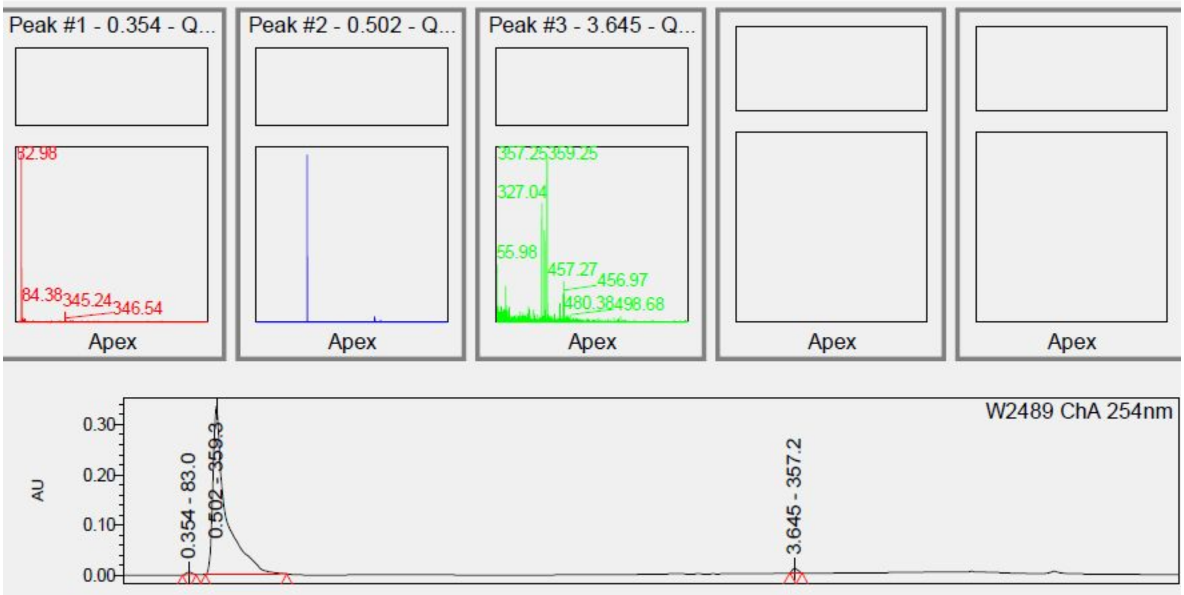

^{1}H NMR and ^{13}C NMR of compound **22**.

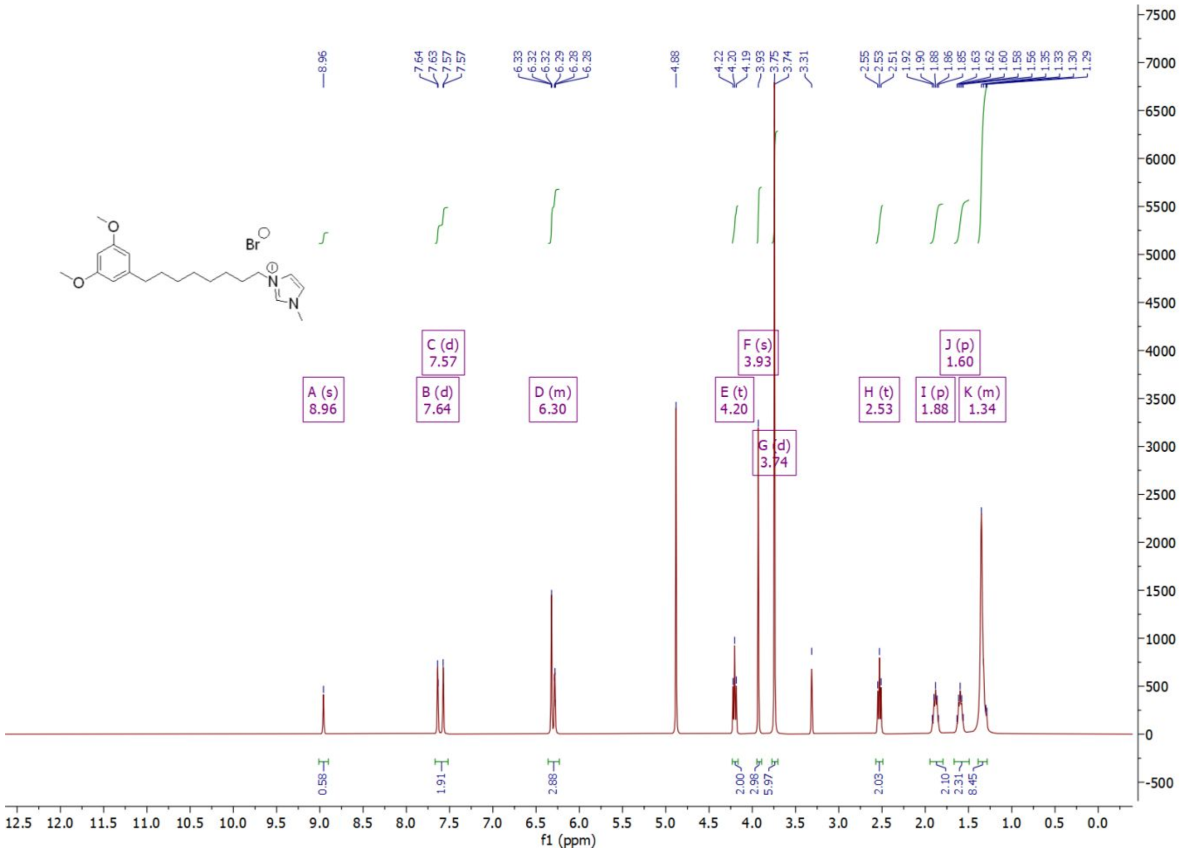

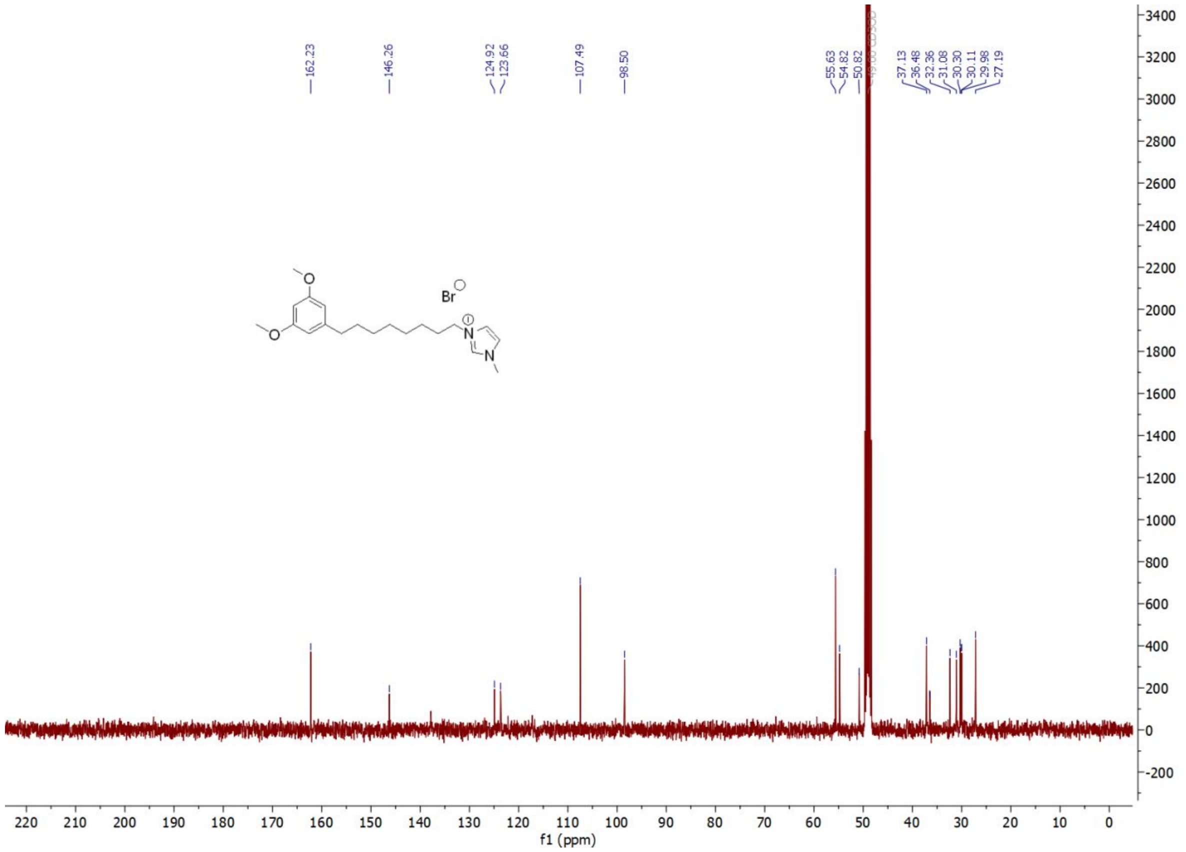

^{1}H NMR, ^{13}C NMR and HPLC-MS chromatogram of compound **23**

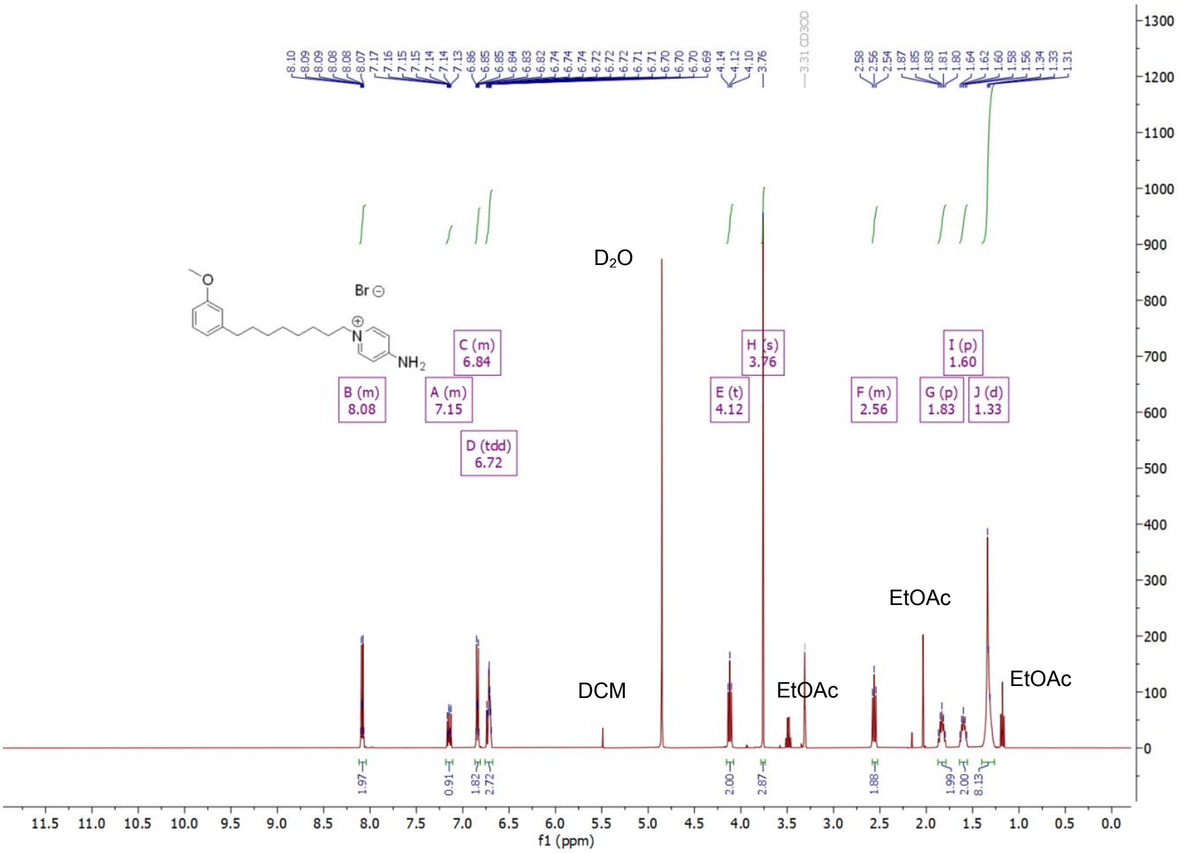

.

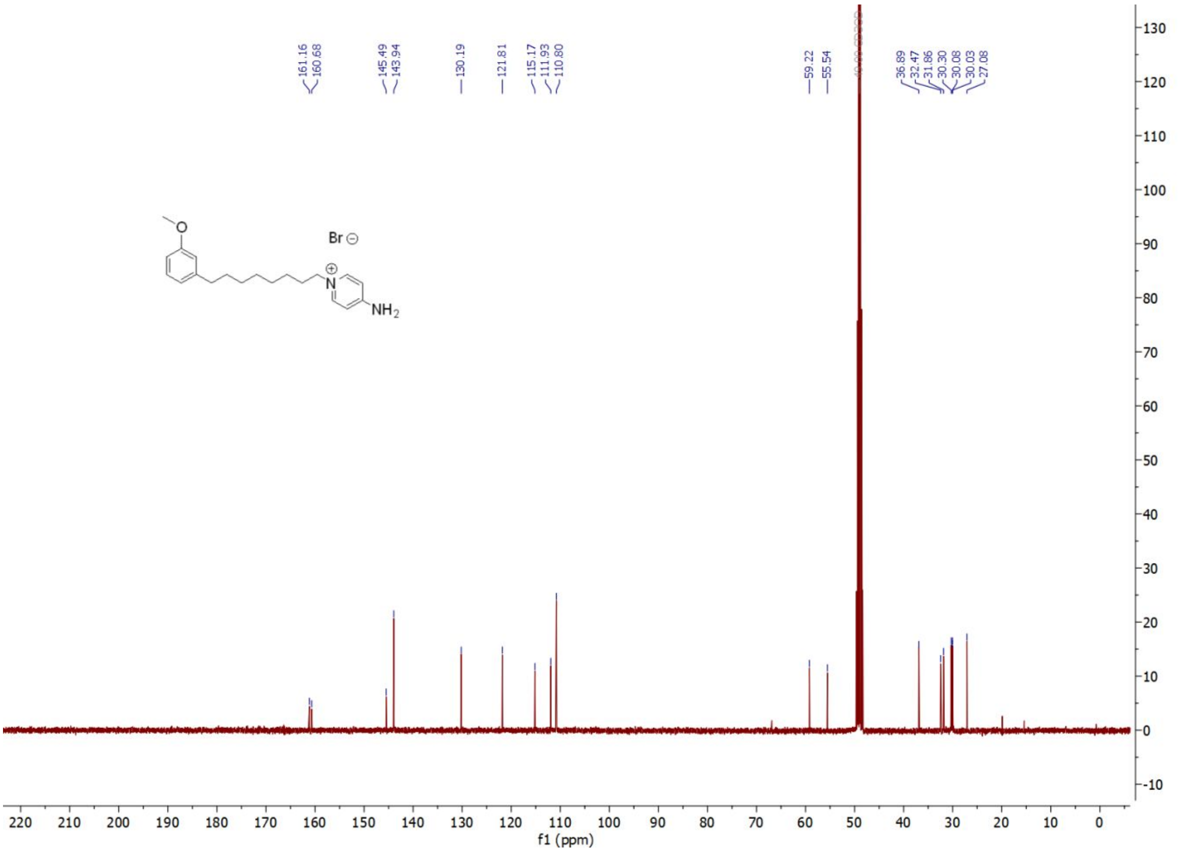

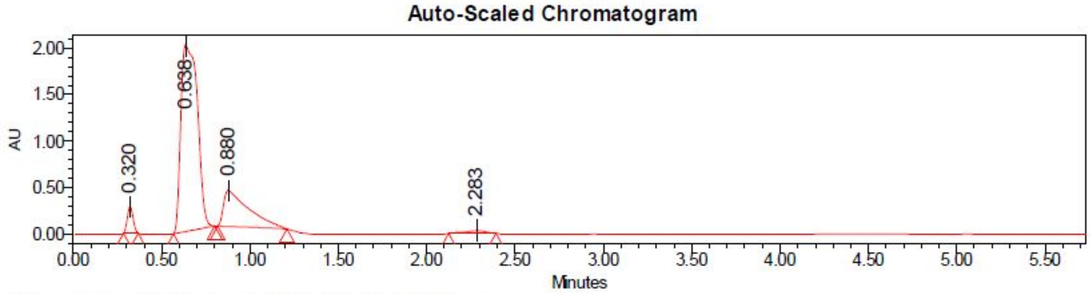

Processed Channel: W2489 ChA 254nm

	Processed Channel	Retention Time (min)	Area	% Area	Height
1	W2489 ChA 254nm	0.320	650097	3.66	285471
2	W2489 ChA 254nm	0.638	13026912	73.38	2017796
3	W2489 ChA 254nm	0.880	3821491	21.53	390715
4	W2489 ChA 254nm	2.283	253017	1.43	30506

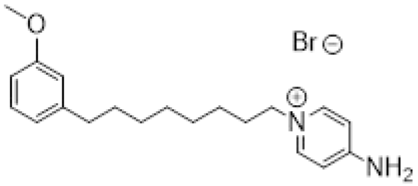

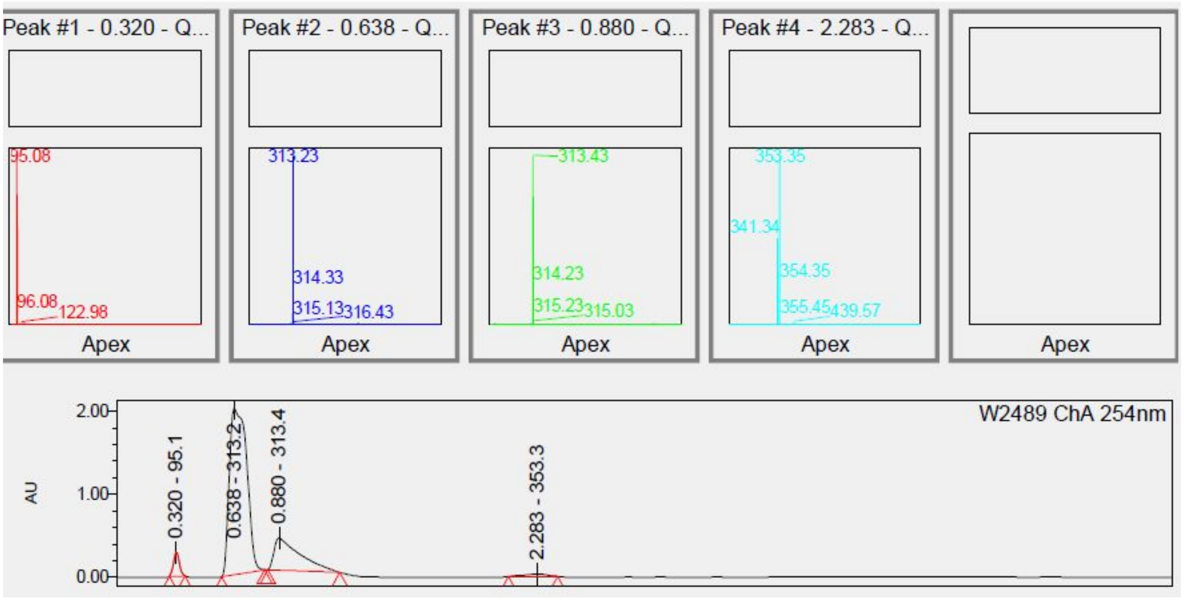

^{1}H NMR, ^{13}C NMR and HPLC-MS chromatogram of compound **24**.

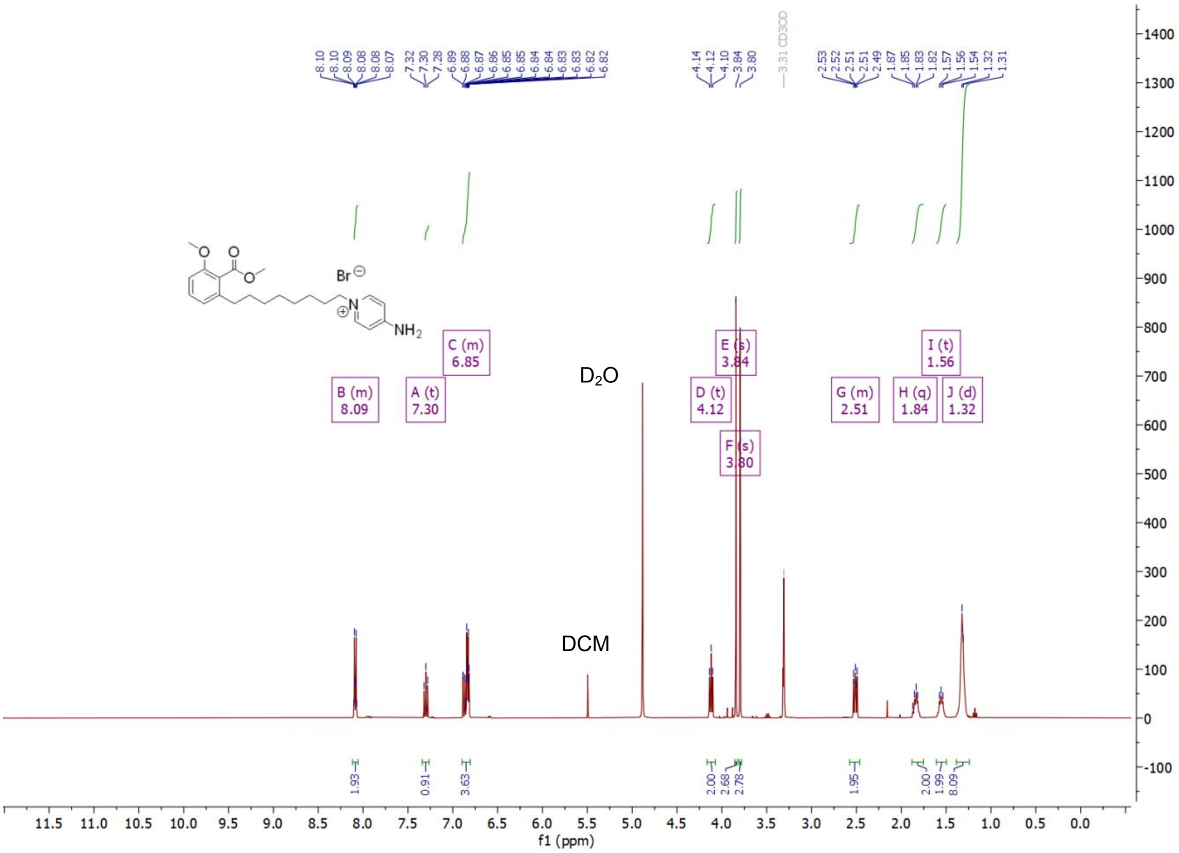

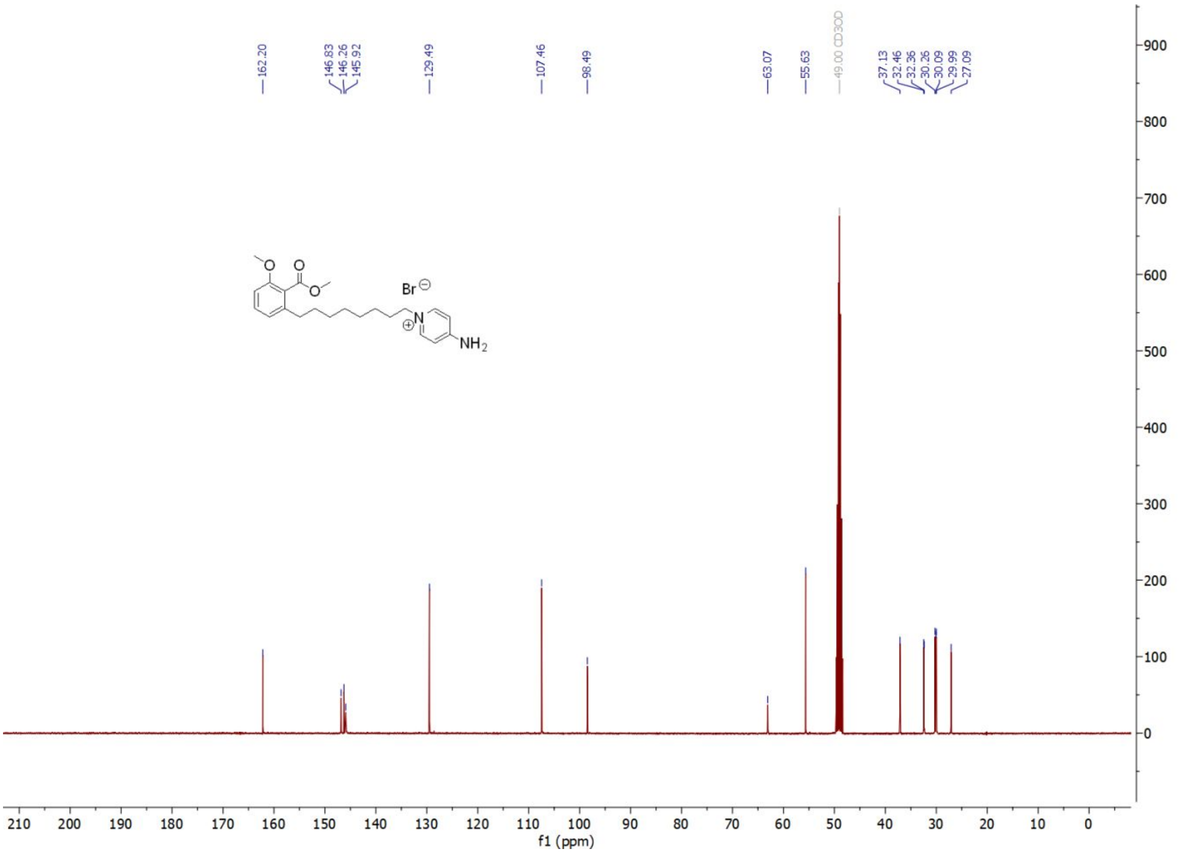

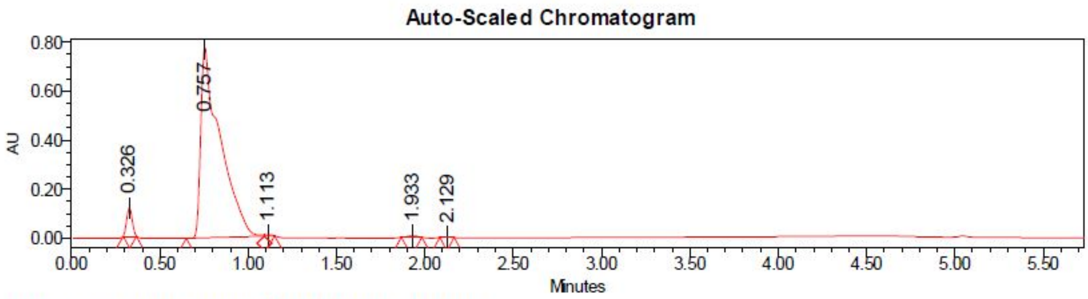

Processed Channel: W2489 ChA 254nm

	Processed Channel	Retention Time (min)	Area	% Area	Height
1	W2489 ChA 254nm	0.326	252253	4.02	115244
2	W2489 ChA 254nm	0.757	5974926	95.23	771378
3	W2489 ChA 254nm	1.113	12302	0.20	4431
4	W2489 ChA 254nm	1.933	25673	0.41	6275
5	W2489 ChA 254nm	2.129	9048	0.14	2966

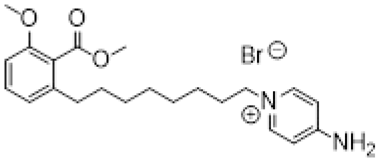

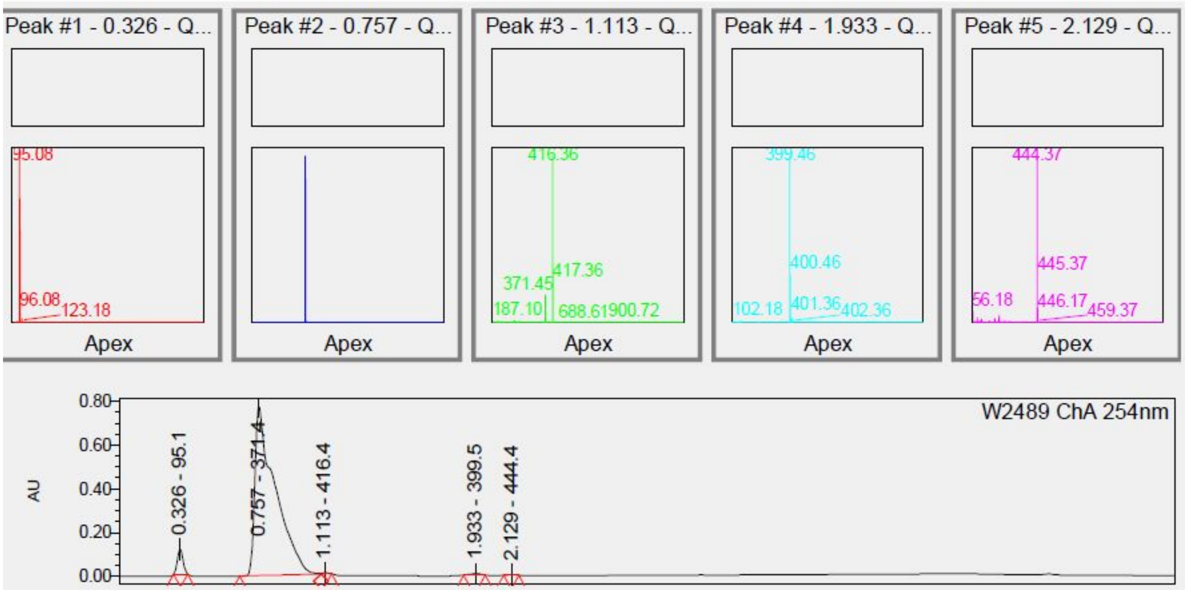

^{1}H NMR, ^{13}C NMR and HPLC-MS chromatogram of compound **25**.

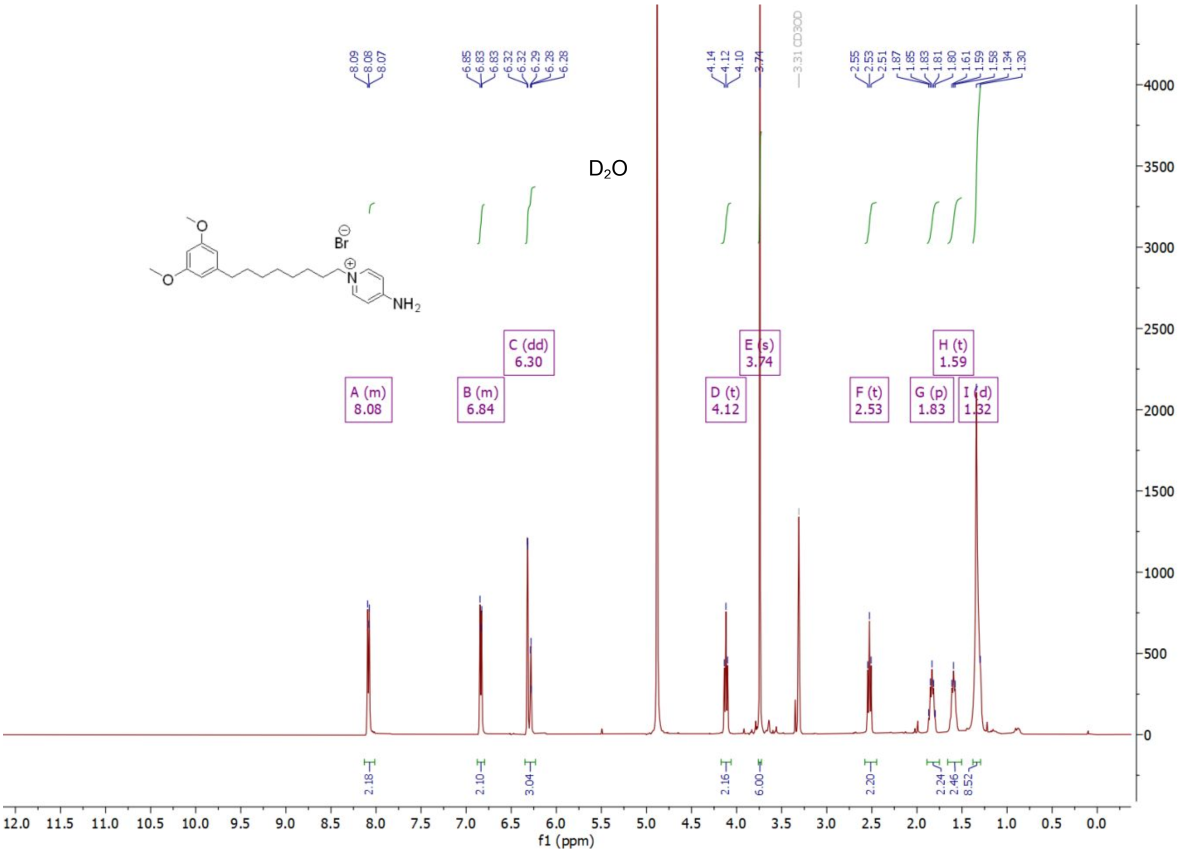

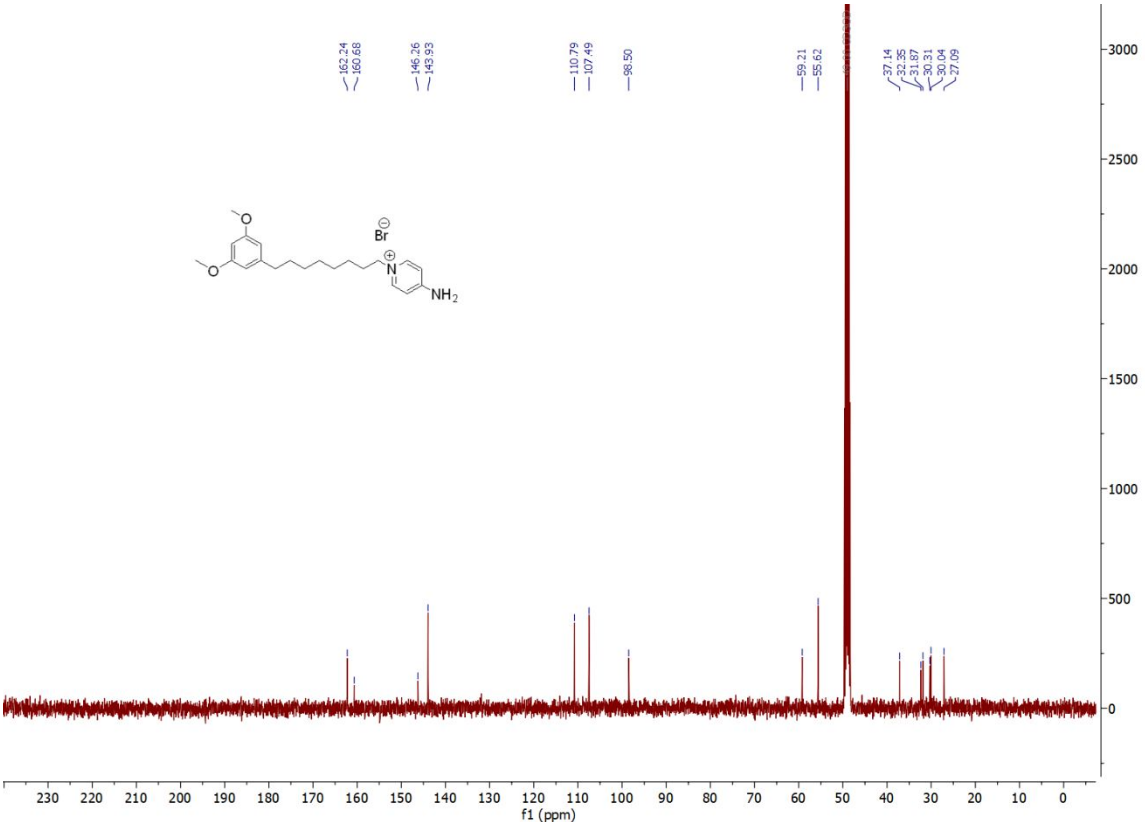

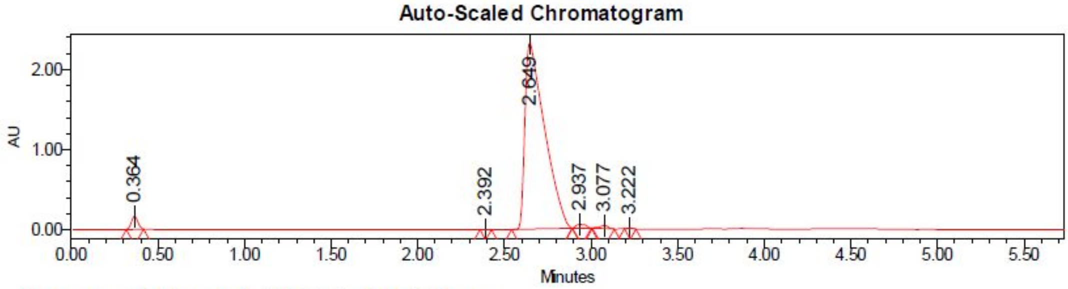

Processed Channel: W2489 ChA 254nm

	Processed Channel	Retention Time (min)	Area	% Area	Height
1	W2489 ChA 254nm	0.364	419630	2.20	158969
2	W2489 ChA 254nm	2.392	7196	0.04	3473
3	W2489 ChA 254nm	2.649	18283736	96.01	2312673
4	W2489 ChA 254nm	2.937	181016	0.95	46060
5	W2489 ChA 254nm	3.077	127015	0.67	38648
6	W2489 ChA 254nm	3.222	25321	0.13	11465

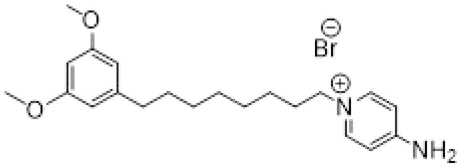

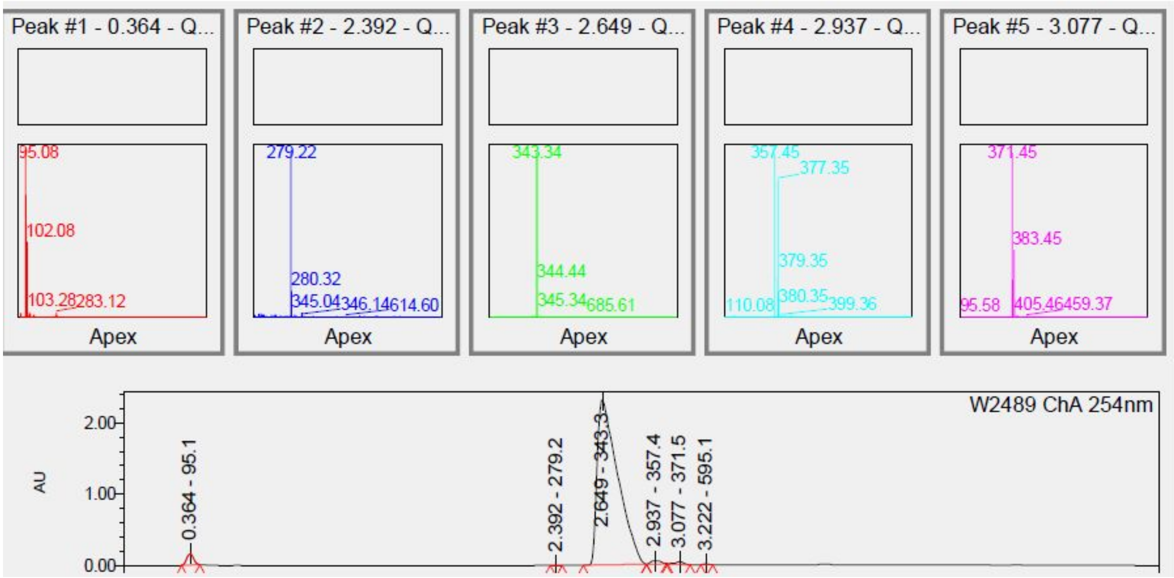

6. References

(1) U.S. EPA. *Report on the 2013 U.S. Environmental Protection Agency (EPA) International Decontamination Research and Development Conference*; 2014; Vol. Research T.

(2) US EPA. Interpretive Assistance Document for Assessment of Discrete Organic Chemicals. Sustainable Futures Summary Assessment. **2013**, 20.

(3) Goyanes, A.; Buanz, A. B. M.; Hatton, G. B.; Gaisford, S.; Basit, A. W. Targeting Drugs to Mitochondria. *Eur. J. Pharm. Biopharm.* **2015**, *89*, 157–162.

(4) Zielonka, J.; Joseph, J.; Sikora, A.; Hardy, M.; Ouari, O.; Vasquez-Vivar, J.; Cheng, G.; Lopez, M.; Kalyanaraman, B. Mitochondria-Targeted Triphenylphosphonium-Based Compounds: Syntheses, Mechanisms of Action, and Therapeutic and Diagnostic Applications. *Chem. Rev.* **2017**, *117* (15), 10043–10120. https://doi.org/10.1021/acs.chemrev.7b00042.

(5) Torchilin, V. P. Recent Approaches to Intracellular Delivery of Drugs and DNA and Organelle Targeting. *Annu. Rev. Biomed. Eng.* **2006**, *8*, 343–375. https://doi.org/10.1146/annurev.bioeng.8.061505.095735.

(6) Horobin, R. W.; Trapp, S.; Weissig, V. Mitochondriotropics: A Review of Their Mode of Action, and Their Applications for Drug and DNA Delivery to Mammalian Mitochondria. *J. Control. Release* **2007**, *121* (3), 125–136. https://doi.org/10.1016/j.jconrel.2007.05.040.

(7) Ritchie, T. J.; Macdonald, S. J. F. The Impact of Aromatic Ring Count on Compound Developability - Are Too Many Aromatic Rings a Liability in Drug Design? *Drug Discov. Today* **2009**, *14* (21–22), 1011–1020. https://doi.org/10.1016/j.drudis.2009.07.014.

(8) Rossi, M.; Freschi, M.; De Camargo Nascente, L.; Salerno, A.; De Melo Viana Teixeira, S.; Nachon, F.; Chantegreil, F.; Soukup, O.; Prchal, L.; Malaguti, M.; Bergamini, C.; Bartolini, M.; Angeloni, C.; Hrelia, S.; Soares Romeiro, L. A.; Bolognesi, M. L. Sustainable Drug Discovery of Multi-Target-Directed Ligands for Alzheimer's Disease. *J. Med. Chem.* **2021**, *64* (8), 4972–4990. https://doi.org/10.1021/acs.jmedchem.1c00048.
